# Understanding the patterns and health impact of indoor air pollutant exposures in Bradford, UK: a study protocol

**DOI:** 10.1136/bmjopen-2023-081099

**Published:** 2023-11-30

**Authors:** Erika Ikeda, Jacqueline Hamilton, Chantelle Wood, Lia Chatzidiakou, Thomas Warburton, Athina Ruangkanit, Yunqi Shao, Denisa Genes, Dagmar Waiblinger, Tiffany C Yang, Chiara Giorio, Gordon McFiggans, Simon P O'Meara, Pete Edwards, Elizabeth Bates, David R Shaw, Roderic L Jones, Nicola Carslaw, Rosemary McEachan

**Affiliations:** 1 Bradford Institute for Health Research, Born in Bradford, Bradford Teaching Hospitals NHS Foundation Trust, Bradford, UK; 2 Department of Chemistry, Wolfson Atmospheric Chemistry Laboratories, University of York, York, UK; 3 Department of Psychology, The University of Sheffield, Sheffield, UK; 4 Yusuf Hamied Department of Chemistry, University of Cambridge, Cambridge, UK; 5 Department of Earth and Environmental Science, School of Natural Sciences, Centre for Atmospheric Science, The University of Manchester, Manchester, UK; 6 National Centre for Atmospheric Science, University of York, York, UK; 7 City of Bradford Metropolitan District Council, Bradford, UK; 8 Department of Environment and Geography, University of York, York, UK

**Keywords:** EPIDEMIOLOGIC STUDIES, Health Equity, Observational Study, PUBLIC HEALTH, QUALITATIVE RESEARCH, Surveys and Questionnaires

## Abstract

**Introduction:**

Relative to outdoor air pollution, there is little evidence examining the composition and concentrations of indoor air pollution and its associated health impacts. The INGENIOUS project aims to provide the comprehensive understanding of indoor air pollution in UK homes.

**Methods and analysis:**

‘Real Home Assessment’ is a cross-sectional, multimethod study within INGENIOUS. This study monitors indoor air pollutants over 2 weeks using low-cost sensors placed in three rooms in 300 Born in Bradford (BiB) households. Building audits are completed by researchers, and participants are asked to complete a home survey and a health and behaviour questionnaire, in addition to recording household activities and health symptoms on at least 1 weekday and 1 weekend day. A subsample of 150 households will receive more intensive measurements of volatile organic compound and particulate matter for 3 days. Qualitative interviews conducted with 30 participants will identify key barriers and enablers of effective ventilation practices. Outdoor air pollution is measured in 14 locations across Bradford to explore relationships between indoor and outdoor air quality. Data will be analysed to explore total concentrations of indoor air pollutants, how these vary with building characteristics, and whether they are related to health symptoms. Interviews will be analysed through content and thematic analysis.

**Ethics and dissemination:**

Ethical approval has been obtained from the NHS Health Research Authority Yorkshire and the Humber (Bradford Leeds) Research Ethics Committee (22/YH/0288). We will disseminate findings using our websites, social media, publications and conferences. Data will be open access through the BiB, the Open Science Framework and the UK Data Service.

**Strengths and limitations of this study**

- We collect detailed assessments of indoor air quality with 300 families in the longitudinal Born in Bradford birth cohort.
- Commercially available, low-cost air pollutant sensors are used to provide summaries of the most common pollutants coupled with survey measurements to capture building characteristics as well as occupant behaviours and health.
- Detailed assessments of volatile organic compounds and particulate matter using state of the art instrumentation and qualitative interviews are conducted in a subsample.
- To minimise participant burden, pollution measurements are collected over a 2 week period only.
- While one of the largest assessments of indoor air quality in UK homes to date, large sample sizes will be needed to confidently investigate associations with health outcomes.

## Introduction

Air pollution is thought to be responsible for between 28 000 and 36 000 deaths each year in the UK alone.^1^ Long-term exposure to outdoor air pollution has been associated with higher rates of cardiovascular and respiratory illness, birth-defects and neuro-degenerative disorders.^2^ Evidence derived from large-scale epidemiological studies indicate a causal relationship between exposure to outdoor air pollution and increased rates of mortality and morbidity.^3^ Most measurements, modelling and regulations of air pollution have so far focused on the outdoor environment.^4^ Equivalent research for indoor environments lags significantly behind, despite estimations that in developed countries such as the UK, we spend on average around 90% of our time indoors, with approximately two-thirds of this in our homes.^5^ This may be partly due to limitations in the availability, maintenance and cost of indoor air quality monitors, with nearly all studies of health effects having used data from fixed outdoor air pollution monitoring networks.^4^ This lack of data on indoor air pollution may result in inadequate exposure metrics and large uncertainties surrounding its sources and health impacts.

In the UK and globally, there is a dearth of evidence on the sources and transformations of indoor air pollutants.^6 7^ The limited evidence available shows that indoor air is a multidimensional issue influenced by the inter-relationship between indoor and outdoor air sources and transformation processes; building design, management and use; and human behaviour (eg, cooking, cleaning and ventilation).^6^ Since many of these dependencies are specific to location and social factors (eg, weather conditions, building regulations and cultural and social practices), comprehensive measurements with coordinated UK-specific modelling are needed for understanding indoor air quality relevant to UK homes. Without a fundamental understanding of how air pollution is caused, transformed and distributed in UK homes, research aiming to develop behavioural, technical or policy interventions to reduce future air pollution exposures may have little impact, or even be counterproductive.

The INGENIOUS (understandING the sourcEs, traNsformations and fate of IndOor air pollUtantS) project consists of seven linked work packages to understand the causes and concentration of, and health burden caused by, indoor air pollution (https://ingenious.york.ac.uk/). The current protocol describes research conducted with Born in Bradford (BiB) (https://borninbradford.nhs.uk/). For ease, we refer to this part of the project as the ‘Real Home Assessment’ study.

## Methods and analysis

### Research questions

Real Home Assessment is a cross-sectional, multimethod study which has four research questions:

1. What are the patterns of indoor air pollution in typical UK homes and are there inequalities in exposure to indoor air pollution?
2. How do physical characteristics of buildings or occupant behaviours contribute to indoor air pollution?
3. How are indoor air pollution and housing quality related to levels of respiratory symptoms and mental health?
4. What are the barriers and enablers of ventilation behaviours in the home?

Research questions 1–3 will be answered using quantitative data collected from air quality monitors and questionnaires from 300 homes over a 2 week period. Research question 4 will be answered using data from qualitative semi-structured interviews.

### Study design and setting

This study is conducted in Bradford which is an urban, multicultural city located in the North of England, UK. Bradford is the seventh largest metropolitan district in the UK with a population of >546 000.^8^ Approximately 67% of the population identify as White British and 20% as of Pakistani origin.^8^ About 34% of Bradford residents live in the most deprived neighbourhoods in England.^9^ The BiB is a prospective pregnancy and birth cohort which follows the health and well-being of over 12 500 families with children born in the city of Bradford, UK between 2007 and 2011.^10^ Half of the cohort are of South Asian origin, reflective of the demographic of young adults in this population (eg, more likely to be of childbearing age) within the city.^10^ The study began in August 2022 and will complete by July 2025.

### Participant characteristics and recruitment

A total of 300 families will be recruited from the BiB cohort who took part in the recent follow-up (2017–2020), BiB Growing Up study (in which BiB children were aged 7–11 years).^11^ The sample size of 300 was chosen based on pragmatic considerations including the intensive nature of data collection and the resources available to fund the study. Potential families were identified by stratifying the cohort by child ethnicity (ie, South Asian, White British and other), housing tenure (ie, private/mortgaged and rented) and presence of childhood asthma (ie, asthma: children had asthma symptoms up to 2 years before the Growing Up data collection and non-asthma) to maximise the variability and relevance of the sample at the local and national levels (table 1). To be eligible to participate in the Real Home Assessment study, a parent needs to be able to give informed consent; a household suitable to instal indoor air quality monitors; and a family able to complete questionnaires and diaries. A family is excluded from the study if a parent is unable to give informed consent and/or communicate in English.

**Table 1:** The recruitment target of 300 BiB families stratified by child ethnicity, housing tenure and childhood asthma

		Housing tenure			
		Private/mortgaged property		Rented property	
		(n=210; 70%)		(n=90, 30%)	
		Asthma (50%)	Non-asthma (50%)	Asthma (50%)	Non-asthma (50%)
Ethnicity	South Asian	n=48; 16.0%	n=47; 15.7%	n=20; 6.7%	n=20; 6.7%
	(n=135; 45%)				
	White British	n=48; 16.0%	n=47; 15.7%	n=20; 6.7%	n=20; 6.7%
	(n=135; 45%)				
	Other	n=10; 3.3%	n=11; 3.7%	n=5; 1.7%	n=4; 1.3%
	(n=30; 10%)				

BiB, Born in Bradford.

Recruitment of the 300 families began in March 2023 with final visits due to occur in April 2024. Subsamples of 150 and 30 (out of 300) families are further invited for additional assessments of indoor air pollution and qualitative interviews, respectively (see Procedure).

### Procedure

**Figure 1:**
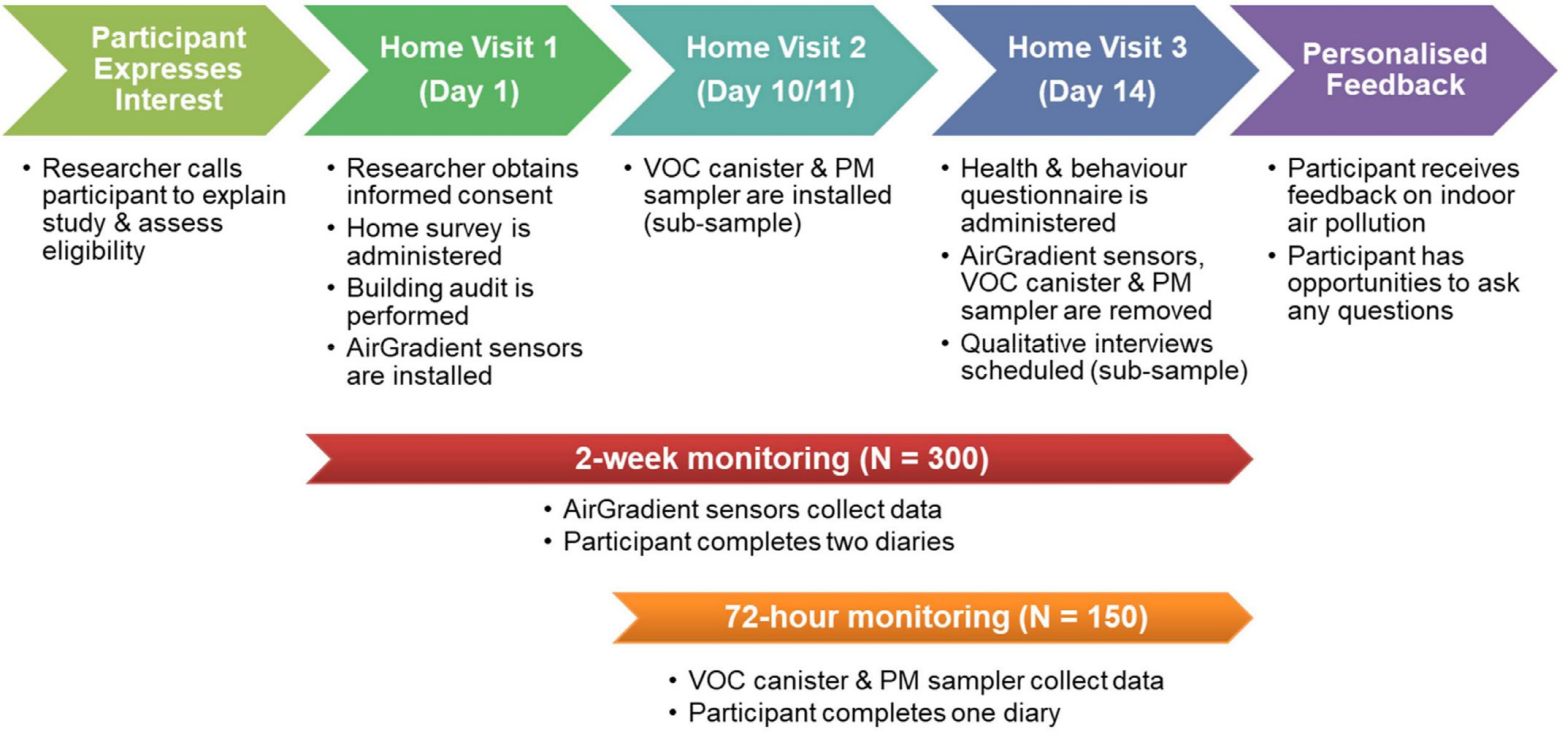
The procedure of recruitment and data collection. PM, particulate matter; VOC, volatile organic compound.

**Figure 2:**
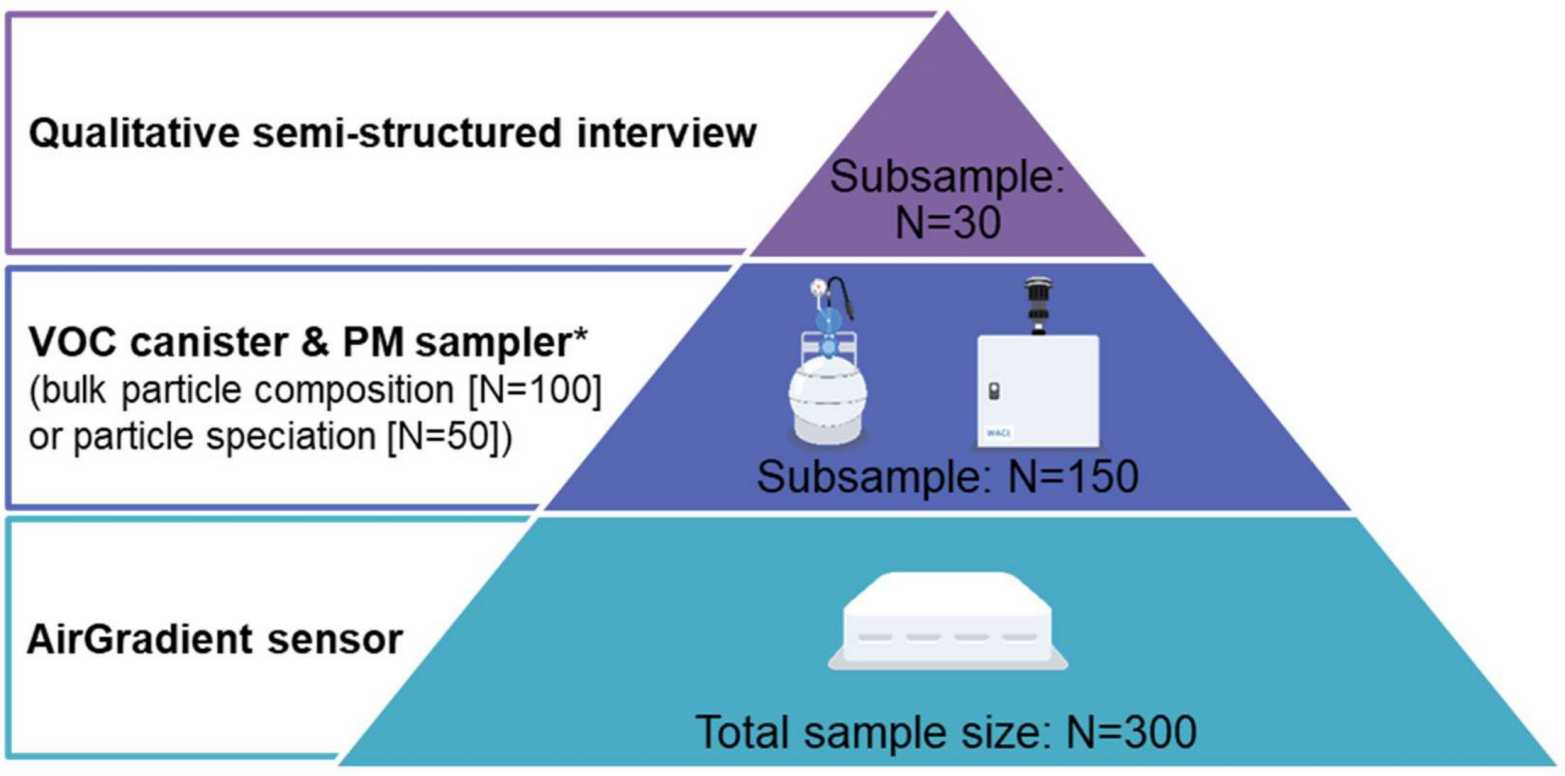
Tiered indoor air quality assessment. *150 filters collected from PM samplers are analysed for either bulk particle chemical composition or detailed particle speciation. PM, particulate matter; VOC, volatile organic compound.

Figure 1 outlines the procedure of recruitment and data collection which involves placing air quality monitors (including AirGradient sensors, volatile organic compound (VOC) canister and particulate matter (PM) sampler) in participants’ homes and conducting survey measurements and qualitative interviews over a 2 week period. We deploy a tiered approach to indoor air quality assessment, and the sample size is selected to maximise breadth of data and be deliverable within the funded project timelines (figure 2).

Eligible participants who have previously given consent to be contacted by BiB receive a phone call from the research team for recruitment. Participants are provided with the aim of the study and an overview of the study timeline and data collection. If the family is willing to take part in the study, a home visit is scheduled at a convenient time. Participant information sheets are provided electronically via email to participants before, or in person during the first home visit where written informed consent is obtained and the participants have the opportunity to ask questions.

Data collection occurs over 14 days with two home visits. During the first home visit (day 1), a member of the research team places a portable Wi-Fi hub at the participant’s property, and installs three low-cost (ie, AirGradient) sensors, which are connected to the Wi-Fi hub in three rooms (where participants are more likely to spend time and pollutants are likely to be generated): a living room, a kitchen and a child’s bedroom (or the room where a BiB child sleeps).^12^ The researcher then conducts a building audit to identify key characteristics of the property, and the participant completes a home survey which assesses the condition of their house. Instructions are given by members of the research team on how to complete the provided activity and symptom diaries over the next 2 weeks. On the same visit, a subsample of participants are also invited to take part in a 3 day period of more intensive monitoring of VOCs in a living room and PM in a kitchen which would take place at the end of the 2 week period (figure 2). Among the families agreeing to take part in additional VOC and PM monitoring, an appointment is agreed between the research team and participant for an additional home visit (preferably on day 10 or 11) to install a VOC canister and a PM sampler and provide another set of diaries. In the PM samplers, the filters are used for either bulk particle chemical composition (n=100) or detailed particle speciation (n=50) analyses (figure 2).

On day 14, the research team returns to collect the air quality monitors and diaries. The participant completes a questionnaire on the health and behaviours of the participant and their child(ren) over the past 2 weeks. The subsample of families who complete the additional VOC and PM monitoring are further invited for a qualitative interview exploring barriers and enablers to ventilation behaviours in the home. A total of 30 semi-structured interviews are conducted online or in-person after obtaining informed consent.

Following the completion of the data monitoring period, participants are provided with a personalised feedback report outlining the levels of air pollution in their home measured based on the sensors’ readings, and are given the opportunity to ask questions about the study. All participants receive a £50 voucher in recognition of their time and effort, and those participating in the interview receive an additional £20 voucher.

### Indoor air quality measurements

#### AirGradient sensor

**Figure 3:**
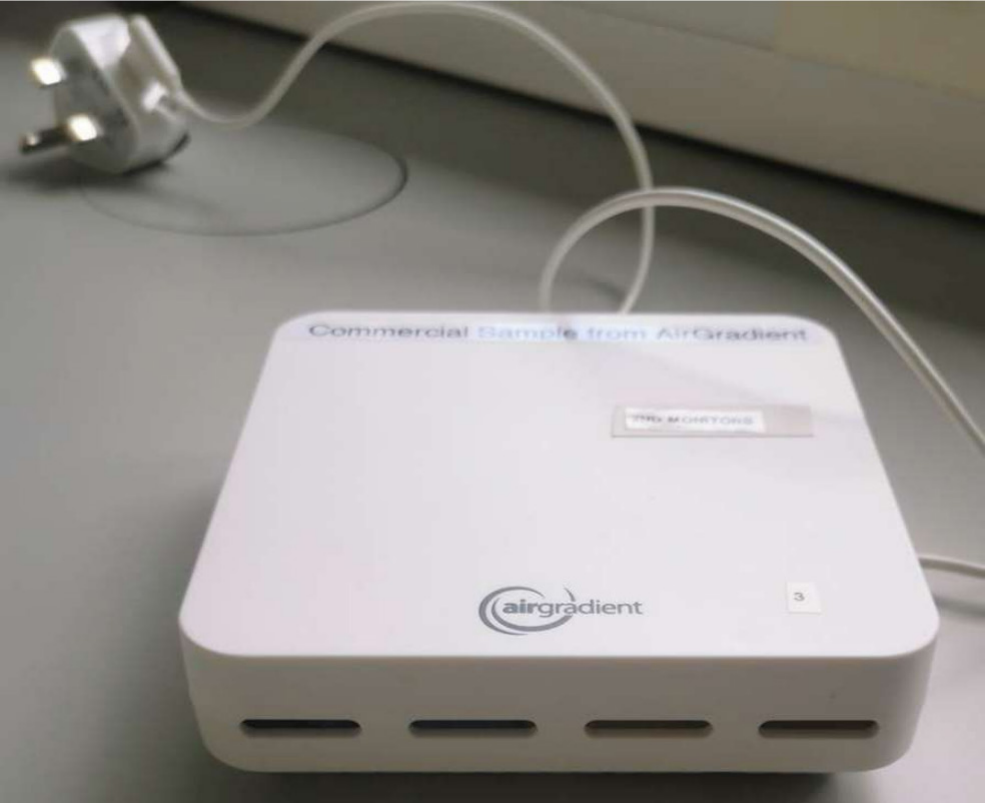
Image of the AirGradient sensor.

All homes receive commercially available low-cost sensors, AirGradient^13^ that measures (1 min resolution) and transmits (5 min average intervals) air pollution data to secure server through a Wi-Fi hotspot provided by the research team. The sensor was selected based on its performance in validation tests, ease of use, size, robustness, price and low noise operation to minimise disturbance of occupants (figure 3). The AirGradient contains a series of sensors: a SenseAir S8 carbon dioxide (CO_2_) sensor using non-dispersive infrared technology to measure CO_2_ concentration (parts per million by volume (ppm)); a Plantower PMS5003 sensor with laser scattering technology to measure three size fractions of PM concentration (PM_1_, PM_2.5_, PM_10_ in micrograms per cubic metre (µg/m^3^)); a Sensirion SGP41 total volatile organic compound (TVOC)/nitrogen oxides (NOx) sensor to measure TVOC concentration (parts per billion by volume (ppb)) and a Sensirion SHT3x/4x sensor to measure temperature and relative humidity. Where possible, the sensors are placed on a table or shelf and away from external walls and windows^14^ in a living room, kitchen and BiB child’s bedroom while minimising interference with normal occupant behaviours.

#### VOC canister

**Figure 4:**
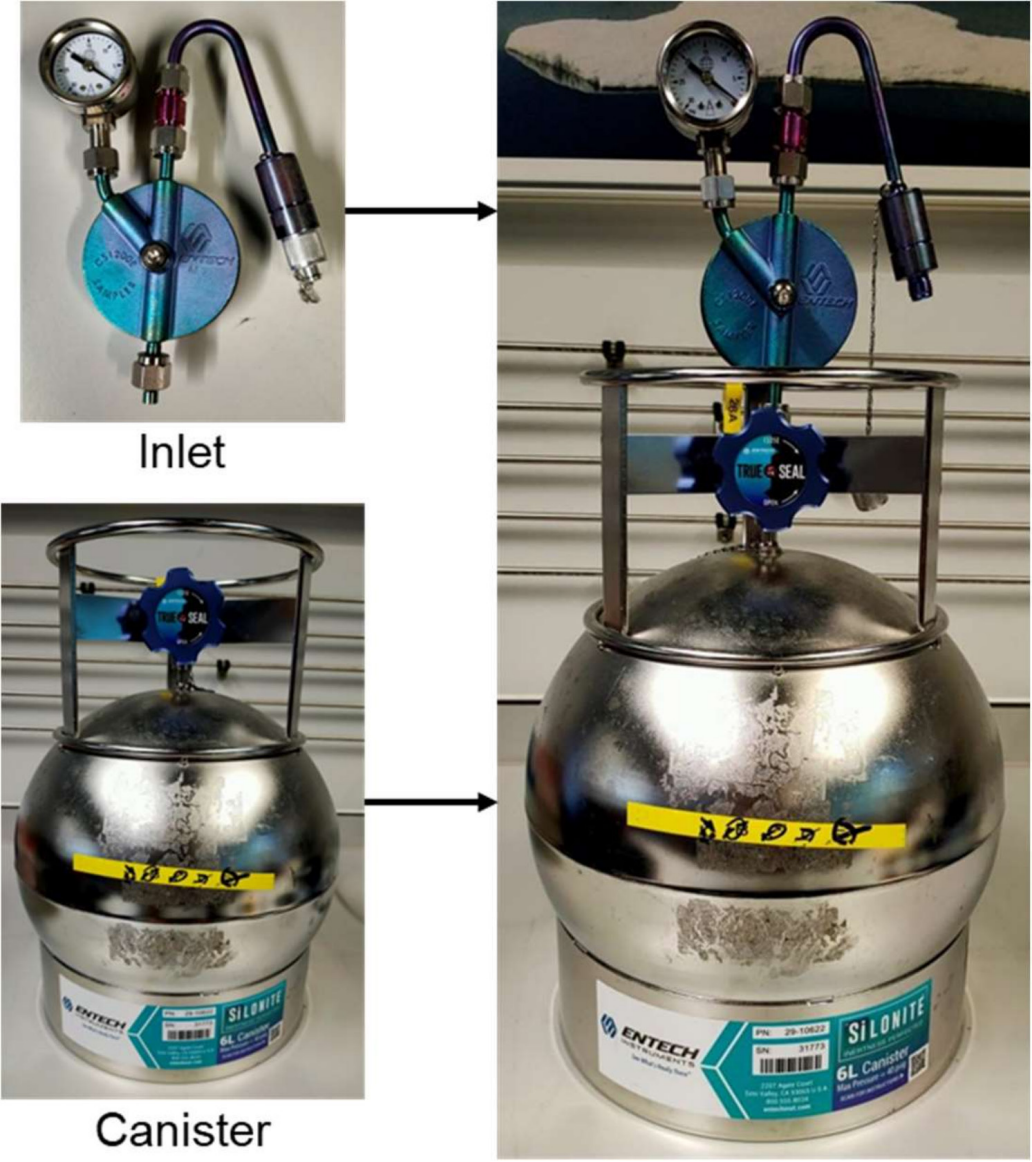
Images of the Entech 6 litre canister and an inlet.

In a subsample of 150 homes, a VOC canister collects air over a 3 day period. Six-litre vacuum-intake stainless-steel canisters from either Entech (CA, USA) or Restek (PA, USA) are used, and a flow-restrictive inlet is attached (figure 4). Prior to deployment, each canister is evacuated with a pressure below 0.01 Pa to ensure there is no residual ambient air in the canister. During the deployment, the research team attaches a flow-restrictive inlet onto a canister and places the canister on the floor within the living space and away from external walls and windows^14^ while minimising interference with normal occupant behaviours. The canister air sample collected from each home is processed off-line in a laboratory, using a custom thermal desorption unit linked to a two-column Agilent 7890A gas chromatograph utilising flame ionisation detection and an Agilent 5977A quadrupole mass spectrometer (GC-FID-QMS).^15^ A broad range of compounds of different molecular sizes and chemical functionality are targeted including ethane from natural gas, butane from aerosol spray products, monoterpenes from fragrances and ethanol from solvent use.

#### PM sampler

**Figure 5:**
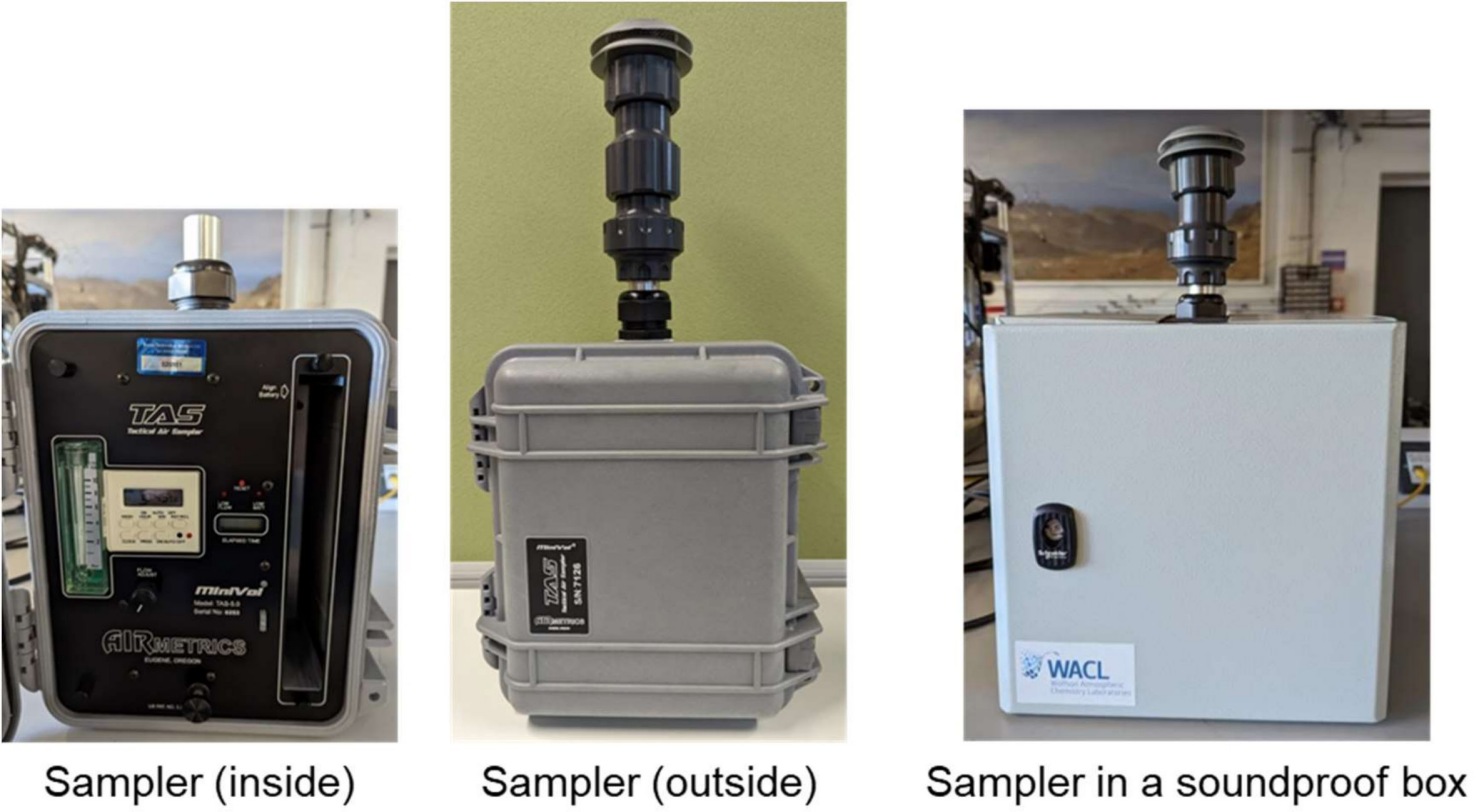
Images of the Minivol Tactical Air Sampler and a soundproof box.

The same participant subsample receiving a VOC canister (n=150) also host samplers to collect PM onto filter papers for analysis using advanced analytical instrumentation. A Minivol Tactical Air Sampler with a PM_2.5_ impactor protruding from the top is used to collect PM less than 2.5 microns in diameter onto pre-based 47 mm filter papers (figure 5). The sampler draws air through the polytetrafluoroethylene filter using a pump set at 3 L·min^−1^ over the course of approximately 72 hours. The exact sample time is recorded by the research team who starts and stops the sampling process. To minimise the sound from the pump, the sampler is enclosed by a custom-built metal box lined with sound proofing foams (Dodo Sound Stopper Pro v2) (see figure 5). The soundproofing enclosure reduced the sound emitted by 10 dBA to a final sound level of 50 dBA. Where possible, the sampler is placed on a kitchen countertop or table and away from windows.^14^ Out of 150 filter samples, two-thirds (n=100) are analysed to characterise the bulk chemical composition of particles. A 25 mm punch is excised from the 47 mm filter to subsequently analyse organic aerosol composition using a time-of-flight chemical ionisation mass spectrometer with iodide ionisation coupled with a filter inlet for gases and aerosols (FIGAERO-CIMS).^16 17^ An appropriate fraction of residual filter material will be extracted using methanol and aerodyne high-resolution time-of-flight aerosol mass spectrometer (HR-ToF-AMS)^18^ to analyse both bulk inorganic and organic chemical composition. One-third (n=50) of the filter samples collected are analysed to obtain the concentrations of specific chemical compounds that are tracers of specific PM sources and/or are known to have toxic effects in humans. The filters are extracted using accelerated solvent extraction and analysed using two-dimensional gas chromatography coupled with time-of-flight mass spectrometry (GC×GC-ToF-MS).^19^

### Outdoor air quality monitoring

In collaboration with the City of Bradford Metropolitan District Council, outdoor air pollutants (PM_10_, PM_2.5_, NO_X_, ozone [O_3_], CO_2_) are being measured using Earthsense Zephyr^20^ devices in 10 locations across the Bradford city since June 2023. In addition, four AQMesh^21^ units which measure the same target pollutants as the Zephyr devices have been deployed in the residential areas of Bradford since July 2023, following a 2 month collection period with reference devices at the Manchester Air Quality supersite. The data from both devices will be combined with those from local reference monitors to estimate outdoor air pollution in the vicinity of participants’ homes, and thus examine the influence of indoor and outdoor air exchange on indoor air pollutant measurements.

### Survey measurements

Table 2 summarises the four surveys (ie, home survey, building audit, health and behaviour questionnaire, activity and symptom diary) which are available in online supplemental files 1 and 2. These measurements have been developed based on validated questionnaires, existing evidence and input from experts in indoor air quality. Considering participant burden, these measurements (except the diary) are primarily administered online using REDCap (Research Electronic Data Capture),^22^ which potentially reduces the amount of time taken to complete the measurements (due to branching/skip logic) and aids understanding of questions (due to visuals), compared with the paper-and-pencil version. The online platform also reduces researcher burden and errors (entering and managing data).

10.1136/bmjopen-2023-081099.supp1 Supplementary data

**Table 2:** Summary of surveys

	Home survey	Building audit	Health and behaviour questionnaire	Activity and symptom diary
Completed on	Day 1	Day 1	Day 14	Household behaviour: 1 weekday and 1 weekend day (n=300); 1 day during the 72-hour monitoring period (n=150) Health symptoms (n=300): days 1–14 Meals cooked at home (n=150): days 10/11–14
Completed by	Participant	Researcher	Participant	Participant
Content	Section I: Home	Section I: House	Section I: Your perception of your home and air quality	Household behaviour
	Residence at the address	Property type and age	Home satisfaction	Cooking
	Housing tenure	Building level	Air quality perception	Cleaning
	Outdoor environment	Energy Performance Certificate (EPC) rating	Section II: You/your household’s behaviour at home	Products
	Number, type and state of rooms	Outdoor environment	Meal time	Heating
	Presence of mould and damp	Number of windows and doors leading to the outside	Use of non-stick cookware, personal care products, fragrances and cleaning products	Ventilation
	Heating type	Sensor ID and location	Frequency of dusting and vacuuming	Personal care
	Cooking fuel type	Section II: Room	Method and location of washing and drying clothes	Occupancy
	Ventilation type	Room size	Use of solid fuel appliance	Other experiences related to air quality
	Presence of chimney	Floor material	Section III: Health	Individual* health symptom
	Home occupancy	Widow direction	Asthma (BiB child and parent)	Time at home
	Pet ownership	Use of curtains/blinds/shutters	Rhinitis (BiB child and parent)	Asthma
	Use of air purifier		Eczema (BiB child and parent)	Rhinitis
	Section II: Behaviour		Behavioural screening (BiB child)	Eczema
	Smoking habit		Mental well-being (BiB parent)	Mood
			Section IV: About you and your household	Additional measurements
			Household size	Type of main meals cooked
			Food security	
			Financial security	

*Members of the family (eg, BiB mother and BiB child).

BiB, Born in Bradford.

#### Home survey

The 40-item home survey collects information on the condition and characteristics of the participant’s home, the outdoor environment around the home and people’s smoking behaviours inside and outside the home (online supplemental file 1). The survey is self-administered electronically using a tablet or on paper by a participant during the first visit. The survey takes approximately 10 min to complete.

#### Building audit

The 32-item building audit collects information on the type of property and the characteristics of the rooms in which the sensors are installed known to affect indoor levels of air pollution such as room size, presence and placement of windows, and presence of extractor fans or hoods (online supplemental file 1). The research team completes the audit electronically using a tablet or on paper during the first visit. The audit takes approximately 10 min to complete.

#### Health and behaviour questionnaire

The 250-item health and behaviour questionnaire assesses individual symptoms of child and parent respiratory and allergic health (International Study of Asthma and Allergies in Childhood (ISAAC);^23^ Global Asthma Network^24^), child behavioural screening (Strengths and Difficulties Questionnaire^25 26^) and parent mental health (Short Warwick-Edinburgh Mental Well-being Scale;^27^ Patient Health Questionnaire—Depression Module^28^), as well as socioeconomic circumstances and household behaviours such as ventilation and cooking habits (online supplemental file 1). A participant completes the questionnaire electronically using a tablet or on paper on day 14. The questionnaire takes approximately 20 min to complete.

#### Activity and symptom diary

Paper-based diaries are used to record key household activity patterns (28 items) and 14 individual health indicators of respiratory and allergic symptoms (based on ISAAC^23^) and mood (based on Visual Analogue Mood Scale^29^ (online supplemental file 2). Individual health symptoms are self-reported by members of the family (eg, BiB mother and BiB child) every day during the 2 week period. For household activity patterns, the participants are asked to select 1 weekday and 1 weekend day to record time spent doing key household activities that are related to indoor air pollution (eg, cooking, cleaning and ventilation), and provide a description of relevant activities using free text. For the subset of participants who host a VOC canister and a PM sampler (n=150), activities are recorded for one additional day and main meals cooked at home are recoded every day during the 72-hour monitoring period.

### Qualitative interview

Content for the semi-structured interviews was developed based on the COM-B (Capacity, Opportunity, Motivation-Behaviour) Model,^30^ which provides a systematic framework for understanding behaviours in terms of people’s physical and psychological capability: physical and social opportunity; and automatic and reflexive motivation. The interview takes approximately 60 min, and is conducted either face-to-face or online, depending on the participant’s preference, and will be audio-recorded and transcribed. An interview guide is available on the Open Science Framework.^31^

### Data analysis plan

Quantitative data from the air quality monitors (ie, the concentration of pollutants and compounds) and survey measurements will be reported descriptively using frequencies (for categorical variables), or medians and IQR or means and SD (for continuous variables). Any qualitative data from the survey measurements (ie, text) will be used as supplementay information for air pollution data. Any missing data of the variables measured and reasons for their absence will be examined, reported and handled appropriately in analyses. We do not expect any missing sociodemographic data as the data have already been collected through the BiB cohort study (eg, Growing Up^11^). Based on the research questions, we will select appropriate statistical methods to analyse the data, as specified below.

#### What are the patterns of indoor air pollution in typical UK homes and are there inequalities in exposure to indoor air pollution?

Descriptive analyses of the AirGradient sensor data will be performed, including examining daily concentrations of indoor air pollution (means or medians), peaks in exposure and daily time spent above the WHO or UK recommendations for air pollution.^32–34^ Compounds with known toxicological effects will be highlighted and compared with the WHO or UK guidelines for indoor air quality.^32–34^ All the air pollution data including CO_2_, VOCs and PM (concentration and composition) will be examined with all participants together as well as stratified by housing tenure (ie, private/mortgaged and rented), ethnicity (ie, South Asian, White British and other) and Indices of Multiple Deprivation (IMD; in quintiles) in England (national) and Bradford (local). Differences within each variable will be examined using independent t-test, analysis of variance or Wilcoxon-Mann-Whitney test.

#### How do physical characteristics of buildings or occupant behaviours contribute to indoor air pollution?

To identify subgroups of the participants based on shared factors, we aim to (1) identify latent profiles of building characteristics and occupant behaviours using latent class analysis, and (2) examine whether these profiles are associated with indoor air pollution using mixed effect models, adjusting for covariates including housing tenure, ethnicity and English or Bradford IMD using regression models.

#### How are indoor air pollution and housing quality related to levels of respiratory symptoms and mental health?

Mixed-effects models will be used to examine the relationship between changing indoor air pollution levels on respiratory symptoms and mood. The exposure variable will be measured as continuous (eg, the daily and 2 week average concentration of indoor air pollution) or binary (eg, the daily amount of time pollution levels exceeded the WHO or UK guidelines for indoor air quality^32 34^). Daily occurrences of respiratory symptoms (binary outcome: yes/no) and daily measures of mood (continuous outcome: 1–10) assessed using the symptom diaries will be examined using mixed-effects logistic regression and mixed-effects linear regression, respectively. Multivariable logistic regression, adjusting for relevant confounding variables, will be used to explore the association between indoor air pollution (ie, 2 week average concentrations) and the health outcomes (ie, the state of respiratory health and mental well-being derived from the health and behaviour questionnaire). Covariates will be included to reduce confounding and improve precision including subject-specific (eg, age, sex, ethnicity, British or Bradford IMD and smoking habits) and time-dependent (eg, day of the week, season, temperature and relative humidity).

To examine the lag relationship over time between exposure (indoor air pollution) and outcomes (respiratory symptoms and mood), we will assess the same day (lag 0), 1 day, 2 day and 3 day lags as well as the average of lag 0–3 days. Potential effect modification by housing quality (eg, housing tenure and presence of damp) and season will be examined.

#### What are the barriers and enablers of ventilation behaviours in the home?

Anonymised interview transcripts will be coded and analysed using NVivo qualitative data analysis software^35^ or paper-based techniques. A hybrid deductive/inductive content and thematic analysis approach will be employed, based on the methods described by Atkins *et al*,^36^ Smith *et al* 37 and Prothero *et al*.^38^ This will consist of two phases which entail development of an initial codebook based on the determinants of behaviour (ie, domains) specified in the Theoretical Domains Framework (TDF),^39^ mapped onto the COM-B framework.^30^ In phase 1, 10% of the transcripts will be independently deductively content analysed by two researchers, by coding text to the TDF domains in the codebook. We will also retain flexibility to identify additional codes not encompassed by the TDF during this stage, by adding these to the codebook where relevant. Coding will then be discussed by the two researchers, with the aim of building consensus, and refining the codebook. Where consensus cannot be reached, a third researcher will be consulted, or the relevant text will be attributed to more than one TDF domain. The first researcher will then use the revised codebook to code the remainder of the transcripts, adding and discussing any revisions or additional codes with the second researcher or broader team as needed.

In phase 2, an inductive thematic analysis will be conducted on the text marked with codes during phase 1, following the process identified by Braun and Clarke^40^ and focused on identifying specific barriers and enablers of ventilation behaviours. Ten per cent of transcripts will be independently coded by two researchers, and an initial list of inductive codes will be added to each researcher’s codebook under the code headings from phase 1. The first researcher will then use the revised codebook to code the remainder of the transcripts, adding and discussing any revisions or additional codes with the second researcher or broader team as needed. Themes will then be generated, reviewed and defined by the first researcher, in discussion with the second researcher or broader team. Content relating to current ventilation behaviours will be summarised descriptively. Potential targets for intervention will then be prioritised by the first researcher in discussion with the broader team, based on the following criteria: (1) frequency of theme (ie, number of participants reporting a specific barrier or enabler); (2) number of barrier or enabler themes per TDF domain (where relevant); (3) discordance within or across themes (ie, the same factor acting as a barrier and enabler of behaviour in different participants or in different contexts) and (4) perceived importance of theme for participants.

### Patient and public involvement

In Real Home Assessment, public involvement has been central to the development of the study and has been embedded within all research activities including the design, conduct and dissemination of the study. BiB has long-established public participation groups (eg, parent governors and public research advisory group) which meet regularly to provide lived experience, expertise and guidance to the research team. The groups, for example, provided us feedback on the feasibility of the survey measurements (eg, item wording and survey length) during the pilot testing. We have used a variety of different channels to give members of the public a chance to engage in the air pollution research, including hosting regular ‘open-space’ community meetings, attending existing community events (eg, science festivals), and circulating briefing notes and newsletters. For study participants, we provide a personalised feedback report (online supplemental file 3) after the completion of the data monitoring period.

### Ethics and dissemination

Ethical approval has been obtained from the National Health Service Health Research Authority Yorkshire and the Humber (Bradford Leeds) Research Ethics Committee: 22/YH/0288, 11 January 2023. Participants give informed consent by receiving an information sheet and signing a consent form after they have had the opportunity to discuss the study with a member of the research team. We will disseminate research outputs using various channels including our INGENIOUS project website,^41^ BiB website,^42^ social media, scientific publications, press releases as well as conference presentations and posters. Air quality and health data will be open access via existing BiB data access procedure.^42^ An open-access data set for the qualitative interviews (ie, anonymised transcripts) will be also made available on the UK Data Service, and the interview materials are available on the Open Science Framework.^31^

## Discussion

Exposure to airborne pollutants during childhood can increase the risk and exacerbation of airway diseases with lifelong consequences.^43 44^ Yet, there is little evidence to constrain or quantify indoor air pollutant emissions, building-to-building variability, chemical speciation of indoor air pollutants, interactions between indoor-generated and outdoor-generated air pollution, or environmental and socioeconomic factors that can contribute to elevated indoor air pollutant exposures. Real Home Assessment as part of the INGENIOUS project is one of the first research studies that measures indoor air pollution in UK homes at a large scale. Findings from the Real Home Assessment will contribute to the understanding of how indoor air pollution in UK homes is generated, prevented and reduced, as well as insights into social, ethnic and health inequalities associated with indoor air pollution.

Deprived households are more likely to experience poor indoor air quality than more affluent households due to factors such as greater exposure to second-hand smoke and higher outdoor air pollutant concentrations.^2^ However, the reality may be considerably more nuanced. On one hand, lower quality and older housing may be less airtight (ie, less energy efficient) than new-build homes allowing outdoor air pollution to penetrate inside but indoor emissions to escape more easily. On the other hand, large, expensive town houses converted to flats can be poorly ventilated, and suffer from higher indoor-generated air pollutant concentrations following poor retrofitting practices.^2^

Recruiting households from the BiB cohort in the current study is valuable in terms of the diversity of the cohort (covering a multiethnic population that has high rates of deprivation) and the detailed data collection (including information about the health, social circumstances and lifestyle characteristics of children). Over 30% of BiB families live in a rented property, one-third of families have more than five occupants, and 13% of families have at least one child with doctor-diagnosed asthma (based on the Growing Up data^11^). This profile makes the BiB cohort a particularly relevant population group in which to explore patterns of indoor air pollution.

The tiered approach of air quality monitoring allows us to provide both summary (with common pollutant concentrations) and detailed (with a large number of pollutant speciation) assessments of indoor air quality. Indoor air quality and health is an under-researched area, and this study will be one of the largest to date and will collect detailed measures of indoor air pollution and health outcomes.^45^ Along with the air quality monitors, we use four survey measurements to capture potential contributing emission sources of indoor air pollution from the building and occupant behaviours. Results from the semi-structured interviews will be used to inform the design of behaviour change interventions to reduce exposure to indoor air pollutants. These interventions will be co-designed with community members and evaluated in a later stage of the INGENIOUS project and will be described in a separate protocol.

Real Home Assessment is a cross-sectional study which captures a snapshot of indoor air pollutant concentrations and occupant behaviours and health symptoms over a 2 week period. The sample size of 300 families was selected to maximise the breadth of data and be deliverable within the funded project timelines. While this will be one of the largest studies of indoor air pollution in UK homes to date, it will still be underpowered to definitively investigate associations between indoor air quality and health outcomes. Given the burdensome nature of data collection, it will be important for researchers in this area to harmonise methods to allow combining data from similar studies across the UK. We publish this protocol and share our methods as a first step in this wider endeavour. Recruiting participants and collecting data (including air pollutant concentrations and occupant behaviours) in the domestic setting can be more challenging and resource-intensive than doing so in public spaces and measuring outdoor air pollution through national and local authorities.^7^ Thus, we consider Real Home Assessment as exploratory research to assess the feasibility of collecting indoor air pollution in homes, which is likely to be the primary indoor environment where most people spend their time. However, we cannot ignore air pollution exposures in other indoor settings such as schools, workplaces, transport and shops. Future research will aim to capture total personal exposure using multipollutant sensor platforms^46^ and advanced computational modelling for time-activity patterns.^47^

## Supplementary Material

Reviewer comments

Author's manuscript

## References

[1] Office for Health Improvement & Disparities . Air pollution: applying all our health. 2022. Available: https://www.gov.uk/government/publications/air-pollution-applying-all-our-health/air-pollution-applying-all-our-health [Accessed 12 Sep 2023].

[2] Laverge J , Delghust M , Janssens A . Carbon dioxide concentrations and humidity levels measured in Belgian Standard and low energy dwellings with common ventilation strategies. International Journal of Ventilation 2015;14:165–80. 10.1080/14733315.2015.11684078

[3] Cohen AJ , Brauer M , Burnett R , et al . Estimates and 25-year trends of the global burden of disease attributable to ambient air pollution: an analysis of data from the global burden of diseases study 2015. Lancet 2017;389:1907–18. 10.1016/S0140-6736(17)30505-6 28408086 PMC5439030

[4] Dominski FH , Lorenzetti Branco JH , Buonanno G , et al . Effects of air pollution on health: A mapping review of systematic reviews and meta-analyses. Environ Res 2021;201:111487. 10.1016/j.envres.2021.111487 34116013

[5] Klepeis NE , Nelson WC , Ott WR , et al . The National human activity pattern survey (NHAPS): A resource for assessing exposure to environmental Pollutants. J Expo Anal Environ Epidemiol 2001;11:231–52. 10.1038/sj.jea.7500165 11477521

[6] Department of Health and Social Care . Chief Medical Officer’s annual report 2022: Air pollution. 2022.

[7] Air Quality Expert Group . Indoor air quality. 2022. 10.5281/zenodo.6523605

[8] City of Bradford Metropolitan District Council . Population. 2022. Available: https://ubd.bradford.gov.uk/about-us/population/

[9] Noble S , McLennan D , Noble M , et al . The English indices of deprivation 2019: research report; 2019.

[10] Wright J , Small N , Raynor P , et al . Cohort profile: the born in Bradford multi-ethnic family cohort study. Int J Epidemiol 2013;42:978–91. 10.1093/ije/dys112 23064411

[11] Bird PK , McEachan RRC , Mon-Williams M , et al . Growing up in Bradford: protocol for the age 7-11 follow up of the born in Bradford birth cohort. BMC Public Health 2019;19:939. 10.1186/s12889-019-7222-2 31300003 PMC6626420

[12] Royal College of Physicians . Every breath we take: The lifelong impact of air pollution. London, UK, 2016.

[13] AirGradient Limited . Airgradient. 2023. Available: https://www.airgradient.com [Accessed 22 Sep 2023].

[14] The Chartered Institution of Building Services Engineers (CIBSE) . TM61 Operational performance of buildings. London, UK, 2020.

[15] Warburton T , Grange SK , Hopkins JR , et al . The impact of plug-in fragrance diffusers on residential indoor VOC concentrations. Environ Sci: Processes Impacts 2023;25:805–17. 10.1039/D2EM00444E 36883522

[16] Lopez-Hilfiker FD , Mohr C , Ehn M , et al . A novel method for online analysis of gas and particle composition: description and evaluation of a filter inlet for gases and aerosols (FIGAERO). Atmos Meas Tech 2014;7:983–1001. 10.5194/amt-7-983-2014

[17] Lee BH , Lopez-Hilfiker FD , Mohr C , et al . An iodide-adduct high-resolution time-of-flight chemical-Ionization mass spectrometer: application to atmospheric inorganic and organic compounds. Environ Sci Technol 2014;48:6309–17. 10.1021/es500362a 24800638

[18] Jimenez JL , Jayne JT , Shi Q , et al . Ambient aerosol sampling using the Aerodyne aerosol mass spectrometer. J Geophys Res 2003;108:1–13. 10.1029/2001JD001213

[19] Stewart GJ , Nelson BS , Acton WJF , et al . Emissions of intermediate-volatility and semi-volatile organic compounds from domestic fuels used in Delhi, India. Atmos Chem Phys 2021;21:2407–26. 10.5194/acp-21-2407-2021

[20] EarthSense Systems Limited . Zephyr. 2023. Available: https://www.earthsense.co.uk/zephyr [Accessed 22 Sep 2023].

[21] Environmental Instruments Limited . Aqmesh. 2023. Available: https://www.aqmesh.com [Accessed 22 Sep 2023].

[22] Vanderblit University . Redcap. 2023. Available: https://projectredcap.org [Accessed 27 Sep 2023].

[23] Ellwood P , Asher MI , Beasley R , et al . Phase three Manual of the International study of asthma and allergies in childhood (ISAAC). Int J Tuberc Lung Dis 2000;9:10–6. 15675544

[24] Global Asthma Network . Phase I Manual: Global surveillance: prevalence, severity, management and risk factors. Auckland, New Zealand, 2015.

[25] Goodman R . The strengths and difficulties questionnaire: A research NOTE. J Child Psychol Psychiatry 1997;38:581–6. 10.1111/j.1469-7610.1997.tb01545.x 9255702

[26] Goodman R . The extended version of the strengths and difficulties questionnaire as a guide to child psychiatric Caseness and consequent burden. J Child Psychol Psychiatry 1999;40:791–9. 10433412

[27] Shah N , Cader M , Andrews B , et al . Short Warwick-Edinburgh mental well-being scale (SWEMWBS): performance in a clinical sample in relation to PHQ-9 and GAD-7. Health Qual Life Outcomes 2021;19:260.:260. 10.1186/s12955-021-01882-x 34819104 PMC8611866

[28] Kroenke K , Spitzer RL , Williams JBW . The PHQ-9: validity of a brief depression severity measure. J Gen Intern Med 2001;16:606–13. 10.1046/j.1525-1497.2001.016009606.x 11556941 PMC1495268

[29] van Rijsbergen GD , Bockting CLH , Berking M , et al . Can a one-item mood scale do the trick? predicting relapse over 5.5-years in recurrent depression. PLoS One 2012;7:e46796. 10.1371/journal.pone.0046796 23056456 PMC3463530

[30] Michie S , van Stralen MM , West R . The behaviour change wheel: A new method for Characterising and designing behaviour change interventions. Implement Sci 2011;6:42. 10.1186/1748-5908-6-42 21513547 PMC3096582

[31] Wood C , Genes D . INGENIOUS: Co-production and evaluation of behaviour change interventions to improve indoor air quality in the UK, . 2023 Available: https://osf.io/sf9np/?view_only=76c443e757f84ecbb16f6705ebe0766a [Accessed 6 Oct 2023].

[32] World Health Organization . WHO guidelines for air quality: selected pollutants. Copenhagen, Denmark, 2010. 23741784

[33] World Health Organization . WHO global air quality guidelines: Particulate matter (PM2.5 and PM10), ozone, nitrogen dioxide, sulfur dioxide and carbon monoxide. Geneva, Switzerland, 2021. 34662007

[34] Public Health England . Indoor Air Quality Guidelines for selected Volatile Organic Compounds (VOCs) in the UK About Public Health England. London, UK, 2019.

[35] Lumivero . Nvivo. 2023. Available: https://lumivero.com/products/nvivo [Accessed 6 Oct 2023].

[36] Atkins L , Francis J , Islam R , et al . A guide to using the theoretical domains framework of behaviour change to investigate implementation problems. Implement Sci 2017;12:77.:77. 10.1186/s13012-017-0605-9 28637486 PMC5480145

[37] Smith D , Cartwright M , Dyson J , et al . Barriers and Enablers of recognition and response to deteriorating patients in the acute hospital setting: A theory-driven interview study using the theoretical domains framework. J Adv Nurs 2021;77:2831–44. 10.1111/jan.14830 33739478

[38] Prothero L , Lawrenson JG , Cartwright M , et al . Barriers and Enablers to diabetic eye screening attendance: an interview study with young adults with type 1 diabetes. Diabet Med 2022;39:e14751. 10.1111/dme.14751 34837256 PMC9304253

[39] Cane J , O’Connor D , Michie S . Validation of the theoretical framework. Implement Sci 2012;7:1–17. 10.1186/1748-5908-7-37 PMC3483008 22530986

[40] Braun V , Clarke V . Using thematic analysis in psychology. Qualitative Research in Psychology 2006;3:77–101. 10.1191/1478088706qp063oa

[41] INGENIOUS . Ingenious. 2023. Available: https://ingenious.york.ac.uk [Accessed 22 Sep 2023].

[42] Born in Bradford . Born in Bradford. 2023. Available: https://borninbradford.nhs.uk [Accessed 22 Sep 2023].

[43] Kelly FJ , Fussell JC . Air pollution and airway disease. Clin Exp Allergy 2011;41:1059–71. 10.1111/j.1365-2222.2011.03776.x 21623970

[44] Raju S , Siddharthan T , McCormack MC . Indoor air pollution and respiratory health. Clin Chest Med 2020;41:825–43. 10.1016/j.ccm.2020.08.014 33153698 PMC7665158

[45] Parliamentary Office of Science and Technology (POST) . Postbrief 54: indoor air quality. 2023. 10.58248/PB54

[46] Chatzidiakou L , Krause A , Popoola OAM , et al . Characterising low-cost sensors in highly portable platforms to quantify personal exposure in diverse environments. Atmos Meas Tech 2019;12:4643–57. 10.5194/amt-12-1-2019 31534556 PMC6751078

[47] Chatzidiakou L , Krause A , Kellaway M , et al . Automated classification of time-activity-location patterns for improved estimation of personal exposure to air pollution. Environ Health 2022;21:1–21. 10.1186/s12940-022-00939-8 36482402 PMC9733291

