# Supplementary material for: Understanding the patterns and health impact of indoor air pollutant exposures in Bradford, UK: a study protocol
Source: BMJ Open. 2023 Nov 30;13(12):e081099. doi: 10.1136/bmjopen-2023-081099 (PMC10711829; doi:10.1136/bmjopen-2023-081099)
Supplement: Supplementary data [file bmjopen-2023-081099supp001.pdf]

Supplementary File 1. Survey measurements

INGENIOUS

Survey measurements

V2.0 (17/03/2023)

This document includes: Home survey on Day 1, Building audit on Day 1, and Health and behaviour questionnaire on Day 14.

Contents

Home Survey....2
Section I: Home....2
Section II: Behaviour....7
Building Audit....9
Section I: House....9
Section II: Room....12
Health & Behaviour Questionnaire....15
Section I: Your Perception of Your Home and Air Quality....15
Section II: You/Your Household's Behaviour at Home....16
Section III: Health....23
Section IV: About You and Your Household....29

Ikeda E, *et al. BMJ Open* 2023; 13:e081099. doi: 10.1136/bmjopen-2023-081099

Home Survey

Note to Research Assistant:

Please enter participant ID and write today's date BEFORE giving the survey to participant.

*Please enter participant ID (i.e. ING…..): ___________

*Please write today's date: ___/___/_____ (dd/mm/yyyy)

*must provide value

Dear Participant,

We are interested to know about your home. We would be grateful if you help us by answering as many of these questions as possible. All the answers you give are confidential.

Thank you for taking part in the INGENIOUS study!

Section I: Home

This section is about the house you live in at the moment.

1	*How long have you lived at your current home?	Years: _____<br>Months: _____
2	*Does your household own or rent this accommodation?	○ I/we own it or live with the person who owns it (includes homes bought with a mortgage)<br>○ It is rented from the local council

INGENIOUS_Survey measurements_V2.0_17032023

Ikeda E, *et al. BMJ Open* 2023; 13:e081099. doi: 10.1136/bmjopen-2023-081099

		○ It is rented from a housing association or housing trust<br>○ It is rented from a private landlord<br>○ Other (e.g. I live here rent free or home comes with job)
3a	*How far away from this home is the nearest greenspace area (e.g. park, playing field, public garden, woodland or other green space), regardless of size and function?	○ Less than 5 min walk<br>○ 5-10 min walk<br>○ 11-20 min walk<br>○ 21-30 min walk<br>○ More than 30 min walk<br>○ Don't know
3b	*Do you have a garden or a yard in your home?	○ Yes<br>○ No<br>○ Don't know
3c	*Is your home nearby (i.e. within 5-min walking distance):<br>(Tick all that apply)	☐ Busy road<br>☐ Motorway<br>☐ Car park<br>☐ Petrol station<br>☐ Industry (e.g. factory)<br>☐ Waste management site<br>☐ Agricultural land<br>☐ Construction<br>☐ Airport<br>☐ Restaurant or takeaway<br>☐ None of the above<br>☐ Don't know
4	*Do you have an attached garage in your home?	○ Yes<br>○ No<br>○ Don't know
5a	*How many *separate* living or dining rooms does your home have?<br>*Only count rooms that are closed off/separated by a door*	______
5b	*When was the floor of the living or dining room laid (where a sensor will be placed)?	○ In the last 6 months<br>○ 6 months to 1 year ago<br>○ More than 1 year ago<br>○ Don't know
5c	*<u>In the past 12 months</u>, has the interior of the living or dining room (where a sensor will be placed) been refurbished, renovated or painted?	○ Yes<br>○ No<br>○ Don't know
6a	*How many *separate* kitchens does your home have?<br>*Only count rooms that are closed off/separated by a door*	______

6b	*When was the floor of the kitchen laid (where a sensor will be placed)?	○ In the last 6 months<br>○ 6 months to 1 year ago<br>○ More than 1 year ago<br>○ Don't know
6c	*Which floor is the kitchen on (where a sensor will be placed)?	○ Property is on one level<br>○ Basement<br>○ Ground floor<br>○ First floor<br>○ Second floor<br>○ Third floor<br>○ Don't know
6d	*In the past 12 months, has the interior of the kitchen (where a sensor will be placed) been refurbished, renovated or painted?	○ Yes<br>○ No<br>○ Don't know
7a	*How many *separate* bedrooms does your home have?<br>*Only count rooms that are closed off/separated by a door*	______
7b	*When was the floor of the bedroom laid (where a sensor will be placed)?	○ In the last 6 months<br>○ 6 months to 1 year ago<br>○ More than 1 year ago<br>○ Don't know
7c	*In the past 12 months, has the interior of the bedroom (where a sensor will be placed) been refurbished, renovated or painted?	○ Yes<br>○ No<br>○ Don't know
7d	*How many people share the bedroom where a sensor will be placed?	______
8a	*How many *separate* bathrooms does your home have?<br>*Only count rooms that are closed off/separated by a door (e.g. toilet)*	______
8b	*Which floors are main (i.e. regularly used) bathrooms on?<br>(Tick all that apply)	☐ Property is on one level<br>☐ Basement<br>☐ Ground floor<br>☐ First floor<br>☐ Second floor<br>☐ Third floor<br>☐ Don't know
9a	*Are you aware of any signs or smells of mould or damp in your home?	○ Yes<br>○ No (Go to Q10)<br>○ Don't know (Go to Q10)
9b	*In which rooms are there signs or smells of mould or damp?<br>(Tick all that apply)	☐ Living/dining room<br>☐ Kitchen

Ikeda E, et al. BMJ Open 2023; 13:e081099. doi: 10.1136/bmjopen-2023-081099

		☐ Adult's bedroom<br>☐ Child's bedroom<br>☐ Bathroom<br>☐ Basement<br>☐ Other (write in) ____________________
10	*What is the most common type of windows in your whole house?	○ Single glazed windows<br>○ Double or triple glazed windows<br>○ Don't know
11a	*How do you heat your home?<br>(Tick all that apply)	☐ Gas central heating<br>☐ Oil central heating<br>☐ Fixed gas fire<br>☐ Fixed electric heating<br>☐ Night storage heaters<br>☐ Open fires<br>☐ Wood stoves (solid fuel/wood/coal)<br>☐ Pellet boiler<br>☐ Portable heaters: electric, bottled gas/paraffin, oil-filled<br>☐ Other (write in) ____________________<br>☐ Don't know
12a	*What fuel do you use for cooking?<br>(Tick all that apply)	☐ Gas mains<br>☐ Gas bottles<br>☐ Electricity<br>☐ Other (write in) ____________________<br>☐ Don't know

The next question is about ventilation options (please see the photo as examples).

Ikeda E, *et al. BMJ Open* 2023; 13:e081099. doi: 10.1136/bmjopen-2023-081099

Extractor hood:

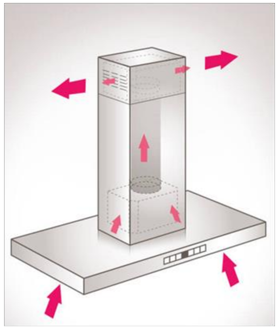

(to inside)

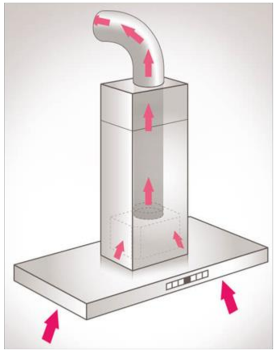

(to outside)

Extractor fan:

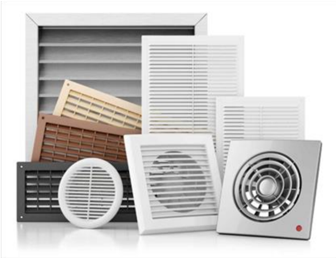

Trickle vents:

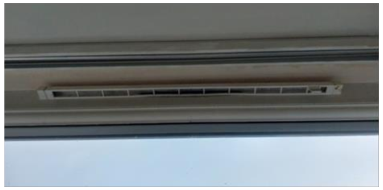

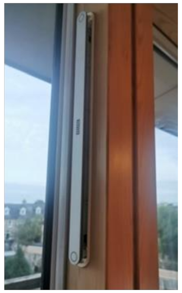

13a	*Does your home have trickle vents?	○ Yes<br>○ No (Go to Q15)<br>○ Don't know (Go to Q15)
13b	*Are the trickle vents open or closed?	○ Trickle vents are always open<br>○ Trickle vents are always closed<br>○ Trickle vents are open or closed depending on the season<br>○ Don't know
14a	*What options are available for ventilating the kitchen? (Tick all that apply)	☐ Extractor hood to inside<br>☐ Extractor hood/fan to outside<br>☐ Window<br>☐ Door to outside<br>☐ Trickle vent<br>☐ There is no means of ventilation<br>☐ Don't know
14b	*What options are available for ventilating the main bathroom/toilet? (Tick all that apply)	☐ Extractor fan<br>☐ Window<br>☐ Trickle vent<br>☐ There is no means of ventilation<br>☐ Don't know
15	*On a normal day, how often are doors to the outside opened and closed?	○ 0-2 times<br>○ 3-10 times<br>○ 11-20 times<br>○ More than 20 times<br>○ Don't know

Ikeda E, *et al. BMJ Open* 2023; 13:e081099. doi: 10.1136/bmjopen-2023-081099

16	*Does your home have a chimney that is *not blocked off*?<br>*Air and smoke can flow out of the chimney*	○ Yes<br>○ No (including using a 'chimney sheep')<br>○ My home doesn't have a chimney<br>○ Don't know
17	*When are people normally in your home?<br>(Tick all that apply)	☐ During the day / school time (9:00-14:59 [24-hour clock])<br>☐ After school (15:00-17:59 [24-hour clock])<br>☐ Evening (18:00-22:59 [24-hour clock])<br>☐ Night (23:00-8:59 [24-hour clock])
18a	*Are there any pets in your household?	○ Yes<br>○ No (Go to Q19a)<br>○ Don't know (Go to Q19a)
18b	*How many pet(s) do you own?	______
19a	*Do you have an air purifier in your home?	○ Yes<br>○ No (Go to Q20a)<br>○ Don't know (Go to Q20a)
19b	What brand or model of air purifier do you have?	____________________

Section II: Behaviour

This section is about smoking behaviour.

1a	*How often do people smoke cigarettes, e-cigarettes, cigar or pipes inside this home?	○ Every day<br>○ Multiple times a week<br>○ Once or twice a week<br>○ Less than once a week<br>○ Never<br>○ Don't know
1b	*How often do people smoke cigarettes, e-cigarettes, cigar or pipes within a close area to this home (e.g. courtyard, with window open, stairwell)?	○ Every day<br>○ Multiple times a week<br>○ Once or twice a week<br>○ Less than once a week<br>○ Never<br>○ Don't know

Thank you for participation.

If you have any questions, please contact us: 01274 364474 or borninbradford@bthft.nhs.uk

INGENIOUS_Survey measurements_V2.0_17032023

Ikeda E, *et al. BMJ Open* 2023; 13:e081099. doi: 10.1136/bmjopen-2023-081099

Building Audit

Please complete the audit below.
Thank you!

INGENIOUS

We are family BORNINBRADFORD

*Please enter participant ID (i.e. ING…..): ___________

*Please write today's date: ___/___/_____ (dd/mm/yyyy)

*must provide value

Section I: House

This section is about the property the participant lives in at the moment.

1	*What type of home is this?	○ Detached house<br>○ Semi-detached house<br>○ Bungalow<br>○ Terraced (including end terrace)<br>○ Flat, maisonette or apartment (in a purpose - built block of flats or tenement)<br>○ Flat, maisonette or apartment (part of converted or shared house, including bedsits)<br>○ Flat, maisonette or apartment (part of another converted building, e.g. former school, church or warehouse)<br>○ Flat, maisonette or apartment (in a commercial building, e.g. in an office building, hotel or over a shop)
2	*When was this home built?	○ Before 1914<br>○ 1914-1944

Ikeda E, *et al. BMJ Open* 2023; 13:e081099. doi: 10.1136/bmjopen-2023-081099

		○ 1945-1964<br>○ 1965-1980<br>○ 1981-1990<br>○ 1991-2002<br>○ 2003 or later<br>○ Don't know
3	*How many floors does the property have?	______
4	*Which floor does the participant live on?<br>(-1 = Basement, 0 = Ground floor, 1 = First floor, 2 = Second floor, etc.)<br><br>*If the participant lives on more than one floors, use comma (,) to list all the floors (e.g. -1, 0, 1).*	__________
5	*What is the energy efficiency rating of the building (which is shown in an energy performance certificate [EPC])?<br><br>*If the participant doesn't know the rating, find an energy certificate using postcode via https://www.gov.uk/find-energy-certificate.*	○ A<br>○ B<br>○ C<br>○ D<br>○ E<br>○ F<br>○ G<br>○ The building has not been certified<br>○ Don't know
6a	*Do any of the windows in the living/dining room (where a sensor will be placed) look out mainly onto grey space, including man-made materials (e.g. roads, buildings)?	○ Yes, at least one window has a main view of grey space<br>○ No, they have a mixed view of grey and green space<br>○ No, they have a main view of green space, including parks and gardens (either public or private) regardless of size and function<br>○ Don't have access to living/dining room
6b	*Do any of the windows in the kitchen (where a sensor will be placed) look out mainly onto grey space, including man-made materials (e.g. roads, buildings)?	○ Yes, at least one window has a main view of grey space<br>○ No, they have a mixed view of grey and green space<br>○ No, they have a main view of green space, including parks and gardens (either public or private) regardless of size and function<br>○ Don't have access to kitchen
7	*How many windows (which can be opened) does the property have?	______
8a	*How many doors lead to the outside?	______

The next question is about draft excluders (please see the photo as examples).

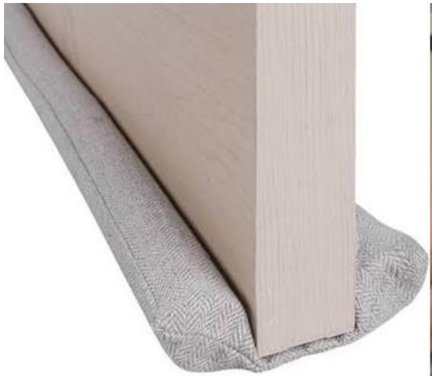
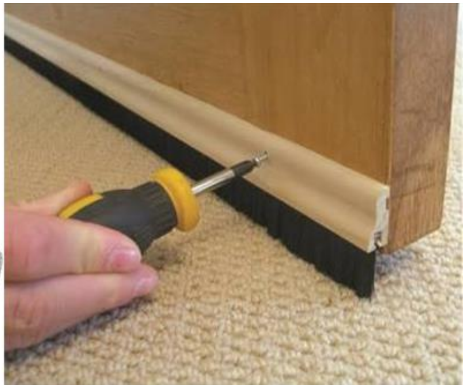

8b	*Are draft excluders attached to outside doors?	○ Yes, all of the doors<br>○ Yes, some of the doors<br>○ No<br>○ Don't have access to outside doors
9a	*Please enter the first sensor's ID:	___________
9b	*Which room is the first sensor installed?	○ Living/dining room<br>○ Kitchen<br>○ Child's bedroom<br>○ Adult's bedroom
9c	*Which floor is the first sensor installed?	○ Basement<br>○ Ground floor<br>○ First floor<br>○ Second floor<br>○ Third floor<br>○ Property is on one level
9d	*Please enter the second sensor's ID:	___________
9e	*Which room is the second sensor installed?	○ Living/dining room<br>○ Kitchen<br>○ Child's bedroom<br>○ Adult's bedroom<br>○ The second sensor is not installed
9f	*Which floor is the second sensor installed?	○ Basement<br>○ Ground floor<br>○ First floor<br>○ Second floor<br>○ Third floor

Ikeda E, *et al. BMJ Open* 2023; 13:e081099. doi: 10.1136/bmjopen-2023-081099

		○ Property is on one level<br>○ The second sensor is not installed
9g	*Please enter the third sensor's ID:	___________
9h	*Which room is the third sensor installed?	○ Living/dining room<br>○ Kitchen<br>○ Child's bedroom<br>○ Adult's bedroom<br>○ The third sensor is not installed
9i	*Which floor is the third sensor installed?	○ Basement<br>○ Ground floor<br>○ First floor<br>○ Second floor<br>○ Third floor<br>○ Property is on one level<br>○ The third sensor is not installed

Section II: Room

This section is about the rooms the property has.

1a	What is the dimension of the living/dining room (where a sensor will be placed)?	Length: _____ metre<br>Width: _____ metre<br>Height: _____ metre
1b	*What material is the floor of the living/dining room (where a sensor will be placed)?<br>(Tick all that apply)	☐ Carpet/rug<br>☐ Engineered wood/solid wood<br>☐ Vinyl/laminate<br>☐ Tile<br>☐ There is no living/dining room
1c	*Which direction does the window(s) of the living/dining room face (where a sensor will be placed)?<br>(Tick all that apply if multiple windows face multiple directions)<br><br>*Please round to the nearest cardinal direction.*	☐ North<br>☐ North East<br>☐ East<br>☐ South East<br>☐ South<br>☐ South West<br>☐ West<br>☐ North West<br>☐ There is no window in the living/dining room
1d	*Does the living/dining room (where a sensor will be placed) have curtains/blinds/shutters?	☐ Yes, all of the windows<br>☐ Yes, some of the windows

		☐ No<br>☐ There is no window in the living/dining room
2a	What is the dimension of the kitchen (where a sensor will be placed)?	Length: _____ metre<br>Width: _____ metre<br>Height: _____ metre
2b	*What material is the floor of the kitchen (where a sensor will be placed)?<br>(Tick all that apply)	☐ Carpet/rug<br>☐ Engineered wood/solid wood<br>☐ Vinyl/laminate<br>☐ Tile<br>☐ There is no kitchen
2c	*Which direction does the window(s) of the kitchen face (where a sensor will be placed)?<br>(Tick all that apply if multiple windows face multiple directions)<br><br>*Please round to the nearest cardinal direction.*	☐ North<br>☐ North East<br>☐ East<br>☐ South East<br>☐ South<br>☐ South West<br>☐ West<br>☐ North West<br>☐ There is no window in the kitchen
2d	*Does the kitchen (where a sensor will be placed) have curtains/blinds/shutters?	☐ Yes, all of the windows<br>☐ Yes, some of the windows<br>☐ No<br>☐ There is no window in the kitchen
2e	*Is the kitchen in open-plan or attached to the living/dining room?	☐ Yes<br>☐ No<br>☐ There is no kitchen
3a	What is the dimension of the bedroom (where a sensor will be placed)?	Length: _____ metre<br>Width: _____ metre<br>Height: _____ metre
3b	*What material is the floor of the bedroom (where a sensor will be placed)?<br>(Tick all that apply)	☐ Carpet/rug<br>☐ Engineered wood/solid wood<br>☐ Vinyl/laminate<br>☐ Tile<br>☐ There is no bedroom
3c	*Which direction does the window(s) of the bedroom face (where a sensor will be placed)?<br>(Tick all that apply if multiple windows face multiple directions)	☐ North<br>☐ North East<br>☐ East<br>☐ South East

Ikeda E, *et al. BMJ Open* 2023; 13:e081099. doi: 10.1136/bmjopen-2023-081099

	Please round to the nearest cardinal direction.	☐ South<br>☐ South West<br>☐ West<br>☐ North West<br>☐ There is no window in the bedroom
3d	*Does the bedroom (where a sensor will be placed) have curtains/blinds/shutters?	☐ Yes, all of the windows<br>☐ Yes, some of the windows<br>☐ No<br>☐ There is no window in the bedroom

Thank you for completing the audit.

Health & Behaviour Questionnaire

Note to Research Assistant:

Please enter participant ID and write today's date BEFORE giving the survey to participant.

*Please enter participant ID (i.e. ING…..): ____________

*Please write today's date: ___/___/_____ (dd/mm/yyyy)

*must provide value

Dear Participant,

We are interested to understand your perception of your home, your/your household's behaviour at home, and your/your child's health and wellbeing. We would be grateful if you could help us by answering as many of these questions as possible. All the answers you give are confidential.

Thank you for taking part in the INGENIOUS study!

INGENIOUS

We are family
BORNINBRADFORD

Section I: Your Perception of Your Home and Air Quality

		Very dissatisfied	Fairly dissatisfied	Neither satisfied nor dissatisfied	Fairly satisfied	Very satisfied
1a	*In general, how satisfied or dissatisfied are you with the general condition of your home?	○	○	○	○	○

INGENIOUS_Survey measurements_V2.0_17032023

Ikeda E, *et al. BMJ Open* 2023; 13:e081099. doi: 10.1136/bmjopen-2023-081099

1b	*Overall, how satisfied or dissatisfied are you with your home as a place to live?	○	○	○	○	○
1c	*Overall, how satisfied or dissatisfied are you with the quality of the air inside your home?	○	○	○	○	○
		Very poor	Poor	Neither good nor poor	Good	Very good
2a	*How do you rate the quality of the air in your home?	○	○	○	○	○
2b	*How do you rate the quality of the air in your street?	○	○	○	○	○

Section II: You/Your Household's Behaviour at Home

1a	*Do you/people in your household usually have breakfast at home on weekdays?	○ Yes<br>○ No (Go to Q1c)<br>○ Don't know (Go to Q1c)
1b	What time do you/people in your household usually have breakfast at home on weekdays?<br>(24-hour clock: 6:00=6:00 a.m., 18:00=6:00 p.m.)	___:___
1c	*Do you/people in your household usually have breakfast at home on the weekend?	○ Yes<br>○ No (Go to Q1e)<br>○ Don't know (Go to Q1e)
1d	What time do you/people in your household usually have breakfast at home on the weekend?<br>(24-hour clock: 6:00=6:00 a.m., 18:00=6:00 p.m.)	___:___
1e	*Do you/people in your household usually have lunch at home on weekdays?	○ Yes<br>○ No (Go to Q1g)<br>○ Don't know (Go to Q1g)
1f	What time do you/people in your household usually have lunch at home on weekdays?<br>(24-hour clock: 1:00=1:00 a.m., 13:00=1:00 p.m.)	___:___
1g	*Do you/people in your household usually have lunch at home on Saturdays?	○ Yes<br>○ No (Go to Q1i)<br>○ Don't know (Go to Q1i)
1h	What time do you/people in your household usually have lunch at home on Saturdays?<br>(24-hour clock: 1:00=1:00 a.m., 13:00=1:00 p.m.)	___:___
1i	*Do you/people in your household usually have lunch at home on Sundays?	○ Yes<br>○ No (Go to Q1k)<br>○ Don't know (Go to Q1k)

Ikeda E, *et al. BMJ Open* 2023; 13:e081099. doi: 10.1136/bmjopen-2023-081099

1j	What time do you/people in your household usually have lunch at home on Sundays?<br>(24-hour clock: 1:00=1:00 a.m., 13:00=1:00 p.m.)	___:___
1k	*Do you/people in your household usually have dinner at home on weekdays?	○ Yes<br>○ No (Go to Q1m)<br>○ Don't know (Go to Q1m)
1l	What time do you/people in your household usually have dinner at home on weekdays?<br>(24-hour clock: 7:00=7:00 a.m., 19:00=7:00 p.m.)	___:___
1m	*Do you/people in your household usually have dinner at home on Saturdays?	○ Yes<br>○ No (Go to Q1o)<br>○ Don't know (Go to Q1o)
1n	What time do you/people in your household usually have dinner at home on Saturdays?<br>(24-hour clock: 7:00=7:00 a.m., 19:00=7:00 p.m.)	___:___
1o	*Do you/people in your household usually have dinner at home on Sundays?	○ Yes<br>○ No (Go to Q2a)<br>○ Don't know (Go to Q2a)
1p	What time do you/people in your household usually have dinner at home on Sundays?<br>(24-hour clock: 7:00=7:00 a.m., 19:00=7:00 p.m.)	___:___

Please select the answer that best describes your household's activities of each over the last 2 weeks:

2a	Has your household used non-stick cookware?	○ Yes, every day<br>○ Yes, multiple times a week<br>○ Yes, once or twice a week<br>○ Yes, less than once a week<br>○ No, we don't use non-stick pans (Go to Q3)<br>○ Don't know (Go to Q3)
2b	*How old is your non-stick cookware?	○ Under 6 months<br>○ Between 6 months and 1 year<br>○ More than 1 year<br>○ Don't know
3a	*Have you/people in your household used hair sprays or deodorant sprays daily?	○ Yes<br>○ No (Go to Q4a)<br>○ Don't know (Go to Q4a)
3b	*How many people in your household have used hair sprays or deodorant sprays daily?	_____

Ikeda E, *et al. BMJ Open* 2023; 13:e081099. doi: 10.1136/bmjopen-2023-081099

4a	*Which home fragrances does your household use or burn? (Tick all that apply)	☐ Candle<br>☐ Oud<br>☐ Uunsi/dabqaad/incense burner<br>☐ Incense sticks<br>☐ Reed diffusers<br>☐ Oil diffusers<br>☐ Air fresheners (including plug in)<br>☐ Other (write in) ____________________<br>☐ No (we don't use or burn home fragrances) (Go to Q5a)<br>☐ Don't know (Go to Q5a)
4c	How many times a week, on average, does your household use or burn any of the following home fragrances?<br><br>*Candle, oud, uunsi/dabqaad/incense burner incense sticks, reed diffusers, oil diffusers, air fresheners (including plug-in), other*	_____

		Every day	Multiple times a week	Once or twice a week	Less than once a week	Never	Don't know
5a	*How often does your household use spray surface/floor/kitchen/bathroom cleaners?	○	○	○	○	○	○
5b	*How often does your household use furniture or other spray polishes?	○	○	○	○	○	○
5c	*How often does your household use liquid surface/floor/kitchen/bathroom cleaners?	○	○	○	○	○	○
5d	*How often does your household use bleach?	○	○	○	○	○	○
6	*How often is your home vacuumed?	○	○	○	○	○	○
7a	*How often do you (or someone else in the household) dust your home?	○	○	○	○	○ (Go to Q8a)	○ (Go to Q8a)

7b	*Which is the main method used for dusting?	○ Wet wipe<br>○ Dry wipe<br>○ Brushing off/using a feather duster<br>○ Using a microfibre duster<br>○ Hoover/vacuum cleaner<br>○ Don't know
8a	*Do you do laundry (including washing and drying clothes) in your home?	○ Yes, washing machine only<br>○ Yes, washing machine and tumble dryer<br>○ Yes, other (write in) ____________________<br>○ No (Go to Q9a)<br>○ Don't know (Go to Q9a)

Ikeda E, *et al. BMJ Open* 2023; 13:e081099. doi: 10.1136/bmjopen-2023-081099

8c	*Which room do you use for washing your clothes in the home?	○ In the kitchen (with a sensor)<br>○ In the utility room<br>○ In the bathroom<br>○ Other (write in) ____________________<br>○ Don't know
8e	*Where/How do you dry your clothes?<br>(Tick all that apply)	☐ In the kitchen (with a sensor)<br>☐ In the living/dining room (with a sensor)<br>☐ In the bedroom (with a sensor)<br>☐ In the bedroom (without a sensor)<br>☐ In the bathroom<br>☐ Outside<br>☐ Using a tumble dryer<br>☐ Other (write in) ____________________<br>☐ Don't know
9a	*Does your household use a wood stove (solid fuel/wood/coal) or an open fire?	○ Yes<br>○ No (Go to Section III: Health, Q1a)
9b	*How many solid fuel appliances do you use?	_____

Please answer the following questions for each appliance separately.

If you use more than 3 solid fuel appliances, please answer for the most-commonly-used 3 appliances (i.e. Appliances 1, 2 and 3).

9c	*What type of solid fuel appliance is *Appliance 1*?	○ Wood or coal burning stoves (solid fuel/wood/coal)<br>○ Open fires (Go to Q9g)<br>○ Biomass boiler (Go to Q9g)<br>○ Other (write in) ____________________ (Go to Q9g)
9e	What make or model (if known) of stove is *Appliance 1*?	____________________
9f	*Is *Appliance 1* *DEFRA*-approved and/or Eco Design Ready?<br>*Department for Environment, Food and Rural Affairs*	○ Yes<br>○ No<br>○ Don't know
9g	*Did you purchase or install *Appliance 1*, or was it there when you moved in?	○ I purchased/installed this appliance<br>○ The appliance was there when I moved in (Go to Q9j)<br>○ Don't know (Go to Q9j)
9h	*What considerations did you have in mind when deciding which wood or coal burning stove to purchase?<br>(Tick all that apply)	☐ Emission rates<br>☐ Environmental impact<br>☐ Best suited for our home and/or needs<br>☐ Price<br>☐ Recommendation<br>☐ The look of the stove<br>☐ Other (write in) ____________________

Ikeda E, *et al. BMJ Open* 2023; 13:e081099. doi: 10.1136/bmjopen-2023-081099

		☐ Don't know
9j	*Did you get any training on using *Appliance 1* when it was installed or when you moved in?	○ Yes<br>○ No, I didn't get given any training or information (Go to Q9m)<br>○ No, I didn't get given any training or information, but I sought information/advice independently (Go to Q9m)<br>○ Don't know (Go to Q9m)
9k	*What training on using *Appliance 1* did you get?<br>(Tick all that apply)	☐ Written instructions<br>☐ Video instructions (e.g. an online guidance video)<br>☐ Someone demonstrated in person<br>☐ Other (write in) ____________________
9m	*When was your chimney or flue for *Appliance 1* last cleaned or maintained?<br>(Tick all that apply)	☐ Professionally cleaned in the last 6 months<br>☐ Professionally cleaned in the last 12 months<br>☐ Cleaned or maintained by someone in the household in the last 6 months<br>☐ Cleaned or maintained by someone in the household in the last 12 months<br>☐ Has not been cleaned or maintained in more than a year<br>☐ Not relevant to this solid fuel appliance<br>☐ Don't know
9n	*What type of fuel do you use in *Appliance 1*?<br>(Tick all that apply)	☐ House coal<br>☐ Smokeless coal<br>☐ Other type of coal<br>☐ Unseasoned wood<br>☐ Seasoned wood<br>☐ Waste wood (e.g. old furniture, pallets or fence panels)<br>☐ Rubbish<br>☐ Manufactured log briquettes<br>☐ Peat<br>☐ Wood pellets<br>☐ Other (write in) ____________________<br>☐ Don't know
9p	*What type of solid fuel appliance is *Appliance 2*?	○ Wood or coal burning stoves (solid fuel/wood/coal)<br>○ Open fires (Go to Q9t)<br>○ Biomass boiler (Go to Q9t)<br>○ Other (write in) ____________________ (Go to Q9t)
9r	What make or model (if known) of stove is *Appliance 2*?	____________________
9s	*Is *Appliance 2* *DEFRA*-approved and/or Eco Design Ready?<br>*Department for Environment, Food and Rural Affairs*	○ Yes<br>○ No<br>○ Don't know

9t	*Did you purchase or install *Appliance 2*, or was it there when you moved in?	○ I purchased/installed this appliance<br>○ The appliance was there when I moved in (Go to Q9w)<br>○ Don't know (Go to Q9w)
9u	*What considerations did you have in mind when deciding which wood or coal burning stove to purchase?<br>(Tick all that apply)	☐ Emission rates<br>☐ Environmental impact<br>☐ Best suited for our home and/or needs<br>☐ Price<br>☐ Recommendation<br>☐ The look of the stove<br>☐ Other (write in) ____________________<br>☐ Don't know
9w	*Did you get any training on using *Appliance 2* when it was installed or when you moved in?	○ Yes<br>○ No, I didn't get given any training or information (Go to Q9z)<br>○ No, I didn't get given any training or information, but I sought information/advice independently (Go to Q9z)<br>○ Don't know (Go to Q9z)
9x	*What training on using *Appliance 2* did you get?<br>(Tick all that apply)	☐ Written instructions<br>☐ Video instructions (e.g. an online guidance video)<br>☐ Someone demonstrated in person<br>☐ Other (write in) ____________________
9z	*When was your chimney or flue for *Appliance 2* last cleaned or maintained?<br>(Tick all that apply)	☐ Professionally cleaned in the last 6 months<br>☐ Professionally cleaned in the last 12 months<br>☐ Cleaned or maintained by someone in the household in the last 6 months<br>☐ Cleaned or maintained by someone in the household in the last 12 months<br>☐ Has not been cleaned or maintained in more than a year<br>☐ Not relevant to this solid fuel appliance<br>☐ Don't know
9aa	*What type of fuel do you use in *Appliance 2*?<br>(Tick all that apply)	☐ House coal<br>☐ Smokeless coal<br>☐ Other type of coal<br>☐ Unseasoned wood<br>☐ Seasoned wood<br>☐ Waste wood (e.g. old furniture, pallets or fence panels)<br>☐ Rubbish<br>☐ Manufactured log briquettes<br>☐ Peat<br>☐ Wood pellets

		☐ Other (write in) ____________________<br>☐ Don't know
9ac	*What type of solid fuel appliance is *Appliance 3*?	○ Wood or coal burning stoves (solid fuel/wood/coal)<br>○ Open fires (Go to Q9ag)<br>○ Biomass boiler (Go to Q9ag)<br>○ Other (write in) ____________________ (Go to Q9ag)
9ae	What make or model (if known) of stove is *Appliance 3*?	____________________
9af	*Is *Appliance 3* *DEFRA*-approved and/or Eco Design Ready?<br>*Department for Environment, Food and Rural Affairs*	○ Yes<br>○ No<br>○ Don't know
9ag	*Did you purchase or install *Appliance 3*, or was it there when you moved in?	○ I purchased/installed this appliance<br>○ The appliance was there when I moved in (Go to Q9aj)<br>○ Don't know (Go to Q9aj)
9ah	*What considerations did you have in mind when deciding which wood or coal burning stove to purchase?<br>(Tick all that apply)	☐ Emission rates<br>☐ Environmental impact<br>☐ Best suited for our home and/or needs<br>☐ Price<br>☐ Recommendation<br>☐ The look of the stove<br>☐ Other (write in) ____________________<br>☐ Don't know
9aj	*Did you get any training on using *Appliance 3* when it was installed or when you moved in?	○ Yes<br>○ No, I didn't get given any training or information (Go to Q9am)<br>○ No, I didn't get given any training or information, but I sought information/advice independently (Go to Q9am)<br>○ Don't know (Go to Q9am)
9ak	*What training on using *Appliance 3* did you get?<br>(Tick all that apply)	☐ Written instructions<br>☐ Video instructions (e.g. an online guidance video)<br>☐ Someone demonstrated in person<br>☐ Other (write in) ____________________
9am	*When was your chimney or flue for *Appliance 3* last cleaned or maintained?<br>(Tick all that apply)	☐ Professionally cleaned in the last 6 months<br>☐ Professionally cleaned in the last 12 months<br>☐ Cleaned or maintained by someone in the household in the last 6 months<br>☐ Cleaned or maintained by someone in the household in the last 12 months<br>☐ Has not been cleaned or maintained in more than a year<br>☐ Not relevant to this solid fuel appliance

Ikeda E, *et al. BMJ Open* 2023; 13:e081099. doi: 10.1136/bmjopen-2023-081099

		☐ Don't know
9an	*What type of fuel do you use in *Appliance 3*? (Tick all that apply)	☐ House coal<br>☐ Smokeless coal<br>☐ Other type of coal<br>☐ Unseasoned wood<br>☐ Seasoned wood<br>☐ Waste wood (e.g. old furniture, pallets or fence panels)<br>☐ Rubbish<br>☐ Manufactured log briquettes<br>☐ Peat<br>☐ Wood pellets<br>☐ Other (write in) ____________________<br>☐ Don't know
9ap	*Are you aware of government regulations regarding burning authorised fuels and exempt appliances?	○ Yes, and I'm familiar with the content of the regulations<br>○ Yes, but I'm not familiar with the content of the regulations<br>○ No, but I know what an exempt appliance is<br>○ No, but I know what authorised fuels are<br>○ No, and I don't know what either authorised fuels or exempt appliances are
9aq	*Do you live in a smoke control area?	○ Yes<br>○ No<br>○ I know what a smoke control area is, but I don't know if I live in one<br>○ I don't know what a smoke control area is
9ar	*Are you aware of government regulations about smoke coming from chimneys that are not in smoke control areas?	○ Yes<br>○ No, but I know what a smoke control area is<br>○ No, and I don't know what a smoke control area is

Section III: Health

This section is about your/your BiB child's health and wellbeing.

Please give your answers on YOUR BiB CHILD's health.

0a	How many BiB children do you have?<br><br>If you have more than one BiB child, but would like to answer ONLY one of your BiB children's health & wellbeing questions, please type '1'.	_____<br><br>If you type more than ONE, please ask a BiB researcher for another set of health & wellbeing questions for your other BiB children.
0b	What is your BiB child's date of birth?	___/___/_____ (dd/mm/yyyy)

1a	*Has your BiB child ever had wheezing or whistling in the chest at any time in the past?	○ Yes<br>○ No (Go to Q1f)<br>○ Don't know (Go to Q1f)
1b	*Has your BiB child had wheezing or whistling in the chest in the past 2 weeks?	○ Yes<br>○ No (Go to Q1f)<br>○ Don't know (Go to Q1f)
1c	*How many attacks of wheezing has your BiB child had in the past 2 weeks?	○ None<br>○ 1<br>○ 2<br>○ 3<br>○ 4<br>○ More than 4<br>○ Don't know
1d	*In the past 2 weeks, how often, on average, has your BiB child's sleep been disturbed due to wheezing?	○ Never woken with wheezing<br>○ Less than one night per week<br>○ One or more nights per week<br>○ Don't know
1e	*In the past 2 weeks, has wheezing ever been severe enough to limit your BiB child's speech to only one or two words at a time between breaths?	○ Yes<br>○ No<br>○ Don't know
1f	*Has your BiB child ever had asthma?	○ Yes<br>○ No (Go to Q1h)<br>○ Don't know (Go to Q1h)
1g	*Has your BiB child ever been diagnosed by a doctor as having asthma?	○ Yes<br>○ No<br>○ Don't know
1h	*In the past 2 weeks, has your BiB child's chest sounded wheezy during or after exercise?	○ Yes<br>○ No<br>○ Don't know
1i	*In the past 2 weeks, has your BiB child had a dry cough at night, apart from a cough associated with a cold or chest infection?	○ Yes<br>○ No<br>○ Don't know
1j	*Has your BiB child taken any medicines for asthma or breathing difficulties (chest tightness, shortness of breath) in the past 2 weeks?	○ Yes<br>○ No<br>○ Don't know
2a	*Has your BiB child ever had a problem with sneezing, or a running, or blocked nose when he/she/they DID NOT have a cold or the flu?	○ Yes<br>○ No (Go to Q2f)<br>○ Don't know (Go to Q2f)

2b	*<u>In the past 2 weeks</u>, has your BiB child had a problem with sneezing, or a running, or blocked nose when he/she/they DID NOT have a cold or the flu?	○ Yes<br>○ No (Go to Q2f)<br>○ Don't know (Go to Q2f)
2c	*<u>In the past 2 weeks</u>, has this nose problem been accompanied by itchy-watery eyes?	○ Yes<br>○ No<br>○ Don't know
2d	*<u>In the past 2 weeks</u>, how many days did this nose problem occur?	______
2e	*<u>In the past 2 weeks</u>, how much did this nose problem interfere with your BiB child's daily activities?	○ Not at all<br>○ A little<br>○ A moderate amount<br>○ A lot<br>○ Don't know
2f	*Has your BiB child <u>ever</u> had hayfever?	○ Yes<br>○ No<br>○ Don't know
3a	*Has your BiB child <u>ever</u> had an itchy rash which was coming and going for at least six months?	○ Yes<br>○ No (Go to Q3g)<br>○ Don't know (Go to Q3g)
3b	*Has your BiB child had this itchy rash at any time <u>in the past 2 weeks</u>?	○ Yes<br>○ No (Go to Q3g)<br>○ Don't know (Go to Q3g)
3c	*Has this itchy rash <u>at any time</u> affected any of the following places?<br><br>*The folds of the elbows, behind the knees, in front of the ankles, under the buttocks, or around the neck, ears or eyes*	○ Yes<br>○ No<br>○ Don't know
3d	*At what age did this itchy rash first occur?	○ Under 2 years<br>○ Age 2-4 years<br>○ Age 5 or more<br>○ Don't know
3e	*Has this rash cleared completely at any time <u>during the past 2 weeks</u>?	○ Yes<br>○ No<br>○ Don't know
3f	*<u>In the past 2 weeks</u>, how often, on average, have your BiB child been kept awake at night by this itchy rash?	○ Never in the past 2 weeks<br>○ Less than one night per week<br>○ One or more nights per week<br>○ Don't know
3g	*Has your BiB child <u>ever</u> had eczema?	○ Yes

○ No
○ Don't know

Please give your answers on the basis of YOUR BiB CHILD's behaviour over the last 2 weeks. It would help us if you answered all items as best you can even if you are not absolutely certain.

		Not true	Somewhat true	Certainly true
4a	*Considerate of other people's feelings	○	○	○
4b	*Restless, overactive, cannot stay still for long	○	○	○
4c	*Often complains of headaches, stomach-aches or sickness	○	○	○
4d	*Shares readily with other children (treats, toys, pencils etc.)	○	○	○
4e	*Often has temper tantrums or hot tempers	○	○	○
4f	*Rather solitary, tends to play alone	○	○	○
4g	*Generally obedient, usually does what adults request	○	○	○
4h	*Many worries, often seems worried	○	○	○
4i	*Helpful if someone is hurt, upset or feeling ill	○	○	○
4j	*Constantly fidgeting or squirming	○	○	○
4k	*Has at least one good friend	○	○	○
4l	*Often fights with other children or bullies them	○	○	○
4m	*Often unhappy, down-hearted or tearful	○	○	○
4n	*Generally liked by other children	○	○	○
4o	*Easily distracted, concentration wanders	○	○	○
4p	*Nervous or clingy in new situations, easily loses confidence	○	○	○
4q	*Kind to younger children	○	○	○
4r	*Often lies or cheats	○	○	○
4s	*Picked on or bullied by other children	○	○	○
4t	*Often volunteers to help others (parents, teachers, other children)	○	○	○
4u	*Thinks things out before acting	○	○	○
4v	*Steals from home, school or elsewhere	○	○	○
4w	*Gets on better with adults than with other children	○	○	○
4x	*Many fears, easily scared	○	○	○
4y	*Sees tasks through to the end, good attention span	○	○	○

4z *Overall, do you think that your BiB child has difficulties in one or more of the following areas:
○ No (Go to Q5a)
○ Yes – minor difficulties

	Emotions, concentration, behaviour or being able to get on with other people?	○ Yes – definite difficulties<br>○ Yes – severe difficulties
4aa	*How long have these difficulties been present?	○ Less than a month<br>○ 1-5 months<br>○ 6-12 months<br>○ Over a year
4ab	*Do the difficulties upset or distress your BiB child?	○ Not at all<br>○ Only a little<br>○ Quite a lot<br>○ A great deal

	Do the difficulties interfere with your BiB child's everyday life in the following areas?	Not at all	Only a little	Quite a lot	A great deal
4ac	*Home life	○	○	○	○
4ad	*Friendships	○	○	○	○
4ae	*Classroom learning	○	○	○	○
4af	*Leisure activities	○	○	○	○

4ag	*Do the difficulties put a burden on you or the family as a whole?	○ Not at all<br>○ Only a little<br>○ Quite a lot<br>○ A great deal

Please refer to YOUR health (and not to the health of your BiB child).

5a	*Do you ever have trouble with your breathing?	○ Never<br>○ Only rarely<br>○ Repeatedly, but it always gets completely better<br>○ Continuously, so that your breathing is never quite right
5b	*Have you had wheezing or whistling in your chest at any time in the past 2 weeks?	○ Yes<br>○ No (Go to Q5i)
5c	*How many attacks of wheezing have you had in the past 2 weeks?	○ None<br>○ 1<br>○ 2<br>○ 3<br>○ 4<br>○ More than 4
5d	*In the past 2 weeks, how often, on average, has your sleep been disturbed due to wheezing?	○ Never woken with wheezing<br>○ Less than one night per week

		○ One or more nights per week
5e	*Have you <u>ever</u> been breathless when the wheezing noise was present?	○ Yes<br>○ No
5f	*<u>In the past 2 weeks</u>, how often, on average, has your sleep been disturbed due to shortness of breath?	○ Never<br>○ Less than one night per week<br>○ One or more nights per week
5g	*<u>In the past 2 weeks</u>, how often, on average, has your sleep been disturbed due to coughing?	○ Never<br>○ Less than one night per week<br>○ One or more nights per week
5h	*<u>In the past 2 weeks</u>, has wheezing ever been severe enough to limit your speech to only one or two words at a time between breaths?	○ Yes<br>○ No
5i	*Have you <u>ever</u> had asthma?	○ Yes<br>○ No (Go to Q5o)
5j	*Was your asthma confirmed <u>by a doctor</u>?	○ Yes<br>○ No
5k	*How old were you when you had your first attack of asthma?<br><br>*If you have never had an asthma attack, please type '88'.*<br>*If you <u>don't know</u> the answer, please type '99'.*	______
5l	*Have you had an asthma attack <u>in the past 2 weeks</u>?	○ Yes<br>○ No
5m	*Have you used any inhaled medicines (e.g. salbutamol) to help your breathing at any time <u>in the past 2 weeks</u> (when you DID NOT have a cold)?	○ Yes<br>○ No
5n	*<u>In the past 2 weeks</u>, how many days was your usual activity (at work or in the home) limited because you had breathing problems?	______
5o	*Have you <u>ever</u> had hay fever?	○ Yes<br>○ No (Go to Q5q)
5p	*Was your hay fever confirmed <u>by a doctor</u>?	○ Yes<br>○ No
5q	*Have you <u>ever</u> had eczema?	○ Yes<br>○ No (Go to Q6a)
5r	*Was your eczema confirmed <u>by a doctor</u>?	○ Yes<br>○ No

Please tick the box that best describe YOUR experience of each <u>over the last 2 weeks</u>:

Ikeda E, *et al. BMJ Open* 2023; 13:e081099. doi: 10.1136/bmjopen-2023-081099

		None of the time	Rarely	Some of the time	Often	All of the time
6a	*I've been feeling optimistic about the future.	○	○	○	○	○
6b	*I've been feeling useful.	○	○	○	○	○
6c	*I've been feeling relaxed.	○	○	○	○	○
6d	*I've been dealing with problems well.	○	○	○	○	○
6e	*I've been thinking clearly.	○	○	○	○	○
6f	*I've been feeling close to other people.	○	○	○	○	○
6g	*I've been able to make up my own mind about things.	○	○	○	○	○

How often have YOU been bothered by any of the following problems over the last 2 weeks?

		Not at all	Several days	More than half the days	Nearly every day
7a	*Little interest or pleasure in doing things	○	○	○	○
7b	*Feeling down, depressed, or hopeless	○	○	○	○
7c	*Trouble falling or staying asleep, or sleeping too much	○	○	○	○
7d	*Feeling tired or having little energy	○	○	○	○
7e	*Poor appetite or overeating	○	○	○	○
7f	*Feeling bad about yourself – or that you are a failure or have let yourself or your family down	○	○	○	○
7g	*Trouble concentrating on things, such as reading the newspaper or watching television	○	○	○	○
7h	*Moving or speaking so slowly that other people could have noticed? Or the opposite – being so fidgety or restless that you have been moving around a lot more than usual	○	○	○	○

Section IV: About You and Your Household

This section is about your socio-economic circumstances.

1a	*How many people including yourself are there in your household?	_____
1b	*How many children (aged under 18) are there in your household?	_____

Please read each statement below and tell us whether the statement was *often true*, *sometimes true*, or *never true* for you or anyone in your household in the last 12 months:

INGENIOUS_Survey measurements_V2.0_17032023

		Often true	Sometimes true	Never true	Don't know	Do not wish to answer
2a	*The food that I/we bought just didn't last, and I/we didn't have money to get more.	○	○	○	○	○
2b	*I/we couldn't afford to eat balanced meals.	○	○	○	○	○

2c	*In the last 12 months, did you or other adults in your household ever cut the size of your meals or skip meals because there wasn't enough money for food?	○ Yes<br>○ No (Go to Q2e)<br>○ Don't know (Go to Q2e)<br>○ Do not wish to answer (Go to Q2e)
2d	*How often did you or other adults cut the size of your meals or skip meals?	○ Almost every month<br>○ Some months but not every month<br>○ Only 1 or 2 months<br>○ Don't know<br>○ Do not wish to answer
2e	*In the last 12 months, did you or other adults ever eat less than you felt you should because there wasn't enough money for food?	○ Yes<br>○ No<br>○ Don't know<br>○ Do not wish to answer
2f	*In the last 12 months, were you ever hungry but didn't eat because there wasn't enough money for food?	○ Yes<br>○ No<br>○ Don't know<br>○ Do not wish to answer
3	*When you are at home on a typical day in winter, are you (and everyone in your household) warm enough?	○ Yes, always<br>○ Yes, sometimes<br>○ No, rarely<br>○ No, never<br>○ Don't know<br>○ Do not wish to answer
4a	*Which of the following is the main way you keep the inside of your house cool in warm weather?<br>(Tick all that apply)	☐ Natural ventilation or shade (e.g. open windows or doors, using heat absorbing shades)<br>☐ Open outside doors<br>☐ Electronic devices (e.g. fans or air conditioning)<br>☐ Other (write in) ____________________<br>☐ Don't know
5	*How do you pay for your electricity/gas? (Tick only one)	○ Pre-payment meter<br>○ Regular bill<br>○ Direct Debit<br>○ Included in rent

		○ Don't know
6a	*How well would you say your household is managing financially these days?<br><br>Would you say you are:	○ Living comfortably<br>○ Doing alright<br>○ Just about getting by<br>○ Finding it quite difficult<br>○ Finding it very difficult<br>○ Do not wish to answer
6b	*Compared to a year ago, how would you say your household is doing financially now?	○ Better off<br>○ Worse off<br>○ About the same<br>○ Do not wish to answer
6c	*How often would you say you have been worried about money during the last few weeks?	○ Almost all the time<br>○ Quite often<br>○ Only sometimes<br>○ Never<br>○ Do not wish to answer
7	Please tell us anything else you would like to comment on in relation to the study.	

Thank you for participation.

If you have any questions, please contact us: xxxxx xxxxxx or borninbradford@bthft.nhs.uk

We are family
BORNINBRADFORD

Ikeda E, et al. BMJ Open 2023; 13:e081099. doi: 10.1136/bmjopen-2023-081099

BMJ Publishing Group Limited (BMJ) disclaims all liability and responsibility arising from any reliance placed on this supplemental material which has been supplied by the author(s)

Supplementary File 2. Diary

Participant ID: ING____________

Health (Week 1)

What is your age? ____ years old **What is your gender?** ☐ Female ☐ Male ☐ Non-binary ☐ Do not wish to answer ☐ Wish to enter own response (____________)

<table>
<tr><th>Please circle one for the following symptoms you had today.
dd/mm/yyyy (ddd) e.g. 09/03/2023 (Thu)</th><th>Day 1
/ /
( )</th><th>Day 2
/ /
( )</th><th>Day 3
/ /
( )</th><th>Day 4
/ /
( )</th><th>Day 5
/ /
( )</th><th>Day 6
/ /
( )</th><th>Day 7
/ /
( )</th></tr>
<tr><td>1. I was at home today.</td><td>YES | NO</td><td>YES | NO</td><td>YES | NO</td><td>YES | NO</td><td>YES | NO</td><td>YES | NO</td><td>YES | NO</td></tr>
<tr><td>If YES, what time? (24 hour clock)</td><td>: – :
: – :
: – :</td><td>: – :
: – :
: – :</td><td>: – :
: – :
: – :</td><td>: – :
: – :
: – :</td><td>: – :
: – :
: – :</td><td>: – :
: – :
: – :</td><td>: – :
: – :
: – :</td></tr>
<tr><td>2. I had breathing difficulties.</td><td>YES | NO</td><td>YES | NO</td><td>YES | NO</td><td>YES | NO</td><td>YES | NO</td><td>YES | NO</td><td>YES | NO</td></tr>
<tr><td>If YES, what time? (24 hour clock)</td><td>: – :</td><td>: – :</td><td>: – :</td><td>: – :</td><td>: – :</td><td>: – :</td><td>: – :</td></tr>
<tr><td>3. I had wheezing in the chest.</td><td>YES | NO</td><td>YES | NO</td><td>YES | NO</td><td>YES | NO</td><td>YES | NO</td><td>YES | NO</td><td>YES | NO</td></tr>
<tr><td>If YES, what time? (24 hour clock)</td><td>: – :</td><td>: – :</td><td>: – :</td><td>: – :</td><td>: – :</td><td>: – :</td><td>: – :</td></tr>
<tr><td>4. I had shortness of breath.</td><td>YES | NO</td><td>YES | NO</td><td>YES | NO</td><td>YES | NO</td><td>YES | NO</td><td>YES | NO</td><td>YES | NO</td></tr>
<tr><td>If YES, what time? (24 hour clock)</td><td>: – :</td><td>: – :</td><td>: – :</td><td>: – :</td><td>: – :</td><td>: – :</td><td>: – :</td></tr>
<tr><td>5. I had chest tightness.</td><td>YES | NO</td><td>YES | NO</td><td>YES | NO</td><td>YES | NO</td><td>YES | NO</td><td>YES | NO</td><td>YES | NO</td></tr>
<tr><td>If YES, what time? (24 hour clock)</td><td>: – :</td><td>: – :</td><td>: – :</td><td>: – :</td><td>: – :</td><td>: – :</td><td>: – :</td></tr>
<tr><td>6. I had chest pain.</td><td>YES | NO</td><td>YES | NO</td><td>YES | NO</td><td>YES | NO</td><td>YES | NO</td><td>YES | NO</td><td>YES | NO</td></tr>
<tr><td>If YES, what time? (24 hour clock)</td><td>: – :</td><td>: – :</td><td>: – :</td><td>: – :</td><td>: – :</td><td>: – :</td><td>: – :</td></tr>
<tr><td>7. I had a dry cough when I did not have a cold or the flu.</td><td>YES | NO</td><td>YES | NO</td><td>YES | NO</td><td>YES | NO</td><td>YES | NO</td><td>YES | NO</td><td>YES | NO</td></tr>
<tr><td>If YES, what time? (24 hour clock)</td><td>: – :</td><td>: – :</td><td>: – :</td><td>: – :</td><td>: – :</td><td>: – :</td><td>: – :</td></tr>
<tr><td>8. I had a problem with sneezing when I did not have a cold or the flu.</td><td>YES | NO</td><td>YES | NO</td><td>YES | NO</td><td>YES | NO</td><td>YES | NO</td><td>YES | NO</td><td>YES | NO</td></tr>
<tr><td>If YES, what time? (24 hour clock)</td><td>: – :</td><td>: – :</td><td>: – :</td><td>: – :</td><td>: – :</td><td>: – :</td><td>: – :</td></tr>
<tr><td>9. I had a runny nose when I did not have a cold or the flu.</td><td>YES | NO</td><td>YES | NO</td><td>YES | NO</td><td>YES | NO</td><td>YES | NO</td><td>YES | NO</td><td>YES | NO</td></tr>
<tr><td>If YES, what time? (24 hour clock)</td><td>: – :</td><td>: – :</td><td>: – :</td><td>: – :</td><td>: – :</td><td>: – :</td><td>: – :</td></tr>
<tr><td>10. I had a blocked nose when I did not have a cold or the flu.</td><td>YES | NO</td><td>YES | NO</td><td>YES | NO</td><td>YES | NO</td><td>YES | NO</td><td>YES | NO</td><td>YES | NO</td></tr>
<tr><td>If YES, what time? (24 hour clock)</td><td>: – :</td><td>: – :</td><td>: – :</td><td>: – :</td><td>: – :</td><td>: – :</td><td>: – :</td></tr>
<tr><td>11. I had itchy-watery eyes when I did not have a cold or the flu.</td><td>YES | NO</td><td>YES | NO</td><td>YES | NO</td><td>YES | NO</td><td>YES | NO</td><td>YES | NO</td><td>YES | NO</td></tr>
<tr><td>If YES, what time? (24 hour clock)</td><td>: – :</td><td>: – :</td><td>: – :</td><td>: – :</td><td>: – :</td><td>: – :</td><td>: – :</td></tr>
<tr><td>12. I had an itchy rash.</td><td>YES | NO</td><td>YES | NO</td><td>YES | NO</td><td>YES | NO</td><td>YES | NO</td><td>YES | NO</td><td>YES | NO</td></tr>
<tr><td>If YES, what time? (24 hour clock)</td><td>: – :</td><td>: – :</td><td>: – :</td><td>: – :</td><td>: – :</td><td>: – :</td><td>: – :</td></tr>
<tr><td>13. I had dry skin.</td><td>YES | NO</td><td>YES | NO</td><td>YES | NO</td><td>YES | NO</td><td>YES | NO</td><td>YES | NO</td><td>YES | NO</td></tr>
<tr><td>If YES, what time? (24 hour clock)</td><td>: – :</td><td>: – :</td><td>: – :</td><td>: – :</td><td>: – :</td><td>: – :</td><td>: – :</td></tr>
<tr><td>At the moment I feel: (0=‘sad’ ☹ – 10=‘happy’ ☺)</td><td>___ (0-10)</td><td>___ (0-10)</td><td>___ (0-10)</td><td>___ (0-10)</td><td>___ (0-10)</td><td>___ (0-10)</td><td>___ (0-10)</td></tr>
</table>

Note:

INGENIOUS_Diary (V2.0; 17/03/2023)

Participant ID: ING____________

Health (Week 2)

<table>
<tr><th>Please circle one for the following symptoms you had today.
dd/mm/yyyy (ddd) e.g. 09/03/2023 (Thu)</th><th>Day 8
/ /
( )</th><th>Day 9
/ /
( )</th><th>Day 10
/ /
( )</th><th>Day 11
/ /
( )</th><th>Day 12
/ /
( )</th><th>Day 13
/ /
( )</th><th>Day 14
/ /
( )</th></tr>
<tr><td>1. I was at home today.
If YES, what time? (24 hour clock)</td><td>YES | NO
: – :
: – :
: – :</td><td>YES | NO
: – :
: – :
: – :</td><td>YES | NO
: – :
: – :
: – :</td><td>YES | NO
: – :
: – :
: – :</td><td>YES | NO
: – :
: – :
: – :</td><td>YES | NO
: – :
: – :
: – :</td><td>YES | NO
: – :
: – :
: – :</td></tr>
<tr><td>2. I had breathing difficulties.
If YES, what time? (24 hour clock)</td><td>YES | NO
: – :</td><td>YES | NO
: – :</td><td>YES | NO
: – :</td><td>YES | NO
: – :</td><td>YES | NO
: – :</td><td>YES | NO
: – :</td><td>YES | NO
: – :</td></tr>
<tr><td>3. I had wheezing in the chest.
If YES, what time? (24 hour clock)</td><td>YES | NO
: – :</td><td>YES | NO
: – :</td><td>YES | NO
: – :</td><td>YES | NO
: – :</td><td>YES | NO
: – :</td><td>YES | NO
: – :</td><td>YES | NO
: – :</td></tr>
<tr><td>4. I had shortness of breath.
If YES, what time? (24 hour clock)</td><td>YES | NO
: – :</td><td>YES | NO
: – :</td><td>YES | NO
: – :</td><td>YES | NO
: – :</td><td>YES | NO
: – :</td><td>YES | NO
: – :</td><td>YES | NO
: – :</td></tr>
<tr><td>5. I had chest tightness.
If YES, what time? (24 hour clock)</td><td>YES | NO
: – :</td><td>YES | NO
: – :</td><td>YES | NO
: – :</td><td>YES | NO
: – :</td><td>YES | NO
: – :</td><td>YES | NO
: – :</td><td>YES | NO
: – :</td></tr>
<tr><td>6. I had chest pain.
If YES, what time? (24 hour clock)</td><td>YES | NO
: – :</td><td>YES | NO
: – :</td><td>YES | NO
: – :</td><td>YES | NO
: – :</td><td>YES | NO
: – :</td><td>YES | NO
: – :</td><td>YES | NO
: – :</td></tr>
<tr><td>7. I had a dry cough when I did not have a cold or the flu.
If YES, what time? (24 hour clock)</td><td>YES | NO
: – :</td><td>YES | NO
: – :</td><td>YES | NO
: – :</td><td>YES | NO
: – :</td><td>YES | NO
: – :</td><td>YES | NO
: – :</td><td>YES | NO
: – :</td></tr>
<tr><td>8. I had a problem with sneezing when I did not have a cold or the flu.
If YES, what time? (24 hour clock)</td><td>YES | NO
: – :</td><td>YES | NO
: – :</td><td>YES | NO
: – :</td><td>YES | NO
: – :</td><td>YES | NO
: – :</td><td>YES | NO
: – :</td><td>YES | NO
: – :</td></tr>
<tr><td>9. I had a runny nose when I did not have a cold or the flu.
If YES, what time? (24 hour clock)</td><td>YES | NO
: – :</td><td>YES | NO
: – :</td><td>YES | NO
: – :</td><td>YES | NO
: – :</td><td>YES | NO
: – :</td><td>YES | NO
: – :</td><td>YES | NO
: – :</td></tr>
<tr><td>10. I had a blocked nose when I did not have a cold or the flu.
If YES, what time? (24 hour clock)</td><td>YES | NO
: – :</td><td>YES | NO
: – :</td><td>YES | NO
: – :</td><td>YES | NO
: – :</td><td>YES | NO
: – :</td><td>YES | NO
: – :</td><td>YES | NO
: – :</td></tr>
<tr><td>11. I had itchy-watery eyes when I did not have a cold or the flu.
If YES, what time? (24 hour clock)</td><td>YES | NO
: – :</td><td>YES | NO
: – :</td><td>YES | NO
: – :</td><td>YES | NO
: – :</td><td>YES | NO
: – :</td><td>YES | NO
: – :</td><td>YES | NO
: – :</td></tr>
<tr><td>12. I had an itchy rash.
If YES, what time? (24 hour clock)</td><td>YES | NO
: – :</td><td>YES | NO
: – :</td><td>YES | NO
: – :</td><td>YES | NO
: – :</td><td>YES | NO
: – :</td><td>YES | NO
: – :</td><td>YES | NO
: – :</td></tr>
<tr><td>13. I had dry skin.
If YES, what time? (24 hour clock)</td><td>YES | NO
: – :</td><td>YES | NO
: – :</td><td>YES | NO
: – :</td><td>YES | NO
: – :</td><td>YES | NO
: – :</td><td>YES | NO
: – :</td><td>YES | NO
: – :</td></tr>
<tr><td>At the moment I feel: (0=‘sad’ ☹ – 10=‘happy’ ☺)</td><td>___ (0-10)</td><td>___ (0-10)</td><td>___ (0-10)</td><td>___ (0-10)</td><td>___ (0-10)</td><td>___ (0-10)</td><td>___ (0-10)</td></tr>
</table>

Note:

INGENIOUS_Diary (V2.0; 17/03/2023)

BMJ Publishing Group Limited (BMJ) disclaims all liability and responsibility arising from any reliance placed on this supplemental material which has been supplied by the author(s)

Weekday

DATE: (please circle one) **Mon** / **Tue** / **Wed** / **Thu** / **Fri** , (please write in) ____/____/______ (dd/mm/yyyy) Participant ID: ING____________ Page 1

PLEASE FILL IN IF APPLICABLE

COOKING

What time did you or someone else in your household do the following activities in your home? (24 hour clock)

Frying	Boiling | Steaming	Baking | Grilling Roasting	Using an extractor hood | fan
: – :	: – :	: – :	: – :
: – :	: – :	: – :	: – :
: – :	: – :	: – :	: – :

CLEANING

What time did you or someone else in your household do the following activities in your home? (24 hour clock)

Vacuuming | Dusting	Using cleaning sprays | furniture polishes
: – :	: – :
: – :	: – :
: – :	: – :

PRODUCTS

What time did you or someone else in your household use the following products in your home? (24 hour clock)

Air freshener sprays Plug-in air fresheners Insecticides | Fly sprays	Candle | Incense | Other fragrance burning sources
: – :	: – :
: – :	: – :
: – :	: – :

HEATING

Were the following heating sources on in your home? (Please circle one each)

Central heating	Central boiler
YES | NO	YES | NO

(YES for timer or temperature control is set)

What time did you or someone else in your household use stoves (solid fuel | wood | coal) or open fires? (24 hour clock)

: – :	: – :
: – :	: – :

VENTILATION

What time did you or someone else in your household open the windows or the doors (leading to outside) in the following rooms (*where a sensor is placed)? (24 hour clock)

Living room*	Kitchen*	Bedroom*	Main bathroom	Use an extractor fan
: – :	: – :	: – :	: – :	: – :
: – :	: – :	: – :	: – :	: – :
: – :	: – :	: – :	: – :	: – :

PERSONAL CARE

What time did you or someone else in your household do the following activities in your home? (24 hour clock)

Taking a shower | bath		Using deodorant | aftershave | perfume | hairspray		Smoking | Using e-cigarettes (in | outside of your house)	
: – :	: – :	: – :	: – :	: – :	: – :
: – :	: – :	: – :	: – :	: – :	: – :

OCCUPANCY

What time were there people at home? How many people?

: – :	___ people	: – :	___ people
: – :	___ people	: – :	___ people
: – :	___ people		

INGENIOUS_Diary (V2.0; 17/03/2023)

INGENIOUS

Weekday

DATE: (please circle one) **Mon** / **Tue** / **Wed** / **Thu** / **Fri** , (please write in) ____/____/______ (dd/mm/yyyy) Participant ID: ING___________

NOTES:

COOKING

E.g. Lots of smoke from a toaster; Left a frying pan on too long and produced lots of smokes

CLEANING

E.g. Used cleaning sprays on Sun morning (if not recorded in the diary); Used eco friendly or natural cleaning products

PRODUCTS

E.g. Used air fresheners in the living room; Burnt candles in the kitchen

HEATING

E.g. Used portable radiant/electric heaters in the living room & child's bedrooms in the night

PERSONAL CARE

E.g. Smelled smoking/e-cigarettes from neighbours; Used deodorant/perfume in the main bathroom

VENTILATION

E.g. Opened patio and bifold doors to get fresh air; Used an air purifier in the living room; Used an dehumidifier in the bathroom and child's bedroom

OTHER EXPERIENCES RELATED TO AIR QUALITY

E.g. Smelled my neighbour's garden fire from inside of the house; Had drain smells in the bathroom; Smelled something (don't know what it is) from the outside

OCCUPANCY

E.g. No one was at home on Sat; My dog and cat stayed inside of the house

BMJ Publishing Group Limited (BMJ) disclaims all liability and responsibility arising from any reliance placed on this supplemental material which has been supplied by the author(s)

Weekend day

DATE: (please circle one) **Sat** / **Sun**, (please write in) ____/____/______ (dd/mm/yyyy) Participant ID: ING____________ Page 3

COOKING

What time did you or someone else in your household do the following activities in your home? (24 hour clock)

Frying	Boiling | Steaming	Baking | Grilling Roasting	Using an extractor hood | fan
: – :	: – :	: – :	: – :
: – :	: – :	: – :	: – :
: – :	: – :	: – :	: – :

CLEANING

What time did you or someone else in your household do the following activities in your home? (24 hour clock)

Vacuuming | Dusting	Using cleaning sprays | furniture polishes
: – :	: – :
: – :	: – :
: – :	: – :

PRODUCTS

What time did you or someone else in your household use the following products in your home? (24 hour clock)

Air freshener sprays Plug-in air fresheners Insecticides | Fly sprays	Candle | Incense | Other fragrance burning sources
: – :	: – :
: – :	: – :
: – :	: – :

HEATING

Were the following heating sources on in your home? (Please circle one each)

Central heating	Central boiler
YES | NO	YES | NO

(YES for timer or temperature control is set)

What time did you or someone else in your household use stoves (solid fuel | wood | coal) or open fires? (24 hour clock)

: – :	: – :
: – :	: – :

VENTILATION

What time did you or someone else in your household open the windows or the doors (leading to outside) in the following rooms (*where a sensor is placed)? (24 hour clock)

Living room*	Kitchen*	Bedroom*	Main bathroom	Use an extractor fan
: – :	: – :	: – :	: – :	: – :
: – :	: – :	: – :	: – :	: – :
: – :	: – :	: – :	: – :	: – :

PERSONAL CARE

What time did you or someone else in your household do the following activities in your home? (24 hour clock)

Taking a shower | bath		Using deodorant | aftershave | perfume | hairspray		Smoking | Using e-cigarettes (in | outside of your house)	
: – :	: – :	: – :	: – :	: – :	: – :
: – :	: – :	: – :	: – :	: – :	: – :

OCCUPANCY

What time were there people at home? How many people?

: – :	____ people	: – :	____ people
: – :	____ people	: – :	____ people
: – :	____ people		

INGENIOUS_Diary (V2.0; 17/03/2023)

INGENIOUS

BMJ Publishing Group Limited (BMJ) disclaims all liability and responsibility arising from any reliance placed on this supplemental material which has been supplied by the author(s)

Weekend day

DATE: (please circle one) **Sat** / **Sun**, (please write in) ____/____/______ (dd/mm/yyyy) Participant ID: ING____________

NOTES:

COOKING

E.g. Lots of smoke from a toaster; Left a frying pan on too long and produced lots of smokes

CLEANING

E.g. Used cleaning sprays on Sun morning (if not recorded in the diary); Used eco friendly or natural cleaning products

PRODUCTS

E.g. Used air fresheners in the living room; Burnt candles in the kitchen

HEATING

E.g. Used portable radiant/electric heaters in the living room & child's bedrooms in the night

PERSONAL CARE

E.g. Smelled smoking/e-cigarettes from neighbours; Used deodorant/perfume in the main bathroom

VENTILATION

E.g. Opened patio and bifold doors to get fresh air; Used an air purifier in the living room; Used an dehumidifier in the bathroom and child's bedroom

OTHER EXPERIENCES RELATED TO AIR QUALITY

E.g. Smelled my neighbour's garden fire from inside of the house; Had drain smells in the bathroom; Smelled something (don't know what it is) from the outside

OCCUPANCY

E.g. No one was at home on Sat; My dog and cat stayed inside of the house

INGENIOUS_Diary (V2.0; 17/03/2023)

INGENIOUS

Diary for Additional Measurements

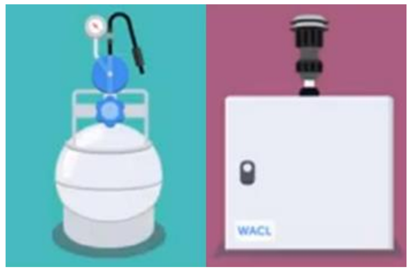

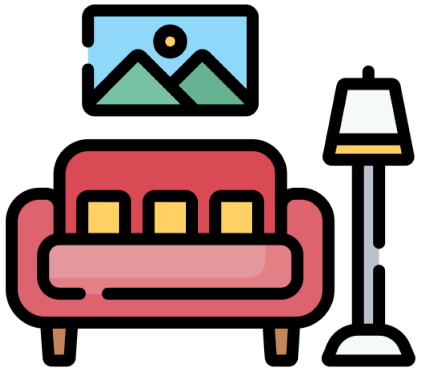

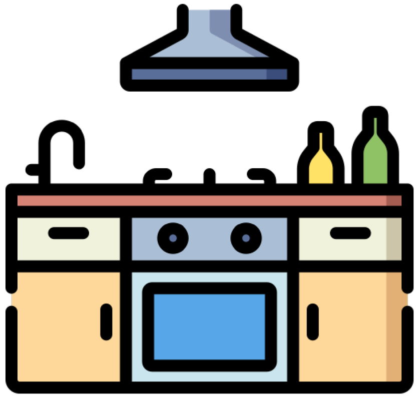

INGENIOUS

We are family
BORNINBRADFORD

Participant ID: ING____________

Additional Measurements

Cooking (PM sampler)

COOKING

Can you please tell us what you cooked for your main meals on each day of sampling?

[Drop-off] Date: ___/___/_____

Mon | Tue | Wed | Thu | Fri | Sat | Sun

Did you cook today?

YES | NO

If YES, what did you cook for your main meals today?

Did you cook meat (e.g. beef, lamb, chicken, pork) and/or seafood (including fish) today?

YES | NO

Date: ___/___/_____

Mon | Tue | Wed | Thu | Fri | Sat | Sun

Did you cook today?

YES | NO

If YES, what did you cook for your main meals today?

Did you cook meat (e.g. beef, lamb, chicken, pork) and/or seafood (including fish) today?

YES | NO

Date: ___/___/_____

Mon | Tue | Wed | Thu | Fri | Sat | Sun

Did you cook today?

YES | NO

If YES, what did you cook for your main meals today?

Did you cook meat (e.g. beef, lamb, chicken, pork) and/or seafood (including fish) today?

YES | NO

Date: ___/___/_____

Mon | Tue | Wed | Thu | Fri | Sat | Sun

Did you cook today?

YES | NO

If YES, what did you cook for your main meals today?

Did you cook meat (e.g. beef, lamb, chicken, pork) and/or seafood (including fish) today?

YES | NO

Date: ___/___/_____

Mon | Tue | Wed | Thu | Fri | Sat | Sun

Did you cook today?

YES | NO

If YES, what did you cook for your main meals today?

Did you cook meat (e.g. beef, lamb, chicken, pork) and/or seafood (including fish) today?

YES | NO

NOTES:

DATE: (please circle one) **Mon** / **Tue** / **Wed** / **Thu** / **Fri** / **Sat** / **Sun**, (please write in) ____/____/______ (dd/mm/yyyy) Participant ID: ING____________

PLEASE FILL IN IF APPLICABLE

COOKING

What time did you or someone else in your household do the following activities in your home? (24 hour clock)

Frying	Boiling | Steaming	Baking | Grilling Roasting	Using an extractor hood | fan
: — :	: — :	: — :	: — :
: — :	: — :	: — :	: — :
: — :	: — :	: — :	: — :

CLEANING

What time did you or someone else in your household do the following activities in your home? (24 hour clock)

Vacuuming | Dusting	Using cleaning sprays | furniture polishes
: — :	: — :
: — :	: — :
: — :	: — :

PRODUCTS

What time did you or someone else in your household use the following products in your home? (24 hour clock)

Air freshener sprays Plug-in air fresheners Insecticides | Fly sprays	Candle | Incense | Other fragrance burning sources
: — :	: — :
: — :	: — :
: — :	: — :

HEATING

Were the following heating sources on in your home? (Please circle one each)

Central heating	Central boiler
YES | NO	YES | NO

(YES for timer or temperature control is set)

What time did you or someone else in your household use stoves (solid fuel | wood | coal) or open fires? (24 hour clock)

: — :	: — :
: — :	: — :

VENTILATION

What time did you or someone else in your household open the windows or the doors (leading to outside) in the following rooms (*where a sensor is placed)? (24 hour clock)

Living room*	Kitchen*	Bedroom*	Main bathroom	Use an extractor fan
: — :	: — :	: — :	: — :	: — :
: — :	: — :	: — :	: — :	: — :
: — :	: — :	: — :	: — :	: — :

PERSONAL CARE

What time did you or someone else in your household do the following activities in your home? (24 hour clock)

Taking a shower | bath		Using deodorant | aftershave | perfume | hairspray		Smoking | Using e-cigarettes (in | outside of your house)	
: — :	: — :	: — :	: — :	: — :	: — :
: — :	: — :	: — :	: — :	: — :	: — :

OCCUPANCY

What time were there people at home? How many people?

: — :	___ people	: — :	___ people
: — :	___ people	: — :	___ people
: — :	___ people		

INGENIOUS_Diary (V3.0; 20/04/2023)

DATE: (please circle one) **Mon** / **Tue** / **Wed** / **Thu** / **Fri** / **Sat** / **Sun**, (please write in) ____/____/______ (dd/mm/yyyy) Participant ID: ING___________

NOTES:

COOKING

E.g. Lots of smoke from a toaster; Left a frying pan on too long and produced lots of smokes

CLEANING

E.g. Used cleaning sprays on Sun morning (if not recorded in the diary); Used eco friendly or natural cleaning products

PRODUCTS

E.g. Used air fresheners in the living room; Burnt candles in the kitchen

HEATING

E.g. Used portable radiant/electric heaters in the living room & child's bedrooms in the night

PERSONAL CARE

E.g. Smelled smoking/e-cigarettes from neighbours; Used deodorant/perfume in the main bathroom

VENTILATION

E.g. Opened patio and bifold doors to get fresh air; Used an air purifier in the living room; Used an dehumidifier in the bathroom and child's bedroom

OTHER EXPERIENCES RELATED TO AIR QUALITY

E.g. Smelled my neighbour's garden fire from inside of the house; Had drain smells in the bathroom; Smelled something (don't know what it is) from the outside

OCCUPANCY

E.g. No one was at home on Sat; My dog and cat stayed inside of the house

INGENIOUS_Diary (V3.0; 20/04/2023)

Supplementary File 3. Example of feedback report

Indoor air pollution feedback report

Dear [participant],

Thank you for taking part in the INGENIOUS study and providing scientists with important insights into air quality in real homes. Your data will help us understand so much about the air in our homes and how we can improve it.

This is a summary of some of the data we collected in your home using the AirGradient sensors we placed in different rooms (the kitchen, bedroom, and living space). The graphs below show the average results over different time periods, to help highlight where there were peaks. Seeing the times these occur may help you identify things you do that cause these peaks.

Your measurements were taken from [date] to [date] . We measured particulate matter (particles in the air), carbon dioxide (mostly from human breath), and total volatile organic compounds (most of them you can smell).

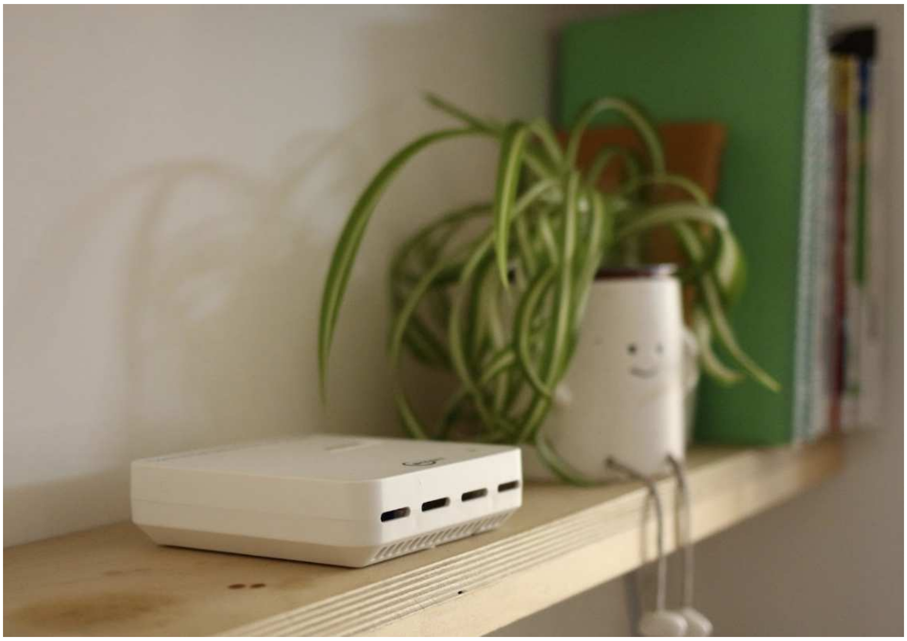

Ikeda E, *et al. BMJ Open* 2023; 13:e081099. doi: 10.1136/bmjopen-2023-081099

Particulate Matter in your kitchen

Particulate matter (PM) is made up of particles (tiny pieces) of solids or liquids that are in the air. PM can come from outside, for example dust from the soil or vehicle exhaust. There are also indoor sources of PM, such as cooking. The graphs below show levels of tiny particles in the air that are 2.5 microns or less in diameter ($PM_{2.5}$). They are also called “fine” particles and are believed to pose the greatest risk to health, particularly for people with heart or lung disease (such as asthma or COPD), babies and children, and the elderly.

On the graphs below, you’ll see time along the bottom (x-axis), and the concentration of chemicals up the left (y-axis). Concentrations are typically measured in units of the mass (micrograms μ) of a chemical per volume of air (cubic meter m^3).

```
Daily (average) levels of particulate matter in the kitchen.
 Blue line shows levels we recorded. Dashed grey line shows the
 maximum daily guideline values which have been recommended
 by the World Health Organization.
```

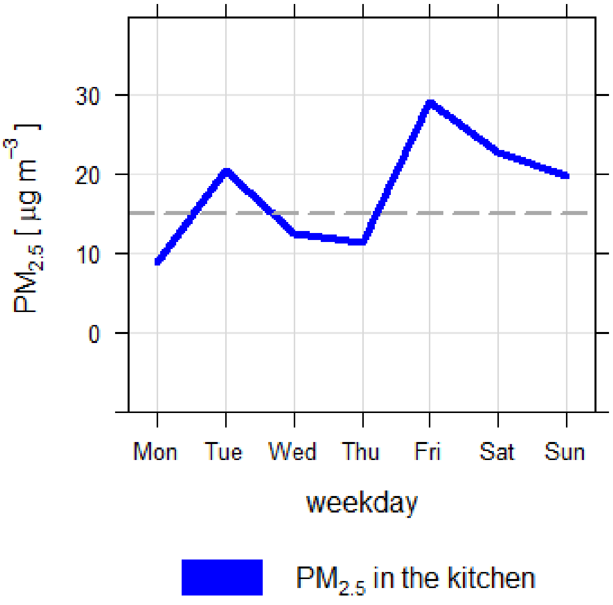

Hourly average levels of particulate matter in the kitchen during the 2 weeks. Time is shown using the 24 hour clock.

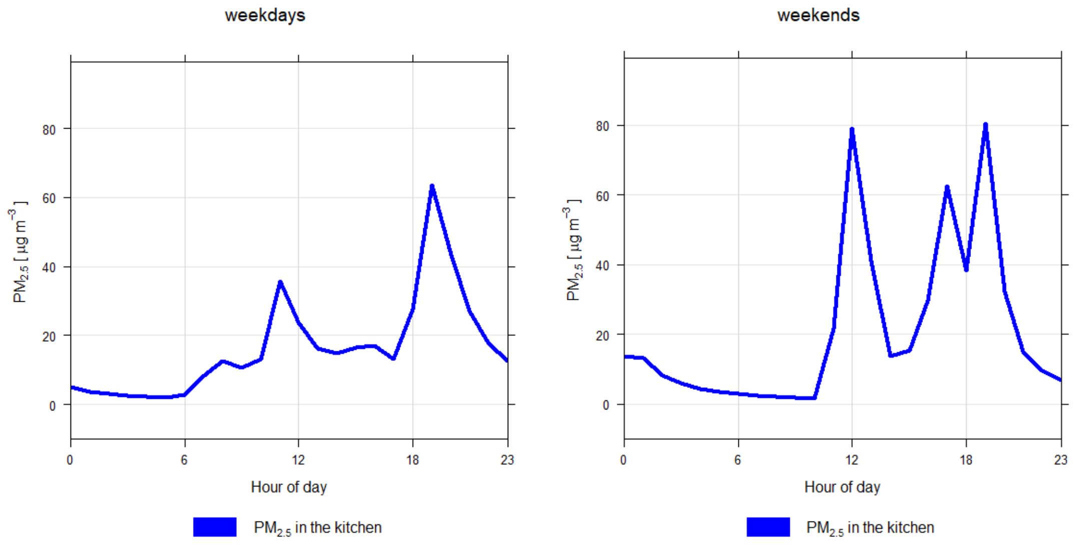

What activities do you think contributed to the peaks seen in these graphs? Think about if there's anything you do differently on certain days, or at different times of day. Activities that could contribute to higher PM levels include cooking, vacuuming or dusting.

If your results are consistently high, you may want to take action to address this, and some suggestions are at the bottom of the document.

Total volatile organic compounds in your living room

Total Volatile Organic Compounds (TVOCs) are a large group of many different chemicals in gas form. They are found in many products we use in our homes. Common indoor sources include personal care products (e.g. deodorants and hair products), paints and cleaning products, smoking and burning wood.

Each chemical has its own toxicity and potential for causing different health effects. Unlike particulate matter, there is no maximum guideline value for TVOCs, and how strong they smell is not a good indicator of

Ikeda E, *et al. BMJ Open* 2023; 13:e081099. doi: 10.1136/bmjopen-2023-081099

health risk. High levels of VOCs can cause eye, nose and, throat irritations, nausea, headaches, dizziness and worsening of asthma symptoms. To protect your health, it is best to limit your exposure to products and materials that contain VOCs.

We measured TVOCs using a low cost sensor, which means that the actual measurements may not be accurate. However, the trends in the measurements are accurate, so the graph below shows you when you have peaks in TVOCs.

```
Daily and hourly average levels of total volatile organic compounds
in the living room during the 2 weeks.The time for each day runs fro
shown by the thicker vertical line.
```

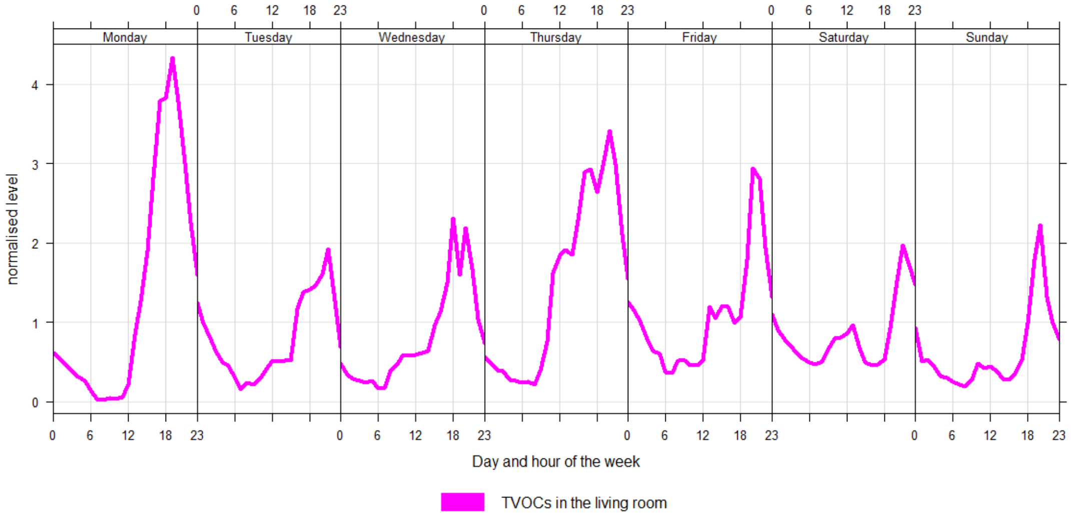

You can see spikes in the TVOCs in the evenings, and sometimes in the morning. What are you doing at that time of day? Is there a link to cooking or cleaning?

Carbon dioxide in your child's bedroom

Carbon dioxide (CO_2) is exhaled in our breath and it is often used as a way to understand how well-ventilated a space is. Studies have shown that high levels may increase the chance of catching virus such as colds, flu, and COVID-19 and also decrease your ability to concentrate.

Ikeda E, *et al. BMJ Open* 2023; 13:e081099. doi: 10.1136/bmjopen-2023-081099

The graph below shows the hourly average levels of CO_2 in your child's bedroom. It is very likely that levels during the night are higher due to the CO_2 breathed out and lower ventilation rates. These levels are not a cause of serious health concerns. Outdoor CO_2 concentrations are shown in the black dashed line and are usually around 400 ppm (might be a bit higher in urban areas). The unit *parts per million* (ppm) refers to molecules. For example, 400 ppm of CO_2 means that for every million molecules of air, there are 400 molecules of CO_2.

```
Hourly average levels of carbon dioxide levels in the
bedroom during the 2 weeks. Time is shown using the 24 hour clock.
```

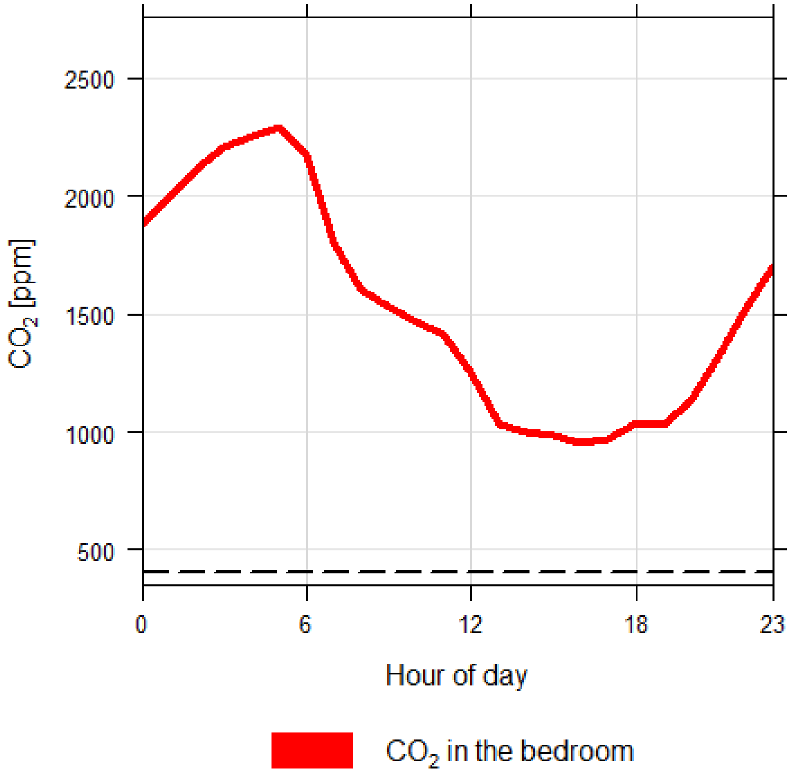

Summary of your indoor air quality

Below we show average levels of particulate matter (in µg/m^3) and CO_2 (in ppm) measured in different rooms over the two week period. The colour

codes give an indication that you may want to consider some actions to improve the indoor air quality in your home.

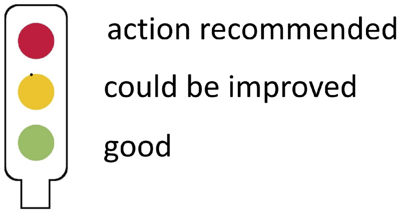

Pollutants	Kitchen	Living_room	Bedroom
$PM_{2.5}$	**17**	**8**	**18**
CO_2	**738**	**749**	**1527**

We have noticed that your maximum PM levels occur at 19 o' clock, most likely during cooking activities. We have also noticed that carbon dioxide concentrations in the bedroom are often quite high exceeding the recommended 1,000 ppm. You may consider ventilating this room more.

Protect yourself and your family

- Even in the cold months, open windows from time to time to allow fresh air to move into the house. This will reduce the levels of many harmful pollutants including PM, TVOCs and CO_2. Aim to open the windows for at least 5 minutes every day, especially if we have found relatively high carbon dioxide levels.
- Use fans/extractors/natural ventilation in the kitchen during cooking to remove fumes, especially if PM levels were above recommended levels. Vacuum the carpets and rugs at least once or twice a week to remove
- dust and allergens, especially in rooms with high PM levels. Consider purchasing low-VOC options for paints and furniture. These information
- are displayed on the label of these products. Where possible, store unused paints, cleaning products or other
- chemicals in a garage, shed or other place where people do not spend much time. Alternatively, store them in a sealed container.

Ikeda E, *et al. BMJ Open* 2023; 13:e081099. doi: 10.1136/bmjopen-2023-081099

We would like to thank you again for your participation. If you have any concerns about these findings, please do not hesitate to contact the BiB Community office at xxxxx xx xxxx

INGENIOUS
Understanding Air Pollution in Homes

[study ID]

Ikeda E, *et al. BMJ Open* 2023; 13:e081099. doi: 10.1136/bmjopen-2023-081099
