# Supplementary material for: Understanding the patterns and health impact of indoor air pollutant exposures in Bradford, UK: a study protocol
Source: BMJ Open. 2023 Nov 30;13(12):e081099. doi: 10.1136/bmjopen-2023-081099 (PMC10711829; doi:10.1136/bmjopen-2023-081099)
Supplement: Author's manuscript [file bmjopen-2023-081099.draft_revisions.pdf]

BMJ Open is committed to open peer review. As part of this commitment we make the peer review history of every article we publish publicly available.

When an article is published we post the peer reviewers' comments and the authors' responses online. We also post the versions of the paper that were used during peer review. These are the versions that the peer review comments apply to.

The versions of the paper that follow are the versions that were submitted during the peer review process. They are not the versions of record or the final published versions. They should not be cited or distributed as the published version of this manuscript.

BMJ Open is an open access journal and the full, final, typeset and author-corrected version of record of the manuscript is available on our site with no access controls, subscription charges or pay-per-view fees (http://bmjopen.bmj.com).

If you have any questions on BMJ Open's open peer review process please email info.bmjopen@bmj.com

BMJ Open

Understanding the patterns and health impact of indoor air pollutant exposures in Bradford, UK: A study protocol

Journal:	*BMJ Open*
Manuscript ID	bmjopen-2023-081099
Article Type:	Protocol
Date Submitted by the Author:	18-Oct-2023
Complete List of Authors:	Ikeda, Erika; Bradford Teaching Hospitals NHS Foundation Trust, Bradford Institute for Health Research, Born in Bradford<br>Hamilton, Jacqueline; University of York, Department of Chemistry, Wolfson Atmospheric Chemistry Laboratories<br>Wood, Chantelle; The University of Sheffield, Department of Psychology<br>Chatzidiakou, Lia; University of Cambridge, Yusuf Hamied Department of Chemistry<br>Warburton, Thomas; University of York, Department of Chemistry, Wolfson Atmospheric Chemistry Laboratories<br>Ruangkanit, Athina; University of York, Department of Chemistry, Wolfson Atmospheric Chemistry Laboratories<br>Shao, Yunqi; The University of Manchester, Department of Earth and Environmental Science, School of Natural Sciences, Centre for Atmospheric Science<br>Genes, Denisa; The University of Sheffield, Department of Psychology<br>Waiblinger, Dagmar; Bradford Teaching Hospitals NHS Foundation Trust, Bradford Institute for Health Research, Born in Bradford<br>Yang, Tiffany; Bradford Teaching Hospitals NHS Foundation Trust, Bradford Institute for Health Research, Born in Bradford<br>Giorio, Chiara; University of Cambridge, Yusuf Hamied Department of Chemistry<br>McFiggans, Gordon; The University of Manchester, Department of Earth and Environmental Science, School of Natural Sciences, Centre for Atmospheric Science; University of York, National Centre for Atmospheric Science<br>O'Meara, Simon; The University of Manchester, Department of Earth and Environmental Science, School of Natural Sciences, Centre for Atmospheric Science; University of York, National Centre for Atmospheric Science<br>Edwards, Pete; University of York, Department of Chemistry, Wolfson Atmospheric Chemistry Laboratories; University of York, National Centre for Atmospheric Science<br>Bates, Elizabeth; City of Bradford Metropolitan District Council, Department of Place<br>Shaw, David; University of York, Department of Environment and Geography<br>Jones, Roderic; University of Cambridge, Yusuf Hamied Department of Chemistry<br>Carslaw, Nicola; University of York, Department of Environment and Geography<br>McEachan, Rosemary; Bradford Teaching Hospitals NHS Foundation

	Trust, Bradford Institute for Health Research, Born in Bradford
Keywords:	EPIDEMIOLOGIC STUDIES, Health Equity, Observational Study, PUBLIC HEALTH, QUALITATIVE RESEARCH, Surveys and Questionnaires

SCHOLARONE™
Manuscripts

Understanding the patterns and health impact of indoor air pollutant exposures in Bradford, UK: A study protocol

Erika Ikeda[1]

Jacqueline Hamilton[2]

Chantelle Wood[3]

Lia Chatzidiakou[4]

Thomas Warburton[2]

Athina Ruangkanit[2]

Yunqi Shao[5]

Denisa Genes[3]

Dagmar Waiblinger[1]

Tiffany C Yang[1]

Chiara Giorio[4]

Gordon McFiggans[5,6]

Simon P O'Meara[5,6]

Pete Edwards[2,6]

Elizabeth Bates[7]

David R Shaw[8]

Roderic L Jones[4]

Nicola Carslaw[8]

Rosemary R C McEachan[1]*

*Corresponding author

Erika Ikeda

Erika.Ikeda@bthft.nhs.uk

Born in Bradford, Bradford Institute for Health Research, Bradford Teaching Hospitals NHS Foundation Trust, Bradford Royal Infirmary, Duckworth Lane, Bradford, BD9 6RJ, UK

For peer review only

[1]Born in Bradford, Bradford Institute for Health Research, Bradford Teaching Hospitals NHS Foundation Trust, Bradford Royal Infirmary, Bradford, BD9 6RJ, UK

[2]Wolfson Atmospheric Chemistry Laboratories, Department of Chemistry, University of York, York, YO10 5DQ, UK

[3]Department of Psychology, University of Sheffield, Sheffield, S1 4DP, UK

[4]Yusuf Hamied Department of Chemistry, University of Cambridge, Cambridge, CB2 1EW, UK

[5]Department of Earth and Environmental Science, Centre for Atmospheric Science, School of Natural Sciences, University of Manchester, Manchester, M13 9PL, UK

[6]National Centre for Atmospheric Science, University of York, York, YO10 5DQ, UK

7Department of Place, City of Bradford Metropolitan District Council, Bradford, BD1 1HX, UK

[8]Department of Environment and Geography, University of York, York, YO10 5NG, UK

For peer review only

For peer review only - http://bmjopen.bmj.com/site/about/guidelines.xhtml

Abstract

Introduction: Relative to outdoor air pollution, there is little evidence examining the composition and concentrations of indoor air pollution and its associated health impacts. The INGENIOUS project aims to provide the comprehensive understanding of indoor air pollution in United Kingdom (UK) homes.

Methods and analysis: 'Real Home Assessment' is a cross-sectional, multi-method study within INGENIOUS. This study monitors indoor air pollutants over two weeks using low-cost sensors placed in three rooms in 300 Born in Bradford (BiB) households. Building audits are completed by researchers, and participants are asked to complete a home survey and a health and behaviour questionnaire, in addition to recording household activities and health symptoms on at least one weekday and one weekend day. A subsample of 150 households receives more intensive measurements of volatile organic compound and particulate matter for three days. Qualitative interviews conducted with 30 participants will identify key barriers and enablers of effective ventilation practices. Outdoor air pollution is measured in 14 locations across Bradford to explore relationships between indoor and outdoor air quality. Data will be analysed to explore total concentrations of indoor air pollutants, how these vary with building characteristics, and whether they are related to health symptoms. Interviews will be analysed through content and thematic analysis.

Ethics and dissemination: Ethical approval has been obtained from the NHS Health Research Authority (22/YH/0288). We will disseminate findings using our websites, social media, publications and conferences. Data will be open access through the BiB, the Open Science Framework and the UK Data Service.

Strengths and limitations of this study

- The Real Home Assessment is one of the first research studies that is designed to understand how indoor air pollution in 300 UK homes is generated, prevented, reduced, and associated with social, ethnic and health inequalities.
- Participants are recruited from the longitudinal, multi-ethnic Born in Bradford cohort allowing linkage of detailed assessments of indoor air quality with rich retrospective and prospective information on health and life circumstances.
- Commercially available, low-cost air pollutant sensors are used to provide summaries of the most common pollutants (carbon dioxide, volatile organic compound [VOC], participate matter [PM]), temperature and relative humidity in homes. Detailed assessments of VOC and PM using state of the art instrumentation will be conducted in a subsample.
- Collection of measurements is time-consuming and can cause burden for participants. To minimise burden, the measurements are collected over a two-week period, and thus we are unable to capture longer term exposures to indoor air pollution.

For peer review only

- Exploratory analyses will be performed to understand the patterns of exposures to indoor air pollution and their contributing factors (occupant and building related), and to explore relationships between symptoms (respiratory and mental health) and exposures.

Keywords

Born in Bradford

Indoor air quality

Air pollution

Volatile organic compound

Particulate matter

Carbon dioxide

Asthma

Behaviour change

Ventilation

For peer review only

1. Introduction

Air pollution is thought to be responsible for between 28,000 and 36,000 deaths each year in the United Kingdom (UK) alone [1]. Long-term exposure to outdoor air pollution has been associated with higher rates of cardiovascular and respiratory illness, birth-defects and neuro-degenerative disorders [2]. Evidence derived from large-scale epidemiological studies indicate a causal relationship between exposure to outdoor air pollution and increased rates of mortality and morbidity [3]. Most measurements, modelling and regulations of air pollution have so far focused on the outdoor environment [4]. Equivalent research for indoor environments lags significantly behind, despite estimations that in developed countries such as the UK, we spend on average around 90% of our time indoors, with approximately two thirds of this in our homes [5]. This may be partly due to limitations in the availability, maintenance and cost of indoor air quality monitors, with nearly all studies of health effects having used data from fixed outdoor air pollution monitoring networks [4]. This lack of data on indoor air pollution may result in inadequate exposure metrics and large uncertainties surrounding its sources and health impacts.

In the UK and globally, there is a dearth of evidence on the sources and transformations of indoor air pollutants [6, 7]. The limited evidence available shows that indoor air is a multidimensional issue influenced by the interrelationship between indoor and outdoor air sources and transformation processes; building design, management and use; and human behaviour (e.g. cooking, cleaning, ventilation) [6]. Since many of these dependencies are specific to location and social factors (e.g. weather conditions, building regulations, cultural and social practices), comprehensive measurements with coordinated UK-specific modelling are needed for understanding indoor air quality relevant to UK homes. Without a fundamental understanding of how air pollution is caused, transformed and distributed in UK homes, research aiming to develop behavioural, technical or policy interventions to reduce future air pollution exposures may have little impact, or even be counterproductive.

The INGENIOUS (understandING the sourcEs, traNsformations and fate of IndOor air pollUtantS) project consists of seven linked work packages to understand the causes and concentration of, and health burden caused by, indoor air pollution (https://ingenious.york.ac.uk/). The current protocol describes research conducted with Born in Bradford (https://borninbradford.nhs.uk/). For ease, we refer to this part of the project as the "Real Home Assessment" study.

2. Methods and Analysis

2.1. Research questions

Real Home Assessment is a cross-sectional, multi-method study which has four research questions:

1) What are the patterns of indoor air pollution in typical UK homes and are there inequalities in exposure to indoor air pollution;
2) How do physical characteristics of buildings or occupant behaviours contribute to indoor air pollution;

For peer review only - http://bmjopen.bmj.com/site/about/guidelines.xhtml

3) How are indoor air pollution and housing quality related to levels of respiratory symptoms and mental health; and
4) What are the barriers and enablers of ventilation behaviours in the home?

Research questions 1-3 will be answered using quantitative data collected from air quality monitors and questionnaires from 300 homes over a 2-week period. Research question 4 will be answered using data from qualitative semi-structured interviews.

2.2. Study design and setting

This study is conducted in Bradford which is an urban, multicultural city located in the North of England, UK. Bradford is the 7th largest metropolitan district in the UK with a population of >546,000 [8]. Approximately 67% of the population identify as White British and 20% as of Pakistani origin [8]. About 34% of Bradford residents live in the most deprived neighbourhoods in England [9]. The Born in Bradford (BiB) is a prospective pregnancy and birth cohort which follows the health and wellbeing of over 12,500 families with children born in the city of Bradford, UK between 2007 and 2011 [10]. Half of the cohort are of South Asian origin, reflective of the demographic of young adults in this population (e.g. more likely to be of child-bearing age) within the city [10].

2.3. Participant characteristics and recruitment

A total of 300 families are recruited from the BiB cohort who took part in the recent follow-up (2017-2020), BiB Growing Up study (in which BiB children were aged 7-11 years) [11]. Potential families were identified by stratifying the cohort by child ethnicity (i.e. South Asian, White British, other), housing tenure (i.e. private/mortgaged, rented) and presence of childhood asthma (i.e. asthma: children had asthma symptoms up to two years before the Growing Up data collection; non-asthma) to maximise the variability and relevance of the sample at the local and national levels (Table 1). To be eligible to participate in the Real Home Assessment study, a parent needs to be able to give informed consent; a household suitable to install indoor air quality monitors; and a family able to complete questionnaires and diaries. A family is excluded from the study if a parent is unable to give informed consent and/or communicate in English.

Recruitment of the 300 families began in March 2023 with final visits due to occur in April 2024. Subsamples of 150 and 30 (out of 300) families are further invited for additional assessments of indoor air pollution and qualitative interviews, respectively (see Section 2.4).

Table 1. The recruitment target of 300 BiB families stratified by child ethnicity, housing tenure and childhood asthma

		**Housing tenure**			
		Private/mortgaged property (N=210; 70%)		Rented property (N=90, 30%)	
		Asthma (50%)	Non-asthma (50%)	Asthma (50%)	Non-asthma (50%)
**Ethnicity**	South Asian	N=48; 16.0%	N=47; 15.7%	N=20; 6.7%	N=20; 6.7%

For peer review only - http://bmjopen.bmj.com/site/about/guidelines.xhtml

(N=135; 45%)				
White British (N=135; 45%)	N=48; 16.0%	N=47; 15.7%	N=20; 6.7%	N=20; 6.7%
Other (N=30; 10%)	N=10; 3.3%	N=11; 3.7%	N=5; 1.7%	N=4; 1.3%

2.4. Procedure

Figure 1 outlines the procedure of recruitment and data collection which involves placing air quality monitors (including AirGradient sensors, volatile organic compound [VOC] canister and particulate matter [PM] sampler) in participants' homes and conducting survey measurements and qualitative interviews over a 2-week period. We deploy a tiered approach to indoor air quality assessment, and the sample size is selected to maximise breadth of data and be deliverable within the funded project timelines (Figure 2).

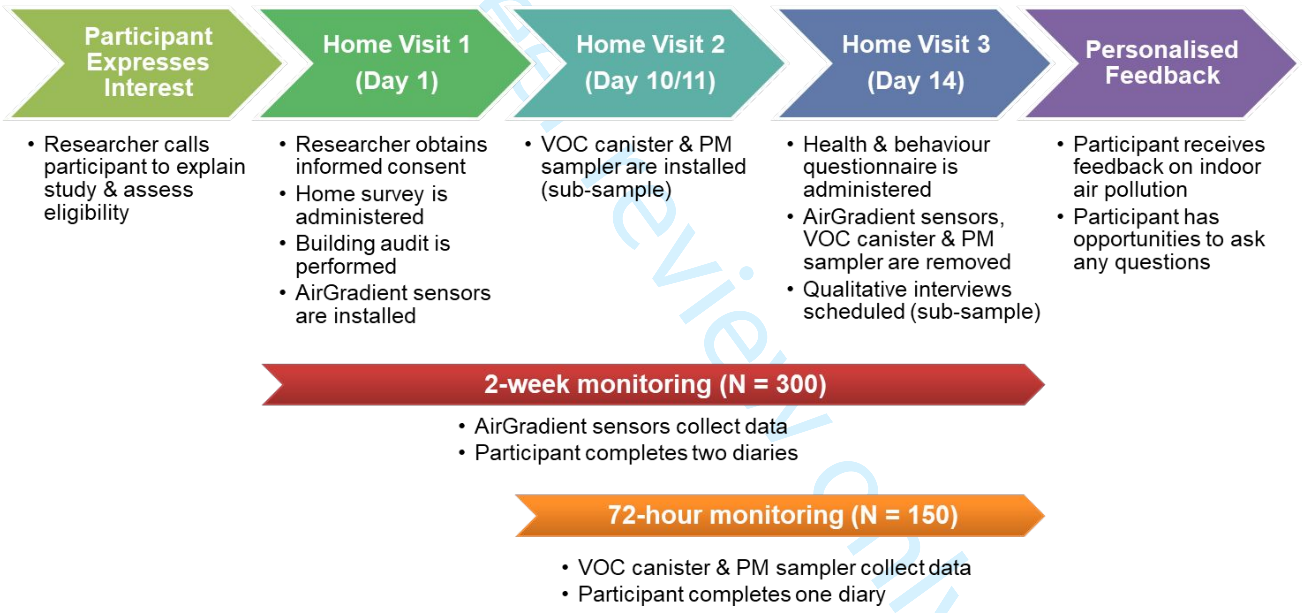

Figure 1. The procedure of recruitment and data collection. VOC = volatile organic compound; PM = particulate matter.

Eligible participants who have previously given consent to be contacted by BiB receive a phone call from the research team for recruitment. Participants are provided with the aim of the study and an overview of the study timeline and data collection. If the family is willing to take part in the study, a home visit is scheduled at a convenient time. Participant information sheets are provided electronically via email to participants before, or in person during the first home visit where written informed consent is obtained and the participants have the opportunity to ask questions.

For peer review only - http://bmjopen.bmj.com/site/about/guidelines.xhtml

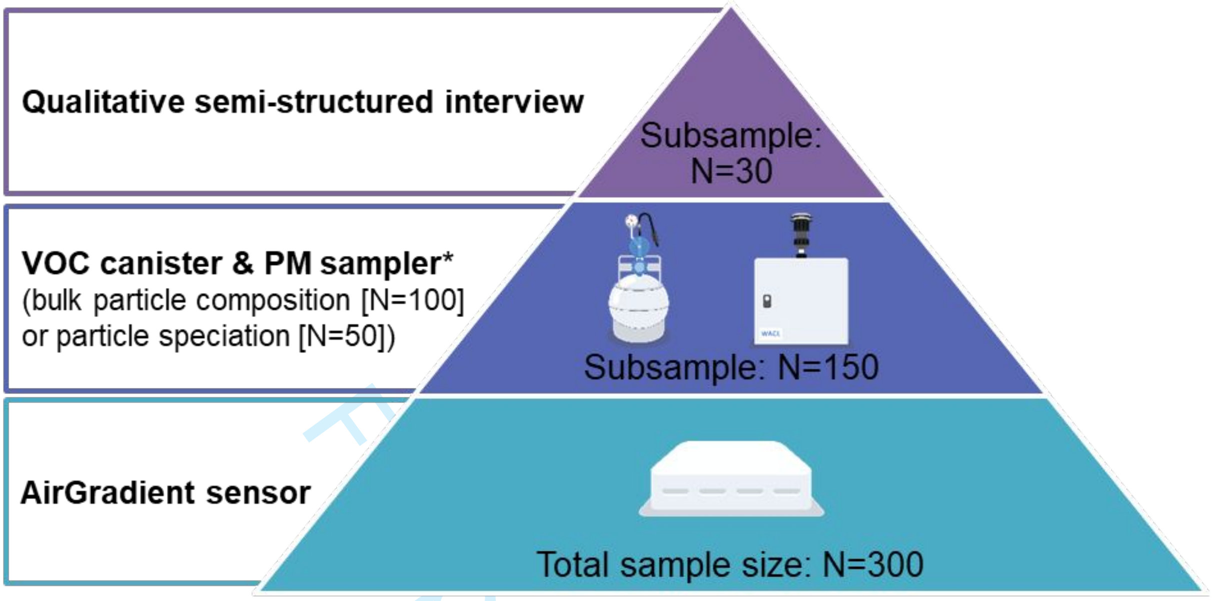

Figure 2. Tiered indoor air quality assessment. VOC = volatile organic compound; PM = particulate matter. *150 filters collected from PM samplers are analysed for either bulk particle chemical composition or detailed particle speciation.

Data collection occurs over 14 days with two home visits. During the first home visit (Day 1), a member of the research team places a portable Wi-Fi hub at the participant's property, and installs three low-cost (i.e. AirGradient) sensors, which are connected to the Wi-Fi hub in three rooms (where participants are more likely to spend time and pollutants are likely to be generated): a living room, a kitchen and a child's bedroom (or the room where a BiB child sleeps) [12]. The researcher then conducts a building audit to identify key characteristics of the property, and the participant completes a home survey which assesses the condition of their house. Instructions are given by members of the research team on how to complete the provided activity and symptom diaries over the next two weeks. On the same visit, a subsample of participants are also invited to take part in a 3-day period of more intensive monitoring of VOCs in a living room and PM in a kitchen which would take place at the end of the 2-week period (Figure 2). Among the families agreeing to take part in additional VOC and PM monitoring, an appointment is agreed between the research team and participant for an additional home visit (preferably on Day 10 or 11) to install a VOC canister and a PM sampler and provide another set of diaries. In the PM samplers, the filters are used for either bulk particle chemical composition (N=100) or detailed particle speciation (N=50) analyses (Figure 2).

On Day 14, the researcher returns to collect the air quality monitors and diaries. The participant completes a questionnaire on the health and behaviours of the participant and their child(ren) over the past two weeks. The subsample of families who complete the additional VOC and PM monitoring are further invited for a qualitative interview exploring barriers and enablers to ventilation behaviours in the home. A total of 30 semi-structured interviews are conducted online or in-person after obtaining informed consent.

For peer review only

Following the completion of the data monitoring period, participants are provided with a personalised feedback report outlining the levels of air pollution in their home measured based on the sensors' readings, and are given the opportunity to ask questions about the study. All participants receive a £50 voucher in recognition of their time and effort, and those participating in the interview receive an additional £20 voucher.

2.5. Indoor air quality measurements

2.5.1. AirGradient sensor

All homes receive commercially available low-cost sensors, AirGradient [13] that measures (1-min resolution) and transmits (5-min average intervals) air pollution data to secure server through a Wi-Fi hotspot provided by the research team. The sensor was selected based on its performance in validation tests, ease of use, size, robustness, price and low noise operation to minimise disturbance of occupants (Figure 3). The AirGradient contains a series of sensors: a SenseAir S8 carbon dioxide (CO_2) sensor using non-dispersive infrared technology to measure CO_2 concentration (parts per million by volume [ppm]); a Plantower PMS5003 sensor with laser scattering technology to measure three size fractions of PM concentration (PM_1, $PM_{2.5}$, PM_{10} in micrograms per cubic metre [μg/m^3]); a Sensirion SGP41 total volatile organic compound (TVOC) / nitrogen oxides (NO_X) sensor to measure TVOC concentration (parts per billion by volume [ppb]); and a Sensirion SHT3x/4x sensor to measure temperature and relative humidity. Where possible, the sensors are placed on a table or shelf and away from external walls and windows [14] in a living room, kitchen and BiB child bedroom while minimising interference with normal occupant behaviours.

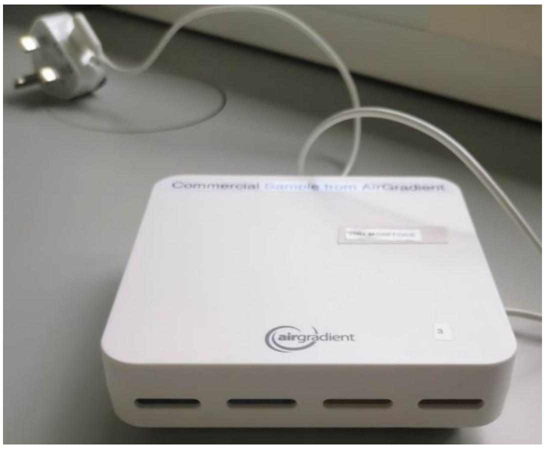

Figure 3. Image of the AirGradient sensor

2.5.2. VOC canister

In a subsample of 150 homes, a VOC canister collects air over a 3-day period. Six-litre vacuum-intake stainless-steel canisters from either Entech (CA, USA) or Restek (PA, USA) are used, and a flow-restrictive inlet is attached (Figure 4). Prior to deployment, each canister is evacuated with a pressure below 0.01 Pa

For peer review only - http://bmjopen.bmj.com/site/about/guidelines.xhtml

to ensure there is no residual ambient air in the canister. During the deployment, the research team attaches a flow-restrictive inlet onto a canister and places the canister on the floor within the living space and away from external walls and windows [14] while minimising interference with normal occupant behaviours. The canister air sample collected from each home is processed off-line in a laboratory, using a custom thermal desorption unit linked to a two-column Agilent 7890A gas chromatograph utilising flame ionisation detection and an Agilent 5977A quadrupole mass spectrometer (GC-FID-QMS) [15]. A broad range of compounds of different molecular sizes and chemical functionality are targeted including ethane from natural gas, butane from aerosol spray products, monoterpenes from fragrances, and ethanol from solvent use.

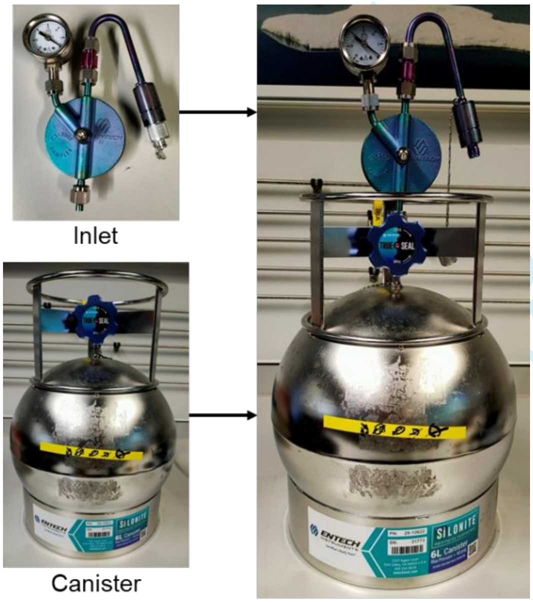

Figure 4. Images of the Entech 6-litre canister and an inlet

For peer review only

2.5.3. PM sampler

The same participant subsample receiving a VOC canister (N=150) also host samplers to collect PM onto filter papers for analysis using advanced analytical instrumentation. A Minivol Tactical Air Sampler with a $PM_{2.5}$ impactor protruding from the top is used to collect PM less than 2.5 microns in diameter onto pre-based 47 mm filter papers (Figure 5). The sampler draws air through the polytetrafluoroethylene filter using a pump set at 3 L·min^{-1} over the course of approximately 72 hours. The exact sample time is recorded by a trained researcher who starts and stops the sampling process. To minimise the sound from the pump, the sampler is enclosed by a custom-built metal box lined with sound proofing foams (Dodo Sound Stopper Pro v2) (see Figure 5). The soundproofing enclosure reduced the sound emitted by 10 dBA to a final sound level of 50 dBA. Where possible, the sampler is placed on a kitchen countertop or table and away from windows [14]. Out of 150 filter samples, two-thirds (N=100) are analysed to characterise the bulk chemical composition of particles. A 25mm punch is excised from the 47 mm filter to subsequently analyse organic aerosol composition using a time-of-flight chemical ionisation mass spectrometer with iodide ionisation

For peer review only - http://bmjopen.bmj.com/site/about/guidelines.xhtml

coupled with a filter inlet for gases and aerosols (FIGAERO-CIMS) [16, 17]. An appropriate fraction of residual filter material will be extracted using methanol and Aerodyne high-resolution time-of-flight aerosol mass spectrometer (HR-ToF-AMS) [18] to analyse both bulk inorganic and organic chemical composition. One third (N=50) of the filter samples collected are analysed to obtain the concentrations of specific chemical compounds that are tracers of specific PM sources and/or are known to have toxic effects in humans. The filters are extracted using accelerated solvent extraction and analysed using two-dimensional gas chromatography coupled with time-of-flight mass spectrometry (GC×GC-ToF-MS) [19].

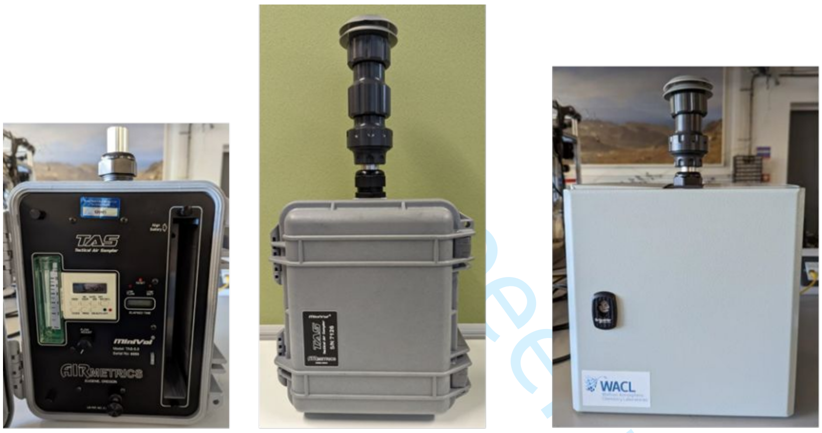

Sampler (inside) Sampler (outside) Sampler in a soundproof box

Figure 5. Images of the Minivol Tactical Air Sampler and a soundproof box

2.6. Outdoor air quality monitoring

In collaboration with the City of Bradford Metropolitan District Council, outdoor air pollutants (PM_{10}, $PM_{2.5}$, NO_X, ozone [O_3], CO_2) are being measured using Earthsense Zephyr [20] devices in 10 locations across the Bradford city since June 2023. In addition, four AQMesh [21] units which measure the same target pollutants as the Zephyr devices have been deployed in the residential areas of Bradford since July 2023, following a 2-month collection period with reference devices at the Manchester Air Quality supersite. The data from both devices will be combined with those from local reference monitors to estimate outdoor air pollution in the vicinity of participants' homes, and thus examine the influence of indoor and outdoor air exchange on indoor air pollutant measurements.

2.7. Survey measurements

Table 2 summarises the four surveys (i.e. home survey, building audit, health and behaviour questionnaire, activity and symptom diary) which are available in Supplementary Files 1 and 2. These measurements have been developed based on validated questionnaires, existing evidence and input from experts in indoor air quality. Considering participant burden, these measurements (except the diary) are primarily administered online using REDCap (Research Electronic Data Capture) [22] which potentially reduces the

For peer review only - http://bmjopen.bmj.com/site/about/guidelines.xhtml

amount of time taken to complete the measurements (due to branching/skip logic) and aids understanding of questions (due to visuals), compared with the paper-and-pencil version. The online platform also reduces researcher burden and errors (entering and managing data).

For peer review only

For peer review only - http://bmjopen.bmj.com/site/about/guidelines.xhtml

Table 2. Summary of surveys

	Home survey	Building audit	Health & behaviour questionnaire	Activity and symptom diary
Completed on	Day 1	Day 1	Day 14	*Household behaviour*: one weekday & one weekend day (N=300); one day during the 72-hour monitoring period (N=150)<br>*Health symptoms* (N=300): Days 1-14<br>*Meals cooked at home* (N=150): Days 10/11-14
Completed by	Participant	Researcher	Participant	Participant
Content	*Section I: Home*	*Section I: House*	*Section I: Your perception of your home & air quality*	*Household behaviour*
	- Residence at the address	- Property type & age	- Home satisfaction	- Cooking
	- Housing tenure	- Building level	- Air quality perception	- Cleaning
	- Outdoor environment	- Energy Performance Certificate (EPC) rating	*Section II: You/your household's behaviour at home*	- Products
	- Number, type & state of rooms	- Outdoor environment	- Meal time	- Heating
	- Presence of mould & damp	- Number of windows & doors leading to the outside	- Use of non-stick cookware, personal care products, fragrances & cleaning products	- Ventilation
	- Heating type	- Sensor ID & location	- Frequency of dusting & vacuuming	- Personal care
	- Cooking fuel type	*Section II: Room*	- Method & location of washing & drying clothes	- Occupancy
	- Ventilation type	- Room size	- Use of solid fuel appliance	- Other experiences related to air quality
	- Presence of chimney	- Floor material	*Section III: Health*	*Individual* health symptom*
	- Home occupancy	- Widow direction	- Asthma (BiB child & parent)	- Time at home
	- Pet ownership	- Use of curtains/blinds/shutters	- Rhinitis (BiB child & parent)	- Asthma
	- Use of air purifier		- Eczema (BiB child & parent)	- Rhinitis
	Section II: Behaviour		- Behavioural screening (BiB parent)	- Eczema
	- Smoking habit		- Mental wellbeing (BiB parent)	- Mood

For peer review only

	Section IV: About you and your household	*Additional measurements*
	Household size	- Type of main meals cooked
	- Food security	
	- Financial security	

BiB=Born in Bradford. *Members of the family (e.g. BiB mother, BiB child).

For peer review only

For peer review only - http://bmjopen.bmj.com/site/about/guidelines.xhtml

2.7.1. Home survey

The 40-item home survey collects information on the condition and characteristics of the participant's home, the outdoor environment around the home, and people's smoking behaviours inside and outside the home (Supplementary File 1). The survey is self-administered electronically using a tablet or on paper by a participant during the first visit. The survey takes approximately 10 minutes to complete.

2.7.2. Building audit

The 32-item building audit collects information on the type of property and the characteristics of the rooms in which the sensors are installed known to affect indoor levels of air pollution such as room size, presence and placement of windows, and presence of extractor fans or hoods (Supplementary File 1). A trained researcher completes the audit electronically using a tablet or on paper during the first visit. The audit takes approximately 10 minutes to complete.

2.7.3. Health and behaviour questionnaire

The 250-item health and behaviour questionnaire assesses individual symptoms of child and parent respiratory and allergic health (International Study of Asthma and Allergies in Childhood [ISAAC] [23]; Global Asthma Network [24]), child behavioural screening (Strengths and Difficulties Questionnaire [25, 26]) and parent mental health (Short Warwick-Edinburgh Mental Wellbeing Scale [27]; Patient Health Questionnaire – Depression Module [28]), as well as socio-economic circumstances and household behaviours such as ventilation and cooking habits (Supplementary File 1). A participant completes the questionnaire electronically using a tablet or on paper on Day 14. The questionnaire takes approximately 20 minutes to complete.

2.7.4. Activity and symptom diary

Paper-based diaries are used to record key household activity patterns (28 items) and 14 individual health indicators of respiratory and allergic symptoms (based on ISAAC [23]) and mood (based on Visual Analogue Mood Scale [29] (Supplementary File 2). Individual health symptoms are self-reported by members of the family (e.g. BiB mother, BiB child) every day during the 2-week period. For household activity patterns, the participants are asked to select one weekday and one weekend day to record time spent doing key household activities that are related to indoor air pollution (e.g. cooking, cleaning, ventilation), and provide a description of relevant activities using free text. For the subset of participants who host a VOC canister and a PM sampler (N=150), activities are recorded for one additional day and main meals cooked at home are recoded every day during the 72-hour monitoring period.

2.8. Qualitative interview

Content for the semi-structured interviews was developed based on the COM-B (Capacity, Opportunity, Motivation – Behaviour) Model [30] which provides a systematic framework for understanding behaviours in terms of people's physical and psychological capability: physical and social opportunity; and automatic and reflexive motivation. The interview takes approximately 60 minutes, and is conducted either face-to-

For peer review only - http://bmjopen.bmj.com/site/about/guidelines.xhtml

face or online, depending on the participant's preference, and will be audio-recorded and transcribed. An interview guide is available on the Open Science Framework [31].

2.9. Data analysis plan

Quantitative data from the air quality monitors (i.e. the concentration of pollutants and compounds) and survey measurements will be reported descriptively using frequencies (for categorical variables), or medians and inter-quartile ranges or means and standard deviations (for continuous variables). Any qualitative data from the survey measurements (i.e. text) will be used as supplementary information for air pollution data. Any missing data of the variables measured and reasons for their absence will be examined, reported and handled appropriately in analyses. We do not expect any missing socio-demographic data as the data have already been collected through the BiB cohort study (e.g. Growing Up [11]). Based on the research questions, we will select appropriate statistical methods to analyse the data, as specified below.

2.9.1. What are the patterns of indoor air pollution in typical UK homes and are there inequalities in exposure to indoor air pollution?

Descriptive analyses of the AirGradient sensor data will be performed, including examining daily concentrations of indoor air pollution (means or medians), peaks in exposure and daily time spent above the World Health Organization (WHO) or UK recommendations for air pollution [32–34]. Compounds with known toxicological effects will be highlighted and compared to the WHO or UK guidelines for indoor air quality [32–34]. All the air pollution data including CO_2, VOCs and PM (concentration and composition) will be examined with all participants together as well as stratified by housing tenure (i.e. private/mortgaged, rented), ethnicity (i.e. South Asian, White British, Other), and Indices of Multiple Deprivation (IMD; in quintiles) in England (national) and Bradford (local). Differences within each variable will be examined using independent T-test, analysis of variance (ANOVA) or Wilcoxon-Mann-Whitney test.

2.9.2. How do physical characteristics of buildings or occupant behaviours contribute to indoor air pollution?

To identify subgroups of the participants based on shared factors, we aim to (i) identify latent profiles of building characteristics and occupant behaviours using latent class analysis, and (ii) examine whether these profiles are associated with indoor air pollution using mixed effect models, adjusting for covariates including housing tenure, ethnicity and English or Bradford IMD using regression models.

2.9.3. How are indoor air pollution and housing quality related to levels of respiratory symptoms and mental health?

Mixed effects models will be used to examine the relationship between changing indoor air pollution levels on respiratory symptoms and mood. The exposure variable will be measured as continuous (e.g. the daily and 2-week average concentration of indoor air pollution) or binary (e.g. the daily amount of time pollution levels exceeded the WHO or UK guidelines for indoor air quality [32, 34]). Daily occurrences of respiratory

For peer review only - http://bmjopen.bmj.com/site/about/guidelines.xhtml

symptoms (binary outcome: yes/no) and daily measures of mood (continuous outcome: 1-10) assessed using the symptom diaries will be examined using mixed effects logistic regression and mixed effects linear regression, respectively. Multivariable logistic regression, adjusting for relevant confounding variables, will be used to explore the association between indoor air pollution (i.e. 2-week average concentrations) and the health outcomes (i.e. the state of respiratory health and mental wellbeing derived from the health and behaviour questionnaire). Covariates will be included to reduce confounding and improve precision including subject-specific (e.g. age, sex, ethnicity, British or Bradford IMD, smoking habits) and time-dependent (e.g. day of the week, season, temperature, relative humidity).

To examine the lag relationship over time between exposure (indoor air pollution) and outcomes (respiratory symptoms and mood), we will assess the same day (lag 0), 1-day, 2-day and 3-day lags as well as the average of lag 0-3 days. Potential effect modification by housing quality (e.g. housing tenure, presence of damp) and season will be examined.

2.9.4. What are the barriers and enablers of ventilation behaviours in the home?

Anonymised interview transcripts will be coded and analysed using NVivo qualitative data analysis software [35] or paper-based techniques. A hybrid deductive/inductive content and thematic analysis approach will be employed, based on the methods described by Atkins et al. [36], Smith et al. [37] and Prothero et al. [38]. This will consist of two phases which entail development of an initial codebook based on the determinants of behaviour (i.e. domains) specified in the Theoretical Domains Framework (TDF) [39], mapped onto the COM-B framework [30]. In Phase 1, 10% of the transcripts will be independently deductively content analysed by two researchers, by coding text to the TDF domains in the codebook. We will also retain flexibility to identify additional codes not encompassed by the TDF during this stage, by adding these to the codebook where relevant. . Coding will then be discussed by the two researchers, with the aim of building consensus, and refining the codebook. Where consensus cannot be reached, a third researcher will be consulted, or the relevant text will be attributed to more than one TDF domain. The first researcher will then use the revised codebook to code the remainder of the transcripts, adding and discussing any revisions or additional codes with the second researcher or broader team as needed.

In Phase 2, an inductive thematic analysis will be conducted on the text marked with codes during Phase 1, following the process identified by Braun and Clarke [40] and focused on identifying specific barriers and enablers of ventilation behaviours. Ten percent of transcripts will be independently coded by two researchers, and an initial list of inductive codes will be added to each researcher's codebook under the code headings from Phase 1. The first researcher will then use the revised codebook to code the remainder of the transcripts, adding and discussing any revisions or additional codes with the second researcher or broader team as needed. Themes will then be generated, reviewed and defined by the first researcher, in discussion with the second researcher or broader team. Content relating to current ventilation behaviours will be summarised descriptively. Potential targets for intervention will then be prioritised by the first researcher in discussion with the broader team, based on the following criteria: (1) frequency of theme (i.e. number of participants reporting a specific barrier or enabler); (2) number of barrier or enabler themes per

For peer review only - http://bmjopen.bmj.com/site/about/guidelines.xhtml

TDF domain (where relevant); (3) discordance within or across themes (i.e. the same factor acting as a barrier and facilitator of behaviour in different participants or in different contexts); (4) perceived importance of theme for participants.

2.10. Patient and public involvement

In Real Home Assessment, public involvement has been central to the development of the study and has been embedded within all research activities including the design, conduct and dissemination of the study. BiB has long-established public participation groups (e.g. parent governors, public research advisory group) which meet regularly to provide lived experience, expertise and guidance to the research team. The groups, for example, provided us feedback on the feasibility of the survey measurements (e.g. item wording, survey length) during the pilot testing. We have used a variety of different channels to give members of the public a chance to engage in the air pollution research, including hosting regular 'open space' community meetings, attending existing community events (e.g. science festivals), and circulating briefing notes and newsletters. For study participants, we provide a personalised feedback report (Supplementary File 3) after the completion of the data monitoring period.

2.11 Ethics and dissemination

Ethical approval has been obtained from the National Health Service Health Research Authority Yorkshire and the Humber (Bradford Leeds) Research Ethics Committee: 22/YH/0288, 11th January 2023. Participants give informed consent by receiving an information sheet and signing a consent form after they have had the opportunity to discuss the project with a member of the research team. We will disseminate research outputs using various channels including our INGENIOUS project website [41], BiB website [42], social media, scientific publications, press releases as well as conference presentations and posters. Air quality and health data will be open access via existing Born in Bradford data access procedure [42]. An open-access dataset for the qualitative interviews (i.e. anonymised transcripts) will be also made available on the UK Data Service, and the interview materials are available on the Open Science Framework [31].

3. Discussion

Exposure to airborne pollutants during childhood can increase the risk and exacerbation of airway diseases with lifelong consequences [43, 44]. Yet, there is little evidence to constrain or quantify indoor air pollutant emissions, building-to-building variability, chemical speciation of indoor air pollutants, interactions between indoor- and outdoor-generated air pollution, or environmental and socio-economic factors that can contribute to elevated indoor air pollutant exposures. Real Home Assessment as part of the INGENIOUS project is one of the first research studies that measures indoor air pollution in UK homes at a large scale. Findings from the Real Home Assessment will contribute to the understanding of how indoor air pollution in UK homes is generated, prevented and reduced, as well as insights into social, ethnic and health inequalities associated with indoor air pollution.

For peer review only - http://bmjopen.bmj.com/site/about/guidelines.xhtml
For peer review only

Deprived households are more likely to experience poor indoor air quality than more affluent households due to factors such as greater exposure to second hand smoke and higher outdoor air pollutant concentrations [2]. However, the reality may be considerably more nuanced. On one hand, lower quality and older housing may be less airtight (i.e. less energy efficient) than new-build homes allowing outdoor air pollution to penetrate inside but indoor emissions to escape more easily. On the other hand, large, expensive town houses converted to flats can be poorly ventilated, and suffer from higher indoor-generated air pollutant concentrations following poor retrofitting practices [2].

Recruiting households from the BiB cohort in the current study is valuable in terms of the diversity of the cohort (covering a multi-ethnic population that has high rates of deprivation) and the detailed data collection (including information about the health, social circumstances and lifestyle characteristics of children). Over 30% of BiB families live in a rented property, one-third of families have more than five occupants, and 13% of families have at least one child with doctor-diagnosed asthma (based on the Growing Up data [11]). This profile makes the BiB cohort a particularly relevant population group in which to explore patterns of indoor air pollution.

The tiered approach of air quality monitoring allows us to provide both summary (with common pollutant concentrations) and detailed (with a large number of pollutant speciation) assessments of indoor air quality. Indoor air quality and health is an under-researched area, and this study will be one of the largest to date will collect detailed measures of indoor air pollution and health outcomes [45]. Along with the air quality monitors, we use four survey measurements to capture potential contributing emission sources of indoor air pollution from the building and occupant behaviours. Results from the semi-structured interviews will be used to inform the design of behaviour change interventions to reduce exposure to indoor air pollutants. These interventions will be co-designed with community members and evaluated in a later stage of the INGENIOUS project and will be described in a separate protocol.

Real Home Assessment is a cross-sectional study which captures a snapshot of indoor air pollutant concentrations and occupant behaviours and health symptoms over a 2-week period. The sample size of 300 families was selected to maximise the breadth of data and be deliverable within the funded project timelines. Recruiting participants and collecting data (including air pollutant concentrations and occupant behaviours) in the domestic setting can be more challenging and resource-intensive than doing so in public spaces and measuring outdoor air pollution through national and local authorities [7]. Thus, we consider Real Home Assessment as exploratory research to assess the feasibility of collecting indoor air pollution in homes, which is likely to be the primary indoor environment where most people spend their time. However, we cannot ignore air pollution exposures in other indoor settings such as schools, workplaces, transport and shops. Future research will aim to capture total personal exposure using multi-pollutant sensor platforms [46] and advanced computational modelling for time-activity patterns [47]. Given the burdensome nature of data collection, it will be important for researchers in this area to harmonise methods to allow combining data from similar studies across the UK. We publish this protocol and share our methods as a first step in this wider endeavour.

For peer review only - http://bmjopen.bmj.com/site/about/guidelines.xhtml

References

1. Office for Health Improvement & Disparities (2022) Air pollution: Applying all our health. https://www.gov.uk/government/publications/air-pollution-applying-all-our-health/air-pollution-applying-all-our-health. Accessed 12 Sep 2023
2. Laverge J, Delghust M, Janssens A (2015) Carbon dioxide concentrations and humidity levels measured in Belgian standard and low energy dwellings with common ventilation strategies. Int J Vent 14:165–180
3. Cohen AJ, Brauer M, Burnett R, et al (2017) Estimates and 25-year trends of the global burden of disease attributable to ambient air pollution: an analysis of data from the Global Burden of Diseases Study 2015. Lancet 389:1907–1918
4. Dominski FH, Lorenzetti Branco JH, Buonanno G, Stabile L, Gameiro da Silva M, Andrade A (2021) Effects of air pollution on health: A mapping review of systematic reviews and meta-analyses. Environ Res 201:111487
5. Klepeis NE, Nelson WC, Ott WR, Robinson JP, Tsang AM, Switzer P, Behar J V., Hern SC, Engelmann WH (2001) The National Human Activity Pattern Survey (NHAPS): A resource for assessing exposure to environmental pollutants. J Expo Anal Environ Epidemiol 11:231–252
6. Department of Health and Social Care (2022) Chief Medical Officer's annual report 2022: Air pollution.
7. Air Quality Expert Group (2022) Indoor air quality. https://doi.org/10.5281/zenodo.6523605
8. City of Bradford Metropolitan District Council (2022) Population. https://ubd.bradford.gov.uk/about-us/population/.
9. Noble S, McLennan D, Noble M, Plunkett E, Gutacker N, Silk M, Wright G (2019) The English Indices of Deprivation 2019: Research report.
10. Wright J, Small N, Raynor P, et al (2013) Cohort profile: The born in bradford multi-ethnic family cohort study. Int J Epidemiol 42:978–991
11. Bird PK, McEachan RRC, Mon-Williams M, et al (2019) Growing up in Bradford: Protocol for the age 7-11 follow up of the Born in Bradford birth cohort. BMC Public Health 19:1–12
12. Royal College of Physicians (2016) Every breath we take: The lifelong impact of air pollution. London, UK
13. AirGradient Limited (2023) AirGradient. https://www.airgradient.com/. Accessed 22 Sep 2023
14. The Chartered Institution of Building Services Engineers (CIBSE) (2020) TM61 Operational performance of buildings. London, UK
15. Warburton T, Grange SK, Hopkins JR, Andrews SJ, Lewis AC, Owen N, Jordan C, Adamson G,

For peer review only

Xia B (2023) The impact of plug-in fragrance diffusers on residential indoor VOC concentrations. Environ Sci Process Impacts 25:805–817

16. Lopez-Hilfiker FD, Mohr C, Ehn M, et al (2014) A novel method for online analysis of gas and particle composition: Description and evaluation of a filter inlet for gases and AEROsols (FIGAERO). Atmos Meas Tech 7:983–1001

17. Lee BH, Lopez-Hilfiker FD, Mohr C, Kurtén T, Worsnop DR, Thornton JA (2014) An iodide-adduct high-resolution time-of-flight chemical-ionization mass spectrometer: Application to atmospheric inorganic and organic compounds. Environ Sci Technol 48:6309–6317

18. Jimenez JL, Jayne JT, Shi Q, et al (2003) Ambient aerosol sampling using the Aerodyne aerosol mass spectrometer. J Geophys Res Atmos 108:1–13

19. Stewart GJ, Nelson BS, Acton WJF, et al (2021) Emissions of intermediate-volatility and semi-volatile organic compounds from domestic fuels used in Delhi, India. Atmos Chem Phys 21:2407–2426

20. EarthSense Systems Limited (2023) Zephyr. https://www.earthsense.co.uk/zephyr. Accessed 22 Sep 2023

21. Environmental Instruments Limited (2023) AQMesh. https://www.aqmesh.com/. Accessed 22 Sep 2023

22. Vanderblit University (2023) REDCap. https://projectredcap.org/. Accessed 27 Sep 2023

23. Ellwood P, Asher MI, Beasley R, Clayton TO, Stewart a W (2000) Phase Three Manual of the International Study of Asthma and Allergies in Childhood (ISAAC). Int J Tuberc Lung Dis 9:10–16

24. Global Asthma Network (2015) Phase I Manual: Global surveillance: prevalence, severity, management and risk factors. Auckland, New Zealand

25. Goodman R (1997) The strengths and difficulties questionnaire: A research note. J Child Psychol Psychiatry 38:581–586

26. Goodman R (1999) The extended version of the Strengths and Difficulties Questionnaire as a guide to child psychiatric caseness and consequent burden. J Child Psychol Psychiatry 40:791–799

27. Shah N, Cader M, Andrews B, McCabe R, Stewart-Brown SL (2021) Short Warwick-Edinburgh Mental Well-being Scale (SWEMWBS): performance in a clinical sample in relation to PHQ-9 and GAD-7. Health Qual Life Outcomes 19:1–9

28. Kroenke K, Spitzer RL, Williams JBW (2001) The PHQ-9: Validity of a brief depression severity measure. J Gen Intern Med 16:606–613

29. van Rijsbergen GD, Bockting CLH, Berking M, Koeter MWJ, Schene AH (2012) Can a one-item mood scale do the trick? Predicting relapse over 5.5-years in recurrent depression. PLoS One 7:1–5

For peer review only - http://bmjopen.bmj.com/site/about/guidelines.xhtml

30. Michie S, van Stralen MM, West R (2011) The behaviour change wheel: A new method for characterising and designing behaviour change interventions. Implement Sci 6:1–11

31. Wood C, Genes D (2023) INGENIOUS: Co-production and evaluation of behaviour change interventions to improve indoor air quality in the UK. https://osf.io/sf9np/?view_only=76c443e757f84ecbb16f6705ebe0766a. Accessed 6 Oct 2023

32. World Health Organization (2010) WHO guidelines for air quality: selected pollutants. Copenhagen, Denmark

33. World Health Organization (2021) WHO global air quality guidelines: Particulate matter (PM2.5 and PM10), ozone, nitrogen dioxide, sulfur dioxide and carbon monoxide. Geneva, Switzerland

34. Public Health England (2019) Indoor Air Quality Guidelines for selected Volatile Organic Compounds (VOCs) in the UK About Public Health England. London, UK

35. Lumivero (2023) NVivo. https://lumivero.com/products/nvivo/. Accessed 6 Oct 2023

36. Atkins L, Francis J, Islam R, et al (2017) A guide to using the Theoretical Domains Framework of behaviour change to investigate implementation problems. Implement Sci 12:1–18

37. Smith D, Cartwright M, Dyson J, Hartin J, Aitken LM (2021) Barriers and enablers of recognition and response to deteriorating patients in the acute hospital setting: A theory-driven interview study using the Theoretical Domains Framework. J Adv Nurs 77:2831–2844

38. Prothero L, Lawrenson JG, Cartwright M, et al (2022) Barriers and enablers to diabetic eye screening attendance: An interview study with young adults with type 1 diabetes. Diabet Med 39:1–16

39. Cane J, O'Connor D, Michie S (2012) Validation of the theoretical framework. Implement Sci 7:1–17

40. Braun V, Clarke V (2006) Using thematic analysis in psychology. Qual Res Psychol 3:77–101

41. INGENIOUS (2023) INGENIOUS. https://ingenious.york.ac.uk/. Accessed 22 Sep 2023

42. Born in Bradford (2023) Born in Bradford. https://borninbradford.nhs.uk/. Accessed 22 Sep 2023

43. Kelly FJ, Fussell JC (2011) Air pollution and airway disease. Clin Exp Allergy 41:1059–1071

44. Raju S, Siddharthan T, McCormack MC (2020) Outdoor air pollution and respiratory health. Clin Chest Med 41:825–843

45. Parliamentary Office of Science and Technology (POST) (2023) POSTbrief 54: Indoor air quality. https://doi.org/10.58248/PB54

46. Chatzidiakou L, Krause A, Popoola O, et al (2019) Characterising low-cost sensors in highly portable platforms to quantify personal exposure in diverse environments. Atmos Meas Tech 12:4643–4657

For peer review only

For peer review only - http://bmjopen.bmj.com/site/about/guidelines.xhtml

47. Chatzidiakou L, Krause A, Kellaway M, Han Y, Li Y, Martin E, Kelly FJ, Zhu T, Barratt B, Jones RL (2022) Automated classification of time-activity-location patterns for improved estimation of personal exposure to air pollution. Environ Heal 21:1–21

For peer review only

Acknowledgement

Born in Bradford is only possible because of the enthusiasm and commitment of the children and parents in BiB. We are grateful to all the participants, health professionals, schools and researchers who have made Born in Bradford happen. We would like to acknowledge Kane Armatage and Sally Jones from the City of Bradford Metropolitan District Council for their involvement in outdoor air quality monitoring.

Declarations

Authors' contributions

All authors made substantial contributions to the conception or design of the work, and read and approved the final manuscript. EI, CW, LC, TW, AR, YS, DG, DW, SPO, PE, EB and DRS contributed to the acquisition, analysis or interpretation of data. EI drafted the manuscript, and JH, CW, LC, TW, AR, YS, DG, DW, TCY, CG, GM, SPO, PE, EB, DRS, NC and RRCM reviewed and revised it.

Funding

INGENIOUS is funded by UK Research and Innovation Natural Environment Research Council (NE/W002256/1) and has undergone a rigorous peer-review process as part of the application process. RM received funding from the National Institute for Health and Care Research Applied Collaboration for Yorkshire and Humber (NHIR200166). AR is funded by the Natural Environment Research Council PANORAMA Doctoral Training Program. The Zephyr sensor network utilised in the project is operated by City of Bradford Metropolitan District Council and was purchased using a Department for Environment, Food and Rural Affairs Air Quality Grant obtained by the local authority. The funders have no role in the design of the study, the collection, analysis and interpretation of the data, the writing of the manuscript, and the decision to submit the manuscript for publication. The views expressed in this publication are those of the author(s) and not necessarily those of the National Institute for Health and Care Research or the Department of Health and Social Care.

Competing interests

The authors declare that they have no competing interests.

For peer review only

For peer review only - http://bmjopen.bmj.com/site/about/guidelines.xhtml

INGENIOUS

Survey measurements

V2.0 (17/03/2023)

This document includes: Home survey on Day 1, Building audit on Day 1, and Health and behaviour questionnaire on Day 14.

Contents

Home Survey....2
Section I: Home....2
Section II: Behaviour....7
Building Audit....9
Section I: House....9
Section II: Room....12
Health & Behaviour Questionnaire....15
Section I: Your Perception of Your Home and Air Quality....15
Section II: You/Your Household's Behaviour at Home....16
Section III: Health....23
Section IV: About You and Your Household....29

For peer review only

Home Survey

Note to Research Assistant:

Please enter participant ID and write today's date BEFORE giving the survey to participant.

*Please enter participant ID (i.e. ING…..): ___________

*Please write today's date: ___/___/_____ (dd/mm/yyyy)

*must provide value

For peer review only

Dear Participant,

We are interested to know about your home. We would be grateful if you help us by answering as many of these questions as possible. All the answers you give are confidential.

Thank you for taking part in the INGENIOUS study!

INGENIOUS

We are family BORNINBRADFORD

Section I: Home

This section is about the house you live in at the moment.

1	*How long have you lived at your current home?	Years: _____<br>Months: _____
2	*Does your household own or rent this accommodation?	○ I/we own it or live with the person who owns it (includes homes bought with a mortgage)<br>○ It is rented from the local council

For peer review only - http://bmjopen.bmj.com/site/about/guidelines.xhtml

		○ It is rented from a housing association or housing trust<br>○ It is rented from a private landlord<br>○ Other (e.g. I live here rent free or home comes with job)
3a	*How far away from this home is the nearest greenspace area (e.g. park, playing field, public garden, woodland or other green space), regardless of size and function?	○ Less than 5 min walk<br>○ 5-10 min walk<br>○ 11-20 min walk<br>○ 21-30 min walk<br>○ More than 30 min walk<br>○ Don't know
3b	*Do you have a garden or a yard in your home?	○ Yes<br>○ No<br>○ Don't know
3c	*Is your home nearby (i.e. within 5-min walking distance):<br>(Tick all that apply)	☐ Busy road<br>☐ Motorway<br>☐ Car park<br>☐ Petrol station<br>☐ Industry (e.g. factory)<br>☐ Waste management site<br>☐ Agricultural land<br>☐ Construction<br>☐ Airport<br>☐ Restaurant or takeaway<br>☐ None of the above<br>☐ Don't know
4	*Do you have an attached garage in your home?	○ Yes<br>○ No<br>○ Don't know
5a	*How many *separate* living or dining rooms does your home have?<br>*Only count rooms that are closed off/separated by a door*	_____
5b	*When was the floor of the living or dining room laid (where a sensor will be placed)?	○ In the last 6 months<br>○ 6 months to 1 year ago<br>○ More than 1 year ago<br>○ Don't know
5c	*In the past 12 months, has the interior of the living or dining room (where a sensor will be placed) been refurbished, renovated or painted?	○ Yes<br>○ No<br>○ Don't know
6a	*How many *separate* kitchens does your home have?<br>*Only count rooms that are closed off/separated by a door*	_____

For peer review only - http://bmjopen.bmj.com/site/about/guidelines.xhtml

For peer review only

6b	*When was the floor of the kitchen laid (where a sensor will be placed)?	○ In the last 6 months<br>○ 6 months to 1 year ago<br>○ More than 1 year ago<br>○ Don't know
6c	*Which floor is the kitchen on (where a sensor will be placed)?	○ Property is on one level<br>○ Basement<br>○ Ground floor<br>○ First floor<br>○ Second floor<br>○ Third floor<br>○ Don't know
6d	*In the past 12 months, has the interior of the kitchen (where a sensor will be placed) been refurbished, renovated or painted?	○ Yes<br>○ No<br>○ Don't know
7a	*How many *separate* bedrooms does your home have?<br>*Only count rooms that are closed off/separated by a door*	______
7b	*When was the floor of the bedroom laid (where a sensor will be placed)?	○ In the last 6 months<br>○ 6 months to 1 year ago<br>○ More than 1 year ago<br>○ Don't know
7c	*In the past 12 months, has the interior of the bedroom (where a sensor will be placed) been refurbished, renovated or painted?	○ Yes<br>○ No<br>○ Don't know
7d	*How many people share the bedroom where a sensor will be placed?	______
8a	*How many *separate* bathrooms does your home have?<br>*Only count rooms that are closed off/separated by a door (e.g. toilet)*	______
8b	*Which floors are main (i.e. regularly used) bathrooms on?<br>(Tick all that apply)	☐ Property is on one level<br>☐ Basement<br>☐ Ground floor<br>☐ First floor<br>☐ Second floor<br>☐ Third floor<br>☐ Don't know
9a	*Are you aware of any signs or smells of mould or damp in your home?	○ Yes<br>○ No (Go to Q10)<br>○ Don't know (Go to Q10)
9b	*In which rooms are there signs or smells of mould or damp?<br>(Tick all that apply)	☐ Living/dining room<br>☐ Kitchen

For peer review only - http://bmjopen.bmj.com/site/about/guidelines.xhtml

		☐ Adult's bedroom<br>☐ Child's bedroom<br>☐ Bathroom<br>☐ Basement<br>☐ Other (write in) ____________________
10	*What is the most common type of windows in your whole house?	○ Single glazed windows<br>○ Double or triple glazed windows<br>○ Don't know
11a	*How do you heat your home?<br>(Tick all that apply)	☐ Gas central heating<br>☐ Oil central heating<br>☐ Fixed gas fire<br>☐ Fixed electric heating<br>☐ Night storage heaters<br>☐ Open fires<br>☐ Wood stoves (solid fuel/wood/coal)<br>☐ Pellet boiler<br>☐ Portable heaters: electric, bottled gas/paraffin, oil-filled<br>☐ Other (write in) ____________________<br>☐ Don't know
12a	*What fuel do you use for cooking?<br>(Tick all that apply)	☐ Gas mains<br>☐ Gas bottles<br>☐ Electricity<br>☐ Other (write in) ____________________<br>☐ Don't know

The next question is about ventilation options (please see the photo as examples).

For peer review only

For peer review only - http://bmjopen.bmj.com/site/about/guidelines.xhtml

Extractor hood:

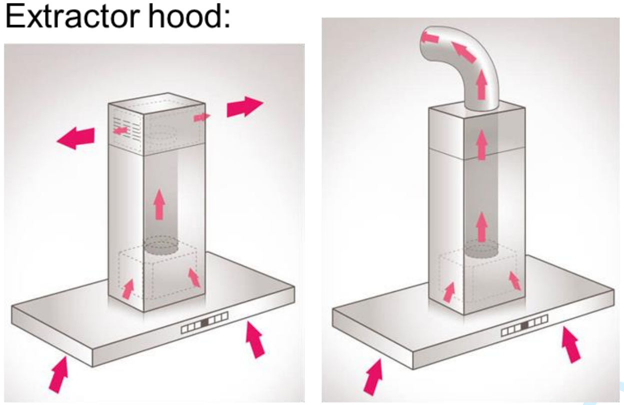

(to inside) (to outside)

Extractor fan:

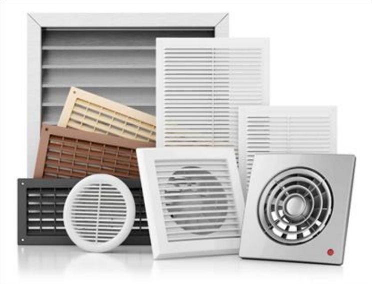

Trickle vents:

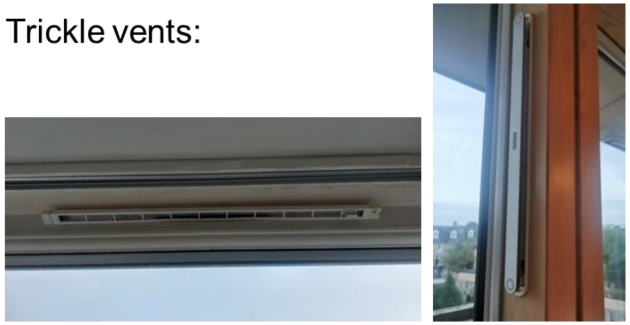

For peer review only

13a	*Does your home have trickle vents?	○ Yes<br>○ No (Go to Q15)<br>○ Don't know (Go to Q15)
13b	*Are the trickle vents open or closed?	○ Trickle vents are always open<br>○ Trickle vents are always closed<br>○ Trickle vents are open or closed depending on the season<br>○ Don't know
14a	*What options are available for ventilating the kitchen? (Tick all that apply)	☐ Extractor hood to inside<br>☐ Extractor hood/fan to outside<br>☐ Window<br>☐ Door to outside<br>☐ Trickle vent<br>☐ There is no means of ventilation<br>☐ Don't know
14b	*What options are available for ventilating the main bathroom/toilet? (Tick all that apply)	☐ Extractor fan<br>☐ Window<br>☐ Trickle vent<br>☐ There is no means of ventilation<br>☐ Don't know
15	*On a normal day, how often are doors to the outside opened and closed?	○ 0-2 times<br>○ 3-10 times<br>○ 11-20 times<br>○ More than 20 times<br>○ Don't know

For peer review only - http://bmjopen.bmj.com/site/about/guidelines.xhtml

16	*Does your home have a chimney that is *not blocked off*? *Air and smoke can flow out of the chimney*	○ Yes<br>○ No (including using a 'chimney sheep')<br>○ My home doesn't have a chimney<br>○ Don't know
17	*When are people normally in your home? (Tick all that apply)	☐ During the day / school time (9:00-14:59 [24-hour clock])<br>☐ After school (15:00-17:59 [24-hour clock])<br>☐ Evening (18:00-22:59 [24-hour clock])<br>☐ Night (23:00-8:59 [24-hour clock])
18a	*Are there any pets in your household?	○ Yes<br>○ No (Go to Q19a)<br>○ Don't know (Go to Q19a)
18b	*How many pet(s) do you own?	______
19a	*Do you have an air purifier in your home?	○ Yes<br>○ No (Go to Q20a)<br>○ Don't know (Go to Q20a)
19b	What brand or model of air purifier do you have?	____________________

Section II: Behaviour

This section is about smoking behaviour.

1a	*How often do people smoke cigarettes, e-cigarettes, cigar or pipes inside this home?	○ Every day<br>○ Multiple times a week<br>○ Once or twice a week<br>○ Less than once a week<br>○ Never<br>○ Don't know
1b	*How often do people smoke cigarettes, e-cigarettes, cigar or pipes within a close area to this home (e.g. courtyard, with window open, stairwell)?	○ Every day<br>○ Multiple times a week<br>○ Once or twice a week<br>○ Less than once a week<br>○ Never<br>○ Don't know

Thank you for participation.

If you have any questions, please contact us: 01274 364474 or borninbradford@bthft.nhs.uk

For peer review only - http://bmjopen.bmj.com/site/about/guidelines.xhtml

For peer review only

For peer review only - http://bmjopen.bmj.com/site/about/guidelines.xhtml

Building Audit

Please complete the audit below.
Thank you!

*Please enter participant ID (i.e. ING…..): ___________

*Please write today's date: ___/___/_____ (dd/mm/yyyy)

*must provide value

Section I: House

This section is about the property the participant lives in at the moment.

1	*What type of home is this?	○ Detached house<br>○ Semi-detached house<br>○ Bungalow<br>○ Terraced (including end terrace)<br>○ Flat, maisonette or apartment (in a purpose - built block of flats or tenement)<br>○ Flat, maisonette or apartment (part of converted or shared house, including bedsits)<br>○ Flat, maisonette or apartment (part of another converted building, e.g. former school, church or warehouse)<br>○ Flat, maisonette or apartment (in a commercial building, e.g. in an office building, hotel or over a shop)
2	*When was this home built?	○ Before 1914<br>○ 1914-1944

For peer review only - http://bmjopen.bmj.com/site/about/guidelines.xhtml

For peer review only

		○ 1945-1964<br>○ 1965-1980<br>○ 1981-1990<br>○ 1991-2002<br>○ 2003 or later<br>○ Don't know
3	*How many floors does the property have?	_____
4	*Which floor does the participant live on?<br>(-1 = Basement, 0 = Ground floor, 1 = First floor, 2 = Second floor, etc.)<br><br>*If the participant lives on more than one floors, use comma (,) to list all the floors (e.g. -1, 0, 1).*	__________
5	*What is the energy efficiency rating of the building (which is shown in an energy performance certificate [EPC])?<br><br>*If the participant doesn't know the rating, find an energy certificate using postcode via https://www.gov.uk/find-energy-certificate.*	○ A<br>○ B<br>○ C<br>○ D<br>○ E<br>○ F<br>○ G<br>○ The building has not been certified<br>○ Don't know
6a	*Do any of the windows in the living/dining room (where a sensor will be placed) look out mainly onto grey space, including man-made materials (e.g. roads, buildings)?	○ Yes, at least one window has a main view of grey space<br>○ No, they have a mixed view of grey and green space<br>○ No, they have a main view of green space, including parks and gardens (either public or private) regardless of size and function<br>○ Don't have access to living/dining room
6b	*Do any of the windows in the kitchen (where a sensor will be placed) look out mainly onto grey space, including man-made materials (e.g. roads, buildings)?	○ Yes, at least one window has a main view of grey space<br>○ No, they have a mixed view of grey and green space<br>○ No, they have a main view of green space, including parks and gardens (either public or private) regardless of size and function<br>○ Don't have access to kitchen
7	*How many windows (which can be opened) does the property have?	_____
8a	*How many doors lead to the outside?	_____

The next question is about draft excluders (please see the photo as examples).

For peer review only - http://bmjopen.bmj.com/site/about/guidelines.xhtml

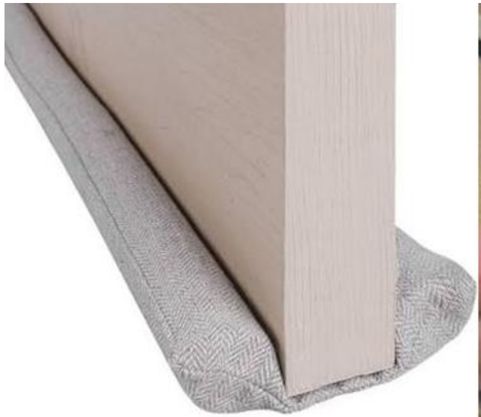

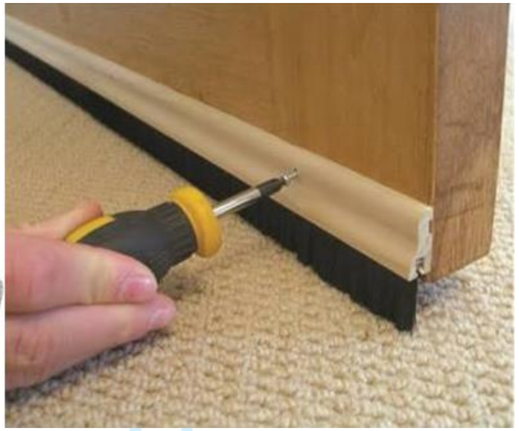

8b	*Are draft excluders attached to outside doors?	○ Yes, all of the doors<br>○ Yes, some of the doors<br>○ No<br>○ Don’t have access to outside doors
9a	*Please enter the first sensor's ID:	___________
9b	*Which room is the first sensor installed?	○ Living/dining room<br>○ Kitchen<br>○ Child’s bedroom<br>○ Adult’s bedroom
9c	*Which floor is the first sensor installed?	○ Basement<br>○ Ground floor<br>○ First floor<br>○ Second floor<br>○ Third floor<br>○ Property is on one level
9d	*Please enter the second sensor's ID:	___________
9e	*Which room is the second sensor installed?	○ Living/dining room<br>○ Kitchen<br>○ Child’s bedroom<br>○ Adult’s bedroom<br>○ The second sensor is not installed
9f	*Which floor is the second sensor installed?	○ Basement<br>○ Ground floor<br>○ First floor<br>○ Second floor<br>○ Third floor

For peer review only - http://bmjopen.bmj.com/site/about/guidelines.xhtml

		○ Property is on one level<br>○ The second sensor is not installed
9g	*Please enter the third sensor's ID:	___________
9h	*Which room is the third sensor installed?	○ Living/dining room<br>○ Kitchen<br>○ Child's bedroom<br>○ Adult's bedroom<br>○ The third sensor is not installed
9i	*Which floor is the third sensor installed?	○ Basement<br>○ Ground floor<br>○ First floor<br>○ Second floor<br>○ Third floor<br>○ Property is on one level<br>○ The third sensor is not installed

Section II: Room

This section is about the rooms the property has.

1a	What is the dimension of the living/dining room (where a sensor will be placed)?	Length: _____ metre<br>Width: _____ metre<br>Height: _____ metre
1b	*What material is the floor of the living/dining room (where a sensor will be placed)?<br>(Tick all that apply)	☐ Carpet/rug<br>☐ Engineered wood/solid wood<br>☐ Vinyl/laminate<br>☐ Tile<br>☐ There is no living/dining room
1c	*Which direction does the window(s) of the living/dining room face (where a sensor will be placed)?<br>(Tick all that apply if multiple windows face multiple directions)<br><br>*Please round to the nearest cardinal direction.*	☐ North<br>☐ North East<br>☐ East<br>☐ South East<br>☐ South<br>☐ South West<br>☐ West<br>☐ North West<br>☐ There is no window in the living/dining room
1d	*Does the living/dining room (where a sensor will be placed) have curtains/blinds/shutters?	☐ Yes, all of the windows<br>☐ Yes, some of the windows

For peer review only - http://bmjopen.bmj.com/site/about/guidelines.xhtml

		☐ No<br>☐ There is no window in the living/dining room
2a	What is the dimension of the kitchen (where a sensor will be placed)?	Length: _____ metre<br>Width: _____ metre<br>Height: _____ metre
2b	*What material is the floor of the kitchen (where a sensor will be placed)?<br>(Tick all that apply)	☐ Carpet/rug<br>☐ Engineered wood/solid wood<br>☐ Vinyl/laminate<br>☐ Tile<br>☐ There is no kitchen
2c	*Which direction does the window(s) of the kitchen face (where a sensor will be placed)?<br>(Tick all that apply if multiple windows face multiple directions)<br><br>*Please round to the nearest cardinal direction.*	☐ North<br>☐ North East<br>☐ East<br>☐ South East<br>☐ South<br>☐ South West<br>☐ West<br>☐ North West<br>☐ There is no window in the kitchen
2d	*Does the kitchen (where a sensor will be placed) have curtains/blinds/shutters?	☐ Yes, all of the windows<br>☐ Yes, some of the windows<br>☐ No<br>☐ There is no window in the kitchen
2e	*Is the kitchen in open-plan or attached to the living/dining room?	☐ Yes<br>☐ No<br>☐ There is no kitchen
3a	What is the dimension of the bedroom (where a sensor will be placed)?	Length: _____ metre<br>Width: _____ metre<br>Height: _____ metre
3b	*What material is the floor of the bedroom (where a sensor will be placed)?<br>(Tick all that apply)	☐ Carpet/rug<br>☐ Engineered wood/solid wood<br>☐ Vinyl/laminate<br>☐ Tile<br>☐ There is no bedroom
3c	*Which direction does the window(s) of the bedroom face (where a sensor will be placed)?<br>(Tick all that apply if multiple windows face multiple directions)	☐ North<br>☐ North East<br>☐ East<br>☐ South East

For peer review only

	Please round to the nearest cardinal direction.	☐ South<br>☐ South West<br>☐ West<br>☐ North West<br>☐ There is no window in the bedroom
3d	*Does the bedroom (where a sensor will be placed) have curtains/blinds/shutters?	☐ Yes, all of the windows<br>☐ Yes, some of the windows<br>☐ No<br>☐ There is no window in the bedroom

Thank you for completing the audit.

For peer review only

For peer review only - http://bmjopen.bmj.com/site/about/guidelines.xhtml

Health & Behaviour Questionnaire

Note to Research Assistant:

Please enter participant ID and write today's date BEFORE giving the survey to participant.

*Please enter participant ID (i.e. ING…..): ___________

*Please write today's date: ___/___/_____ (dd/mm/yyyy)

*must provide value

Dear Participant,

We are interested to understand your perception of your home, your/your household's behaviour at home, and your/your child's health and wellbeing. We would be grateful if you could help us by answering as many of these questions as possible. All the answers you give are confidential.

Thank you for taking part in the INGENIOUS study!

For peer review only

Section I: Your Perception of Your Home and Air Quality

		Very dissatisfied	Fairly dissatisfied	Neither satisfied nor dissatisfied	Fairly satisfied	Very satisfied
1a	*In general, how satisfied or dissatisfied are you with the general condition of your home?	○	○	○	○	○

For peer review only - http://bmjopen.bmj.com/site/about/guidelines.xhtml

1b	*Overall, how satisfied or dissatisfied are you with your home as a place to live?	○	○	○	○	○
1c	*Overall, how satisfied or dissatisfied are you with the quality of the air inside your home?	○	○	○	○	○
		Very poor	Poor	Neither good nor poor	Good	Very good
2a	*How do you rate the quality of the air in your home?	○	○	○	○	○
2b	*How do you rate the quality of the air in your street?	○	○	○	○	○

Section II: You/Your Household's Behaviour at Home

1a	*Do you/people in your household usually have breakfast at home <u>on weekdays</u>?	○ Yes<br>○ No (Go to Q1c)<br>○ Don't know (Go to Q1c)
1b	What time do you/people in your household usually have breakfast at home <u>on weekdays</u>?<br>(24-hour clock: 6:00=6:00 a.m., 18:00=6:00 p.m.)	___:___
1c	*Do you/people in your household usually have breakfast at home <u>on the weekend</u>?	○ Yes<br>○ No (Go to Q1e)<br>○ Don't know (Go to Q1e)
1d	What time do you/people in your household usually have breakfast at home <u>on the weekend</u>?<br>(24-hour clock: 6:00=6:00 a.m., 18:00=6:00 p.m.)	___:___
1e	*Do you/people in your household usually have lunch at home <u>on weekdays</u>?	○ Yes<br>○ No (Go to Q1g)<br>○ Don't know (Go to Q1g)
1f	What time do you/people in your household usually have lunch at home <u>on weekdays</u>?<br>(24-hour clock: 1:00=1:00 a.m., 13:00=1:00 p.m.)	___:___
1g	*Do you/people in your household usually have lunch at home <u>on Saturdays</u>?	○ Yes<br>○ No (Go to Q1i)<br>○ Don't know (Go to Q1i)
1h	What time do you/people in your household usually have lunch at home <u>on Saturdays</u>?<br>(24-hour clock: 1:00=1:00 a.m., 13:00=1:00 p.m.)	___:___
1i	*Do you/people in your household usually have lunch at home <u>on Sundays</u>?	○ Yes<br>○ No (Go to Q1k)<br>○ Don't know (Go to Q1k)

For peer review only

1j	What time do you/people in your household usually have lunch at home on Sundays?<br>(24-hour clock: 1:00=1:00 a.m., 13:00=1:00 p.m.)	___:___
1k	*Do you/people in your household usually have dinner at home on weekdays?	○ Yes<br>○ No (Go to Q1m)<br>○ Don't know (Go to Q1m)
1l	What time do you/people in your household usually have dinner at home on weekdays?<br>(24-hour clock: 7:00=7:00 a.m., 19:00=7:00 p.m.)	___:___
1m	*Do you/people in your household usually have dinner at home on Saturdays?	○ Yes<br>○ No (Go to Q1o)<br>○ Don't know (Go to Q1o)
1n	What time do you/people in your household usually have dinner at home on Saturdays?<br>(24-hour clock: 7:00=7:00 a.m., 19:00=7:00 p.m.)	___:___
1o	*Do you/people in your household usually have dinner at home on Sundays?	○ Yes<br>○ No (Go to Q2a)<br>○ Don't know (Go to Q2a)
1p	What time do you/people in your household usually have dinner at home on Sundays?<br>(24-hour clock: 7:00=7:00 a.m., 19:00=7:00 p.m.)	___:___

Please select the answer that best describes your household's activities of each over the last 2 weeks:

2a	Has your household used non-stick cookware?	○ Yes, every day<br>○ Yes, multiple times a week<br>○ Yes, once or twice a week<br>○ Yes, less than once a week<br>○ No, we don't use non-stick pans (Go to Q3)<br>○ Don't know (Go to Q3)
2b	*How old is your non-stick cookware?	○ Under 6 months<br>○ Between 6 months and 1 year<br>○ More than 1 year<br>○ Don't know
3a	*Have you/people in your household used hair sprays or deodorant sprays daily?	○ Yes<br>○ No (Go to Q4a)<br>○ Don't know (Go to Q4a)
3b	*How many people in your household have used hair sprays or deodorant sprays daily?	_____

For peer review only - http://bmjopen.bmj.com/site/about/guidelines.xhtml

4a *Which home fragrances does your household use or burn? (Tick all that apply)

- ☐ Candle
- ☐ Oud
- ☐ Uunsi/dabqaad/incense burner
- ☐ Incense sticks
- ☐ Reed diffusers
- ☐ Oil diffusers
- ☐ Air fresheners (including plug in)
- ☐ Other (write in) ____________________
- ☐ No (we don't use or burn home fragrances) (Go to Q5a)
- ☐ Don't know (Go to Q5a)

4c How many times a week, on average, does your household use or burn any of the following home fragrances? ______

Candle, oud, uunsi/dabqaad/incense burner incense sticks, reed diffusers, oil diffusers, air fresheners (including plug-in), other

		Every day	Multiple times a week	Once or twice a week	Less than once a week	Never	Don't know
5a	*How often does your household use spray surface/floor/kitchen/bathroom cleaners?	○	○	○	○	○	○
5b	*How often does your household use furniture or other spray polishes?	○	○	○	○	○	○
5c	*How often does your household use liquid surface/floor/kitchen/bathroom cleaners?	○	○	○	○	○	○
5d	*How often does your household use bleach?	○	○	○	○	○	○
6	*How often is your home vacuumed?	○	○	○	○	○	○
7a	*How often do you (or someone else in the household) dust your home?	○	○	○	○	○ (Go to Q8a)	○ (Go to Q8a)

7b *Which is the main method used for dusting?

- ○ Wet wipe
- ○ Dry wipe
- ○ Brushing off/using a feather duster
- ○ Using a microfibre duster
- ○ Hoover/vacuum cleaner
- ○ Don't know

8a *Do you do laundry (including washing and drying clothes) in your home?

- ○ Yes, washing machine only
- ○ Yes, washing machine and tumble dryer
- ○ Yes, other (write in) ____________________
- ○ No (Go to Q9a)
- ○ Don't know (Go to Q9a)

For peer review only

For peer review only - http://bmjopen.bmj.com/site/about/guidelines.xhtml

8c	*Which room do you use for washing your clothes in the home?	○ In the kitchen (with a sensor)<br>○ In the utility room<br>○ In the bathroom<br>○ Other (write in) ____________________<br>○ Don't know
8e	*Where/How do you dry your clothes?<br>(Tick all that apply)	☐ In the kitchen (with a sensor)<br>☐ In the living/dining room (with a sensor)<br>☐ In the bedroom (with a sensor)<br>☐ In the bedroom (without a sensor)<br>☐ In the bathroom<br>☐ Outside<br>☐ Using a tumble dryer<br>☐ Other (write in) ____________________<br>☐ Don't know
9a	*Does your household use a wood stove (solid fuel/wood/coal) or an open fire?	○ Yes<br>○ No (Go to Section III: Health, Q1a)
9b	*How many solid fuel appliances do you use?	_____

Please answer the following questions for each appliance separately.

If you use more than 3 solid fuel appliances, please answer for the most-commonly-used 3 appliances (i.e. Appliances 1, 2 and 3).

9c	*What type of solid fuel appliance is *Appliance 1*?	○ Wood or coal burning stoves (solid fuel/wood/coal)<br>○ Open fires (Go to Q9g)<br>○ Biomass boiler (Go to Q9g)<br>○ Other (write in) ____________________ (Go to Q9g)
9e	What make or model (if known) of stove is *Appliance 1*?	____________________
9f	*Is *Appliance 1* *DEFRA*-approved and/or Eco Design Ready?<br>*Department for Environment, Food and Rural Affairs*	○ Yes<br>○ No<br>○ Don't know
9g	*Did you purchase or install *Appliance 1*, or was it there when you moved in?	○ I purchased/installed this appliance<br>○ The appliance was there when I moved in (Go to Q9j)<br>○ Don't know (Go to Q9j)
9h	*What considerations did you have in mind when deciding which wood or coal burning stove to purchase?<br>(Tick all that apply)	☐ Emission rates<br>☐ Environmental impact<br>☐ Best suited for our home and/or needs<br>☐ Price<br>☐ Recommendation<br>☐ The look of the stove<br>☐ Other (write in) ____________________

For peer review only - http://bmjopen.bmj.com/site/about/guidelines.xhtml

		☐ Don't know
9j	*Did you get any training on using *Appliance 1* when it was installed or when you moved in?	○ Yes<br>○ No, I didn't get given any training or information (Go to Q9m)<br>○ No, I didn't get given any training or information, but I sought information/advice independently (Go to Q9m)<br>○ Don't know (Go to Q9m)
9k	*What training on using *Appliance 1* did you get?<br>(Tick all that apply)	☐ Written instructions<br>☐ Video instructions (e.g. an online guidance video)<br>☐ Someone demonstrated in person<br>☐ Other (write in) ______________________
9m	*When was your chimney or flue for *Appliance 1* last cleaned or maintained?<br>(Tick all that apply)	☐ Professionally cleaned in the last 6 months<br>☐ Professionally cleaned in the last 12 months<br>☐ Cleaned or maintained by someone in the household in the last 6 months<br>☐ Cleaned or maintained by someone in the household in the last 12 months<br>☐ Has not been cleaned or maintained in more than a year<br>☐ Not relevant to this solid fuel appliance<br>☐ Don't know
9n	*What type of fuel do you use in *Appliance 1*?<br>(Tick all that apply)	☐ House coal<br>☐ Smokeless coal<br>☐ Other type of coal<br>☐ Unseasoned wood<br>☐ Seasoned wood<br>☐ Waste wood (e.g. old furniture, pallets or fence panels)<br>☐ Rubbish<br>☐ Manufactured log briquettes<br>☐ Peat<br>☐ Wood pellets<br>☐ Other (write in) ______________________<br>☐ Don't know
9p	*What type of solid fuel appliance is *Appliance 2*?	○ Wood or coal burning stoves (solid fuel/wood/coal)<br>○ Open fires (Go to Q9t)<br>○ Biomass boiler (Go to Q9t)<br>○ Other (write in) ______________________ (Go to Q9t)
9r	What make or model (if known) of stove is *Appliance 2*?	______________________
9s	*Is *Appliance 2* *DEFRA*-approved and/or Eco Design Ready?<br>*Department for Environment, Food and Rural Affairs*	○ Yes<br>○ No<br>○ Don't know

For peer review only - http://bmjopen.bmj.com/site/about/guidelines.xhtml

9t	*Did you purchase or install *Appliance 2*, or was it there when you moved in?	○ I purchased/installed this appliance<br>○ The appliance was there when I moved in (Go to Q9w)<br>○ Don't know (Go to Q9w)
9u	*What considerations did you have in mind when deciding which wood or coal burning stove to purchase?<br>(Tick all that apply)	☐ Emission rates<br>☐ Environmental impact<br>☐ Best suited for our home and/or needs<br>☐ Price<br>☐ Recommendation<br>☐ The look of the stove<br>☐ Other (write in) ____________________<br>☐ Don't know
9w	*Did you get any training on using *Appliance 2* when it was installed or when you moved in?	○ Yes<br>○ No, I didn't get given any training or information (Go to Q9z)<br>○ No, I didn't get given any training or information, but I sought information/advice independently (Go to Q9z)<br>○ Don't know (Go to Q9z)
9x	*What training on using *Appliance 2* did you get?<br>(Tick all that apply)	☐ Written instructions<br>☐ Video instructions (e.g. an online guidance video)<br>☐ Someone demonstrated in person<br>☐ Other (write in) ____________________
9z	*When was your chimney or flue for *Appliance 2* last cleaned or maintained?<br>(Tick all that apply)	☐ Professionally cleaned in the last 6 months<br>☐ Professionally cleaned in the last 12 months<br>☐ Cleaned or maintained by someone in the household in the last 6 months<br>☐ Cleaned or maintained by someone in the household in the last 12 months<br>☐ Has not been cleaned or maintained in more than a year<br>☐ Not relevant to this solid fuel appliance<br>☐ Don't know
9aa	*What type of fuel do you use in *Appliance 2*?<br>(Tick all that apply)	☐ House coal<br>☐ Smokeless coal<br>☐ Other type of coal<br>☐ Unseasoned wood<br>☐ Seasoned wood<br>☐ Waste wood (e.g. old furniture, pallets or fence panels)<br>☐ Rubbish<br>☐ Manufactured log briquettes<br>☐ Peat<br>☐ Wood pellets

For peer review only

For peer review only - http://bmjopen.bmj.com/site/about/guidelines.xhtml

		☐ Other (write in) ____________________<br>☐ Don't know
9ac	*What type of solid fuel appliance is *Appliance 3*?	○ Wood or coal burning stoves (solid fuel/wood/coal)<br>○ Open fires (Go to Q9ag)<br>○ Biomass boiler (Go to Q9ag)<br>○ Other (write in) ____________________ (Go to Q9ag)
9ae	What make or model (if known) of stove is *Appliance 3*?	____________________
9af	*Is *Appliance 3* *DEFRA*-approved and/or Eco Design Ready?<br>*Department for Environment, Food and Rural Affairs*	○ Yes<br>○ No<br>○ Don't know
9ag	*Did you purchase or install *Appliance 3*, or was it there when you moved in?	○ I purchased/installed this appliance<br>○ The appliance was there when I moved in (Go to Q9aj)<br>○ Don't know (Go to Q9aj)
9ah	*What considerations did you have in mind when deciding which wood or coal burning stove to purchase?<br>(Tick all that apply)	☐ Emission rates<br>☐ Environmental impact<br>☐ Best suited for our home and/or needs<br>☐ Price<br>☐ Recommendation<br>☐ The look of the stove<br>☐ Other (write in) ____________________<br>☐ Don't know
9aj	*Did you get any training on using *Appliance 3* when it was installed or when you moved in?	○ Yes<br>○ No, I didn't get given any training or information (Go to Q9am)<br>○ No, I didn't get given any training or information, but I sought information/advice independently (Go to Q9am)<br>○ Don't know (Go to Q9am)
9ak	*What training on using *Appliance 3* did you get?<br>(Tick all that apply)	☐ Written instructions<br>☐ Video instructions (e.g. an online guidance video)<br>☐ Someone demonstrated in person<br>☐ Other (write in) ____________________
9am	*When was your chimney or flue for *Appliance 3* last cleaned or maintained?<br>(Tick all that apply)	☐ Professionally cleaned in the last 6 months<br>☐ Professionally cleaned in the last 12 months<br>☐ Cleaned or maintained by someone in the household in the last 6 months<br>☐ Cleaned or maintained by someone in the household in the last 12 months<br>☐ Has not been cleaned or maintained in more than a year<br>☐ Not relevant to this solid fuel appliance

For peer review only - http://bmjopen.bmj.com/site/about/guidelines.xhtml

		☐ Don't know
9an	*What type of fuel do you use in *Appliance 3*? (Tick all that apply)	☐ House coal<br>☐ Smokeless coal<br>☐ Other type of coal<br>☐ Unseasoned wood<br>☐ Seasoned wood<br>☐ Waste wood (e.g. old furniture, pallets or fence panels)<br>☐ Rubbish<br>☐ Manufactured log briquettes<br>☐ Peat<br>☐ Wood pellets<br>☐ Other (write in) ____________________<br>☐ Don't know
9ap	*Are you aware of government regulations regarding burning authorised fuels and exempt appliances?	○ Yes, and I'm familiar with the content of the regulations<br>○ Yes, but I'm not familiar with the content of the regulations<br>○ No, but I know what an exempt appliance is<br>○ No, but I know what authorised fuels are<br>○ No, and I don't know what either authorised fuels or exempt appliances are
9aq	*Do you live in a smoke control area?	○ Yes<br>○ No<br>○ I know what a smoke control area is, but I don't know if I live in one<br>○ I don't know what a smoke control area is
9ar	*Are you aware of government regulations about smoke coming from chimneys that are not in smoke control areas?	○ Yes<br>○ No, but I know what a smoke control area is<br>○ No, and I don't know what a smoke control area is

Section III: Health

This section is about your/your BiB child's health and wellbeing.

Please give your answers on YOUR BiB CHILD's health.

0a	How many BiB children do you have?<br><br>If you have <u>more than one BiB child</u>, but would like to answer ONLY one of your BiB children's health & wellbeing questions, please type '1'.	_____<br><br>If you type <u>more than ONE</u>, please ask a BiB researcher for another set of health & wellbeing questions for your other BiB children.
0b	What is your BiB child's date of birth?	___/___/_____ (dd/mm/yyyy)

For peer review only - http://bmjopen.bmj.com/site/about/guidelines.xhtml

1a	*Has your BiB child ever had wheezing or whistling in the chest at any time in the past?	○ Yes<br>○ No (Go to Q1f)<br>○ Don't know (Go to Q1f)
1b	*Has your BiB child had wheezing or whistling in the chest in the past 2 weeks?	○ Yes<br>○ No (Go to Q1f)<br>○ Don't know (Go to Q1f)
1c	*How many attacks of wheezing has your BiB child had in the past 2 weeks?	○ None<br>○ 1<br>○ 2<br>○ 3<br>○ 4<br>○ More than 4<br>○ Don't know
1d	*In the past 2 weeks, how often, on average, has your BiB child's sleep been disturbed due to wheezing?	○ Never woken with wheezing<br>○ Less than one night per week<br>○ One or more nights per week<br>○ Don't know
1e	*In the past 2 weeks, has wheezing ever been severe enough to limit your BiB child's speech to only one or two words at a time between breaths?	○ Yes<br>○ No<br>○ Don't know
1f	*Has your BiB child ever had asthma?	○ Yes<br>○ No (Go to Q1h)<br>○ Don't know (Go to Q1h)
1g	*Has your BiB child ever been diagnosed by a doctor as having asthma?	○ Yes<br>○ No<br>○ Don't know
1h	*In the past 2 weeks, has your BiB child's chest sounded wheezy during or after exercise?	○ Yes<br>○ No<br>○ Don't know
1i	*In the past 2 weeks, has your BiB child had a dry cough at night, apart from a cough associated with a cold or chest infection?	○ Yes<br>○ No<br>○ Don't know
1j	*Has your BiB child taken any medicines for asthma or breathing difficulties (chest tightness, shortness of breath) in the past 2 weeks?	○ Yes<br>○ No<br>○ Don't know
2a	*Has your BiB child ever had a problem with sneezing, or a running, or blocked nose when he/she/they DID NOT have a cold or the flu?	○ Yes<br>○ No (Go to Q2f)<br>○ Don't know (Go to Q2f)

For peer review only - http://bmjopen.bmj.com/site/about/guidelines.xhtml

2b	*In the past 2 weeks, has your BiB child had a problem with sneezing, or a running, or blocked nose when he/she/they DID NOT have a cold or the flu?	○ Yes<br>○ No (Go to Q2f)<br>○ Don't know (Go to Q2f)
2c	*In the past 2 weeks, has this nose problem been accompanied by itchy-watery eyes?	○ Yes<br>○ No<br>○ Don't know
2d	*In the past 2 weeks, how many days did this nose problem occur?	______
2e	*In the past 2 weeks, how much did this nose problem interfere with your BiB child's daily activities?	○ Not at all<br>○ A little<br>○ A moderate amount<br>○ A lot<br>○ Don't know
2f	*Has your BiB child ever had hayfever?	○ Yes<br>○ No<br>○ Don't know
3a	*Has your BiB child ever had an itchy rash which was coming and going for at least six months?	○ Yes<br>○ No (Go to Q3g)<br>○ Don't know (Go to Q3g)
3b	*Has your BiB child had this itchy rash at any time in the past 2 weeks?	○ Yes<br>○ No (Go to Q3g)<br>○ Don't know (Go to Q3g)
3c	*Has this itchy rash at any time affected any of the following places?<br><br>*The folds of the elbows, behind the knees, in front of the ankles, under the buttocks, or around the neck, ears or eyes*	○ Yes<br>○ No<br>○ Don't know
3d	*At what age did this itchy rash first occur?	○ Under 2 years<br>○ Age 2-4 years<br>○ Age 5 or more<br>○ Don't know
3e	*Has this rash cleared completely at any time during the past 2 weeks?	○ Yes<br>○ No<br>○ Don't know
3f	*In the past 2 weeks, how often, on average, have your BiB child been kept awake at night by this itchy rash?	○ Never in the past 2 weeks<br>○ Less than one night per week<br>○ One or more nights per week<br>○ Don't know
3g	*Has your BiB child ever had eczema?	○ Yes

For peer review only

For peer review only - http://bmjopen.bmj.com/site/about/guidelines.xhtml

○ No
○ Don't know

Please give your answers on the basis of YOUR BiB CHILD's behaviour over the last 2 weeks. It would help us if you answered all items as best you can even if you are not absolutely certain.

		Not true	Somewhat true	Certainly true
4a	*Considerate of other people's feelings	○	○	○
4b	*Restless, overactive, cannot stay still for long	○	○	○
4c	*Often complains of headaches, stomach-aches or sickness	○	○	○
4d	*Shares readily with other children (treats, toys, pencils etc.)	○	○	○
4e	*Often has temper tantrums or hot tempers	○	○	○
4f	*Rather solitary, tends to play alone	○	○	○
4g	*Generally obedient, usually does what adults request	○	○	○
4h	*Many worries, often seems worried	○	○	○
4i	*Helpful if someone is hurt, upset or feeling ill	○	○	○
4j	*Constantly fidgeting or squirming	○	○	○
4k	*Has at least one good friend	○	○	○
4l	*Often fights with other children or bullies them	○	○	○
4m	*Often unhappy, down-hearted or tearful	○	○	○
4n	*Generally liked by other children	○	○	○
4o	*Easily distracted, concentration wanders	○	○	○
4p	*Nervous or clingy in new situations, easily loses confidence	○	○	○
4q	*Kind to younger children	○	○	○
4r	*Often lies or cheats	○	○	○
4s	*Picked on or bullied by other children	○	○	○
4t	*Often volunteers to help others (parents, teachers, other children)	○	○	○
4u	*Thinks things out before acting	○	○	○
4v	*Steals from home, school or elsewhere	○	○	○
4w	*Gets on better with adults than with other children	○	○	○
4x	*Many fears, easily scared	○	○	○
4y	*Sees tasks through to the end, good attention span	○	○	○

4z *Overall, do you think that your BiB child has difficulties in one or more of the following areas:
○ No (Go to Q5a)
○ Yes – minor difficulties

For peer review only

For peer review only - http://bmjopen.bmj.com/site/about/guidelines.xhtml

	Emotions, concentration, behaviour or being able to get on with other people?	○ Yes – definite difficulties<br>○ Yes – severe difficulties
4aa	*How long have these difficulties been present?	○ Less than a month<br>○ 1-5 months<br>○ 6-12 months<br>○ Over a year
4ab	*Do the difficulties upset or distress your BiB child?	○ Not at all<br>○ Only a little<br>○ Quite a lot<br>○ A great deal

	Do the difficulties interfere with your BiB child's everyday life in the following areas?	Not at all	Only a little	Quite a lot	A great deal
4ac	*Home life	○	○	○	○
4ad	*Friendships	○	○	○	○
4ae	*Classroom learning	○	○	○	○
4af	*Leisure activities	○	○	○	○

4ag	*Do the difficulties put a burden on you or the family as a whole?	○ Not at all<br>○ Only a little<br>○ Quite a lot<br>○ A great deal

Please refer to YOUR health (and not to the health of your BiB child).

5a	*Do you ever have trouble with your breathing?	○ Never<br>○ Only rarely<br>○ Repeatedly, but it always gets completely better<br>○ Continuously, so that your breathing is never quite right
5b	*Have you had wheezing or whistling in your chest at any time in the past 2 weeks?	○ Yes<br>○ No (Go to Q5i)
5c	*How many attacks of wheezing have you had in the past 2 weeks?	○ None<br>○ 1<br>○ 2<br>○ 3<br>○ 4<br>○ More than 4
5d	*In the past 2 weeks, how often, on average, has your sleep been disturbed due to wheezing?	○ Never woken with wheezing<br>○ Less than one night per week

For peer review only

		○ One or more nights per week
5e	*Have you ever been breathless when the wheezing noise was present?	○ Yes<br>○ No
5f	*In the past 2 weeks, how often, on average, has your sleep been disturbed due to shortness of breath?	○ Never<br>○ Less than one night per week<br>○ One or more nights per week
5g	*In the past 2 weeks, how often, on average, has your sleep been disturbed due to coughing?	○ Never<br>○ Less than one night per week<br>○ One or more nights per week
5h	*In the past 2 weeks, has wheezing ever been severe enough to limit your speech to only one or two words at a time between breaths?	○ Yes<br>○ No
5i	*Have you ever had asthma?	○ Yes<br>○ No (Go to Q5o)
5j	*Was your asthma confirmed by a doctor?	○ Yes<br>○ No
5k	*How old were you when you had your first attack of asthma?<br><br>*If you have never had an asthma attack, please type '88'.*<br>*If you don't know the answer, please type '99'.*	_____
5l	*Have you had an asthma attack in the past 2 weeks?	○ Yes<br>○ No
5m	*Have you used any inhaled medicines (e.g. salbutamol) to help your breathing at any time in the past 2 weeks (when you DID NOT have a cold)?	○ Yes<br>○ No
5n	*In the past 2 weeks, how many days was your usual activity (at work or in the home) limited because you had breathing problems?	_____
5o	*Have you ever had hay fever?	○ Yes<br>○ No (Go to Q5q)
5p	*Was your hay fever confirmed by a doctor?	○ Yes<br>○ No
5q	*Have you ever had eczema?	○ Yes<br>○ No (Go to Q6a)
5r	*Was your eczema confirmed by a doctor?	○ Yes<br>○ No

Please tick the box that best describe YOUR experience of each over the last 2 weeks:

For peer review only

		None of the time	Rarely	Some of the time	Often	All of the time
6a	*I've been feeling optimistic about the future.	○	○	○	○	○
6b	*I've been feeling useful.	○	○	○	○	○
6c	*I've been feeling relaxed.	○	○	○	○	○
6d	*I've been dealing with problems well.	○	○	○	○	○
6e	*I've been thinking clearly.	○	○	○	○	○
6f	*I've been feeling close to other people.	○	○	○	○	○
6g	*I've been able to make up my own mind about things.	○	○	○	○	○

How often have YOU been bothered by any of the following problems over the last 2 weeks?

		Not at all	Several days	More than half the days	Nearly every day
7a	*Little interest or pleasure in doing things	○	○	○	○
7b	*Feeling down, depressed, or hopeless	○	○	○	○
7c	*Trouble falling or staying asleep, or sleeping too much	○	○	○	○
7d	*Feeling tired or having little energy	○	○	○	○
7e	*Poor appetite or overeating	○	○	○	○
7f	*Feeling bad about yourself – or that you are a failure or have let yourself or your family down	○	○	○	○
7g	*Trouble concentrating on things, such as reading the newspaper or watching television	○	○	○	○
7h	*Moving or speaking so slowly that other people could have noticed? Or the opposite – being so fidgety or restless that you have been moving around a lot more than usual	○	○	○	○

Section IV: About You and Your Household

This section is about your socio-economic circumstances.

1a	*How many people including yourself are there in your household?	_____
1b	*How many children (aged under 18) are there in your household?	_____

Please read each statement below and tell us whether the statement was *often true*, *sometimes true*, or *never true* for you or anyone in your household in the last 12 months:

For peer review only - http://bmjopen.bmj.com/site/about/guidelines.xhtml

		Often true	Sometimes true	Never true	Don't know	Do not wish to answer
2a	*The food that I/we bought just didn't last, and I/we didn't have money to get more.	○	○	○	○	○
2b	*I/we couldn't afford to eat balanced meals.	○	○	○	○	○

2c	*In the last 12 months, did you or other adults in your household ever cut the size of your meals or skip meals because there wasn't enough money for food?	○ Yes<br>○ No (Go to Q2e)<br>○ Don't know (Go to Q2e)<br>○ Do not wish to answer (Go to Q2e)
2d	*How often did you or other adults cut the size of your meals or skip meals?	○ Almost every month<br>○ Some months but not every month<br>○ Only 1 or 2 months<br>○ Don't know<br>○ Do not wish to answer
2e	*In the last 12 months, did you or other adults ever eat less than you felt you should because there wasn't enough money for food?	○ Yes<br>○ No<br>○ Don't know<br>○ Do not wish to answer
2f	*In the last 12 months, were you ever hungry but didn't eat because there wasn't enough money for food?	○ Yes<br>○ No<br>○ Don't know<br>○ Do not wish to answer
3	*When you are at home on a typical day in winter, are you (and everyone in your household) warm enough?	○ Yes, always<br>○ Yes, sometimes<br>○ No, rarely<br>○ No, never<br>○ Don't know<br>○ Do not wish to answer
4a	*Which of the following is the main way you keep the inside of your house cool in warm weather?<br>(Tick all that apply)	☐ Natural ventilation or shade (e.g. open windows or doors, using heat absorbing shades)<br>☐ Open outside doors<br>☐ Electronic devices (e.g. fans or air conditioning)<br>☐ Other (write in) ____________________<br>☐ Don't know
5	*How do you pay for your electricity/gas? (Tick only one)	○ Pre-payment meter<br>○ Regular bill<br>○ Direct Debit<br>○ Included in rent

For peer review only

For peer review only - http://bmjopen.bmj.com/site/about/guidelines.xhtml

		○ Don't know
6a	*How well would you say your household is managing financially these days?<br><br>Would you say you are:	○ Living comfortably<br>○ Doing alright<br>○ Just about getting by<br>○ Finding it quite difficult<br>○ Finding it very difficult<br>○ Do not wish to answer
6b	*Compared to a year ago, how would you say your household is doing financially now?	○ Better off<br>○ Worse off<br>○ About the same<br>○ Do not wish to answer
6c	*How often would you say you have been worried about money during the last few weeks?	○ Almost all the time<br>○ Quite often<br>○ Only sometimes<br>○ Never<br>○ Do not wish to answer
7	Please tell us anything else you would like to comment on in relation to the study.	

Thank you for participation.

If you have any questions, please contact us: xxxxx xxxxxx or borninbradford@bthft.nhs.uk

For peer review only

For peer review only - http://bmjopen.bmj.com/site/about/guidelines.xhtml

Supplementary File 2. Diary

Participant ID: ING____________

Health (Week 1)

What is your age? ____ years old **What is your gender?** ☐ Female ☐ Male ☐ Non-binary ☐ Do not wish to answer ☐ Wish to enter own response (____________)

**Please circle one for the following symptoms you had today.** dd/mm/yyyy (ddd) e.g. 09/03/2023 (Thu)	**Day 1** / / ( )	**Day 2** / / ( )	**Day 3** / / ( )	**Day 4** / / ( )	**Day 5** / / ( )	**Day 6** / / ( )	**Day 7** / / ( )
1. I was at home today.	YES | NO	YES | NO	YES | NO	YES | NO	YES | NO	YES | NO	YES | NO
If YES, what time? (24 hour clock)	: – :<br>: – :<br>: – :	: – :<br>: – :<br>: – :	: – :<br>: – :<br>: – :	: – :<br>: – :<br>: – :	: – :<br>: – :<br>: – :	: – :<br>: – :<br>: – :	: – :<br>: – :<br>: – :
2. I had breathing difficulties.	YES | NO	YES | NO	YES | NO	YES | NO	YES | NO	YES | NO	YES | NO
If YES, what time? (24 hour clock)	: – :	: – :	: – :	: – :	: – :	: – :	: – :
3. I had wheezing in the chest.	YES | NO	YES | NO	YES | NO	YES | NO	YES | NO	YES | NO	YES | NO
If YES, what time? (24 hour clock)	: – :	: – :	: – :	: – :	: – :	: – :	: – :
4. I had shortness of breath.	YES | NO	YES | NO	YES | NO	YES | NO	YES | NO	YES | NO	YES | NO
If YES, what time? (24 hour clock)	: – :	: – :	: – :	: – :	: – :	: – :	: – :
5. I had chest tightness.	YES | NO	YES | NO	YES | NO	YES | NO	YES | NO	YES | NO	YES | NO
If YES, what time? (24 hour clock)	: – :	: – :	: – :	: – :	: – :	: – :	: – :
6. I had chest pain.	YES | NO	YES | NO	YES | NO	YES | NO	YES | NO	YES | NO	YES | NO
If YES, what time? (24 hour clock)	: – :	: – :	: – :	: – :	: – :	: – :	: – :
7. I had a dry cough when I did not have a cold or the flu.	YES | NO	YES | NO	YES | NO	YES | NO	YES | NO	YES | NO	YES | NO
If YES, what time? (24 hour clock)	: – :	: – :	: – :	: – :	: – :	: – :	: – :
8. I had a problem with sneezing when I did not have a cold or the flu.	YES | NO	YES | NO	YES | NO	YES | NO	YES | NO	YES | NO	YES | NO
If YES, what time? (24 hour clock)	: – :	: – :	: – :	: – :	: – :	: – :	: – :
9. I had a runny nose when I did not have a cold or the flu.	YES | NO	YES | NO	YES | NO	YES | NO	YES | NO	YES | NO	YES | NO
If YES, what time? (24 hour clock)	: – :	: – :	: – :	: – :	: – :	: – :	: – :
10. I had a blocked nose when I did not have a cold or the flu.	YES | NO	YES | NO	YES | NO	YES | NO	YES | NO	YES | NO	YES | NO
If YES, what time? (24 hour clock)	: – :	: – :	: – :	: – :	: – :	: – :	: – :
11. I had itchy-watery eyes when I did not have a cold or the flu.	YES | NO	YES | NO	YES | NO	YES | NO	YES | NO	YES | NO	YES | NO
If YES, what time? (24 hour clock)	: – :	: – :	: – :	: – :	: – :	: – :	: – :
12. I had an itchy rash.	YES | NO	YES | NO	YES | NO	YES | NO	YES | NO	YES | NO	YES | NO
If YES, what time? (24 hour clock)	: – :	: – :	: – :	: – :	: – :	: – :	: – :
13. I had dry skin.	YES | NO	YES | NO	YES | NO	YES | NO	YES | NO	YES | NO	YES | NO
If YES, what time? (24 hour clock)	: – :	: – :	: – :	: – :	: – :	: – :	: – :
**At the moment I feel: (0='sad' ☹ – 10='happy' ☺)**	___ (0-10)	___ (0-10)	___ (0-10)	___ (0-10)	___ (0-10)	___ (0-10)	___ (0-10)

Note:

For peer review only - http://bmjopen.bmj.com/site/about/guidelines.xhtml

Participant ID: ING____________

Page 2

Health (Week 2)

<table>
<tr><th>Please circle one for the following symptoms you had today.
dd/mm/yyyy (ddd) e.g. 09/03/2023 (Thu)</th><th>Day 8
/ /
( )</th><th>Day 9
/ /
( )</th><th>Day 10
/ /
( )</th><th>Day 11
/ /
( )</th><th>Day 12
/ /
( )</th><th>Day 13
/ /
( )</th><th>Day 14
/ /
( )</th></tr>
<tr><td>1. I was at home today.
If YES, what time? (24 hour clock)</td><td>YES | NO
: – :
: – :
: – :</td><td>YES | NO
: – :
: – :
: – :</td><td>YES | NO
: – :
: – :
: – :</td><td>YES | NO
: – :
: – :
: – :</td><td>YES | NO
: – :
: – :
: – :</td><td>YES | NO
: – :
: – :
: – :</td><td>YES | NO
: – :
: – :
: – :</td></tr>
<tr><td>2. I had breathing difficulties.
If YES, what time? (24 hour clock)</td><td>YES | NO
: – :</td><td>YES | NO
: – :</td><td>YES | NO
: – :</td><td>YES | NO
: – :</td><td>YES | NO
: – :</td><td>YES | NO
: – :</td><td>YES | NO
: – :</td></tr>
<tr><td>3. I had wheezing in the chest.
If YES, what time? (24 hour clock)</td><td>YES | NO
: – :</td><td>YES | NO
: – :</td><td>YES | NO
: – :</td><td>YES | NO
: – :</td><td>YES | NO
: – :</td><td>YES | NO
: – :</td><td>YES | NO
: – :</td></tr>
<tr><td>4. I had shortness of breath.
If YES, what time? (24 hour clock)</td><td>YES | NO
: – :</td><td>YES | NO
: – :</td><td>YES | NO
: – :</td><td>YES | NO
: – :</td><td>YES | NO
: – :</td><td>YES | NO
: – :</td><td>YES | NO
: – :</td></tr>
<tr><td>5. I had chest tightness.
If YES, what time? (24 hour clock)</td><td>YES | NO
: – :</td><td>YES | NO
: – :</td><td>YES | NO
: – :</td><td>YES | NO
: – :</td><td>YES | NO
: – :</td><td>YES | NO
: – :</td><td>YES | NO
: – :</td></tr>
<tr><td>6. I had chest pain.
If YES, what time? (24 hour clock)</td><td>YES | NO
: – :</td><td>YES | NO
: – :</td><td>YES | NO
: – :</td><td>YES | NO
: – :</td><td>YES | NO
: – :</td><td>YES | NO
: – :</td><td>YES | NO
: – :</td></tr>
<tr><td>7. I had a dry cough when I did not have a cold or the flu.
If YES, what time? (24 hour clock)</td><td>YES | NO
: – :</td><td>YES | NO
: – :</td><td>YES | NO
: – :</td><td>YES | NO
: – :</td><td>YES | NO
: – :</td><td>YES | NO
: – :</td><td>YES | NO
: – :</td></tr>
<tr><td>8. I had a problem with sneezing when I did not have a cold or the flu.
If YES, what time? (24 hour clock)</td><td>YES | NO
: – :</td><td>YES | NO
: – :</td><td>YES | NO
: – :</td><td>YES | NO
: – :</td><td>YES | NO
: – :</td><td>YES | NO
: – :</td><td>YES | NO
: – :</td></tr>
<tr><td>9. I had a runny nose when I did not have a cold or the flu.
If YES, what time? (24 hour clock)</td><td>YES | NO
: – :</td><td>YES | NO
: – :</td><td>YES | NO
: – :</td><td>YES | NO
: – :</td><td>YES | NO
: – :</td><td>YES | NO
: – :</td><td>YES | NO
: – :</td></tr>
<tr><td>10. I had a blocked nose when I did not have a cold or the flu.
If YES, what time? (24 hour clock)</td><td>YES | NO
: – :</td><td>YES | NO
: – :</td><td>YES | NO
: – :</td><td>YES | NO
: – :</td><td>YES | NO
: – :</td><td>YES | NO
: – :</td><td>YES | NO
: – :</td></tr>
<tr><td>11. I had itchy-watery eyes when I did not have a cold or the flu.
If YES, what time? (24 hour clock)</td><td>YES | NO
: – :</td><td>YES | NO
: – :</td><td>YES | NO
: – :</td><td>YES | NO
: – :</td><td>YES | NO
: – :</td><td>YES | NO
: – :</td><td>YES | NO
: – :</td></tr>
<tr><td>12. I had an itchy rash.
If YES, what time? (24 hour clock)</td><td>YES | NO
: – :</td><td>YES | NO
: – :</td><td>YES | NO
: – :</td><td>YES | NO
: – :</td><td>YES | NO
: – :</td><td>YES | NO
: – :</td><td>YES | NO
: – :</td></tr>
<tr><td>13. I had dry skin.
If YES, what time? (24 hour clock)</td><td>YES | NO
: – :</td><td>YES | NO
: – :</td><td>YES | NO
: – :</td><td>YES | NO
: – :</td><td>YES | NO
: – :</td><td>YES | NO
: – :</td><td>YES | NO
: – :</td></tr>
<tr><td>At the moment I feel: (0='sad' ☹ – 10='happy' ☺)</td><td>___ (0-10)</td><td>___ (0-10)</td><td>___ (0-10)</td><td>___ (0-10)</td><td>___ (0-10)</td><td>___ (0-10)</td><td>___ (0-10)</td></tr>
</table>

Note:

INGENIOUS_Diary (V2.0; 17/03/2023)

DATE: (please circle one) **Mon** / **Tue** / **Wed** / **Thu** / **Fri** , (please write in) ____/____/______ (dd/mm/yyyy) Participant ID: ING____________ Page 1

PLEASE FILL IN IF APPLICABLE

COOKING

What time did you or someone else in your household do the following activities in your home? (24 hour clock)

Frying	Boiling | Steaming	Baking | Grilling Roasting	Using an extractor hood | fan
: – :	: – :	: – :	: – :
: – :	: – :	: – :	: – :
: – :	: – :	: – :	: – :

CLEANING

What time did you or someone else in your household do the following activities in your home? (24 hour clock)

Vacuuming | Dusting	Using cleaning sprays | furniture polishes
: – :	: – :
: – :	: – :
: – :	: – :

PRODUCTS

What time did you or someone else in your household use the following products in your home? (24 hour clock)

Air freshener sprays Plug-in air fresheners Insecticides | Fly sprays	Candle | Incense | Other fragrance burning sources
: – :	: – :
: – :	: – :
: – :	: – :

HEATING

Were the following heating sources on in your home? (Please circle one each)

Central heating	Central boiler
YES | NO	YES | NO

(YES for timer or temperature control is set)

What time did you or someone else in your household use stoves (solid fuel | wood | coal) or open fires? (24 hour clock)

: – :	: – :
: – :	: – :

VENTILATION

What time did you or someone else in your household open the windows or the doors (leading to outside) in the following rooms (*where a sensor is placed)? (24 hour clock)

Living room*	Kitchen*	Bedroom*	Main bathroom	Use an extractor fan
: – :	: – :	: – :	: – :	: – :
: – :	: – :	: – :	: – :	: – :
: – :	: – :	: – :	: – :	: – :

PERSONAL CARE

What time did you or someone else in your household do the following activities in your home? (24 hour clock)

Taking a shower | bath		Using deodorant | aftershave | perfume | hairspray		Smoking | Using e-cigarettes (in | outside of your house)	
: – :	: – :	: – :	: – :	: – :	: – :
: – :	: – :	: – :	: – :	: – :	: – :

OCCUPANCY

What time were there people at home? How many people?

: – :	people	: – :	people
: – :	people	: – :	people
: – :	people		

INGENIOUS_Diary (V2.0; 17/03/2023)

INGENIOUS

We are family

DATE: (please circle one) **Mon** / **Tue** / **Wed** / **Thu** / **Fri** , (please write in) ____/____/______ (dd/mm/yyyy) Participant ID: ING___________ Page 2

NOTES:

COOKING

E.g. Lots of smoke from a toaster; Left a frying pan on too long and produced lots of smokes

CLEANING

E.g. Used cleaning sprays on Sun morning (if not recorded in the diary); Used eco friendly or natural cleaning products

PRODUCTS

E.g. Used air fresheners in the living room; Burnt candles in the kitchen

HEATING

E.g. Used portable radiant/electric heaters in the living room & child's bedrooms in the night

PERSONAL CARE

E.g. Smelled smoking/e-cigarettes from neighbours; Used deodorant/perfume in the main bathroom

VENTILATION

E.g. Opened patio and bifold doors to get fresh air; Used an air purifier in the living room; Used an dehumidifier in the bathroom and child's bedroom

OTHER EXPERIENCES RELATED TO AIR QUALITY

E.g. Smelled my neighbour's garden fire from inside of the house; Had drain smells in the bathroom; Smelled something (don't know what it is) from the outside

OCCUPANCY

E.g. No one was at home on Sat; My dog and cat stayed inside of the house

For peer review only - http://bmjopen.bmj.com/site/about/guidelines.xhtml

INGENIOUS

We are family

Weekend day

DATE: (please circle one) **Sat** / **Sun**, (please write in) ____/____/______ (dd/mm/yyyy)

Participant ID: ING____________

COOKING

What time did you or someone else in your household do the following activities in your home? (24 hour clock)

Frying	Boiling | Steaming	Baking | Grilling Roasting	Using an extractor hood | fan
: — :	: — :	: — :	: — :
: — :	: — :	: — :	: — :
: — :	: — :	: — :	: — :

CLEANING

What time did you or someone else in your household do the following activities in your home? (24 hour clock)

Vacuuming | Dusting	Using cleaning sprays | furniture polishes
: — :	: — :
: — :	: — :
: — :	: — :

PRODUCTS

What time did you or someone else in your household use the following products in your home? (24 hour clock)

Air freshener sprays Plug-in air fresheners Insecticides | Fly sprays	Candle | Incense | Other fragrance burning sources
: — :	: — :
: — :	: — :
: — :	: — :

HEATING

Were the following heating sources on in your home? (Please circle one each)

Central heating	Central boiler
YES | NO	YES | NO

(YES for timer or temperature control is set)

What time did you or someone else in your household use stoves (solid fuel | wood | coal) or open fires? (24 hour clock)

: — :	: — :
: — :	: — :

VENTILATION

What time did you or someone else in your household open the windows or the doors (leading to outside) in the following rooms (*where a sensor is placed)? (24 hour clock)

Living room*	Kitchen*	Bedroom*	Main bathroom	Use an extractor fan
: — :	: — :	: — :	: — :	: — :
: — :	: — :	: — :	: — :	: — :
: — :	: — :	: — :	: — :	: — :

PERSONAL CARE

What time did you or someone else in your household do the following activities in your home? (24 hour clock)

Taking a shower | bath		Using deodorant | aftershave | perfume | hairspray		Smoking | Using e-cigarettes (in | outside of your house)	
: — :	: — :	: — :	: — :	: — :	: — :
: — :	: — :	: — :	: — :	: — :	: — :

OCCUPANCY

What time were there people at home? How many people?

: — :	___ people	: — :	___ people
: — :	___ people	: — :	___ people
: — :	___ people		

For peer review only - http://bmjopen.bmj.com/site/about/guidelines.xhtml

INGENIOUS

We are family BORNINBRADFORD

Weekend day

DATE: (please circle one) **Sat** / **Sun**, (please write in) ____/____/______ (dd/mm/yyyy) Participant ID: ING___________

NOTES:

COOKING

E.g. Lots of smoke from a toaster; Left a frying pan on too long and produced lots of smokes

CLEANING

E.g. Used cleaning sprays on Sun morning (if not recorded in the diary); Used eco friendly or natural cleaning products

PRODUCTS

E.g. Used air fresheners in the living room; Burnt candles in the kitchen

HEATING

E.g. Used portable radiant/electric heaters in the living room & child's bedrooms in the night

PERSONAL CARE

E.g. Smelled smoking/e-cigarettes from neighbours; Used deodorant/perfume in the main bathroom

VENTILATION

E.g. Opened patio and bifold doors to get fresh air; Used an air purifier in the living room; Used an dehumidifier in the bathroom and child's bedroom

OTHER EXPERIENCES RELATED TO AIR QUALITY

E.g. Smelled my neighbour's garden fire from inside of the house; Had drain smells in the bathroom; Smelled something (don't know what it is) from the outside

OCCUPANCY

E.g. No one was at home on Sat; My dog and cat stayed inside of the house

For peer review only - http://bmjopen.bmj.com/site/about/guidelines.xhtml

INGENIOUS

We are family

Diary for Additional Measurements

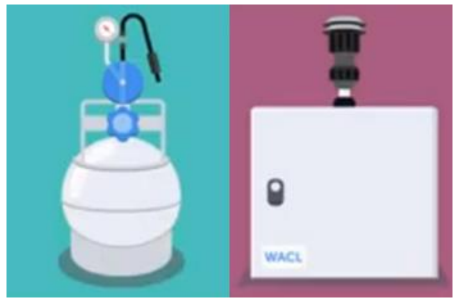

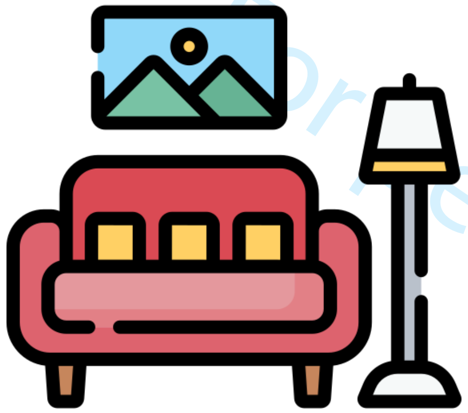

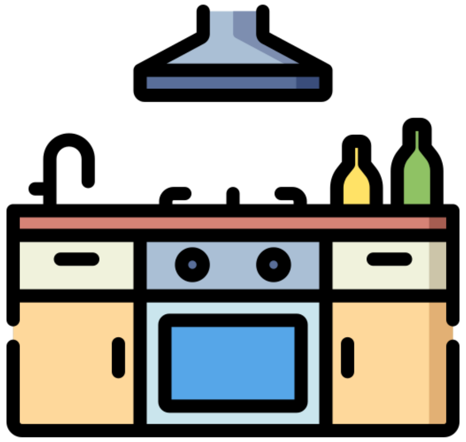

For peer review only

INGENIOUS

We are family
BORNINBRADFORD

Participant ID: ING___________

Additional Measurements

Cooking (PM sampler)

COOKING

Can you please tell us what you cooked for your main meals on each day of sampling?

[Drop-off] Date: __/__/____

Mon | Tue | Wed | Thu | Fri | Sat | Sun

Did you cook today?

YES | NO

If YES, what did you cook for your main meals today?

Did you cook meat (e.g. beef, lamb, chicken, pork) and/or seafood (including fish) today?

YES | NO

Date: __/__/____

Mon | Tue | Wed | Thu | Fri | Sat | Sun

Did you cook today?

YES | NO

If YES, what did you cook for your main meals today?

Did you cook meat (e.g. beef, lamb, chicken, pork) and/or seafood (including fish) today?

YES | NO

Date: __/__/____

Mon | Tue | Wed | Thu | Fri | Sat | Sun

Did you cook today?

YES | NO

If YES, what did you cook for your main meals today?

Did you cook meat (e.g. beef, lamb, chicken, pork) and/or seafood (including fish) today?

YES | NO

Date: __/__/____

Mon | Tue | Wed | Thu | Fri | Sat | Sun

Did you cook today?

YES | NO

If YES, what did you cook for your main meals today?

Did you cook meat (e.g. beef, lamb, chicken, pork) and/or seafood (including fish) today?

YES | NO

Date: __/__/____

Mon | Tue | Wed | Thu | Fri | Sat | Sun

Did you cook today?

YES | NO

If YES, what did you cook for your main meals today?

Did you cook meat (e.g. beef, lamb, chicken, pork) and/or seafood (including fish) today?

YES | NO

NOTES:

For peer review only

DATE: (please circle one) **Mon** / **Tue** / **Wed** / **Thu** / **Fri** / **Sat** / **Sun**, (please write in) ____/____/______ (dd/mm/yyyy) Participant ID: ING____________ Page 3

PLEASE FILL IN IF APPLICABLE

COOKING

What time did you or someone else in your household do the following activities in your home? (24 hour clock)

Frying	Boiling | Steaming	Baking | Grilling Roasting	Using an extractor hood | fan
: — :	: — :	: — :	: — :
: — :	: — :	: — :	: — :
: — :	: — :	: — :	: — :

CLEANING

What time did you or someone else in your household do the following activities in your home? (24 hour clock)

Vacuuming | Dusting	Using cleaning sprays | furniture polishes
: — :	: — :
: — :	: — :
: — :	: — :

PRODUCTS

What time did you or someone else in your household use the following products in your home? (24 hour clock)

Air freshener sprays Plug-in air fresheners Insecticides | Fly sprays	Candle | Incense | Other fragrance burning sources
: — :	: — :
: — :	: — :
: — :	: — :

HEATING

Were the following heating sources on in your home? (Please circle one each)

Central heating	Central boiler
YES | NO	YES | NO

(YES for timer or temperature control is set)

What time did you or someone else in your household use stoves (solid fuel | wood | coal) or open fires? (24 hour clock)

: — :	: — :
: — :	: — :

VENTILATION

What time did you or someone else in your household open the windows or the doors (leading to outside) in the following rooms (*where a sensor is placed)? (24 hour clock)

Living room*	Kitchen*	Bedroom*	Main bathroom	Use an extractor fan
: — :	: — :	: — :	: — :	: — :
: — :	: — :	: — :	: — :	: — :
: — :	: — :	: — :	: — :	: — :

PERSONAL CARE

What time did you or someone else in your household do the following activities in your home? (24 hour clock)

Taking a shower | bath		Using deodorant | aftershave | perfume | hairspray		Smoking | Using e-cigarettes (in | outside of your house)	
: — :	: — :	: — :	: — :	: — :	: — :
: — :	: — :	: — :	: — :	: — :	: — :

OCCUPANCY

What time were there people at home? How many people?

: — :	____ people	: — :	____ people
: — :	____ people	: — :	____ people
: — :	____ people		

For peer review only - http://bmjopen.bmj.com/site/about/guidelines.xhtml

DATE: (please circle one) **Mon** / **Tue** / **Wed** / **Thu** / **Fri** / **Sat** / **Sun**, (please write in) ____/____/______ (dd/mm/yyyy) Participant ID: ING____________ Page 4

NOTES:

COOKING

E.g. Lots of smoke from a toaster; Left a frying pan on too long and produced lots of smokes

CLEANING

E.g. Used cleaning sprays on Sun morning (if not recorded in the diary); Used eco friendly or natural cleaning products

PRODUCTS

E.g. Used air fresheners in the living room; Burnt candles in the kitchen

HEATING

E.g. Used portable radiant/electric heaters in the living room & child's bedrooms in the night

PERSONAL CARE

E.g. Smelled smoking/e-cigarettes from neighbours; Used deodorant/perfume in the main bathroom

VENTILATION

E.g. Opened patio and bifold doors to get fresh air; Used an air purifier in the living room; Used an dehumidifier in the bathroom and child's bedroom

OTHER EXPERIENCES RELATED TO AIR QUALITY

E.g. Smelled my neighbour's garden fire from inside of the house; Had drain smells in the bathroom; Smelled something (don't know what it is) from the outside

OCCUPANCY

E.g. No one was at home on Sat; My dog and cat stayed inside of the house

For peer review only - http://bmjopen.bmj.com/site/about/guidelines.xhtml

Supplementary File 3. Example of feedback report

Indoor air pollution feedback report

Dear [participant],

Thank you for taking part in the INGENIOUS study and providing scientists with important insights into air quality in real homes. Your data will help us understand so much about the air in our homes and how we can improve it.

This is a summary of some of the data we collected in your home using the AirGradient sensors we placed in different rooms (the kitchen, bedroom, and living space). The graphs below show the average results over different time periods, to help highlight where there were peaks. Seeing the times these occur may help you identify things you do that cause these peaks.

Your measurements were taken from [date] to [date] . We measured particulate matter (particles in the air), carbon dioxide (mostly from human breath), and total volatile organic compounds (most of them you can smell).

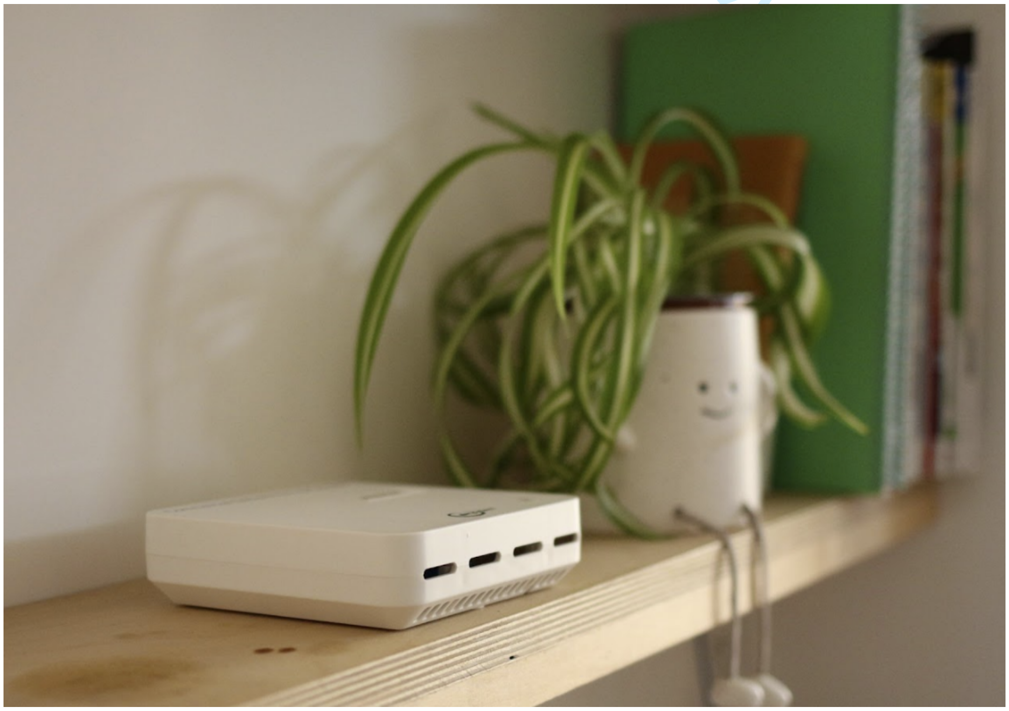

For peer review only - http://bmjopen.bmj.com/site/about/guidelines.xhtml

Particulate Matter in your kitchen

Particulate matter (PM) is made up of particles (tiny pieces) of solids or liquids that are in the air. PM can come from outside, for example dust from the soil or vehicle exhaust. There are also indoor sources of PM, such as cooking. The graphs below show levels of tiny particles in the air that are 2.5 microns or less in diameter ($PM_{2.5}$). They are also called “fine” particles and are believed to pose the greatest risk to health, particularly for people with heart or lung disease (such as asthma or COPD), babies and children, and the elderly.

On the graphs below, you’ll see time along the bottom (x-axis), and the concentration of chemicals up the left (y-axis). Concentrations are typically measured in units of the mass (micrograms μ) of a chemical per volume of air (cubic meter m^3).

```
Daily (average) levels of particulate matter in the kitchen.
 Blue line shows levels we recorded. Dashed grey line shows the
 maximum daily guideline values which have been recommended
 by the World Health Organization.
```

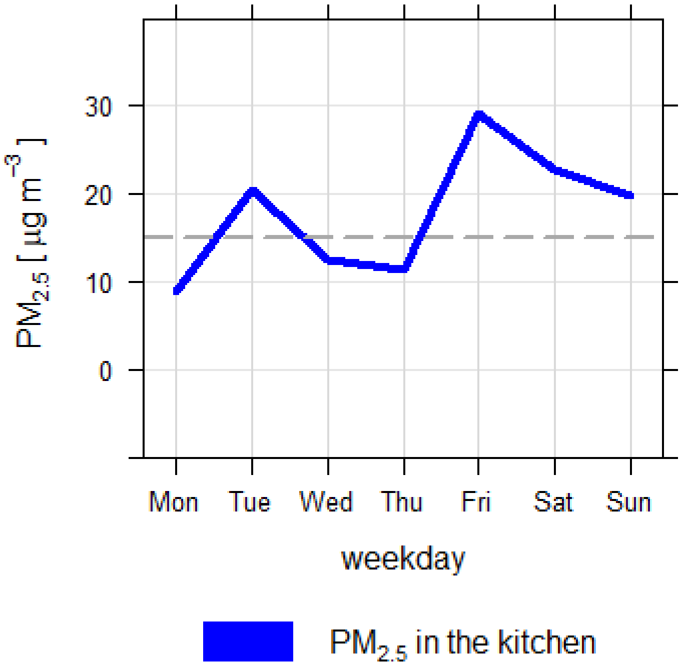

For peer review only

```
Hourly average levels of particulate matter in the kitchen
during the 2 weeks. Time is shown using the 24 hour clock.
```

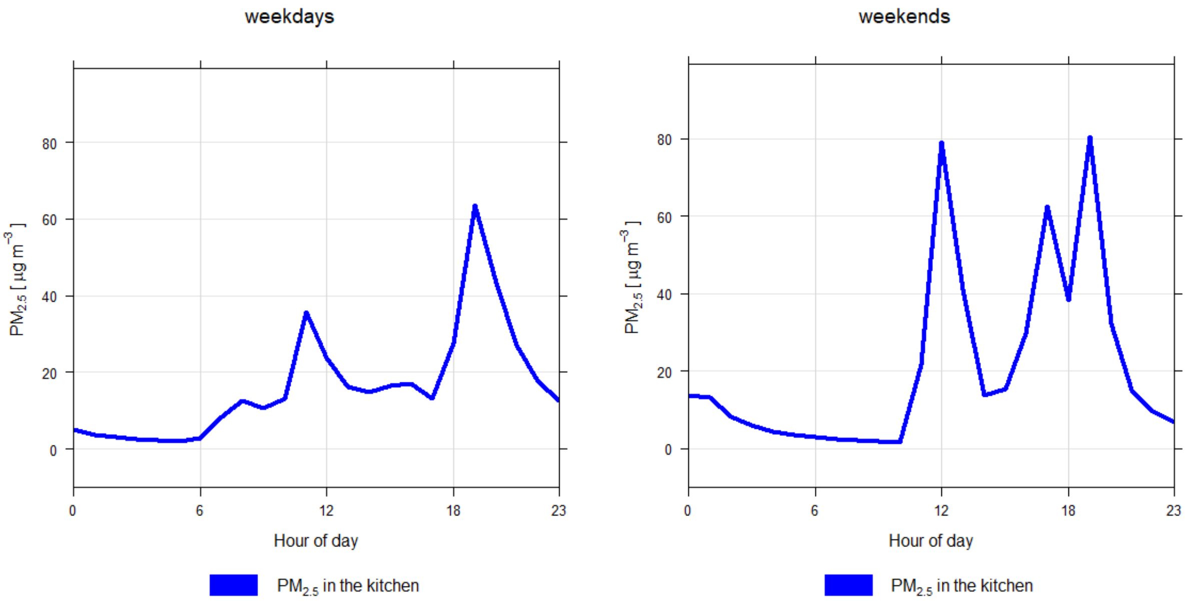

: [illegible] F [illegible] F [illegible]
7 [illegible] ffsfou unz po dfsubo ebzt, psbu J [illegible]
e ffsfou unft pg ebz. Aduwuft uibu dpvme dpouscvuf up ihifs N mfwfmt include cooking, vacuuming or dusting.

If your results are consistently high, you may want to take action to address this, and some suggestions are at the bottom of the document.

Total volatile organic compounds in your living room

Total Volatile Organic Compounds (TVOCs) are a large group of many different chemicals in gas form. They are found in many products we use in our homes. Common indoor sources include personal care products (e.g. deodorants and hair products), paints and cleaning products, smoking and burning wood.

Each chemical has its own toxicity and potential for causing different health effects. Unlike particulate matter, there is no maximum guideline value for TVOCs, and how strong they smell is not a good indicator of

review only

For peer review only - http://bmjopen.bmj.com/site/about/guidelines.xhtml

health risk. High levels of VOCs can cause eye, nose and, throat irritations, nausea, headaches, dizziness and worsening of asthma symptoms. To protect your health, it is best to limit your exposure to products and materials that contain VOCs.

We measured TVOCs using a low cost sensor, which means that the actual measurements may not be accurate. However, the trends in the measurements are accurate, so the graph below shows you when you have peaks in TVOCs.

```
Daily and hourly average levels of total volatile organic compounds
in the living room during the 2 weeks.The time for each day runs fro
shown by the thicker vertical line.
```

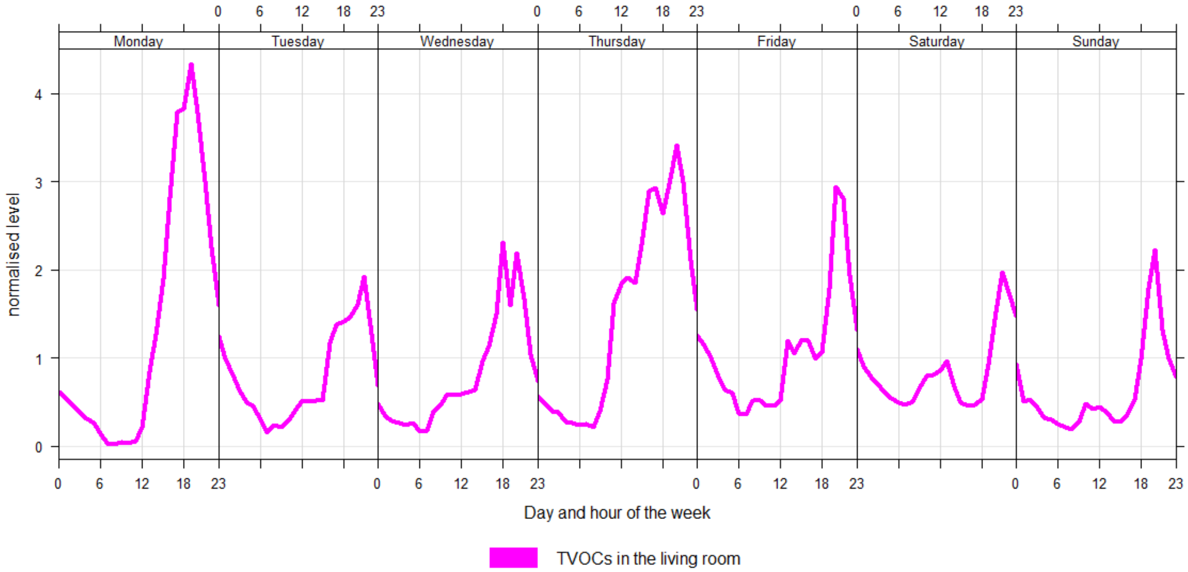

You can see spikes in the TVOCs in the evenings, and sometimes in the morning. What are you doing at that time of day   Is there a link to cooking or cleaning

Carbon dioxide in your child’s bedroom

Carbon dioxide (CO_2) is exhaled in our breath and it is often used as a way to understand how well-ventilated a space is. Studies have shown that high levels may increase the chance of catching virus such as colds, flu, and COVID-19 and also decrease your ability to concentrate.

The graph below shows the hourly average levels of CO_2 in your child's bedroom. It is very likely that levels during the night are higher due to the CO_2 breathed out and lower ventilation rates. These levels are not a cause of serious health concerns. Outdoor CO_2 concentrations are shown in the black dashed line and are usually around 400 ppm (might be a bit higher in urban areas). The unit parts per million (ppm) refers to molecules. For example, 400 ppm of CO_2 means that for every million molecules of air, there are 400 molecules of CO_2.

```
Hourly average levels of carbon dioxide levels in the
bedroom during the 2 weeks. Time is shown using the 24 hour clock.
```

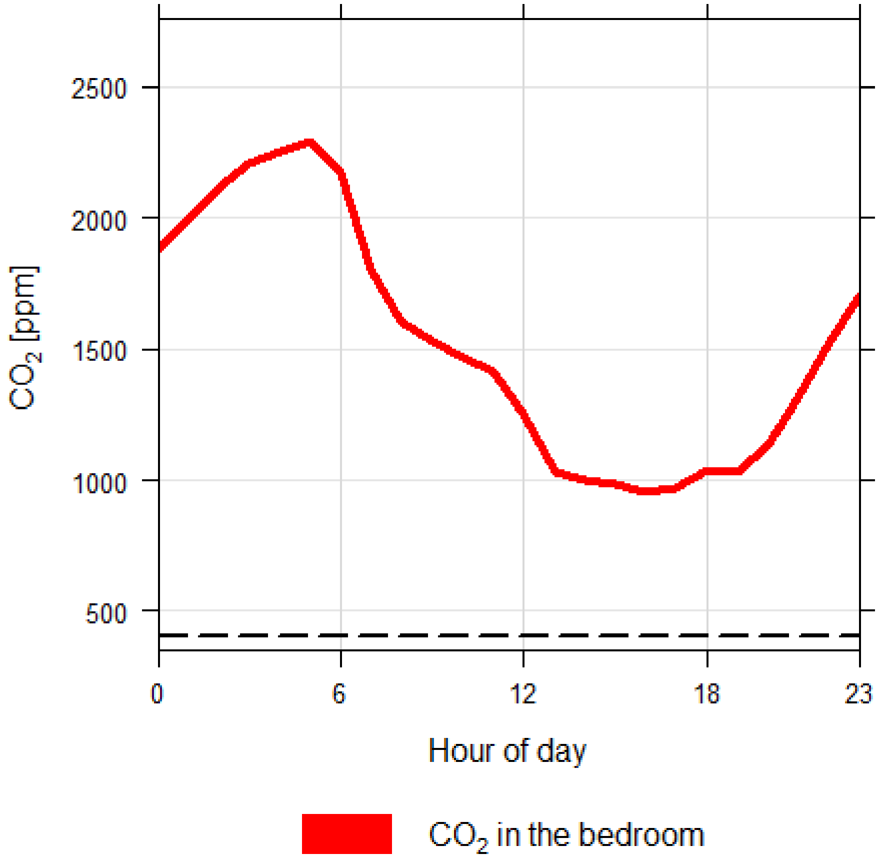

Summary of your indoor air quality

Below we show average levels of particulate matter (in µg/m^3) and CO_2 (in ppm) measured in different rooms over the two week period. The colour

For peer review only - http://bmjopen.bmj.com/site/about/guidelines.xhtml

codes give an indication that you may want to consider some actions to improve the indoor air quality in your home.

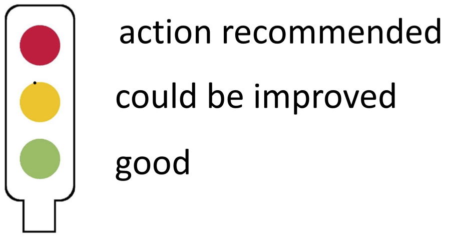

Pollutants	Kitchen	Living_room	Bedroom
$PM_{2.5}$	17	8	18
CO_2	738	749	1527

We have noticed that your maximum PM levels occur at 19 o' clock, most likely during cooking activities. We have also noticed that carbon dioxide concentrations in the bedroom are often quite high exceeding the recommended 1,000 ppm. You may consider ventilating this room more.

Protect yourself and your family

- Even in the cold months, open windows from time to time to allow fresh air to move into the house. This will reduce the levels of many harmful pollutants including PM, TVOCs and CO_2. Aim to open the windows for at least 5 minutes every day, especially if we have found relatively high carbon dioxide levels.
- Use fans/extractors/natural ventilation in the kitchen during cooking to remove fumes, especially if PM levels were above recommended levels.
- Vacuum the carpets and rugs at least once or twice a week to remove dust and allergens, especially in rooms with high PM levels. Consider purchasing low-VOC options for paints and furniture. These information are displayed on the label of these products.
- Where possible, store unused paints, cleaning products or other chemicals in a garage, shed or other place where people do not spend much time. Alternatively, store them in a sealed container.

For peer review only

For peer review only - http://bmjopen.bmj.com/site/about/guidelines.xhtml

We would like to thank you again for your participation. If you have any concerns about these findings, please do not hesitate to contact the BiB Community office at xxxxx xx xxxx

INGENIOUS
Understanding Air Pollution in Homes

[study ID]

For peer review only

For peer review only - http://bmjopen.bmj.com/site/about/guidelines.xhtml

BMJ Open

Understanding the patterns and health impact of indoor air pollutant exposures in Bradford, UK: A study protocol

Journal:	*BMJ Open*
Manuscript ID	bmjopen-2023-081099.R1
Article Type:	Protocol
Date Submitted by the Author:	13-Nov-2023
Complete List of Authors:	Ikeda, Erika; Bradford Teaching Hospitals NHS Foundation Trust, Bradford Institute for Health Research, Born in Bradford<br>Hamilton, Jacqueline; University of York, Department of Chemistry, Wolfson Atmospheric Chemistry Laboratories<br>Wood, Chantelle; The University of Sheffield, Department of Psychology<br>Chatzidiakou, Lia; University of Cambridge, Yusuf Hamied Department of Chemistry<br>Warburton, Thomas; University of York, Department of Chemistry, Wolfson Atmospheric Chemistry Laboratories<br>Ruangkanit, Athina; University of York, Department of Chemistry, Wolfson Atmospheric Chemistry Laboratories<br>Shao, Yunqi; The University of Manchester, Department of Earth and Environmental Science, School of Natural Sciences, Centre for Atmospheric Science<br>Genes, Denisa; The University of Sheffield, Department of Psychology<br>Waiblinger, Dagmar; Bradford Teaching Hospitals NHS Foundation Trust, Bradford Institute for Health Research, Born in Bradford<br>Yang, Tiffany; Bradford Teaching Hospitals NHS Foundation Trust, Bradford Institute for Health Research, Born in Bradford<br>Giorio, Chiara; University of Cambridge, Yusuf Hamied Department of Chemistry<br>McFiggans, Gordon; The University of Manchester, Department of Earth and Environmental Science, School of Natural Sciences, Centre for Atmospheric Science; University of York, National Centre for Atmospheric Science<br>O'Meara, Simon; The University of Manchester, Department of Earth and Environmental Science, School of Natural Sciences, Centre for Atmospheric Science; University of York, National Centre for Atmospheric Science<br>Edwards, Pete; University of York, Department of Chemistry, Wolfson Atmospheric Chemistry Laboratories; University of York, National Centre for Atmospheric Science<br>Bates, Elizabeth; City of Bradford Metropolitan District Council; City of York Council<br>Shaw, David; University of York, Department of Environment and Geography<br>Jones, Roderic; University of Cambridge, Yusuf Hamied Department of Chemistry<br>Carslaw, Nicola; University of York, Environment Department<br>McEachan, Rosemary; Bradford Teaching Hospitals NHS Foundation Trust, Bradford Institute for Health Research, Born in Bradford

**Primary Subject Heading**:	Public health
Secondary Subject Heading:	Public health, Qualitative research, Research methods, Epidemiology
Keywords:	EPIDEMIOLOGIC STUDIES, Health Equity, Observational Study, PUBLIC HEALTH, QUALITATIVE RESEARCH, Surveys and Questionnaires

SCHOLARONE™ Manuscripts

Understanding the patterns and health impact of indoor air pollutant exposures in Bradford, UK: A study protocol

Erika Ikeda[1]

Jacqueline Hamilton[2]

Chantelle Wood[3]

Lia Chatzidiakou[4]

Thomas Warburton[2]

Athina Ruangkanit[2]

Yunqi Shao[5]

Denisa Genes[3]

Dagmar Waiblinger[1]

Tiffany C Yang[1]

Chiara Giorio[4]

Gordon McFiggans[5,6]

Simon P O'Meara[5,6]

Pete Edwards[2,6]

Elizabeth Bates[7]

David R Shaw[8]

Roderic L Jones[4]

Nicola Carslaw[8]

Rosemary R C McEachan[1]*

*Corresponding author

Erika Ikeda

Erika.Ikeda@bthft.nhs.uk

Born in Bradford, Bradford Institute for Health Research, Bradford Teaching Hospitals NHS Foundation Trust, Bradford Royal Infirmary, Duckworth Lane, Bradford, BD9 6RJ, UK

For peer review only

[1]Born in Bradford, Bradford Institute for Health Research, Bradford Teaching Hospitals NHS Foundation Trust, Bradford Royal Infirmary, Bradford, BD9 6RJ, UK

[2]Wolfson Atmospheric Chemistry Laboratories, Department of Chemistry, University of York, York, YO10 5DQ, UK

[3]Department of Psychology, University of Sheffield, Sheffield, S1 4DP, UK

[4]Yusuf Hamied Department of Chemistry, University of Cambridge, Cambridge, CB2 1EW, UK

[5]Department of Earth and Environmental Science, Centre for Atmospheric Science, School of Natural Sciences, University of Manchester, Manchester, M13 9PL, UK

[6]National Centre for Atmospheric Science, University of York, York, YO10 5DQ, UK

[7]Department of Place, City of Bradford Metropolitan District Council, Bradford, BD1 1HX, UK

[8]Department of Environment and Geography, University of York, York, YO10 5NG, UK

For peer review only

For peer review only - http://bmjopen.bmj.com/site/about/guidelines.xhtml

Abstract

Introduction: Relative to outdoor air pollution, there is little evidence examining the composition and concentrations of indoor air pollution and its associated health impacts. The INGENIOUS project aims to provide the comprehensive understanding of indoor air pollution in United Kingdom (UK) homes.

Methods and analysis: 'Real Home Assessment' is a cross-sectional, multi-method study within INGENIOUS. This study monitors indoor air pollutants over two weeks using low-cost sensors placed in three rooms in 300 Born in Bradford (BiB) households. Building audits are completed by researchers, and participants are asked to complete a home survey and a health and behaviour questionnaire, in addition to recording household activities and health symptoms on at least one weekday and one weekend day. A subsample of 150 households will receive more intensive measurements of volatile organic compound and particulate matter for three days. Qualitative interviews conducted with 30 participants will identify key barriers and enablers of effective ventilation practices. Outdoor air pollution is measured in 14 locations across Bradford to explore relationships between indoor and outdoor air quality. Data will be analysed to explore total concentrations of indoor air pollutants, how these vary with building characteristics, and whether they are related to health symptoms. Interviews will be analysed through content and thematic analysis.

Ethics and dissemination: Ethical approval has been obtained from the NHS Health Research Authority Yorkshire and the Humber (Bradford Leeds) Research Ethics Committee (22/YH/0288). We will disseminate findings using our websites, social media, publications and conferences. Data will be open access through the BiB, the Open Science Framework and the UK Data Service.

Strengths and limitations of this study

- We collect detailed assessments of indoor air quality with 300 families in the longitudinal Born in Bradford birth cohort.
- Commercially available, low-cost air pollutant sensors are used to provide summaries of the most common pollutants coupled with survey measurements to capture building characteristics as well as occupant behaviours and health.
- Detailed assessments of volatile organic compounds and particulate matter using state of the art instrumentation and qualitative interviews are conducted in a subsample.
- To minimise participant burden, pollution measurements are collected over a two-week period only.
- Whilst one of the largest assessments of indoor air quality in UK homes to date, large sample sizes will be needed to confidently investigate associations with health outcomes.

For peer review only - http://bmjopen.bmj.com/site/about/guidelines.xhtml

For peer review only

Keywords

Born in Bradford

Indoor air quality

Air pollution

Volatile organic compound

Particulate matter

Carbon dioxide

Asthma

Behaviour change

Ventilation

For peer review only

For peer review only - http://bmjopen.bmj.com/site/about/guidelines.xhtml

1. Introduction

Air pollution is thought to be responsible for between 28,000 and 36,000 deaths each year in the United Kingdom (UK) alone [1]. Long-term exposure to outdoor air pollution has been associated with higher rates of cardiovascular and respiratory illness, birth-defects and neuro-degenerative disorders [2]. Evidence derived from large-scale epidemiological studies indicate a causal relationship between exposure to outdoor air pollution and increased rates of mortality and morbidity [3]. Most measurements, modelling and regulations of air pollution have so far focused on the outdoor environment [4]. Equivalent research for indoor environments lags significantly behind, despite estimations that in developed countries such as the UK, we spend on average around 90% of our time indoors, with approximately two thirds of this in our homes [5]. This may be partly due to limitations in the availability, maintenance and cost of indoor air quality monitors, with nearly all studies of health effects having used data from fixed outdoor air pollution monitoring networks [4]. This lack of data on indoor air pollution may result in inadequate exposure metrics and large uncertainties surrounding its sources and health impacts.

In the UK and globally, there is a dearth of evidence on the sources and transformations of indoor air pollutants [6, 7]. The limited evidence available shows that indoor air is a multidimensional issue influenced by the interrelationship between indoor and outdoor air sources and transformation processes; building design, management and use; and human behaviour (e.g. cooking, cleaning, ventilation) [6]. Since many of these dependencies are specific to location and social factors (e.g. weather conditions, building regulations, cultural and social practices), comprehensive measurements with coordinated UK-specific modelling are needed for understanding indoor air quality relevant to UK homes. Without a fundamental understanding of how air pollution is caused, transformed and distributed in UK homes, research aiming to develop behavioural, technical or policy interventions to reduce future air pollution exposures may have little impact, or even be counterproductive.

The INGENIOUS (understandING the sourcEs, traNsformations and fate of IndOor air pollUtantS) project consists of seven linked work packages to understand the causes and concentration of, and health burden caused by, indoor air pollution (https://ingenious.york.ac.uk/). The current protocol describes research conducted with Born in Bradford (https://borninbradford.nhs.uk/). For ease, we refer to this part of the project as the "Real Home Assessment" study.

2. Methods and Analysis

2.1. Research questions

Real Home Assessment is a cross-sectional, multi-method study which has four research questions:

1) What are the patterns of indoor air pollution in typical UK homes and are there inequalities in exposure to indoor air pollution;
2) How do physical characteristics of buildings or occupant behaviours contribute to indoor air pollution;

For peer review only - http://bmjopen.bmj.com/site/about/guidelines.xhtml

3) How are indoor air pollution and housing quality related to levels of respiratory symptoms and mental health; and
4) What are the barriers and enablers of ventilation behaviours in the home?

Research questions 1-3 will be answered using quantitative data collected from air quality monitors and questionnaires from 300 homes over a 2-week period. Research question 4 will be answered using data from qualitative semi-structured interviews.

2.2. Study design and setting

This study is conducted in Bradford which is an urban, multicultural city located in the North of England, UK. Bradford is the 7th largest metropolitan district in the UK with a population of >546,000 [8]. Approximately 67% of the population identify as White British and 20% as of Pakistani origin [8]. About 34% of Bradford residents live in the most deprived neighbourhoods in England [9]. The Born in Bradford (BiB) is a prospective pregnancy and birth cohort which follows the health and wellbeing of over 12,500 families with children born in the city of Bradford, UK between 2007 and 2011 [10]. Half of the cohort are of South Asian origin, reflective of the demographic of young adults in this population (e.g. more likely to be of child-bearing age) within the city [10]. The study began in August 2022 and will complete by July 2025.

2.3. Participant characteristics and recruitment

A total of 300 families will be recruited from the BiB cohort who took part in the recent follow-up (2017-2020), BiB Growing Up study (in which BiB children were aged 7-11 years) [11]. The sample size of 300 was chosen based on pragmatic considerations including the intensive nature of data collection and the resources available to fund the study. Potential families were identified by stratifying the cohort by child ethnicity (i.e. South Asian, White British, other), housing tenure (i.e. private/mortgaged, rented) and presence of childhood asthma (i.e. asthma: children had asthma symptoms up to two years before the Growing Up data collection; non-asthma) to maximise the variability and relevance of the sample at the local and national levels (Table 1). To be eligible to participate in the Real Home Assessment study, a parent needs to be able to give informed consent; a household suitable to install indoor air quality monitors; and a family able to complete questionnaires and diaries. A family is excluded from the study if a parent is unable to give informed consent and/or communicate in English.

Recruitment of the 300 families began in March 2023 with final visits due to occur in April 2024. Subsamples of 150 and 30 (out of 300) families are further invited for additional assessments of indoor air pollution and qualitative interviews, respectively (see Section 2.4).

Table 1. The recruitment target of 300 BiB families stratified by child ethnicity, housing tenure and childhood asthma

	**Housing tenure**	
	Private/mortgaged property	Rented property

For peer review only - http://bmjopen.bmj.com/site/about/guidelines.xhtml

		(N=210; 70%)		(N=90, 30%)	
		Asthma (50%)	Non-asthma (50%)	Asthma (50%)	Non-asthma (50%)
**Ethnicity**	South Asian (N=135; 45%)	N=48; 16.0%	N=47; 15.7%	N=20; 6.7%	N=20; 6.7%
	White British (N=135; 45%)	N=48; 16.0%	N=47; 15.7%	N=20; 6.7%	N=20; 6.7%
	Other (N=30; 10%)	N=10; 3.3%	N=11; 3.7%	N=5; 1.7%	N=4; 1.3%

2.4. Procedure

Figure 1 outlines the procedure of recruitment and data collection which involves placing air quality monitors (including AirGradient sensors, volatile organic compound [VOC] canister and particulate matter [PM] sampler) in participants' homes and conducting survey measurements and qualitative interviews over a 2-week period. We deploy a tiered approach to indoor air quality assessment, and the sample size is selected to maximise breadth of data and be deliverable within the funded project timelines (Figure 2).

----- Insert Figure 1 here -----

Eligible participants who have previously given consent to be contacted by BiB receive a phone call from the research team for recruitment. Participants are provided with the aim of the study and an overview of the study timeline and data collection. If the family is willing to take part in the study, a home visit is scheduled at a convenient time. Participant information sheets are provided electronically via email to participants before, or in person during the first home visit where written informed consent is obtained and the participants have the opportunity to ask questions.

----- Insert Figure 2 here -----

Data collection occurs over 14 days with two home visits. During the first home visit (Day 1), a member of the research team places a portable Wi-Fi hub at the participant's property, and installs three low-cost (i.e. AirGradient) sensors, which are connected to the Wi-Fi hub in three rooms (where participants are more likely to spend time and pollutants are likely to be generated): a living room, a kitchen and a child's bedroom (or the room where a BiB child sleeps) [12]. The researcher then conducts a building audit to identify key characteristics of the property, and the participant completes a home survey which assesses the condition of their house. Instructions are given by members of the research team on how to complete the provided activity and symptom diaries over the next two weeks. On the same visit, a subsample of participants are

For peer review only

also invited to take part in a 3-day period of more intensive monitoring of VOCs in a living room and PM in a kitchen which would take place at the end of the 2-week period (Figure 2). Among the families agreeing to take part in additional VOC and PM monitoring, an appointment is agreed between the research team and participant for an additional home visit (preferably on Day 10 or 11) to install a VOC canister and a PM sampler and provide another set of diaries. In the PM samplers, the filters are used for either bulk particle chemical composition (N=100) or detailed particle speciation (N=50) analyses (Figure 2).

On Day 14, the researcher returns to collect the air quality monitors and diaries. The participant completes a questionnaire on the health and behaviours of the participant and their child(ren) over the past two weeks. The subsample of families who complete the additional VOC and PM monitoring are further invited for a qualitative interview exploring barriers and enablers to ventilation behaviours in the home. A total of 30 semi-structured interviews are conducted online or in-person after obtaining informed consent.

Following the completion of the data monitoring period, participants are provided with a personalised feedback report outlining the levels of air pollution in their home measured based on the sensors' readings, and are given the opportunity to ask questions about the study. All participants receive a £50 voucher in recognition of their time and effort, and those participating in the interview receive an additional £20 voucher.

2.5. Indoor air quality measurements

2.5.1. AirGradient sensor

All homes receive commercially available low-cost sensors, AirGradient [13] that measures (1-min resolution) and transmits (5-min average intervals) air pollution data to secure server through a Wi-Fi hotspot provided by the research team. The sensor was selected based on its performance in validation tests, ease of use, size, robustness, price and low noise operation to minimise disturbance of occupants (Figure 3). The AirGradient contains a series of sensors: a SenseAir S8 carbon dioxide (CO_2) sensor using non-dispersive infrared technology to measure CO_2 concentration (parts per million by volume [ppm]); a Plantower PMS5003 sensor with laser scattering technology to measure three size fractions of PM concentration (PM_1, $PM_{2.5}$, PM_{10} in micrograms per cubic metre [$\mu g/m^3$]); a Sensirion SGP41 total volatile organic compound (TVOC) / nitrogen oxides (NO_X) sensor to measure TVOC concentration (parts per billion by volume [ppb]); and a Sensirion SHT3x/4x sensor to measure temperature and relative humidity. Where possible, the sensors are placed on a table or shelf and away from external walls and windows [14] in a living room, kitchen and BiB child bedroom while minimising interference with normal occupant behaviours.

----- Insert Figure 3 here -----

For peer review only - http://bmjopen.bmj.com/site/about/guidelines.xhtml

2.5.2. VOC canister

In a subsample of 150 homes, a VOC canister collects air over a 3-day period. Six-litre vacuum-intake stainless-steel canisters from either Entech (CA, USA) or Restek (PA, USA) are used, and a flow-restrictive inlet is attached (Figure 4). Prior to deployment, each canister is evacuated with a pressure below 0.01 Pa to ensure there is no residual ambient air in the canister. During the deployment, the research team attaches a flow-restrictive inlet onto a canister and places the canister on the floor within the living space and away from external walls and windows [14] while minimising interference with normal occupant behaviours. The canister air sample collected from each home is processed off-line in a laboratory, using a custom thermal desorption unit linked to a two-column Agilent 7890A gas chromatograph utilising flame ionisation detection and an Agilent 5977A quadrupole mass spectrometer (GC-FID-QMS) [15]. A broad range of compounds of different molecular sizes and chemical functionality are targeted including ethane from natural gas, butane from aerosol spray products, monoterpenes from fragrances, and ethanol from solvent use.

----- Insert Figure 4 here -----

2.5.3. PM sampler

The same participant subsample receiving a VOC canister (N=150) also host samplers to collect PM onto filter papers for analysis using advanced analytical instrumentation. A Minivol Tactical Air Sampler with a $PM_{2.5}$ impactor protruding from the top is used to collect PM less than 2.5 microns in diameter onto pre-based 47 mm filter papers (Figure 5). The sampler draws air through the polytetrafluoroethylene filter using a pump set at 3 $L \cdot min^{-1}$ over the course of approximately 72 hours. The exact sample time is recorded by a trained researcher who starts and stops the sampling process. To minimise the sound from the pump, the sampler is enclosed by a custom-built metal box lined with sound proofing foams (Dodo Sound Stopper Pro v2) (see Figure 5). The soundproofing enclosure reduced the sound emitted by 10 dBA to a final sound level of 50 dBA. Where possible, the sampler is placed on a kitchen countertop or table and away from windows [14]. Out of 150 filter samples, two-thirds (N=100) are analysed to characterise the bulk chemical composition of particles. A 25mm punch is excised from the 47 mm filter to subsequently analyse organic aerosol composition using a time-of-flight chemical ionisation mass spectrometer with iodide ionisation coupled with a filter inlet for gases and aerosols (FIGAERO-CIMS) [16, 17]. An appropriate fraction of residual filter material will be extracted using methanol and Aerodyne high-resolution time-of-flight aerosol mass spectrometer (HR-ToF-AMS) [18] to analyse both bulk inorganic and organic chemical composition. One third (N=50) of the filter samples collected are analysed to obtain the concentrations of specific chemical compounds that are tracers of specific PM sources and/or are known to have toxic effects in humans. The filters are extracted using accelerated solvent extraction and analysed using two-dimensional gas chromatography coupled with time-of-flight mass spectrometry (GC×GC-ToF-MS) [19].

For peer review only - http://bmjopen.bmj.com/site/about/guidelines.xhtml

----- Insert Figure 5 here -----

2.6. Outdoor air quality monitoring

In collaboration with the City of Bradford Metropolitan District Council, outdoor air pollutants (PM_{10}, $PM_{2.5}$, NO_X, ozone [O_3], CO_2) are being measured using Earthsense Zephyr [20] devices in 10 locations across the Bradford city since June 2023. In addition, four AQMesh [21] units which measure the same target pollutants as the Zephyr devices have been deployed in the residential areas of Bradford since July 2023, following a 2-month collection period with reference devices at the Manchester Air Quality supersite. The data from both devices will be combined with those from local reference monitors to estimate outdoor air pollution in the vicinity of participants' homes, and thus examine the influence of indoor and outdoor air exchange on indoor air pollutant measurements.

2.7. Survey measurements

Table 2 summarises the four surveys (i.e. home survey, building audit, health and behaviour questionnaire, activity and symptom diary) which are available in Supplementary Files 1 and 2. These measurements have been developed based on validated questionnaires, existing evidence and input from experts in indoor air quality. Considering participant burden, these measurements (except the diary) are primarily administered online using REDCap (Research Electronic Data Capture) [22] which potentially reduces the amount of time taken to complete the measurements (due to branching/skip logic) and aids understanding of questions (due to visuals), compared with the paper-and-pencil version. The online platform also reduces researcher burden and errors (entering and managing data).

For peer review only - http://bmjopen.bmj.com/site/about/guidelines.xhtml

Table 2. Summary of surveys

	Home survey	Building audit	Health & behaviour questionnaire	Activity and symptom diary
Completed on	Day 1	Day 1	Day 14	*Household behaviour*: one weekday & one weekend day (N=300); one day during the 72-hour monitoring period (N=150)<br>*Health symptoms* (N=300): Days 1-14<br>*Meals cooked at home* (N=150): Days 10/11-14
Completed by	Participant	Researcher	Participant	Participant
Content	*Section I: Home*	*Section I: House*	*Section I: Your perception of your home & air quality*	*Household behaviour*
	- Residence at the address	- Property type & age	- Home satisfaction	- Cooking
	- Housing tenure	- Building level	- Air quality perception	- Cleaning
	- Outdoor environment	- Energy Performance Certificate (EPC) rating	*Section II: You/your household's behaviour at home*	- Products
	- Number, type & state of rooms	- Outdoor environment	- Meal time	- Heating
	- Presence of mould & damp	- Number of windows & doors leading to the outside	- Use of non-stick cookware, personal care products, fragrances & cleaning products	- Ventilation
	- Heating type	- Sensor ID & location	- Frequency of dusting & vacuuming	- Personal care
	- Cooking fuel type	*Section II: Room*	- Method & location of washing & drying clothes	- Occupancy
	- Ventilation type	- Room size	- Use of solid fuel appliance	- Other experiences related to air quality
	- Presence of chimney	- Floor material	*Section III: Health*	*Individual* health symptom*
	- Home occupancy	- Widow direction	- Asthma (BiB child & parent)	- Time at home
	- Pet ownership	- Use of curtains/blinds/shutters	- Rhinitis (BiB child & parent)	- Asthma
	- Use of air purifier		- Eczema (BiB child & parent)	- Rhinitis
	Section II: Behaviour		- Behavioural screening (BiB parent)	- Eczema
	- Smoking habit		- Mental wellbeing (BiB parent)	- Mood

For peer review only

	Section IV: About you and your household	*Additional measurements*
	Household size	- Type of main meals cooked
	- Food security	
	- Financial security	

BiB=Born in Bradford. *Members of the family (e.g. BiB mother, BiB child).

For peer review only

For peer review only - http://bmjopen.bmj.com/site/about/guidelines.xhtml

2.7.1. Home survey

The 40-item home survey collects information on the condition and characteristics of the participant's home, the outdoor environment around the home, and people's smoking behaviours inside and outside the home (Supplementary File 1). The survey is self-administered electronically using a tablet or on paper by a participant during the first visit. The survey takes approximately 10 minutes to complete.

2.7.2. Building audit

The 32-item building audit collects information on the type of property and the characteristics of the rooms in which the sensors are installed known to affect indoor levels of air pollution such as room size, presence and placement of windows, and presence of extractor fans or hoods (Supplementary File 1). A trained researcher completes the audit electronically using a tablet or on paper during the first visit. The audit takes approximately 10 minutes to complete.

2.7.3. Health and behaviour questionnaire

The 250-item health and behaviour questionnaire assesses individual symptoms of child and parent respiratory and allergic health (International Study of Asthma and Allergies in Childhood [ISAAC] [23]; Global Asthma Network [24]), child behavioural screening (Strengths and Difficulties Questionnaire [25, 26]) and parent mental health (Short Warwick-Edinburgh Mental Wellbeing Scale [27]; Patient Health Questionnaire – Depression Module [28]), as well as socio-economic circumstances and household behaviours such as ventilation and cooking habits (Supplementary File 1). A participant completes the questionnaire electronically using a tablet or on paper on Day 14. The questionnaire takes approximately 20 minutes to complete.

2.7.4. Activity and symptom diary

Paper-based diaries are used to record key household activity patterns (28 items) and 14 individual health indicators of respiratory and allergic symptoms (based on ISAAC [23]) and mood (based on Visual Analogue Mood Scale [29] (Supplementary File 2). Individual health symptoms are self-reported by members of the family (e.g. BiB mother, BiB child) every day during the 2-week period. For household activity patterns, the participants are asked to select one weekday and one weekend day to record time spent doing key household activities that are related to indoor air pollution (e.g. cooking, cleaning, ventilation), and provide a description of relevant activities using free text. For the subset of participants who host a VOC canister and a PM sampler (N=150), activities are recorded for one additional day and main meals cooked at home are recoded every day during the 72-hour monitoring period.

2.8. Qualitative interview

Content for the semi-structured interviews was developed based on the COM-B (Capacity, Opportunity, Motivation – Behaviour) Model [30] which provides a systematic framework for understanding behaviours in terms of people's physical and psychological capability: physical and social opportunity; and automatic and reflexive motivation. The interview takes approximately 60 minutes, and is conducted either face-to-

For peer review only - http://bmjopen.bmj.com/site/about/guidelines.xhtml

face or online, depending on the participant's preference, and will be audio-recorded and transcribed. An interview guide is available on the Open Science Framework [31].

2.9. Data analysis plan

Quantitative data from the air quality monitors (i.e. the concentration of pollutants and compounds) and survey measurements will be reported descriptively using frequencies (for categorical variables), or medians and inter-quartile ranges or means and standard deviations (for continuous variables). Any qualitative data from the survey measurements (i.e. text) will be used as supplementary information for air pollution data. Any missing data of the variables measured and reasons for their absence will be examined, reported and handled appropriately in analyses. We do not expect any missing socio-demographic data as the data have already been collected through the BiB cohort study (e.g. Growing Up [11]). Based on the research questions, we will select appropriate statistical methods to analyse the data, as specified below.

2.9.1. What are the patterns of indoor air pollution in typical UK homes and are there inequalities in exposure to indoor air pollution?

Descriptive analyses of the AirGradient sensor data will be performed, including examining daily concentrations of indoor air pollution (means or medians), peaks in exposure and daily time spent above the World Health Organization (WHO) or UK recommendations for air pollution [32–34]. Compounds with known toxicological effects will be highlighted and compared to the WHO or UK guidelines for indoor air quality [32–34]. All the air pollution data including CO_2, VOCs and PM (concentration and composition) will be examined with all participants together as well as stratified by housing tenure (i.e. private/mortgaged, rented), ethnicity (i.e. South Asian, White British, Other), and Indices of Multiple Deprivation (IMD; in quintiles) in England (national) and Bradford (local). Differences within each variable will be examined using independent T-test, analysis of variance (ANOVA) or Wilcoxon-Mann-Whitney test.

2.9.2. How do physical characteristics of buildings or occupant behaviours contribute to indoor air pollution?

To identify subgroups of the participants based on shared factors, we aim to (i) identify latent profiles of building characteristics and occupant behaviours using latent class analysis, and (ii) examine whether these profiles are associated with indoor air pollution using mixed effect models, adjusting for covariates including housing tenure, ethnicity and English or Bradford IMD using regression models.

2.9.3. How are indoor air pollution and housing quality related to levels of respiratory symptoms and mental health?

Mixed effects models will be used to examine the relationship between changing indoor air pollution levels on respiratory symptoms and mood. The exposure variable will be measured as continuous (e.g. the daily and 2-week average concentration of indoor air pollution) or binary (e.g. the daily amount of time pollution levels exceeded the WHO or UK guidelines for indoor air quality [32, 34]). Daily occurrences of respiratory

For peer review only - http://bmjopen.bmj.com/site/about/guidelines.xhtml

symptoms (binary outcome: yes/no) and daily measures of mood (continuous outcome: 1-10) assessed using the symptom diaries will be examined using mixed effects logistic regression and mixed effects linear regression, respectively. Multivariable logistic regression, adjusting for relevant confounding variables, will be used to explore the association between indoor air pollution (i.e. 2-week average concentrations) and the health outcomes (i.e. the state of respiratory health and mental wellbeing derived from the health and behaviour questionnaire). Covariates will be included to reduce confounding and improve precision including subject-specific (e.g. age, sex, ethnicity, British or Bradford IMD, smoking habits) and time-dependent (e.g. day of the week, season, temperature, relative humidity).

To examine the lag relationship over time between exposure (indoor air pollution) and outcomes (respiratory symptoms and mood), we will assess the same day (lag 0), 1-day, 2-day and 3-day lags as well as the average of lag 0-3 days. Potential effect modification by housing quality (e.g. housing tenure, presence of damp) and season will be examined.

2.9.4. What are the barriers and enablers of ventilation behaviours in the home?

Anonymised interview transcripts will be coded and analysed using NVivo qualitative data analysis software [35] or paper-based techniques. A hybrid deductive/inductive content and thematic analysis approach will be employed, based on the methods described by Atkins et al. [36], Smith et al. [37] and Prothero et al. [38]. This will consist of two phases which entail development of an initial codebook based on the determinants of behaviour (i.e. domains) specified in the Theoretical Domains Framework (TDF) [39], mapped onto the COM-B framework [30]. In Phase 1, 10% of the transcripts will be independently deductively content analysed by two researchers, by coding text to the TDF domains in the codebook. We will also retain flexibility to identify additional codes not encompassed by the TDF during this stage, by adding these to the codebook where relevant. . Coding will then be discussed by the two researchers, with the aim of building consensus, and refining the codebook. Where consensus cannot be reached, a third researcher will be consulted, or the relevant text will be attributed to more than one TDF domain. The first researcher will then use the revised codebook to code the remainder of the transcripts, adding and discussing any revisions or additional codes with the second researcher or broader team as needed.

In Phase 2, an inductive thematic analysis will be conducted on the text marked with codes during Phase 1, following the process identified by Braun and Clarke [40] and focused on identifying specific barriers and enablers of ventilation behaviours. Ten percent of transcripts will be independently coded by two researchers, and an initial list of inductive codes will be added to each researcher’s codebook under the code headings from Phase 1. The first researcher will then use the revised codebook to code the remainder of the transcripts, adding and discussing any revisions or additional codes with the second researcher or broader team as needed. Themes will then be generated, reviewed and defined by the first researcher, in discussion with the second researcher or broader team. Content relating to current ventilation behaviours will be summarised descriptively. Potential targets for intervention will then be prioritised by the first researcher in discussion with the broader team, based on the following criteria: (1) frequency of theme (i.e. number of participants reporting a specific barrier or enabler); (2) number of barrier or enabler themes per

For peer review only - http://bmjopen.bmj.com/site/about/guidelines.xhtml

TDF domain (where relevant); (3) discordance within or across themes (i.e. the same factor acting as a barrier and facilitator of behaviour in different participants or in different contexts); (4) perceived importance of theme for participants.

2.10. Patient and public involvement

In Real Home Assessment, public involvement has been central to the development of the study and has been embedded within all research activities including the design, conduct and dissemination of the study. BiB has long-established public participation groups (e.g. parent governors, public research advisory group) which meet regularly to provide lived experience, expertise and guidance to the research team. The groups, for example, provided us feedback on the feasibility of the survey measurements (e.g. item wording, survey length) during the pilot testing. We have used a variety of different channels to give members of the public a chance to engage in the air pollution research, including hosting regular 'open space' community meetings, attending existing community events (e.g. science festivals), and circulating briefing notes and newsletters. For study participants, we provide a personalised feedback report (Supplementary File 3) after the completion of the data monitoring period.

2.11 Ethics and dissemination

Ethical approval has been obtained from the National Health Service Health Research Authority Yorkshire and the Humber (Bradford Leeds) Research Ethics Committee: 22/YH/0288, 11th January 2023. Participants give informed consent by receiving an information sheet and signing a consent form after they have had the opportunity to discuss the project with a member of the research team. We will disseminate research outputs using various channels including our INGENIOUS project website [41], BiB website [42], social media, scientific publications, press releases as well as conference presentations and posters. Air quality and health data will be open access via existing Born in Bradford data access procedure [42]. An open-access dataset for the qualitative interviews (i.e. anonymised transcripts) will be also made available on the UK Data Service, and the interview materials are available on the Open Science Framework [31].

3. Discussion

Exposure to airborne pollutants during childhood can increase the risk and exacerbation of airway diseases with lifelong consequences [43, 44]. Yet, there is little evidence to constrain or quantify indoor air pollutant emissions, building-to-building variability, chemical speciation of indoor air pollutants, interactions between indoor- and outdoor-generated air pollution, or environmental and socio-economic factors that can contribute to elevated indoor air pollutant exposures. Real Home Assessment as part of the INGENIOUS project is one of the first research studies that measures indoor air pollution in UK homes at a large scale. Findings from the Real Home Assessment will contribute to the understanding of how indoor air pollution in UK homes is generated, prevented and reduced, as well as insights into social, ethnic and health inequalities associated with indoor air pollution.

For peer review only - http://bmjopen.bmj.com/site/about/guidelines.xhtml
For peer review only

Deprived households are more likely to experience poor indoor air quality than more affluent households due to factors such as greater exposure to second hand smoke and higher outdoor air pollutant concentrations [2]. However, the reality may be considerably more nuanced. On one hand, lower quality and older housing may be less airtight (i.e. less energy efficient) than new-build homes allowing outdoor air pollution to penetrate inside but indoor emissions to escape more easily. On the other hand, large, expensive town houses converted to flats can be poorly ventilated, and suffer from higher indoor-generated air pollutant concentrations following poor retrofitting practices [2].

Recruiting households from the BiB cohort in the current study is valuable in terms of the diversity of the cohort (covering a multi-ethnic population that has high rates of deprivation) and the detailed data collection (including information about the health, social circumstances and lifestyle characteristics of children). Over 30% of BiB families live in a rented property, one-third of families have more than five occupants, and 13% of families have at least one child with doctor-diagnosed asthma (based on the Growing Up data [11]). This profile makes the BiB cohort a particularly relevant population group in which to explore patterns of indoor air pollution.

The tiered approach of air quality monitoring allows us to provide both summary (with common pollutant concentrations) and detailed (with a large number of pollutant speciation) assessments of indoor air quality. Indoor air quality and health is an under-researched area, and this study will be one of the largest to date will collect detailed measures of indoor air pollution and health outcomes [45]. Along with the air quality monitors, we use four survey measurements to capture potential contributing emission sources of indoor air pollution from the building and occupant behaviours. Results from the semi-structured interviews will be used to inform the design of behaviour change interventions to reduce exposure to indoor air pollutants. These interventions will be co-designed with community members and evaluated in a later stage of the INGENIOUS project and will be described in a separate protocol.

Real Home Assessment is a cross-sectional study which captures a snapshot of indoor air pollutant concentrations and occupant behaviours and health symptoms over a 2-week period. The sample size of 300 families was selected to maximise the breadth of data and be deliverable within the funded project timelines. While this will be one of the largest studies of indoor air pollution in UK homes to date, it will stil be underpowered to definitively investigate associations between indoor air quality and health outcomes. Given the burdensome nature of data collection, it will be important for researchers in this area to harmonise methods to allow combining data from similar studies across the UK. We publish this protocol and share our methods as a first step in this wider endeavour. Recruiting participants and collecting data (including air pollutant concentrations and occupant behaviours) in the domestic setting can be more challenging and resource-intensive than doing so in public spaces and measuring outdoor air pollution through national and local authorities [7]. Thus, we consider Real Home Assessment as exploratory research to assess the feasibility of collecting indoor air pollution in homes, which is likely to be the primary indoor environment where most people spend their time. However, we cannot ignore air pollution exposures in other indoor settings such as schools, workplaces, transport and shops. Future research will

For peer review only - http://bmjopen.bmj.com/site/about/guidelines.xhtml

aim to capture total personal exposure using multi-pollutant sensor platforms [46] and advanced computational modelling for time-activity patterns [47].

For peer review only

For peer review only - http://bmjopen.bmj.com/site/about/guidelines.xhtml

Figure legends

Figure 1. The procedure of recruitment and data collection. VOC = volatile organic compound; PM = particulate matter.

Figure 2. Tiered indoor air quality assessment. VOC = volatile organic compound; PM = particulate matter. *150 filters collected from PM samplers are analysed for either bulk particle chemical composition or detailed particle speciation.

Figure 3. Image of the AirGradient sensor

Figure 4. Images of the Entech 6-litre canister and an inlet

Figure 5. Images of the Minivol Tactical Air Sampler and a soundproof box

For peer review only

Acknowledgement

Born in Bradford is only possible because of the enthusiasm and commitment of the children and parents in BiB. We are grateful to all the participants, health professionals, schools and researchers who have made Born in Bradford happen. We would like to acknowledge Kane Armatage and Sally Jones from the City of Bradford Metropolitan District Council for their involvement in outdoor air quality monitoring.

Declarations

Authors' contributions

All authors made substantial contributions to the conception or design of the work, and read and approved the final manuscript. EI, CW, LC, TW, AR, YS, DG, DW, SPO, PE, EB and DRS contributed to the acquisition, analysis or interpretation of data. EI drafted the manuscript, and JH, CW, LC, TW, AR, YS, DG, DW, TCY, CG, GM, SPO, PE, EB, DRS, RLJ, NC and RRCM reviewed and revised it.

Funding

INGENIOUS is funded by UK Research and Innovation Natural Environment Research Council (NE/W002256/1) and has undergone a rigorous peer-review process as part of the application process. RM received funding from the National Institute for Health and Care Research Applied Collaboration for Yorkshire and Humber (NHIR200166). AR is funded by the Natural Environment Research Council PANORAMA Doctoral Training Program. The Zephyr sensor network utilised in the project is operated by City of Bradford Metropolitan District Council and was purchased using a Department for Environment, Food and Rural Affairs Air Quality Grant obtained by the local authority. The funders have no role in the design of the study, the collection, analysis and interpretation of the data, the writing of the manuscript, and the decision to submit the manuscript for publication. The views expressed in this publication are those of the author(s) and not necessarily those of the National Institute for Health and Care Research or the Department of Health and Social Care.

Competing interests

The authors declare that they have no competing interests.

For peer review only

References

1. Office for Health Improvement & Disparities (2022) Air pollution: Applying all our health. https://www.gov.uk/government/publications/air-pollution-applying-all-our-health/air-pollution-applying-all-our-health. Accessed 12 Sep 2023

2. Laverge J, Delghust M, Janssens A (2015) Carbon dioxide concentrations and humidity levels measured in Belgian standard and low energy dwellings with common ventilation strategies. Int J Vent 14:165–180

3. Cohen AJ, Brauer M, Burnett R, et al (2017) Estimates and 25-year trends of the global burden of disease attributable to ambient air pollution: an analysis of data from the Global Burden of Diseases Study 2015. Lancet 389:1907–1918

4. Dominski FH, Lorenzetti Branco JH, Buonanno G, Stabile L, Gameiro da Silva M, Andrade A (2021) Effects of air pollution on health: A mapping review of systematic reviews and meta-analyses. Environ Res 201:111487

5. Klepeis NE, Nelson WC, Ott WR, Robinson JP, Tsang AM, Switzer P, Behar J V., Hern SC, Engelmann WH (2001) The National Human Activity Pattern Survey (NHAPS): A resource for assessing exposure to environmental pollutants. J Expo Anal Environ Epidemiol 11:231–252

6. Department of Health and Social Care (2022) Chief Medical Officer's annual report 2022: Air pollution.

7. Air Quality Expert Group (2022) Indoor air quality. https://doi.org/10.5281/zenodo.6523605

8. City of Bradford Metropolitan District Council (2022) Population. https://ubd.bradford.gov.uk/about-us/population/.

9. Noble S, McLennan D, Noble M, Plunkett E, Gutacker N, Silk M, Wright G (2019) The English Indices of Deprivation 2019: Research report.

10. Wright J, Small N, Raynor P, et al (2013) Cohort profile: The born in bradford multi-ethnic family cohort study. Int J Epidemiol 42:978–991

11. Bird PK, McEachan RRC, Mon-Williams M, et al (2019) Growing up in Bradford: Protocol for the age 7-11 follow up of the Born in Bradford birth cohort. BMC Public Health 19:1–12

12. Royal College of Physicians (2016) Every breath we take: The lifelong impact of air pollution. London, UK

13. AirGradient Limited (2023) AirGradient. https://www.airgradient.com/. Accessed 22 Sep 2023

14. The Chartered Institution of Building Services Engineers (CIBSE) (2020) TM61 Operational performance of buildings. London, UK

15. Warburton T, Grange SK, Hopkins JR, Andrews SJ, Lewis AC, Owen N, Jordan C, Adamson G,

For peer review only - http://bmjopen.bmj.com/site/about/guidelines.xhtml

Xia B (2023) The impact of plug-in fragrance diffusers on residential indoor VOC concentrations. Environ Sci Process Impacts 25:805–817

16. Lopez-Hilfiker FD, Mohr C, Ehn M, et al (2014) A novel method for online analysis of gas and particle composition: Description and evaluation of a filter inlet for gases and AEROsols (FIGAERO). Atmos Meas Tech 7:983–1001
17. Lee BH, Lopez-Hilfiker FD, Mohr C, Kurtén T, Worsnop DR, Thornton JA (2014) An iodide-adduct high-resolution time-of-flight chemical-ionization mass spectrometer: Application to atmospheric inorganic and organic compounds. Environ Sci Technol 48:6309–6317
18. Jimenez JL, Jayne JT, Shi Q, et al (2003) Ambient aerosol sampling using the Aerodyne aerosol mass spectrometer. J Geophys Res Atmos 108:1–13
19. Stewart GJ, Nelson BS, Acton WJF, et al (2021) Emissions of intermediate-volatility and semi-volatile organic compounds from domestic fuels used in Delhi, India. Atmos Chem Phys 21:2407–2426
20. EarthSense Systems Limited (2023) Zephyr. https://www.earthsense.co.uk/zephyr. Accessed 22 Sep 2023
21. Environmental Instruments Limited (2023) AQMesh. https://www.aqmesh.com/. Accessed 22 Sep 2023
22. Vanderblit University (2023) REDCap. https://projectredcap.org/. Accessed 27 Sep 2023
23. Ellwood P, Asher MI, Beasley R, Clayton TO, Stewart a W (2000) Phase Three Manual of the International Study of Asthma and Allergies in Childhood (ISAAC). Int J Tuberc Lung Dis 9:10–16
24. Global Asthma Network (2015) Phase I Manual: Global surveillance: prevalence, severity, management and risk factors. Auckland, New Zealand
25. Goodman R (1997) The strengths and difficulties questionnaire: A research note. J Child Psychol Psychiatry 38:581–586
26. Goodman R (1999) The extended version of the Strengths and Difficulties Questionnaire as a guide to child psychiatric caseness and consequent burden. J Child Psychol Psychiatry 40:791–799
27. Shah N, Cader M, Andrews B, McCabe R, Stewart-Brown SL (2021) Short Warwick-Edinburgh Mental Well-being Scale (SWEMWBS): performance in a clinical sample in relation to PHQ-9 and GAD-7. Health Qual Life Outcomes 19:1–9
28. Kroenke K, Spitzer RL, Williams JBW (2001) The PHQ-9: Validity of a brief depression severity measure. J Gen Intern Med 16:606–613
29. van Rijsbergen GD, Bockting CLH, Berking M, Koeter MWJ, Schene AH (2012) Can a one-item mood scale do the trick? Predicting relapse over 5.5-years in recurrent depression. PLoS One 7:1–5

For peer review only

For peer review only - http://bmjopen.bmj.com/site/about/guidelines.xhtml

30. Michie S, van Stralen MM, West R (2011) The behaviour change wheel: A new method for characterising and designing behaviour change interventions. Implement Sci 6:1–11

31. Wood C, Genes D (2023) INGENIOUS: Co-production and evaluation of behaviour change interventions to improve indoor air quality in the UK. https://osf.io/sf9np/?view_only=76c443e757f84ecbb16f6705ebe0766a. Accessed 6 Oct 2023

32. World Health Organization (2010) WHO guidelines for air quality: selected pollutants. Copenhagen, Denmark

33. World Health Organization (2021) WHO global air quality guidelines: Particulate matter (PM2.5 and PM10), ozone, nitrogen dioxide, sulfur dioxide and carbon monoxide. Geneva, Switzerland

34. Public Health England (2019) Indoor Air Quality Guidelines for selected Volatile Organic Compounds (VOCs) in the UK About Public Health England. London, UK

35. Lumivero (2023) NVivo. https://lumivero.com/products/nvivo/. Accessed 6 Oct 2023

36. Atkins L, Francis J, Islam R, et al (2017) A guide to using the Theoretical Domains Framework of behaviour change to investigate implementation problems. Implement Sci 12:1–18

37. Smith D, Cartwright M, Dyson J, Hartin J, Aitken LM (2021) Barriers and enablers of recognition and response to deteriorating patients in the acute hospital setting: A theory-driven interview study using the Theoretical Domains Framework. J Adv Nurs 77:2831–2844

38. Prothero L, Lawrenson JG, Cartwright M, et al (2022) Barriers and enablers to diabetic eye screening attendance: An interview study with young adults with type 1 diabetes. Diabet Med 39:1–16

39. Cane J, O'Connor D, Michie S (2012) Validation of the theoretical framework. Implement Sci 7:1–17

40. Braun V, Clarke V (2006) Using thematic analysis in psychology. Qual Res Psychol 3:77–101

41. INGENIOUS (2023) INGENIOUS. https://ingenious.york.ac.uk/. Accessed 22 Sep 2023

42. Born in Bradford (2023) Born in Bradford. https://borninbradford.nhs.uk/. Accessed 22 Sep 2023

43. Kelly FJ, Fussell JC (2011) Air pollution and airway disease. Clin Exp Allergy 41:1059–1071

44. Raju S, Siddharthan T, McCormack MC (2020) Outdoor air pollution and respiratory health. Clin Chest Med 41:825–843

45. Parliamentary Office of Science and Technology (POST) (2023) POSTbrief 54: Indoor air quality. https://doi.org/10.58248/PB54

46. Chatzidiakou L, Krause A, Popoola O, et al (2019) Characterising low-cost sensors in highly portable platforms to quantify personal exposure in diverse environments. Atmos Meas Tech 12:4643–4657

For peer review only - http://bmjopen.bmj.com/site/about/guidelines.xhtml

47. Chatzidiakou L, Krause A, Kellaway M, Han Y, Li Y, Martin E, Kelly FJ, Zhu T, Barratt B, Jones RL (2022) Automated classification of time-activity-location patterns for improved estimation of personal exposure to air pollution. Environ Heal 21:1–21

For peer review only

For peer review only - http://bmjopen.bmj.com/site/about/guidelines.xhtml

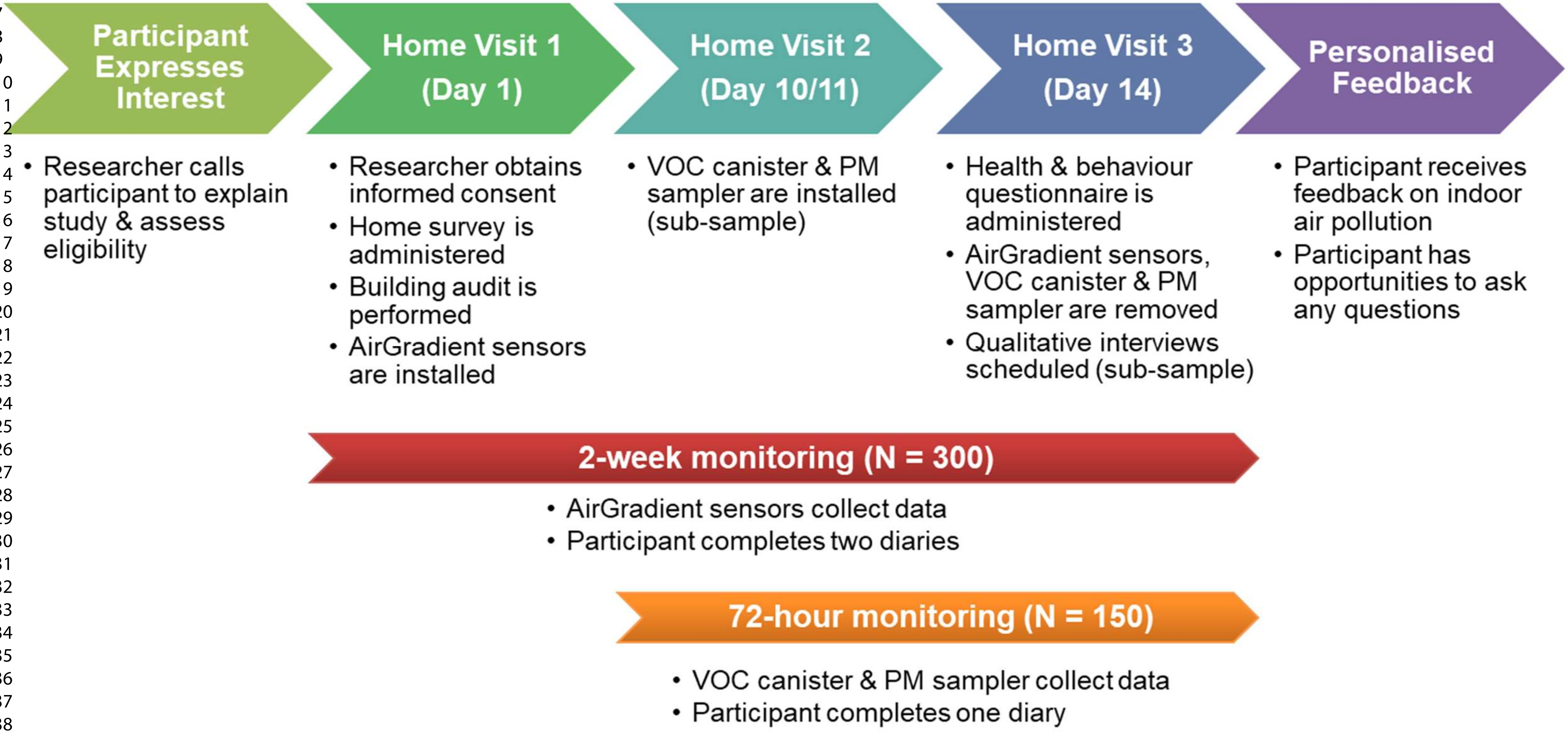
Participant Expresses Interest
Home Visit 1 (Day 1)
Home Visit 2 (Day 10/11)
Home Visit 3 (Day 14)
Personalised Feedback
• Researcher calls participant to explain study & assess eligibility
• Researcher obtains informed consent
• Home survey is administered
• Building audit is performed
• AirGradient sensors are installed
• VOC canister & PM sampler are installed (sub-sample)
• Health & behaviour questionnaire is administered
• AirGradient sensors, VOC canister & PM sampler are removed
• Qualitative interviews scheduled (sub-sample)
• Participant receives feedback on indoor air pollution
• Participant has opportunities to ask any questions
2-week monitoring (N = 300)
• AirGradient sensors collect data
• Participant completes two diaries
72-hour monitoring (N = 150)
• VOC canister & PM sampler collect data
• Participant completes one diary

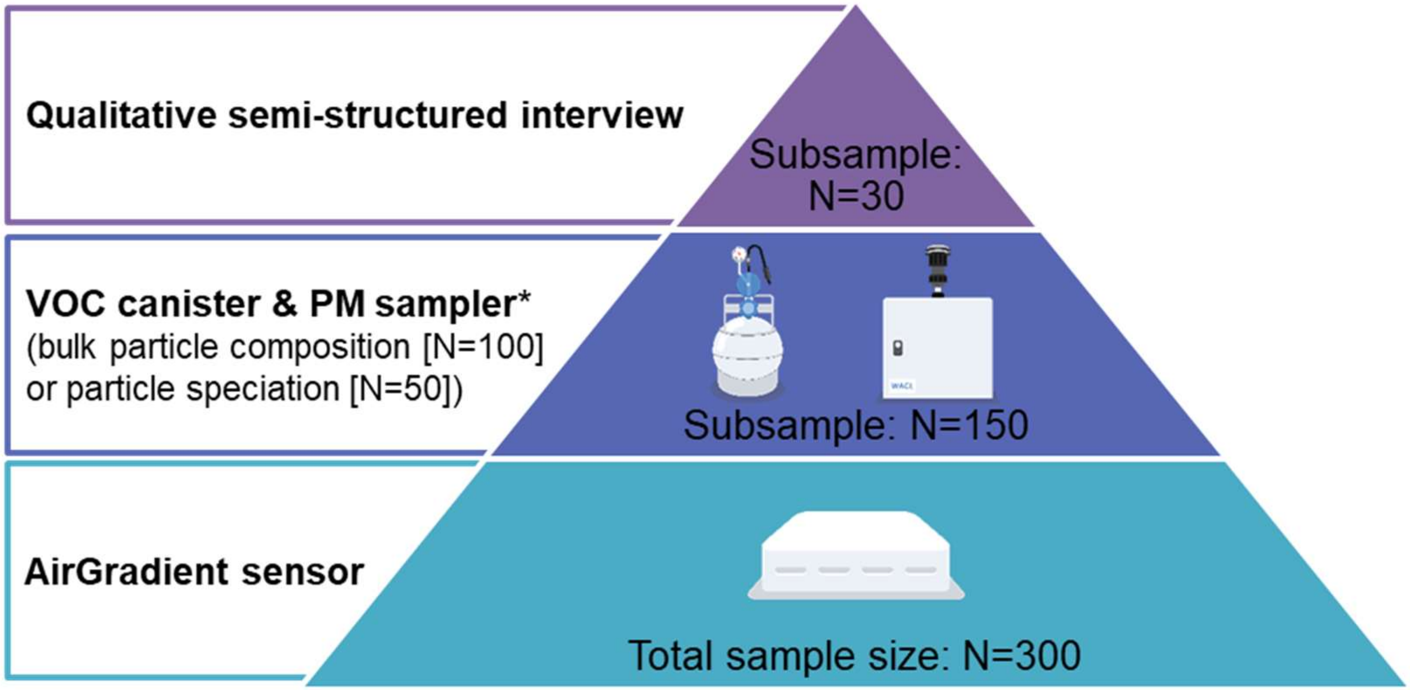
Qualitative semi-structured interview
Subsample: N=30
VOC canister & PM sampler*
(bulk particle composition [N=100]
or particle speciation [N=50])
Subsample: N=150
AirGradient sensor
Total sample size: N=300

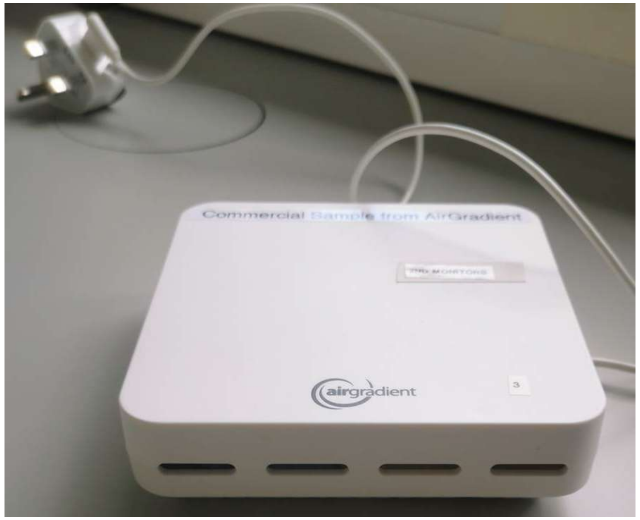
Commercial Sample from AirGradient
airgradient

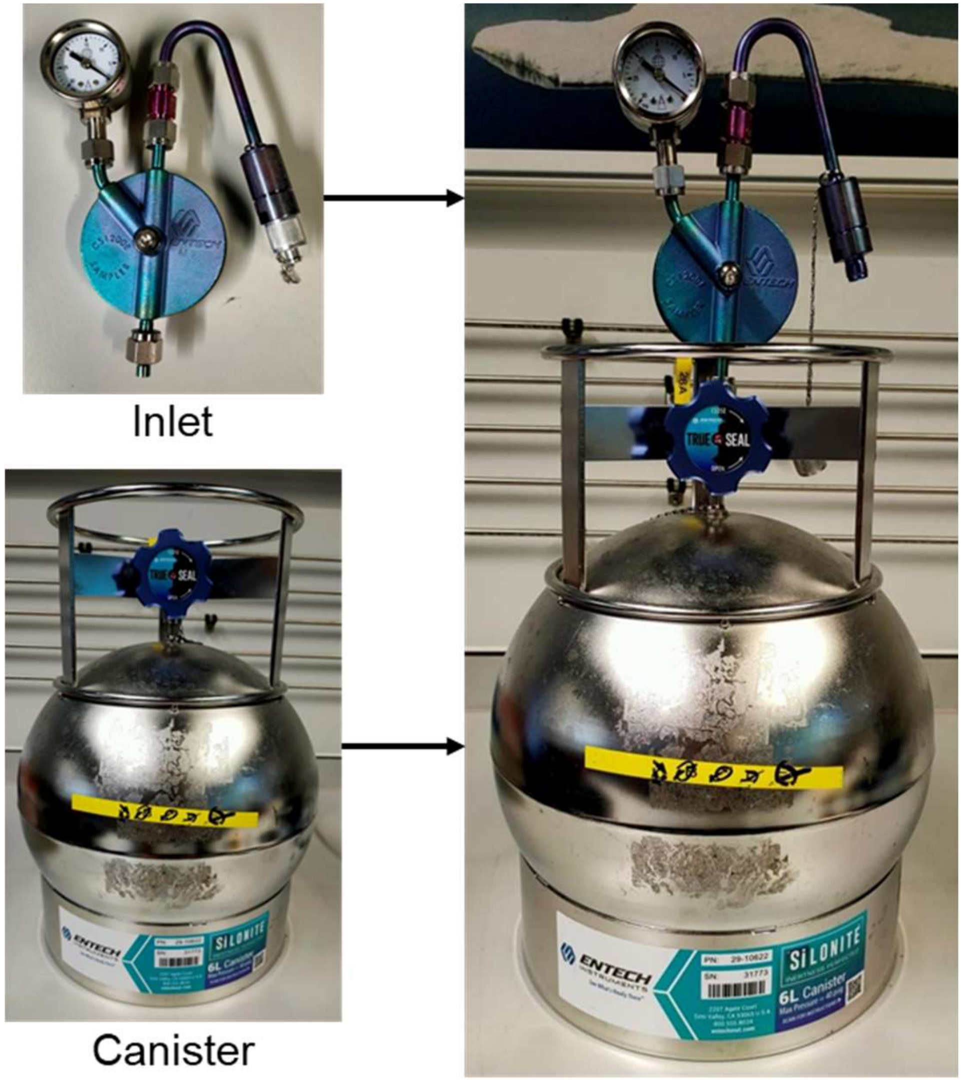
Inlet
Canister

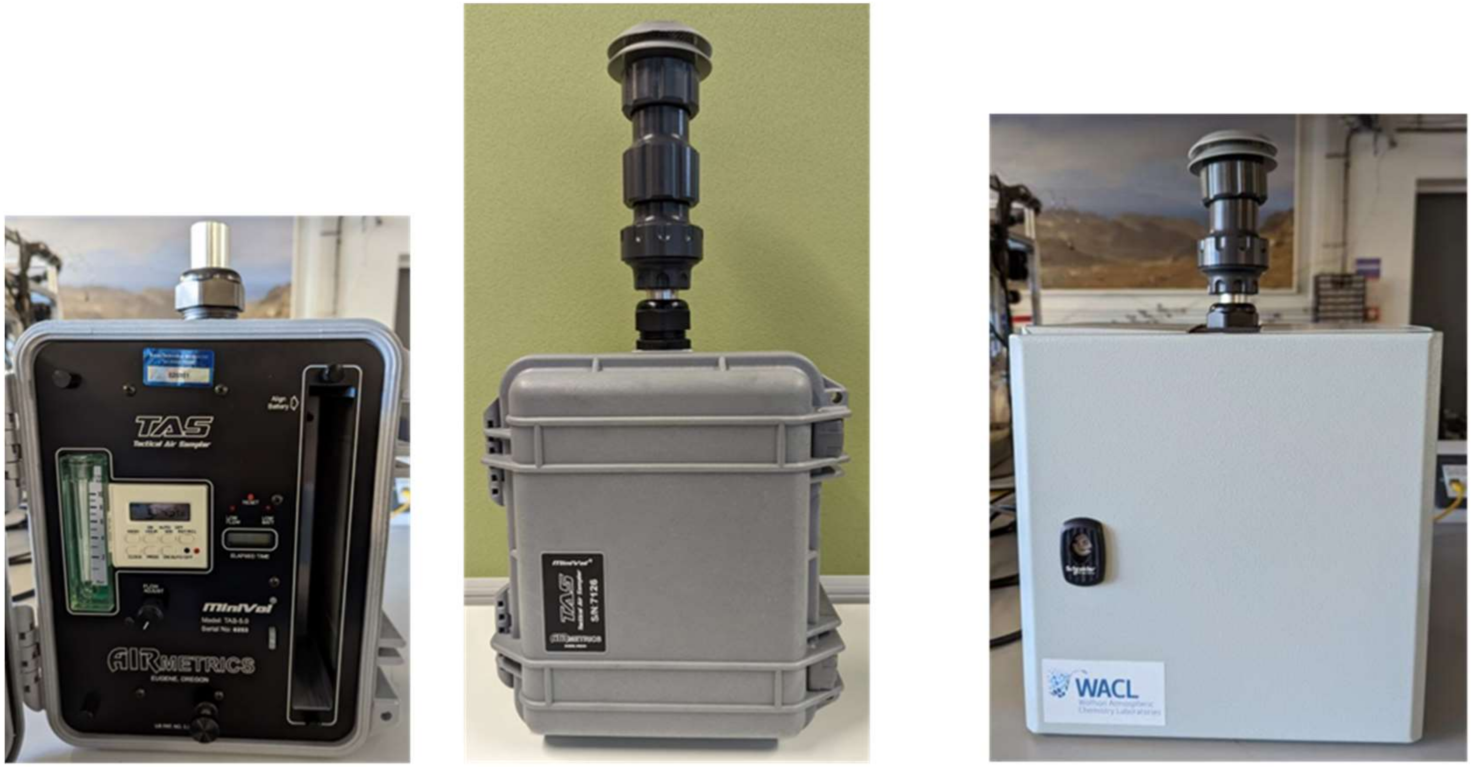

Sampler (inside) Sampler (outside) Sampler in a soundproof box

Supplementary File 1. Survey measurements

INGENIOUS

Survey measurements

V2.0 (17/03/2023)

This document includes: Home survey on Day 1, Building audit on Day 1, and Health and behaviour questionnaire on Day 14.

Contents

Home Survey....2
Section I: Home....2
Section II: Behaviour....7
Building Audit....9
Section I: House....9
Section II: Room....12
Health & Behaviour Questionnaire....15
Section I: Your Perception of Your Home and Air Quality....15
Section II: You/Your Household's Behaviour at Home....16
Section III: Health....23
Section IV: About You and Your Household....29

For peer review only

Home Survey

Note to Research Assistant:

Please enter participant ID and write today's date BEFORE giving the survey to participant.

*Please enter participant ID (i.e. ING…..): ___________

*Please write today's date: ___/___/_____ (dd/mm/yyyy)

*must provide value

For peer review only

Dear Participant,

We are interested to know about your home. We would be grateful if you help us by answering as many of these questions as possible. All the answers you give are confidential.

Thank you for taking part in the INGENIOUS study!

INGENIOUS

We are family BORNINBRADFORD

Section I: Home

This section is about the house you live in at the moment.

1	*How long have you lived at your current home?	Years: _____<br>Months: _____
2	*Does your household own or rent this accommodation?	○ I/we own it or live with the person who owns it (includes homes bought with a mortgage)<br>○ It is rented from the local council

For peer review only - http://bmjopen.bmj.com/site/about/guidelines.xhtml

		○ It is rented from a housing association or housing trust<br>○ It is rented from a private landlord<br>○ Other (e.g. I live here rent free or home comes with job)
3a	*How far away from this home is the nearest greenspace area (e.g. park, playing field, public garden, woodland or other green space), regardless of size and function?	○ Less than 5 min walk<br>○ 5-10 min walk<br>○ 11-20 min walk<br>○ 21-30 min walk<br>○ More than 30 min walk<br>○ Don't know
3b	*Do you have a garden or a yard in your home?	○ Yes<br>○ No<br>○ Don't know
3c	*Is your home nearby (i.e. within 5-min walking distance):<br>(Tick all that apply)	☐ Busy road<br>☐ Motorway<br>☐ Car park<br>☐ Petrol station<br>☐ Industry (e.g. factory)<br>☐ Waste management site<br>☐ Agricultural land<br>☐ Construction<br>☐ Airport<br>☐ Restaurant or takeaway<br>☐ None of the above<br>☐ Don't know
4	*Do you have an attached garage in your home?	○ Yes<br>○ No<br>○ Don't know
5a	*How many *separate* living or dining rooms does your home have?<br>*Only count rooms that are closed off/separated by a door*	_____
5b	*When was the floor of the living or dining room laid (where a sensor will be placed)?	○ In the last 6 months<br>○ 6 months to 1 year ago<br>○ More than 1 year ago<br>○ Don't know
5c	*<u>In the past 12 months</u>, has the interior of the living or dining room (where a sensor will be placed) been refurbished, renovated or painted?	○ Yes<br>○ No<br>○ Don't know
6a	*How many *separate* kitchens does your home have?<br>*Only count rooms that are closed off/separated by a door*	_____

For peer review only - http://bmjopen.bmj.com/site/about/guidelines.xhtml

6b	*When was the floor of the kitchen laid (where a sensor will be placed)?	○ In the last 6 months<br>○ 6 months to 1 year ago<br>○ More than 1 year ago<br>○ Don't know
6c	*Which floor is the kitchen on (where a sensor will be placed)?	○ Property is on one level<br>○ Basement<br>○ Ground floor<br>○ First floor<br>○ Second floor<br>○ Third floor<br>○ Don't know
6d	*In the past 12 months, has the interior of the kitchen (where a sensor will be placed) been refurbished, renovated or painted?	○ Yes<br>○ No<br>○ Don't know
7a	*How many *separate* bedrooms does your home have?<br>*Only count rooms that are closed off/separated by a door*	______
7b	*When was the floor of the bedroom laid (where a sensor will be placed)?	○ In the last 6 months<br>○ 6 months to 1 year ago<br>○ More than 1 year ago<br>○ Don't know
7c	*In the past 12 months, has the interior of the bedroom (where a sensor will be placed) been refurbished, renovated or painted?	○ Yes<br>○ No<br>○ Don't know
7d	*How many people share the bedroom where a sensor will be placed?	______
8a	*How many *separate* bathrooms does your home have?<br>*Only count rooms that are closed off/separated by a door (e.g. toilet)*	______
8b	*Which floors are main (i.e. regularly used) bathrooms on?<br>(Tick all that apply)	☐ Property is on one level<br>☐ Basement<br>☐ Ground floor<br>☐ First floor<br>☐ Second floor<br>☐ Third floor<br>☐ Don't know
9a	*Are you aware of any signs or smells of mould or damp in your home?	○ Yes<br>○ No (Go to Q10)<br>○ Don't know (Go to Q10)
9b	*In which rooms are there signs or smells of mould or damp?<br>(Tick all that apply)	☐ Living/dining room<br>☐ Kitchen

For peer review only - http://bmjopen.bmj.com/site/about/guidelines.xhtml

		☐ Adult's bedroom<br>☐ Child's bedroom<br>☐ Bathroom<br>☐ Basement<br>☐ Other (write in) ____________________
10	*What is the most common type of windows in your whole house?	○ Single glazed windows<br>○ Double or triple glazed windows<br>○ Don't know
11a	*How do you heat your home?<br>(Tick all that apply)	☐ Gas central heating<br>☐ Oil central heating<br>☐ Fixed gas fire<br>☐ Fixed electric heating<br>☐ Night storage heaters<br>☐ Open fires<br>☐ Wood stoves (solid fuel/wood/coal)<br>☐ Pellet boiler<br>☐ Portable heaters: electric, bottled gas/paraffin, oil-filled<br>☐ Other (write in) ____________________<br>☐ Don't know
12a	*What fuel do you use for cooking?<br>(Tick all that apply)	☐ Gas mains<br>☐ Gas bottles<br>☐ Electricity<br>☐ Other (write in) ____________________<br>☐ Don't know

The next question is about ventilation options (please see the photo as examples).

For peer review only

For peer review only - http://bmjopen.bmj.com/site/about/guidelines.xhtml

Extractor hood:

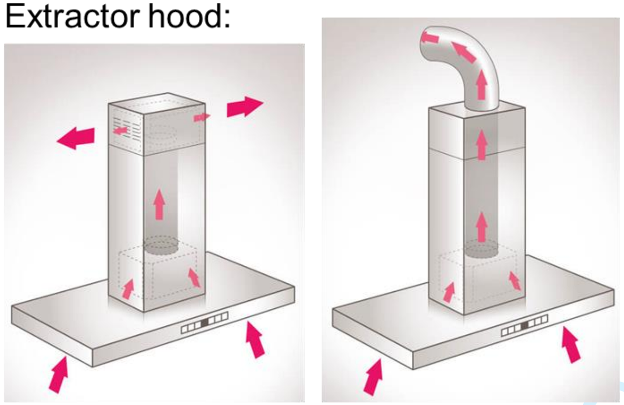

(to inside) (to outside)

Extractor fan:

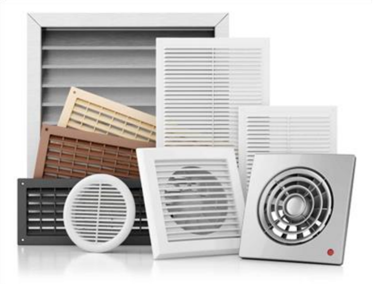

Trickle vents:

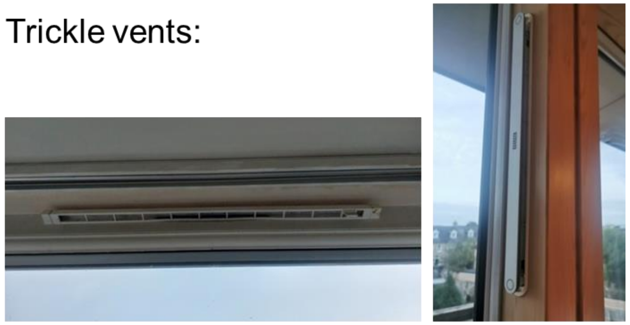

For peer review only

13a	*Does your home have trickle vents?	○ Yes<br>○ No (Go to Q15)<br>○ Don't know (Go to Q15)
13b	*Are the trickle vents open or closed?	○ Trickle vents are always open<br>○ Trickle vents are always closed<br>○ Trickle vents are open or closed depending on the season<br>○ Don't know
14a	*What options are available for ventilating the kitchen? (Tick all that apply)	☐ Extractor hood to inside<br>☐ Extractor hood/fan to outside<br>☐ Window<br>☐ Door to outside<br>☐ Trickle vent<br>☐ There is no means of ventilation<br>☐ Don't know
14b	*What options are available for ventilating the main bathroom/toilet? (Tick all that apply)	☐ Extractor fan<br>☐ Window<br>☐ Trickle vent<br>☐ There is no means of ventilation<br>☐ Don't know
15	*On a normal day, how often are doors to the outside opened and closed?	○ 0-2 times<br>○ 3-10 times<br>○ 11-20 times<br>○ More than 20 times<br>○ Don't know

For peer review only - http://bmjopen.bmj.com/site/about/guidelines.xhtml

16	*Does your home have a chimney that is *not blocked off*?<br>*Air and smoke can flow out of the chimney*	○ Yes<br>○ No (including using a 'chimney sheep')<br>○ My home doesn't have a chimney<br>○ Don't know
17	*When are people normally in your home?<br>(Tick all that apply)	☐ During the day / school time (9:00-14:59 [24-hour clock])<br>☐ After school (15:00-17:59 [24-hour clock])<br>☐ Evening (18:00-22:59 [24-hour clock])<br>☐ Night (23:00-8:59 [24-hour clock])
18a	*Are there any pets in your household?	○ Yes<br>○ No (Go to Q19a)<br>○ Don't know (Go to Q19a)
18b	*How many pet(s) do you own?	_____
19a	*Do you have an air purifier in your home?	○ Yes<br>○ No (Go to Q20a)<br>○ Don't know (Go to Q20a)
19b	What brand or model of air purifier do you have?	____________________

Section II: Behaviour

This section is about smoking behaviour.

1a	*How often do people smoke cigarettes, e-cigarettes, cigar or pipes inside this home?	○ Every day<br>○ Multiple times a week<br>○ Once or twice a week<br>○ Less than once a week<br>○ Never<br>○ Don't know
1b	*How often do people smoke cigarettes, e-cigarettes, cigar or pipes within a close area to this home (e.g. courtyard, with window open, stairwell)?	○ Every day<br>○ Multiple times a week<br>○ Once or twice a week<br>○ Less than once a week<br>○ Never<br>○ Don't know

Thank you for participation.

If you have any questions, please contact us: 01274 364474 or borninbradford@bthft.nhs.uk

For peer review only - http://bmjopen.bmj.com/site/about/guidelines.xhtml

For peer review only

For peer review only - http://bmjopen.bmj.com/site/about/guidelines.xhtml

Building Audit

Please complete the audit below.
Thank you!

INGENIOUS

We are family BORNINBRADFORD

*Please enter participant ID (i.e. ING…..): ___________

*Please write today's date: ___/___/_____ (dd/mm/yyyy)

*must provide value

Section I: House

This section is about the property the participant lives in at the moment.

1	*What type of home is this?	○ Detached house<br>○ Semi-detached house<br>○ Bungalow<br>○ Terraced (including end terrace)<br>○ Flat, maisonette or apartment (in a purpose - built block of flats or tenement)<br>○ Flat, maisonette or apartment (part of converted or shared house, including bedsits)<br>○ Flat, maisonette or apartment (part of another converted building, e.g. former school, church or warehouse)<br>○ Flat, maisonette or apartment (in a commercial building, e.g. in an office building, hotel or over a shop)
2	*When was this home built?	○ Before 1914<br>○ 1914-1944

For peer review only

For peer review only - http://bmjopen.bmj.com/site/about/guidelines.xhtml

		○ 1945-1964<br>○ 1965-1980<br>○ 1981-1990<br>○ 1991-2002<br>○ 2003 or later<br>○ Don't know
3	*How many floors does the property have?	_____
4	*Which floor does the participant live on?<br>(-1 = Basement, 0 = Ground floor, 1 = First floor, 2 = Second floor, etc.)<br><br>*If the participant lives on more than one floors, use comma (,) to list all the floors (e.g. -1, 0, 1).*	__________
5	*What is the energy efficiency rating of the building (which is shown in an energy performance certificate [EPC])?<br><br>*If the participant doesn't know the rating, find an energy certificate using postcode via https://www.gov.uk/find-energy-certificate.*	○ A<br>○ B<br>○ C<br>○ D<br>○ E<br>○ F<br>○ G<br>○ The building has not been certified<br>○ Don't know
6a	*Do any of the windows in the living/dining room (where a sensor will be placed) look out mainly onto grey space, including man-made materials (e.g. roads, buildings)?	○ Yes, at least one window has a main view of grey space<br>○ No, they have a mixed view of grey and green space<br>○ No, they have a main view of green space, including parks and gardens (either public or private) regardless of size and function<br>○ Don't have access to living/dining room
6b	*Do any of the windows in the kitchen (where a sensor will be placed) look out mainly onto grey space, including man-made materials (e.g. roads, buildings)?	○ Yes, at least one window has a main view of grey space<br>○ No, they have a mixed view of grey and green space<br>○ No, they have a main view of green space, including parks and gardens (either public or private) regardless of size and function<br>○ Don't have access to kitchen
7	*How many windows (which can be opened) does the property have?	_____
8a	*How many doors lead to the outside?	_____

The next question is about draft excluders (please see the photo as examples).

For peer review only - http://bmjopen.bmj.com/site/about/guidelines.xhtml

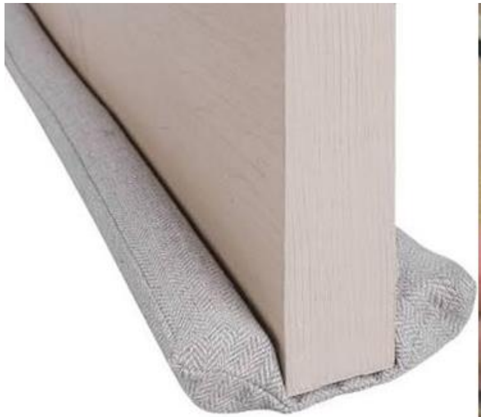
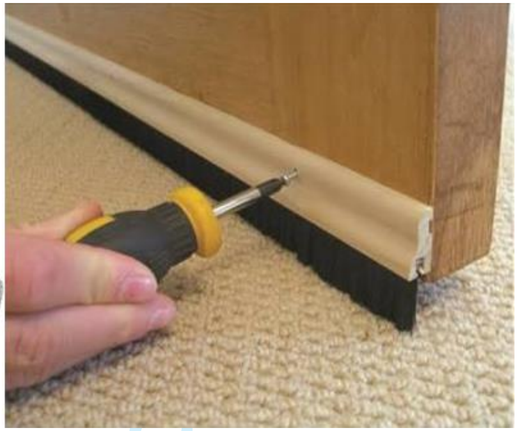

8b	*Are draft excluders attached to outside doors?	○ Yes, all of the doors<br>○ Yes, some of the doors<br>○ No<br>○ Don't have access to outside doors
9a	*Please enter the first sensor's ID:	__________
9b	*Which room is the first sensor installed?	○ Living/dining room<br>○ Kitchen<br>○ Child's bedroom<br>○ Adult's bedroom
9c	*Which floor is the first sensor installed?	○ Basement<br>○ Ground floor<br>○ First floor<br>○ Second floor<br>○ Third floor<br>○ Property is on one level
9d	*Please enter the second sensor's ID:	__________
9e	*Which room is the second sensor installed?	○ Living/dining room<br>○ Kitchen<br>○ Child's bedroom<br>○ Adult's bedroom<br>○ The second sensor is not installed
9f	*Which floor is the second sensor installed?	○ Basement<br>○ Ground floor<br>○ First floor<br>○ Second floor<br>○ Third floor

For peer review only - http://bmjopen.bmj.com/site/about/guidelines.xhtml

		○ Property is on one level<br>○ The second sensor is not installed
9g	*Please enter the third sensor's ID:	__________
9h	*Which room is the third sensor installed?	○ Living/dining room<br>○ Kitchen<br>○ Child's bedroom<br>○ Adult's bedroom<br>○ The third sensor is not installed
9i	*Which floor is the third sensor installed?	○ Basement<br>○ Ground floor<br>○ First floor<br>○ Second floor<br>○ Third floor<br>○ Property is on one level<br>○ The third sensor is not installed

Section II: Room

This section is about the rooms the property has.

1a	What is the dimension of the living/dining room (where a sensor will be placed)?	Length: _____ metre<br>Width: _____ metre<br>Height: _____ metre
1b	*What material is the floor of the living/dining room (where a sensor will be placed)?<br>(Tick all that apply)	☐ Carpet/rug<br>☐ Engineered wood/solid wood<br>☐ Vinyl/laminate<br>☐ Tile<br>☐ There is no living/dining room
1c	*Which direction does the window(s) of the living/dining room face (where a sensor will be placed)?<br>(Tick all that apply if multiple windows face multiple directions)<br><br>*Please round to the nearest cardinal direction.*	☐ North<br>☐ North East<br>☐ East<br>☐ South East<br>☐ South<br>☐ South West<br>☐ West<br>☐ North West<br>☐ There is no window in the living/dining room
1d	*Does the living/dining room (where a sensor will be placed) have curtains/blinds/shutters?	☐ Yes, all of the windows<br>☐ Yes, some of the windows

For peer review only - http://bmjopen.bmj.com/site/about/guidelines.xhtml

		☐ No<br>☐ There is no window in the living/dining room
2a	What is the dimension of the kitchen (where a sensor will be placed)?	Length: _____ metre<br>Width: _____ metre<br>Height: _____ metre
2b	*What material is the floor of the kitchen (where a sensor will be placed)?<br>(Tick all that apply)	☐ Carpet/rug<br>☐ Engineered wood/solid wood<br>☐ Vinyl/laminate<br>☐ Tile<br>☐ There is no kitchen
2c	*Which direction does the window(s) of the kitchen face (where a sensor will be placed)?<br>(Tick all that apply if multiple windows face multiple directions)<br><br>*Please round to the nearest cardinal direction.*	☐ North<br>☐ North East<br>☐ East<br>☐ South East<br>☐ South<br>☐ South West<br>☐ West<br>☐ North West<br>☐ There is no window in the kitchen
2d	*Does the kitchen (where a sensor will be placed) have curtains/blinds/shutters?	☐ Yes, all of the windows<br>☐ Yes, some of the windows<br>☐ No<br>☐ There is no window in the kitchen
2e	*Is the kitchen in open-plan or attached to the living/dining room?	☐ Yes<br>☐ No<br>☐ There is no kitchen
3a	What is the dimension of the bedroom (where a sensor will be placed)?	Length: _____ metre<br>Width: _____ metre<br>Height: _____ metre
3b	*What material is the floor of the bedroom (where a sensor will be placed)?<br>(Tick all that apply)	☐ Carpet/rug<br>☐ Engineered wood/solid wood<br>☐ Vinyl/laminate<br>☐ Tile<br>☐ There is no bedroom
3c	*Which direction does the window(s) of the bedroom face (where a sensor will be placed)?<br>(Tick all that apply if multiple windows face multiple directions)	☐ North<br>☐ North East<br>☐ East<br>☐ South East

For peer review only - http://bmjopen.bmj.com/site/about/guidelines.xhtml

	Please round to the nearest cardinal direction.	☐ South<br>☐ South West<br>☐ West<br>☐ North West<br>☐ There is no window in the bedroom
3d	*Does the bedroom (where a sensor will be placed) have curtains/blinds/shutters?	☐ Yes, all of the windows<br>☐ Yes, some of the windows<br>☐ No<br>☐ There is no window in the bedroom

Thank you for completing the audit.

For peer review only

For peer review only - http://bmjopen.bmj.com/site/about/guidelines.xhtml

Health & Behaviour Questionnaire

Note to Research Assistant:

Please enter participant ID and write today's date BEFORE giving the survey to participant.

*Please enter participant ID (i.e. ING…..): ___________

*Please write today's date: ___/___/_____ (dd/mm/yyyy)

*must provide value

For peer review only

Dear Participant,

We are interested to understand your perception of your home, your/your household's behaviour at home, and your/your child's health and wellbeing. We would be grateful if you could help us by answering as many of these questions as possible. All the answers you give are confidential.

Thank you for taking part in the INGENIOUS study!

INGENIOUS

We are family BORNINBRADFORD

Section I: Your Perception of Your Home and Air Quality

		Very dissatisfied	Fairly dissatisfied	Neither satisfied nor dissatisfied	Fairly satisfied	Very satisfied
1a	*In general, how satisfied or dissatisfied are you with the general condition of your home?	○	○	○	○	○

For peer review only - http://bmjopen.bmj.com/site/about/guidelines.xhtml

1b	*Overall, how satisfied or dissatisfied are you with your home as a place to live?	○	○	○	○	○
1c	*Overall, how satisfied or dissatisfied are you with the quality of the air inside your home?	○	○	○	○	○
		Very poor	Poor	Neither good nor poor	Good	Very good
2a	*How do you rate the quality of the air in your home?	○	○	○	○	○
2b	*How do you rate the quality of the air in your street?	○	○	○	○	○

Section II: You/Your Household's Behaviour at Home

1a	*Do you/people in your household usually have breakfast at home on weekdays?	○ Yes<br>○ No (Go to Q1c)<br>○ Don't know (Go to Q1c)
1b	What time do you/people in your household usually have breakfast at home on weekdays?<br>(24-hour clock: 6:00=6:00 a.m., 18:00=6:00 p.m.)	___:___
1c	*Do you/people in your household usually have breakfast at home on the weekend?	○ Yes<br>○ No (Go to Q1e)<br>○ Don't know (Go to Q1e)
1d	What time do you/people in your household usually have breakfast at home on the weekend?<br>(24-hour clock: 6:00=6:00 a.m., 18:00=6:00 p.m.)	___:___
1e	*Do you/people in your household usually have lunch at home on weekdays?	○ Yes<br>○ No (Go to Q1g)<br>○ Don't know (Go to Q1g)
1f	What time do you/people in your household usually have lunch at home on weekdays?<br>(24-hour clock: 1:00=1:00 a.m., 13:00=1:00 p.m.)	___:___
1g	*Do you/people in your household usually have lunch at home on Saturdays?	○ Yes<br>○ No (Go to Q1i)<br>○ Don't know (Go to Q1i)
1h	What time do you/people in your household usually have lunch at home on Saturdays?<br>(24-hour clock: 1:00=1:00 a.m., 13:00=1:00 p.m.)	___:___
1i	*Do you/people in your household usually have lunch at home on Sundays?	○ Yes<br>○ No (Go to Q1k)<br>○ Don't know (Go to Q1k)

For peer review only - http://bmjopen.bmj.com/site/about/guidelines.xhtml

1j	What time do you/people in your household usually have lunch at home on Sundays?<br>(24-hour clock: 1:00=1:00 a.m., 13:00=1:00 p.m.)	___:___
1k	*Do you/people in your household usually have dinner at home on weekdays?	○ Yes<br>○ No (Go to Q1m)<br>○ Don't know (Go to Q1m)
1l	What time do you/people in your household usually have dinner at home on weekdays?<br>(24-hour clock: 7:00=7:00 a.m., 19:00=7:00 p.m.)	___:___
1m	*Do you/people in your household usually have dinner at home on Saturdays?	○ Yes<br>○ No (Go to Q1o)<br>○ Don't know (Go to Q1o)
1n	What time do you/people in your household usually have dinner at home on Saturdays?<br>(24-hour clock: 7:00=7:00 a.m., 19:00=7:00 p.m.)	___:___
1o	*Do you/people in your household usually have dinner at home on Sundays?	○ Yes<br>○ No (Go to Q2a)<br>○ Don't know (Go to Q2a)
1p	What time do you/people in your household usually have dinner at home on Sundays?<br>(24-hour clock: 7:00=7:00 a.m., 19:00=7:00 p.m.)	___:___

Please select the answer that best describes your household's activities of each over the last 2 weeks:

2a	Has your household used non-stick cookware?	○ Yes, every day<br>○ Yes, multiple times a week<br>○ Yes, once or twice a week<br>○ Yes, less than once a week<br>○ No, we don't use non-stick pans (Go to Q3)<br>○ Don't know (Go to Q3)
2b	*How old is your non-stick cookware?	○ Under 6 months<br>○ Between 6 months and 1 year<br>○ More than 1 year<br>○ Don't know
3a	*Have you/people in your household used hair sprays or deodorant sprays daily?	○ Yes<br>○ No (Go to Q4a)<br>○ Don't know (Go to Q4a)
3b	*How many people in your household have used hair sprays or deodorant sprays daily?	_____

For peer review only - http://bmjopen.bmj.com/site/about/guidelines.xhtml

4a	*Which home fragrances does your household use or burn? (Tick all that apply)	☐ Candle<br>☐ Oud<br>☐ Uunsi/dabqaad/incense burner<br>☐ Incense sticks<br>☐ Reed diffusers<br>☐ Oil diffusers<br>☐ Air fresheners (including plug in)<br>☐ Other (write in) ___________________<br>☐ No (we don't use or burn home fragrances) (Go to Q5a)<br>☐ Don't know (Go to Q5a)
4c	How many times a week, on average, does your household use or burn any of the following home fragrances?<br><br>*Candle, oud, uunsi/dabqaad/incense burner incense sticks, reed diffusers, oil diffusers, air fresheners (including plug-in), other*	_____

		Every day	Multiple times a week	Once or twice a week	Less than once a week	Never	Don't know
5a	*How often does your household use spray surface/floor/kitchen/bathroom cleaners?	○	○	○	○	○	○
5b	*How often does your household use furniture or other spray polishes?	○	○	○	○	○	○
5c	*How often does your household use liquid surface/floor/kitchen/bathroom cleaners?	○	○	○	○	○	○
5d	*How often does your household use bleach?	○	○	○	○	○	○
6	*How often is your home vacuumed?	○	○	○	○	○	○
7a	*How often do you (or someone else in the household) dust your home?	○	○	○	○	○ (Go to Q8a)	○ (Go to Q8a)

7b	*Which is the main method used for dusting?	○ Wet wipe<br>○ Dry wipe<br>○ Brushing off/using a feather duster<br>○ Using a microfibre duster<br>○ Hoover/vacuum cleaner<br>○ Don't know
8a	*Do you do laundry (including washing and drying clothes) in your home?	○ Yes, washing machine only<br>○ Yes, washing machine and tumble dryer<br>○ Yes, other (write in) ___________________<br>○ No (Go to Q9a)<br>○ Don't know (Go to Q9a)

For peer review only - http://bmjopen.bmj.com/site/about/guidelines.xhtml

8c	*Which room do you use for washing your clothes in the home?	○ In the kitchen (with a sensor)<br>○ In the utility room<br>○ In the bathroom<br>○ Other (write in) ____________________<br>○ Don't know
8e	*Where/How do you dry your clothes?<br>(Tick all that apply)	☐ In the kitchen (with a sensor)<br>☐ In the living/dining room (with a sensor)<br>☐ In the bedroom (with a sensor)<br>☐ In the bedroom (without a sensor)<br>☐ In the bathroom<br>☐ Outside<br>☐ Using a tumble dryer<br>☐ Other (write in) ____________________<br>☐ Don't know
9a	*Does your household use a wood stove (solid fuel/wood/coal) or an open fire?	○ Yes<br>○ No (Go to Section III: Health, Q1a)
9b	*How many solid fuel appliances do you use?	_____

Please answer the following questions for each appliance separately.

If you use more than 3 solid fuel appliances, please answer for the most-commonly-used 3 appliances (i.e. Appliances 1, 2 and 3).

9c	*What type of solid fuel appliance is *Appliance 1*?	○ Wood or coal burning stoves (solid fuel/wood/coal)<br>○ Open fires (Go to Q9g)<br>○ Biomass boiler (Go to Q9g)<br>○ Other (write in) ____________________ (Go to Q9g)
9e	What make or model (if known) of stove is *Appliance 1*?	____________________
9f	*Is *Appliance 1* *DEFRA*-approved and/or Eco Design Ready?<br>*Department for Environment, Food and Rural Affairs*	○ Yes<br>○ No<br>○ Don't know
9g	*Did you purchase or install *Appliance 1*, or was it there when you moved in?	○ I purchased/installed this appliance<br>○ The appliance was there when I moved in (Go to Q9j)<br>○ Don't know (Go to Q9j)
9h	*What considerations did you have in mind when deciding which wood or coal burning stove to purchase?<br>(Tick all that apply)	☐ Emission rates<br>☐ Environmental impact<br>☐ Best suited for our home and/or needs<br>☐ Price<br>☐ Recommendation<br>☐ The look of the stove<br>☐ Other (write in) ____________________

For peer review only - http://bmjopen.bmj.com/site/about/guidelines.xhtml

		☐ Don't know
9j	*Did you get any training on using *Appliance 1* when it was installed or when you moved in?	○ Yes<br>○ No, I didn't get given any training or information (Go to Q9m)<br>○ No, I didn't get given any training or information, but I sought information/advice independently (Go to Q9m)<br>○ Don't know (Go to Q9m)
9k	*What training on using *Appliance 1* did you get?<br>(Tick all that apply)	☐ Written instructions<br>☐ Video instructions (e.g. an online guidance video)<br>☐ Someone demonstrated in person<br>☐ Other (write in) ____________________
9m	*When was your chimney or flue for *Appliance 1* last cleaned or maintained?<br>(Tick all that apply)	☐ Professionally cleaned in the last 6 months<br>☐ Professionally cleaned in the last 12 months<br>☐ Cleaned or maintained by someone in the household in the last 6 months<br>☐ Cleaned or maintained by someone in the household in the last 12 months<br>☐ Has not been cleaned or maintained in more than a year<br>☐ Not relevant to this solid fuel appliance<br>☐ Don't know
9n	*What type of fuel do you use in *Appliance 1*?<br>(Tick all that apply)	☐ House coal<br>☐ Smokeless coal<br>☐ Other type of coal<br>☐ Unseasoned wood<br>☐ Seasoned wood<br>☐ Waste wood (e.g. old furniture, pallets or fence panels)<br>☐ Rubbish<br>☐ Manufactured log briquettes<br>☐ Peat<br>☐ Wood pellets<br>☐ Other (write in) ____________________<br>☐ Don't know
9p	*What type of solid fuel appliance is *Appliance 2*?	○ Wood or coal burning stoves (solid fuel/wood/coal)<br>○ Open fires (Go to Q9t)<br>○ Biomass boiler (Go to Q9t)<br>○ Other (write in) ____________________ (Go to Q9t)
9r	What make or model (if known) of stove is *Appliance 2*?	____________________
9s	*Is *Appliance 2* *DEFRA*-approved and/or Eco Design Ready?<br>*Department for Environment, Food and Rural Affairs*	○ Yes<br>○ No<br>○ Don't know

For peer review only - http://bmjopen.bmj.com/site/about/guidelines.xhtml

9t	*Did you purchase or install *Appliance 2*, or was it there when you moved in?	○ I purchased/installed this appliance<br>○ The appliance was there when I moved in (Go to Q9w)<br>○ Don't know (Go to Q9w)
9u	*What considerations did you have in mind when deciding which wood or coal burning stove to purchase?<br>(Tick all that apply)	☐ Emission rates<br>☐ Environmental impact<br>☐ Best suited for our home and/or needs<br>☐ Price<br>☐ Recommendation<br>☐ The look of the stove<br>☐ Other (write in) ___________________<br>☐ Don't know
9w	*Did you get any training on using *Appliance 2* when it was installed or when you moved in?	○ Yes<br>○ No, I didn't get given any training or information (Go to Q9z)<br>○ No, I didn't get given any training or information, but I sought information/advice independently (Go to Q9z)<br>○ Don't know (Go to Q9z)
9x	*What training on using *Appliance 2* did you get?<br>(Tick all that apply)	☐ Written instructions<br>☐ Video instructions (e.g. an online guidance video)<br>☐ Someone demonstrated in person<br>☐ Other (write in) ___________________
9z	*When was your chimney or flue for *Appliance 2* last cleaned or maintained?<br>(Tick all that apply)	☐ Professionally cleaned in the last 6 months<br>☐ Professionally cleaned in the last 12 months<br>☐ Cleaned or maintained by someone in the household in the last 6 months<br>☐ Cleaned or maintained by someone in the household in the last 12 months<br>☐ Has not been cleaned or maintained in more than a year<br>☐ Not relevant to this solid fuel appliance<br>☐ Don't know
9aa	*What type of fuel do you use in *Appliance 2*?<br>(Tick all that apply)	☐ House coal<br>☐ Smokeless coal<br>☐ Other type of coal<br>☐ Unseasoned wood<br>☐ Seasoned wood<br>☐ Waste wood (e.g. old furniture, pallets or fence panels)<br>☐ Rubbish<br>☐ Manufactured log briquettes<br>☐ Peat<br>☐ Wood pellets

For peer review only - http://bmjopen.bmj.com/site/about/guidelines.xhtml

		☐ Other (write in) ____________________<br>☐ Don't know
9ac	*What type of solid fuel appliance is *Appliance 3*?	○ Wood or coal burning stoves (solid fuel/wood/coal)<br>○ Open fires (Go to Q9ag)<br>○ Biomass boiler (Go to Q9ag)<br>○ Other (write in) ____________________ (Go to Q9ag)
9ae	What make or model (if known) of stove is *Appliance 3*?	____________________
9af	*Is *Appliance 3* *DEFRA*-approved and/or Eco Design Ready?<br>*Department for Environment, Food and Rural Affairs*	○ Yes<br>○ No<br>○ Don't know
9ag	*Did you purchase or install *Appliance 3*, or was it there when you moved in?	○ I purchased/installed this appliance<br>○ The appliance was there when I moved in (Go to Q9aj)<br>○ Don't know (Go to Q9aj)
9ah	*What considerations did you have in mind when deciding which wood or coal burning stove to purchase?<br>(Tick all that apply)	☐ Emission rates<br>☐ Environmental impact<br>☐ Best suited for our home and/or needs<br>☐ Price<br>☐ Recommendation<br>☐ The look of the stove<br>☐ Other (write in) ____________________<br>☐ Don't know
9aj	*Did you get any training on using *Appliance 3* when it was installed or when you moved in?	○ Yes<br>○ No, I didn't get given any training or information (Go to Q9am)<br>○ No, I didn't get given any training or information, but I sought information/advice independently (Go to Q9am)<br>○ Don't know (Go to Q9am)
9ak	*What training on using *Appliance 3* did you get?<br>(Tick all that apply)	☐ Written instructions<br>☐ Video instructions (e.g. an online guidance video)<br>☐ Someone demonstrated in person<br>☐ Other (write in) ____________________
9am	*When was your chimney or flue for *Appliance 3* last cleaned or maintained?<br>(Tick all that apply)	☐ Professionally cleaned in the last 6 months<br>☐ Professionally cleaned in the last 12 months<br>☐ Cleaned or maintained by someone in the household in the last 6 months<br>☐ Cleaned or maintained by someone in the household in the last 12 months<br>☐ Has not been cleaned or maintained in more than a year<br>☐ Not relevant to this solid fuel appliance

For peer review only - http://bmjopen.bmj.com/site/about/guidelines.xhtml

		☐ Don't know
9an	*What type of fuel do you use in *Appliance 3*? (Tick all that apply)	☐ House coal<br>☐ Smokeless coal<br>☐ Other type of coal<br>☐ Unseasoned wood<br>☐ Seasoned wood<br>☐ Waste wood (e.g. old furniture, pallets or fence panels)<br>☐ Rubbish<br>☐ Manufactured log briquettes<br>☐ Peat<br>☐ Wood pellets<br>☐ Other (write in) ____________________<br>☐ Don't know
9ap	*Are you aware of government regulations regarding burning authorised fuels and exempt appliances?	○ Yes, and I'm familiar with the content of the regulations<br>○ Yes, but I'm not familiar with the content of the regulations<br>○ No, but I know what an exempt appliance is<br>○ No, but I know what authorised fuels are<br>○ No, and I don't know what either authorised fuels or exempt appliances are
9aq	*Do you live in a smoke control area?	○ Yes<br>○ No<br>○ I know what a smoke control area is, but I don't know if I live in one<br>○ I don't know what a smoke control area is
9ar	*Are you aware of government regulations about smoke coming from chimneys that are not in smoke control areas?	○ Yes<br>○ No, but I know what a smoke control area is<br>○ No, and I don't know what a smoke control area is

Section III: Health

This section is about your/your BiB child's health and wellbeing.

Please give your answers on YOUR BiB CHILD's health.

0a	How many BiB children do you have?<br><br>If you have more than one BiB child, but would like to answer ONLY one of your BiB children's health & wellbeing questions, please type '1'.	_____<br><br>If you type more than ONE, please ask a BiB researcher for another set of health & wellbeing questions for your other BiB children.
0b	What is your BiB child's date of birth?	___/___/_____ (dd/mm/yyyy)

For peer review only

1a	*Has your BiB child ever had wheezing or whistling in the chest at any time in the past?	○ Yes<br>○ No (Go to Q1f)<br>○ Don't know (Go to Q1f)
1b	*Has your BiB child had wheezing or whistling in the chest in the past 2 weeks?	○ Yes<br>○ No (Go to Q1f)<br>○ Don't know (Go to Q1f)
1c	*How many attacks of wheezing has your BiB child had in the past 2 weeks?	○ None<br>○ 1<br>○ 2<br>○ 3<br>○ 4<br>○ More than 4<br>○ Don't know
1d	*In the past 2 weeks, how often, on average, has your BiB child's sleep been disturbed due to wheezing?	○ Never woken with wheezing<br>○ Less than one night per week<br>○ One or more nights per week<br>○ Don't know
1e	*In the past 2 weeks, has wheezing ever been severe enough to limit your BiB child's speech to only one or two words at a time between breaths?	○ Yes<br>○ No<br>○ Don't know
1f	*Has your BiB child ever had asthma?	○ Yes<br>○ No (Go to Q1h)<br>○ Don't know (Go to Q1h)
1g	*Has your BiB child ever been diagnosed by a doctor as having asthma?	○ Yes<br>○ No<br>○ Don't know
1h	*In the past 2 weeks, has your BiB child's chest sounded wheezy during or after exercise?	○ Yes<br>○ No<br>○ Don't know
1i	*In the past 2 weeks, has your BiB child had a dry cough at night, apart from a cough associated with a cold or chest infection?	○ Yes<br>○ No<br>○ Don't know
1j	*Has your BiB child taken any medicines for asthma or breathing difficulties (chest tightness, shortness of breath) in the past 2 weeks?	○ Yes<br>○ No<br>○ Don't know
2a	*Has your BiB child ever had a problem with sneezing, or a running, or blocked nose when he/she/they DID NOT have a cold or the flu?	○ Yes<br>○ No (Go to Q2f)<br>○ Don't know (Go to Q2f)

For peer review only

2b	*In the past 2 weeks, has your BiB child had a problem with sneezing, or a running, or blocked nose when he/she/they DID NOT have a cold or the flu?	○ Yes<br>○ No (Go to Q2f)<br>○ Don't know (Go to Q2f)
2c	*In the past 2 weeks, has this nose problem been accompanied by itchy-watery eyes?	○ Yes<br>○ No<br>○ Don't know
2d	*In the past 2 weeks, how many days did this nose problem occur?	______
2e	*In the past 2 weeks, how much did this nose problem interfere with your BiB child's daily activities?	○ Not at all<br>○ A little<br>○ A moderate amount<br>○ A lot<br>○ Don't know
2f	*Has your BiB child ever had hayfever?	○ Yes<br>○ No<br>○ Don't know
3a	*Has your BiB child ever had an itchy rash which was coming and going for at least six months?	○ Yes<br>○ No (Go to Q3g)<br>○ Don't know (Go to Q3g)
3b	*Has your BiB child had this itchy rash at any time in the past 2 weeks?	○ Yes<br>○ No (Go to Q3g)<br>○ Don't know (Go to Q3g)
3c	*Has this itchy rash at any time affected any of the following places?<br><br>*The folds of the elbows, behind the knees, in front of the ankles, under the buttocks, or around the neck, ears or eyes*	○ Yes<br>○ No<br>○ Don't know
3d	*At what age did this itchy rash first occur?	○ Under 2 years<br>○ Age 2-4 years<br>○ Age 5 or more<br>○ Don't know
3e	*Has this rash cleared completely at any time during the past 2 weeks?	○ Yes<br>○ No<br>○ Don't know
3f	*In the past 2 weeks, how often, on average, have your BiB child been kept awake at night by this itchy rash?	○ Never in the past 2 weeks<br>○ Less than one night per week<br>○ One or more nights per week<br>○ Don't know
3g	*Has your BiB child ever had eczema?	○ Yes

For peer review only

For peer review only - http://bmjopen.bmj.com/site/about/guidelines.xhtml

○ No
○ Don't know

Please give your answers on the basis of YOUR BiB CHILD's behaviour over the last 2 weeks. It would help us if you answered all items as best you can even if you are not absolutely certain.

		Not true	Somewhat true	Certainly true
4a	*Considerate of other people's feelings	○	○	○
4b	*Restless, overactive, cannot stay still for long	○	○	○
4c	*Often complains of headaches, stomach-aches or sickness	○	○	○
4d	*Shares readily with other children (treats, toys, pencils etc.)	○	○	○
4e	*Often has temper tantrums or hot tempers	○	○	○
4f	*Rather solitary, tends to play alone	○	○	○
4g	*Generally obedient, usually does what adults request	○	○	○
4h	*Many worries, often seems worried	○	○	○
4i	*Helpful if someone is hurt, upset or feeling ill	○	○	○
4j	*Constantly fidgeting or squirming	○	○	○
4k	*Has at least one good friend	○	○	○
4l	*Often fights with other children or bullies them	○	○	○
4m	*Often unhappy, down-hearted or tearful	○	○	○
4n	*Generally liked by other children	○	○	○
4o	*Easily distracted, concentration wanders	○	○	○
4p	*Nervous or clingy in new situations, easily loses confidence	○	○	○
4q	*Kind to younger children	○	○	○
4r	*Often lies or cheats	○	○	○
4s	*Picked on or bullied by other children	○	○	○
4t	*Often volunteers to help others (parents, teachers, other children)	○	○	○
4u	*Thinks things out before acting	○	○	○
4v	*Steals from home, school or elsewhere	○	○	○
4w	*Gets on better with adults than with other children	○	○	○
4x	*Many fears, easily scared	○	○	○
4y	*Sees tasks through to the end, good attention span	○	○	○

4z *Overall, do you think that your BiB child has difficulties in one or more of the following areas:
○ No (Go to Q5a)
○ Yes – minor difficulties

For peer review only

For peer review only - http://bmjopen.bmj.com/site/about/guidelines.xhtml

	Emotions, concentration, behaviour or being able to get on with other people?	○ Yes – definite difficulties<br>○ Yes – severe difficulties
4aa	*How long have these difficulties been present?	○ Less than a month<br>○ 1-5 months<br>○ 6-12 months<br>○ Over a year
4ab	*Do the difficulties upset or distress your BiB child?	○ Not at all<br>○ Only a little<br>○ Quite a lot<br>○ A great deal

	Do the difficulties interfere with your BiB child's everyday life in the following areas?	Not at all	Only a little	Quite a lot	A great deal
4ac	*Home life	○	○	○	○
4ad	*Friendships	○	○	○	○
4ae	*Classroom learning	○	○	○	○
4af	*Leisure activities	○	○	○	○

4ag	*Do the difficulties put a burden on you or the family as a whole?	○ Not at all<br>○ Only a little<br>○ Quite a lot<br>○ A great deal

Please refer to YOUR health (and not to the health of your BiB child).

5a	*Do you <u>ever</u> have trouble with your breathing?	○ Never<br>○ Only rarely<br>○ Repeatedly, but it always gets completely better<br>○ Continuously, so that your breathing is never quite right
5b	*Have you had wheezing or whistling in your chest at any time <u>in the past 2 weeks</u>?	○ Yes<br>○ No (Go to Q5i)
5c	*How many attacks of wheezing have you had <u>in the past 2 weeks</u>?	○ None<br>○ 1<br>○ 2<br>○ 3<br>○ 4<br>○ More than 4
5d	*<u>In the past 2 weeks</u>, how often, on average, has your sleep been disturbed due to wheezing?	○ Never woken with wheezing<br>○ Less than one night per week

For peer review only - http://bmjopen.bmj.com/site/about/guidelines.xhtml

		○ One or more nights per week
5e	*Have you ever been breathless when the wheezing noise was present?	○ Yes<br>○ No
5f	*In the past 2 weeks, how often, on average, has your sleep been disturbed due to shortness of breath?	○ Never<br>○ Less than one night per week<br>○ One or more nights per week
5g	*In the past 2 weeks, how often, on average, has your sleep been disturbed due to coughing?	○ Never<br>○ Less than one night per week<br>○ One or more nights per week
5h	*In the past 2 weeks, has wheezing ever been severe enough to limit your speech to only one or two words at a time between breaths?	○ Yes<br>○ No
5i	*Have you ever had asthma?	○ Yes<br>○ No (Go to Q5o)
5j	*Was your asthma confirmed by a doctor?	○ Yes<br>○ No
5k	*How old were you when you had your first attack of asthma?<br><br>*If you have never had an asthma attack, please type '88'.*<br>*If you don't know the answer, please type '99'.*	_____
5l	*Have you had an asthma attack in the past 2 weeks?	○ Yes<br>○ No
5m	*Have you used any inhaled medicines (e.g. salbutamol) to help your breathing at any time in the past 2 weeks (when you DID NOT have a cold)?	○ Yes<br>○ No
5n	*In the past 2 weeks, how many days was your usual activity (at work or in the home) limited because you had breathing problems?	_____
5o	*Have you ever had hay fever?	○ Yes<br>○ No (Go to Q5q)
5p	*Was your hay fever confirmed by a doctor?	○ Yes<br>○ No
5q	*Have you ever had eczema?	○ Yes<br>○ No (Go to Q6a)
5r	*Was your eczema confirmed by a doctor?	○ Yes<br>○ No

Please tick the box that best describe YOUR experience of each over the last 2 weeks:

For peer review only

For peer review only - http://bmjopen.bmj.com/site/about/guidelines.xhtml

		None of the time	Rarely	Some of the time	Often	All of the time
6a	*I've been feeling optimistic about the future.	○	○	○	○	○
6b	*I've been feeling useful.	○	○	○	○	○
6c	*I've been feeling relaxed.	○	○	○	○	○
6d	*I've been dealing with problems well.	○	○	○	○	○
6e	*I've been thinking clearly.	○	○	○	○	○
6f	*I've been feeling close to other people.	○	○	○	○	○
6g	*I've been able to make up my own mind about things.	○	○	○	○	○

How often have YOU been bothered by any of the following problems over the last 2 weeks?

		Not at all	Several days	More than half the days	Nearly every day
7a	*Little interest or pleasure in doing things	○	○	○	○
7b	*Feeling down, depressed, or hopeless	○	○	○	○
7c	*Trouble falling or staying asleep, or sleeping too much	○	○	○	○
7d	*Feeling tired or having little energy	○	○	○	○
7e	*Poor appetite or overeating	○	○	○	○
7f	*Feeling bad about yourself – or that you are a failure or have let yourself or your family down	○	○	○	○
7g	*Trouble concentrating on things, such as reading the newspaper or watching television	○	○	○	○
7h	*Moving or speaking so slowly that other people could have noticed? Or the opposite – being so fidgety or restless that you have been moving around a lot more than usual	○	○	○	○

For peer review only

Section IV: About You and Your Household

This section is about your socio-economic circumstances.

1a	*How many people including yourself are there in your household?	______
1b	*How many children (aged under 18) are there in your household?	______

Please read each statement below and tell us whether the statement was *often true*, *sometimes true*, or *never true* for you or anyone in your household in the last 12 months:

For peer review only - http://bmjopen.bmj.com/site/about/guidelines.xhtml

		Often true	Sometimes true	Never true	Don't know	Do not wish to answer
2a	*The food that I/we bought just didn't last, and I/we didn't have money to get more.	○	○	○	○	○
2b	*I/we couldn't afford to eat balanced meals.	○	○	○	○	○
2c	*In the last 12 months, did you or other adults in your household ever cut the size of your meals or skip meals because there wasn't enough money for food?	○ Yes<br>○ No (Go to Q2e)<br>○ Don't know (Go to Q2e)<br>○ Do not wish to answer (Go to Q2e)				
2d	*How often did you or other adults cut the size of your meals or skip meals?	○ Almost every month<br>○ Some months but not every month<br>○ Only 1 or 2 months<br>○ Don't know<br>○ Do not wish to answer				
2e	*In the last 12 months, did you or other adults ever eat less than you felt you should because there wasn't enough money for food?	○ Yes<br>○ No<br>○ Don't know<br>○ Do not wish to answer				
2f	*In the last 12 months, were you ever hungry but didn't eat because there wasn't enough money for food?	○ Yes<br>○ No<br>○ Don't know<br>○ Do not wish to answer				
3	*When you are at home on a typical day in winter, are you (and everyone in your household) warm enough?	○ Yes, always<br>○ Yes, sometimes<br>○ No, rarely<br>○ No, never<br>○ Don't know<br>○ Do not wish to answer				
4a	*Which of the following is the main way you keep the inside of your house cool in warm weather?<br>(Tick all that apply)	☐ Natural ventilation or shade (e.g. open windows or doors, using heat absorbing shades)<br>☐ Open outside doors<br>☐ Electronic devices (e.g. fans or air conditioning)<br>☐ Other (write in) ____________________<br>☐ Don't know				
5	*How do you pay for your electricity/gas? (Tick only one)	○ Pre-payment meter<br>○ Regular bill<br>○ Direct Debit<br>○ Included in rent				

For peer review only

		○ Don't know
6a	*How well would you say your household is managing financially these days?<br><br>Would you say you are:	○ Living comfortably<br>○ Doing alright<br>○ Just about getting by<br>○ Finding it quite difficult<br>○ Finding it very difficult<br>○ Do not wish to answer
6b	*Compared to a year ago, how would you say your household is doing financially now?	○ Better off<br>○ Worse off<br>○ About the same<br>○ Do not wish to answer
6c	*How often would you say you have been worried about money during the last few weeks?	○ Almost all the time<br>○ Quite often<br>○ Only sometimes<br>○ Never<br>○ Do not wish to answer
7	Please tell us anything else you would like to comment on in relation to the study.	

Thank you for participation.

If you have any questions, please contact us: xxxxx xxxxxx or borninbradford@bthft.nhs.uk

For peer review only

For peer review only - http://bmjopen.bmj.com/site/about/guidelines.xhtml

Participant ID: ING____________

Health (Week 1)

What is your age? ____ years old **What is your gender?** ☐ Female ☐ Male ☐ Non-binary ☐ Do not wish to answer ☐ Wish to enter own response (____________)

<table>
<tr><th>Please circle one for the following symptoms you had today.
dd/mm/yyyy (ddd) e.g. 09/03/2023 (Thu)</th><th>Day 1
/ /
( )</th><th>Day 2
/ /
( )</th><th>Day 3
/ /
( )</th><th>Day 4
/ /
( )</th><th>Day 5
/ /
( )</th><th>Day 6
/ /
( )</th><th>Day 7
/ /
( )</th></tr>
<tr><td>1. I was at home today.</td><td>YES | NO</td><td>YES | NO</td><td>YES | NO</td><td>YES | NO</td><td>YES | NO</td><td>YES | NO</td><td>YES | NO</td></tr>
<tr><td>If YES, what time? (24 hour clock)</td><td>: – :
: – :
: – :</td><td>: – :
: – :
: – :</td><td>: – :
: – :
: – :</td><td>: – :
: – :
: – :</td><td>: – :
: – :
: – :</td><td>: – :
: – :
: – :</td><td>: – :
: – :
: – :</td></tr>
<tr><td>2. I had breathing difficulties.</td><td>YES | NO</td><td>YES | NO</td><td>YES | NO</td><td>YES | NO</td><td>YES | NO</td><td>YES | NO</td><td>YES | NO</td></tr>
<tr><td>If YES, what time? (24 hour clock)</td><td>: – :</td><td>: – :</td><td>: – :</td><td>: – :</td><td>: – :</td><td>: – :</td><td>: – :</td></tr>
<tr><td>3. I had wheezing in the chest.</td><td>YES | NO</td><td>YES | NO</td><td>YES | NO</td><td>YES | NO</td><td>YES | NO</td><td>YES | NO</td><td>YES | NO</td></tr>
<tr><td>If YES, what time? (24 hour clock)</td><td>: – :</td><td>: – :</td><td>: – :</td><td>: – :</td><td>: – :</td><td>: – :</td><td>: – :</td></tr>
<tr><td>4. I had shortness of breath.</td><td>YES | NO</td><td>YES | NO</td><td>YES | NO</td><td>YES | NO</td><td>YES | NO</td><td>YES | NO</td><td>YES | NO</td></tr>
<tr><td>If YES, what time? (24 hour clock)</td><td>: – :</td><td>: – :</td><td>: – :</td><td>: – :</td><td>: – :</td><td>: – :</td><td>: – :</td></tr>
<tr><td>5. I had chest tightness.</td><td>YES | NO</td><td>YES | NO</td><td>YES | NO</td><td>YES | NO</td><td>YES | NO</td><td>YES | NO</td><td>YES | NO</td></tr>
<tr><td>If YES, what time? (24 hour clock)</td><td>: – :</td><td>: – :</td><td>: – :</td><td>: – :</td><td>: – :</td><td>: – :</td><td>: – :</td></tr>
<tr><td>6. I had chest pain.</td><td>YES | NO</td><td>YES | NO</td><td>YES | NO</td><td>YES | NO</td><td>YES | NO</td><td>YES | NO</td><td>YES | NO</td></tr>
<tr><td>If YES, what time? (24 hour clock)</td><td>: – :</td><td>: – :</td><td>: – :</td><td>: – :</td><td>: – :</td><td>: – :</td><td>: – :</td></tr>
<tr><td>7. I had a dry cough when I did not have a cold or the flu.</td><td>YES | NO</td><td>YES | NO</td><td>YES | NO</td><td>YES | NO</td><td>YES | NO</td><td>YES | NO</td><td>YES | NO</td></tr>
<tr><td>If YES, what time? (24 hour clock)</td><td>: – :</td><td>: – :</td><td>: – :</td><td>: – :</td><td>: – :</td><td>: – :</td><td>: – :</td></tr>
<tr><td>8. I had a problem with sneezing when I did not have a cold or the flu.</td><td>YES | NO</td><td>YES | NO</td><td>YES | NO</td><td>YES | NO</td><td>YES | NO</td><td>YES | NO</td><td>YES | NO</td></tr>
<tr><td>If YES, what time? (24 hour clock)</td><td>: – :</td><td>: – :</td><td>: – :</td><td>: – :</td><td>: – :</td><td>: – :</td><td>: – :</td></tr>
<tr><td>9. I had a runny nose when I did not have a cold or the flu.</td><td>YES | NO</td><td>YES | NO</td><td>YES | NO</td><td>YES | NO</td><td>YES | NO</td><td>YES | NO</td><td>YES | NO</td></tr>
<tr><td>If YES, what time? (24 hour clock)</td><td>: – :</td><td>: – :</td><td>: – :</td><td>: – :</td><td>: – :</td><td>: – :</td><td>: – :</td></tr>
<tr><td>10. I had a blocked nose when I did not have a cold or the flu.</td><td>YES | NO</td><td>YES | NO</td><td>YES | NO</td><td>YES | NO</td><td>YES | NO</td><td>YES | NO</td><td>YES | NO</td></tr>
<tr><td>If YES, what time? (24 hour clock)</td><td>: – :</td><td>: – :</td><td>: – :</td><td>: – :</td><td>: – :</td><td>: – :</td><td>: – :</td></tr>
<tr><td>11. I had itchy-watery eyes when I did not have a cold or the flu.</td><td>YES | NO</td><td>YES | NO</td><td>YES | NO</td><td>YES | NO</td><td>YES | NO</td><td>YES | NO</td><td>YES | NO</td></tr>
<tr><td>If YES, what time? (24 hour clock)</td><td>: – :</td><td>: – :</td><td>: – :</td><td>: – :</td><td>: – :</td><td>: – :</td><td>: – :</td></tr>
<tr><td>12. I had an itchy rash.</td><td>YES | NO</td><td>YES | NO</td><td>YES | NO</td><td>YES | NO</td><td>YES | NO</td><td>YES | NO</td><td>YES | NO</td></tr>
<tr><td>If YES, what time? (24 hour clock)</td><td>: – :</td><td>: – :</td><td>: – :</td><td>: – :</td><td>: – :</td><td>: – :</td><td>: – :</td></tr>
<tr><td>13. I had dry skin.</td><td>YES | NO</td><td>YES | NO</td><td>YES | NO</td><td>YES | NO</td><td>YES | NO</td><td>YES | NO</td><td>YES | NO</td></tr>
<tr><td>If YES, what time? (24 hour clock)</td><td>: – :</td><td>: – :</td><td>: – :</td><td>: – :</td><td>: – :</td><td>: – :</td><td>: – :</td></tr>
<tr><td>At the moment I feel: (0='sad' ☹ – 10='happy' ☺)</td><td>___ (0-10)</td><td>___ (0-10)</td><td>___ (0-10)</td><td>___ (0-10)</td><td>___ (0-10)</td><td>___ (0-10)</td><td>___ (0-10)</td></tr>
</table>

Note:

For peer review only - http://bmjopen.bmj.com/site/about/guidelines.xhtml

Participant ID: ING____________

Page 2

Health (Week 2)

<table>
<tr><th>Please circle one for the following symptoms you had today.
dd/mm/yyyy (ddd) e.g. 09/03/2023 (Thu)</th><th>Day 8
/ /
( )</th><th>Day 9
/ /
( )</th><th>Day 10
/ /
( )</th><th>Day 11
/ /
( )</th><th>Day 12
/ /
( )</th><th>Day 13
/ /
( )</th><th>Day 14
/ /
( )</th></tr>
<tr><td>1. I was at home today.
If YES, what time? (24 hour clock)</td><td>YES | NO
: – :
: – :
: – :</td><td>YES | NO
: – :
: – :
: – :</td><td>YES | NO
: – :
: – :
: – :</td><td>YES | NO
: – :
: – :
: – :</td><td>YES | NO
: – :
: – :
: – :</td><td>YES | NO
: – :
: – :
: – :</td><td>YES | NO
: – :
: – :
: – :</td></tr>
<tr><td>2. I had breathing difficulties.
If YES, what time? (24 hour clock)</td><td>YES | NO
: – :</td><td>YES | NO
: – :</td><td>YES | NO
: – :</td><td>YES | NO
: – :</td><td>YES | NO
: – :</td><td>YES | NO
: – :</td><td>YES | NO
: – :</td></tr>
<tr><td>3. I had wheezing in the chest.
If YES, what time? (24 hour clock)</td><td>YES | NO
: – :</td><td>YES | NO
: – :</td><td>YES | NO
: – :</td><td>YES | NO
: – :</td><td>YES | NO
: – :</td><td>YES | NO
: – :</td><td>YES | NO
: – :</td></tr>
<tr><td>4. I had shortness of breath.
If YES, what time? (24 hour clock)</td><td>YES | NO
: – :</td><td>YES | NO
: – :</td><td>YES | NO
: – :</td><td>YES | NO
: – :</td><td>YES | NO
: – :</td><td>YES | NO
: – :</td><td>YES | NO
: – :</td></tr>
<tr><td>5. I had chest tightness.
If YES, what time? (24 hour clock)</td><td>YES | NO
: – :</td><td>YES | NO
: – :</td><td>YES | NO
: – :</td><td>YES | NO
: – :</td><td>YES | NO
: – :</td><td>YES | NO
: – :</td><td>YES | NO
: – :</td></tr>
<tr><td>6. I had chest pain.
If YES, what time? (24 hour clock)</td><td>YES | NO
: – :</td><td>YES | NO
: – :</td><td>YES | NO
: – :</td><td>YES | NO
: – :</td><td>YES | NO
: – :</td><td>YES | NO
: – :</td><td>YES | NO
: – :</td></tr>
<tr><td>7. I had a dry cough when I did not have a cold or the flu.
If YES, what time? (24 hour clock)</td><td>YES | NO
: – :</td><td>YES | NO
: – :</td><td>YES | NO
: – :</td><td>YES | NO
: – :</td><td>YES | NO
: – :</td><td>YES | NO
: – :</td><td>YES | NO
: – :</td></tr>
<tr><td>8. I had a problem with sneezing when I did not have a cold or the flu.
If YES, what time? (24 hour clock)</td><td>YES | NO
: – :</td><td>YES | NO
: – :</td><td>YES | NO
: – :</td><td>YES | NO
: – :</td><td>YES | NO
: – :</td><td>YES | NO
: – :</td><td>YES | NO
: – :</td></tr>
<tr><td>9. I had a runny nose when I did not have a cold or the flu.
If YES, what time? (24 hour clock)</td><td>YES | NO
: – :</td><td>YES | NO
: – :</td><td>YES | NO
: – :</td><td>YES | NO
: – :</td><td>YES | NO
: – :</td><td>YES | NO
: – :</td><td>YES | NO
: – :</td></tr>
<tr><td>10. I had a blocked nose when I did not have a cold or the flu.
If YES, what time? (24 hour clock)</td><td>YES | NO
: – :</td><td>YES | NO
: – :</td><td>YES | NO
: – :</td><td>YES | NO
: – :</td><td>YES | NO
: – :</td><td>YES | NO
: – :</td><td>YES | NO
: – :</td></tr>
<tr><td>11. I had itchy-watery eyes when I did not have a cold or the flu.
If YES, what time? (24 hour clock)</td><td>YES | NO
: – :</td><td>YES | NO
: – :</td><td>YES | NO
: – :</td><td>YES | NO
: – :</td><td>YES | NO
: – :</td><td>YES | NO
: – :</td><td>YES | NO
: – :</td></tr>
<tr><td>12. I had an itchy rash.
If YES, what time? (24 hour clock)</td><td>YES | NO
: – :</td><td>YES | NO
: – :</td><td>YES | NO
: – :</td><td>YES | NO
: – :</td><td>YES | NO
: – :</td><td>YES | NO
: – :</td><td>YES | NO
: – :</td></tr>
<tr><td>13. I had dry skin.
If YES, what time? (24 hour clock)</td><td>YES | NO
: – :</td><td>YES | NO
: – :</td><td>YES | NO
: – :</td><td>YES | NO
: – :</td><td>YES | NO
: – :</td><td>YES | NO
: – :</td><td>YES | NO
: – :</td></tr>
<tr><td>At the moment I feel: (0='sad' ☹ – 10='happy' ☺)</td><td>___ (0-10)</td><td>___ (0-10)</td><td>___ (0-10)</td><td>___ (0-10)</td><td>___ (0-10)</td><td>___ (0-10)</td><td>___ (0-10)</td></tr>
</table>

Note:

Weekday

DATE: (please circle one) **Mon** / **Tue** / **Wed** / **Thu** / **Fri** , (please write in) ____/____/______ (dd/mm/yyyy) Participant ID: ING____________ Page 1

PLEASE FILL IN IF APPLICABLE

COOKING

What time did you or someone else in your household do the following activities in your home? (24 hour clock)

Frying	Boiling | Steaming	Baking | Grilling Roasting	Using an extractor hood | fan
: – :	: – :	: – :	: – :
: – :	: – :	: – :	: – :
: – :	: – :	: – :	: – :

CLEANING

What time did you or someone else in your household do the following activities in your home? (24 hour clock)

Vacuuming | Dusting	Using cleaning sprays | furniture polishes
: – :	: – :
: – :	: – :
: – :	: – :

PRODUCTS

What time did you or someone else in your household use the following products in your home? (24 hour clock)

Air freshener sprays Plug-in air fresheners Insecticides | Fly sprays	Candle | Incense | Other fragrance burning sources
: – :	: – :
: – :	: – :
: – :	: – :

HEATING

Were the following heating sources on in your home? (Please circle one each)

Central heating	Central boiler
YES | NO	YES | NO

(YES for timer or temperature control is set)

What time did you or someone else in your household use stoves (solid fuel | wood | coal) or open fires? (24 hour clock)

: – :	: – :
: – :	: – :

VENTILATION

What time did you or someone else in your household open the windows or the doors (leading to outside) in the following rooms (*where a sensor is placed)? (24 hour clock)

Living room*	Kitchen*	Bedroom*	Main bathroom	Use an extractor fan
: – :	: – :	: – :	: – :	: – :
: – :	: – :	: – :	: – :	: – :
: – :	: – :	: – :	: – :	: – :

PERSONAL CARE

What time did you or someone else in your household do the following activities in your home? (24 hour clock)

Taking a shower | bath		Using deodorant | aftershave | perfume | hairspray		Smoking | Using e-cigarettes (in | outside of your house)	
: – :	: – :	: – :	: – :	: – :	: – :
: – :	: – :	: – :	: – :	: – :	: – :

OCCUPANCY

What time were there people at home? How many people?

: – :	___ people	: – :	___ people
: – :	___ people	: – :	___ people
: – :	___ people		

INGENIOUS_Diary (V2.0; 17/03/2023)

INGENIOUS

We are family

DATE: (please circle one) **Mon** / **Tue** / **Wed** / **Thu** / **Fri** , (please write in) ____/____/______ (dd/mm/yyyy) Participant ID: ING___________ Page 2

NOTES:

COOKING

E.g. Lots of smoke from a toaster; Left a frying pan on too long and produced lots of smokes

CLEANING

E.g. Used cleaning sprays on Sun morning (if not recorded in the diary); Used eco friendly or natural cleaning products

PRODUCTS

E.g. Used air fresheners in the living room; Burnt candles in the kitchen

HEATING

E.g. Used portable radiant/electric heaters in the living room & child's bedrooms in the night

PERSONAL CARE

E.g. Smelled smoking/e-cigarettes from neighbours; Used deodorant/perfume in the main bathroom

VENTILATION

E.g. Opened patio and bifold doors to get fresh air; Used an air purifier in the living room; Used an dehumidifier in the bathroom and child's bedroom

OTHER EXPERIENCES RELATED TO AIR QUALITY

E.g. Smelled my neighbour's garden fire from inside of the house; Had drain smells in the bathroom; Smelled something (don't know what it is) from the outside

OCCUPANCY

E.g. No one was at home on Sat; My dog and cat stayed inside of the house

INGENIOUS_Diary (V2.0; 17/03/2023)

INGENIOUS

We are family

DATE: (please circle one) **Sat** / **Sun**, (please write in) ____/____/______ (dd/mm/yyyy) Participant ID: ING____________

COOKING

What time did you or someone else in your household do the following activities in your home? (24 hour clock)

Frying	Boiling | Steaming	Baking | Grilling Roasting	Using an extractor hood | fan
: — :	: — :	: — :	: — :
: — :	: — :	: — :	: — :
: — :	: — :	: — :	: — :

CLEANING

What time did you or someone else in your household do the following activities in your home? (24 hour clock)

Vacuuming | Dusting	Using cleaning sprays | furniture polishes
: — :	: — :
: — :	: — :
: — :	: — :

PRODUCTS

What time did you or someone else in your household use the following products in your home? (24 hour clock)

Air freshener sprays Plug-in air fresheners Insecticides | Fly sprays	Candle | Incense | Other fragrance burning sources
: — :	: — :
: — :	: — :
: — :	: — :

HEATING

Were the following heating sources on in your home? (Please circle one each)

Central heating	Central boiler
YES | NO	YES | NO

(YES for timer or temperature control is set)

What time did you or someone else in your household use stoves (solid fuel | wood | coal) or open fires? (24 hour clock)

: — :	: — :
: — :	: — :

VENTILATION

What time did you or someone else in your household open the windows or the doors (leading to outside) in the following rooms (*where a sensor is placed)? (24 hour clock)

Living room*	Kitchen*	Bedroom*	Main bathroom	Use an extractor fan
: — :	: — :	: — :	: — :	: — :
: — :	: — :	: — :	: — :	: — :
: — :	: — :	: — :	: — :	: — :

PERSONAL CARE

What time did you or someone else in your household do the following activities in your home? (24 hour clock)

Taking a shower | bath		Using deodorant | aftershave | perfume | hairspray		Smoking | Using e-cigarettes (in | outside of your house)	
: — :	: — :	: — :	: — :	: — :	: — :
: — :	: — :	: — :	: — :	: — :	: — :

OCCUPANCY

What time were there people at home? How many people?

: — :	people	: — :	people
: — :	people	: — :	people
: — :	people		

INGENIOUS_Diary (V2.0; 17/03/2023)

INGENIOUS

bib We are family BORNINBRADFORD

BMJ Open

Weekend day

DATE: (please circle one) **Sat** / **Sun**, (please write in) ____/____/______ (dd/mm/yyyy) Participant ID: ING____________ Page 4

NOTES:

COOKING

E.g. Lots of smoke from a toaster; Left a frying pan on too long and produced lots of smokes

CLEANING

E.g. Used cleaning sprays on Sun morning (if not recorded in the diary); Used eco friendly or natural cleaning products

PRODUCTS

E.g. Used air fresheners in the living room; Burnt candles in the kitchen

HEATING

E.g. Used portable radiant/electric heaters in the living room & child's bedrooms in the night

PERSONAL CARE

E.g. Smelled smoking/e-cigarettes from neighbours; Used deodorant/perfume in the main bathroom

VENTILATION

E.g. Opened patio and bifold doors to get fresh air; Used an air purifier in the living room; Used an dehumidifier in the bathroom and child's bedroom

OTHER EXPERIENCES RELATED TO AIR QUALITY

E.g. Smelled my neighbour's garden fire from inside of the house; Had drain smells in the bathroom; Smelled something (don't know what it is) from the outside

OCCUPANCY

E.g. No one was at home on Sat; My dog and cat stayed inside of the house

For peer review only - http://bmjopen.bmj.com/site/about/guidelines.xhtml

INGENIOUS

We are family

Diary for Additional Measurements

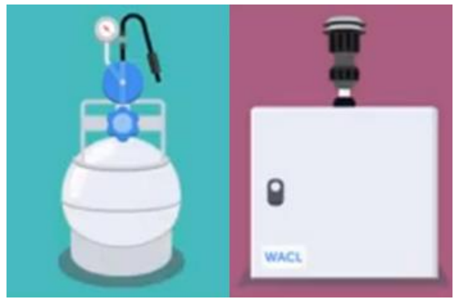

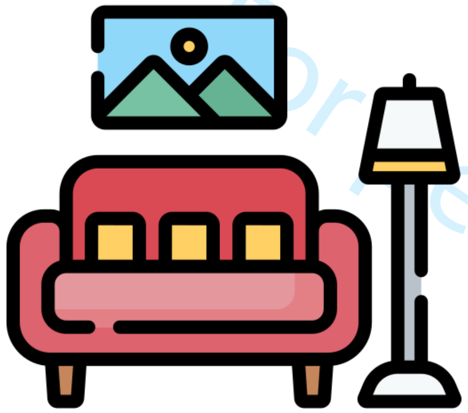

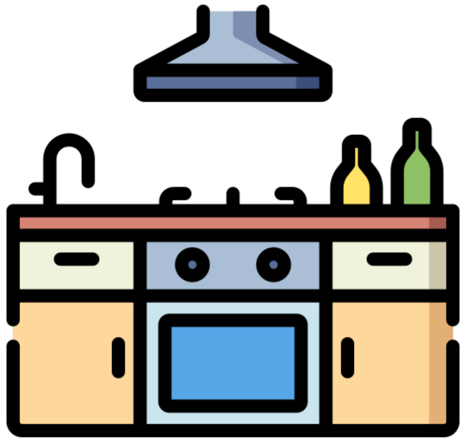

For peer review only

INGENIOUS

We are family
BORNINBRADFORD

BMJ Open

Participant ID: ING___________

Additional Measurements

Cooking (PM sampler)

COOKING

Can you please tell us what you cooked for your main meals on each day of sampling?

[Drop-off] Date: __/__/____

Mon | Tue | Wed | Thu | Fri | Sat | Sun

Did you cook today?

YES | NO

If YES, what did you cook for your main meals today?

Did you cook meat (e.g. beef, lamb, chicken, pork) and/or seafood (including fish) today?

YES | NO

Date: __/__/____

Mon | Tue | Wed | Thu | Fri | Sat | Sun

Did you cook today?

YES | NO

If YES, what did you cook for your main meals today?

Did you cook meat (e.g. beef, lamb, chicken, pork) and/or seafood (including fish) today?

YES | NO

Date: __/__/____

Mon | Tue | Wed | Thu | Fri | Sat | Sun

Did you cook today?

YES | NO

If YES, what did you cook for your main meals today?

Did you cook meat (e.g. beef, lamb, chicken, pork) and/or seafood (including fish) today?

YES | NO

Date: __/__/____

Mon | Tue | Wed | Thu | Fri | Sat | Sun

Did you cook today?

YES | NO

If YES, what did you cook for your main meals today?

Did you cook meat (e.g. beef, lamb, chicken, pork) and/or seafood (including fish) today?

YES | NO

Date: __/__/____

Mon | Tue | Wed | Thu | Fri | Sat | Sun

Did you cook today?

YES | NO

If YES, what did you cook for your main meals today?

Did you cook meat (e.g. beef, lamb, chicken, pork) and/or seafood (including fish) today?

YES | NO

NOTES:

For peer review only

For peer review only - http://bmjopen.bmj.com/site/about/guidelines.xhtml

DATE: (please circle one) **Mon** / **Tue** / **Wed** / **Thu** / **Fri** / **Sat** / **Sun**, (please write in) ____/____/______ (dd/mm/yyyy) Participant ID: ING____________

PLEASE FILL IN IF APPLICABLE

COOKING

What time did you or someone else in your household do the following activities in your home? (24 hour clock)

Frying	Boiling | Steaming	Baking | Grilling Roasting	Using an extractor hood | fan
: — :	: — :	: — :	: — :
: — :	: — :	: — :	: — :
: — :	: — :	: — :	: — :

CLEANING

What time did you or someone else in your household do the following activities in your home? (24 hour clock)

Vacuuming | Dusting	Using cleaning sprays | furniture polishes
: — :	: — :
: — :	: — :
: — :	: — :

PRODUCTS

What time did you or someone else in your household use the following products in your home? (24 hour clock)

Air freshener sprays Plug-in air fresheners Insecticides | Fly sprays	Candle | Incense | Other fragrance burning sources
: — :	: — :
: — :	: — :
: — :	: — :

HEATING

Were the following heating sources on in your home? (Please circle one each)

Central heating	Central boiler
YES | NO	YES | NO

(YES for timer or temperature control is set)

What time did you or someone else in your household use stoves (solid fuel | wood | coal) or open fires? (24 hour clock)

: — :	: — :
: — :	: — :

VENTILATION

What time did you or someone else in your household open the windows or the doors (leading to outside) in the following rooms (*where a sensor is placed)? (24 hour clock)

Living room*	Kitchen*	Bedroom*	Main bathroom	Use an extractor fan
: — :	: — :	: — :	: — :	: — :
: — :	: — :	: — :	: — :	: — :
: — :	: — :	: — :	: — :	: — :

PERSONAL CARE

What time did you or someone else in your household do the following activities in your home? (24 hour clock)

Taking a shower | bath		Using deodorant | aftershave | perfume | hairspray		Smoking | Using e-cigarettes (in | outside of your house)	
: — :	: — :	: — :	: — :	: — :	: — :
: — :	: — :	: — :	: — :	: — :	: — :

OCCUPANCY

What time were there people at home? How many people?

: — :	____ people	: — :	____ people
: — :	____ people	: — :	____ people
: — :	____ people		

For peer review only - http://bmjopen.bmj.com/site/about/guidelines.xhtml

INGENIOUS

We are family

DATE: (please circle one) **Mon** / **Tue** / **Wed** / **Thu** / **Fri** / **Sat** / **Sun**, (please write in) ____/____/______ (dd/mm/yyyy) Participant ID: ING____________ Page 4

NOTES:

COOKING

E.g. Lots of smoke from a toaster; Left a frying pan on too long and produced lots of smokes

CLEANING

E.g. Used cleaning sprays on Sun morning (if not recorded in the diary); Used eco friendly or natural cleaning products

PRODUCTS

E.g. Used air fresheners in the living room; Burnt candles in the kitchen

HEATING

E.g. Used portable radiant/electric heaters in the living room & child's bedrooms in the night

PERSONAL CARE

E.g. Smelled smoking/e-cigarettes from neighbours; Used deodorant/perfume in the main bathroom

VENTILATION

E.g. Opened patio and bifold doors to get fresh air; Used an air purifier in the living room; Used an dehumidifier in the bathroom and child's bedroom

OTHER EXPERIENCES RELATED TO AIR QUALITY

E.g. Smelled my neighbour's garden fire from inside of the house; Had drain smells in the bathroom; Smelled something (don't know what it is) from the outside

OCCUPANCY

E.g. No one was at home on Sat; My dog and cat stayed inside of the house

INGENIOUS We are family BORNINBRADFORD

Indoor air pollution feedback report

Dear [participant],

Thank you for taking part in the INGENIOUS study and providing scientists with important insights into air quality in real homes. Your data will help us understand so much about the air in our homes and how we can improve it.

This is a summary of some of the data we collected in your home using the AirGradient sensors we placed in different rooms (the kitchen, bedroom, and living space). The graphs below show the average results over different time periods, to help highlight where there were peaks. Seeing the times these occur may help you identify things you do that cause these peaks.

Your measurements were taken from [date] to [date] . We measured particulate matter (particles in the air), carbon dioxide (mostly from human breath), and total volatile organic compounds (most of them you can smell).

For peer review

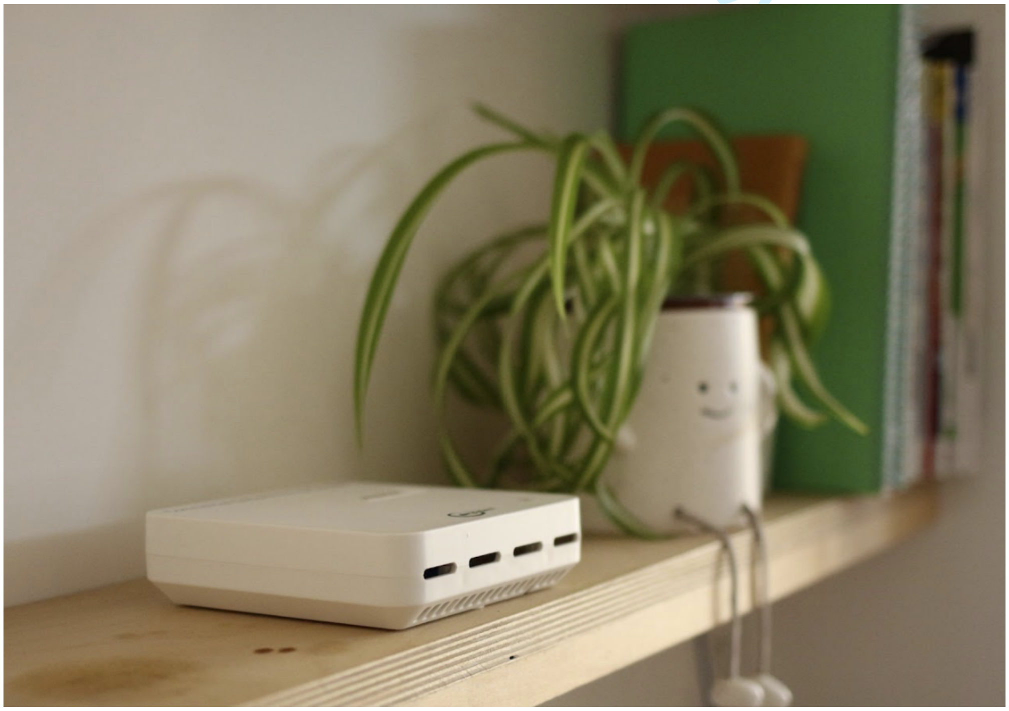

For peer review only - http://bmjopen.bmj.com/site/about/guidelines.xhtml

Particulate Matter in your kitchen

Particulate matter (PM) is made up of particles (tiny pieces) of solids or liquids that are in the air. PM can come from outside, for example dust from the soil or vehicle exhaust. There are also indoor sources of PM, such as cooking. The graphs below show levels of tiny particles in the air that are 2.5 microns or less in diameter ($PM_{2.5}$). They are also called “fine” particles and are believed to pose the greatest risk to health, particularly for people with heart or lung disease (such as asthma or COPD), babies and children, and the elderly.

On the graphs below, you’ll see time along the bottom (x-axis), and the concentration of chemicals up the left (y-axis). Concentrations are typically measured in units of the mass (micrograms µ) of a chemical per volume of air (cubic meter m^3).

```
Daily (average) levels of particulate matter in the kitchen.
 Blue line shows levels we recorded. Dashed grey line shows the
 maximum daily guideline values which have been recommended
 by the World Health Organization.
```

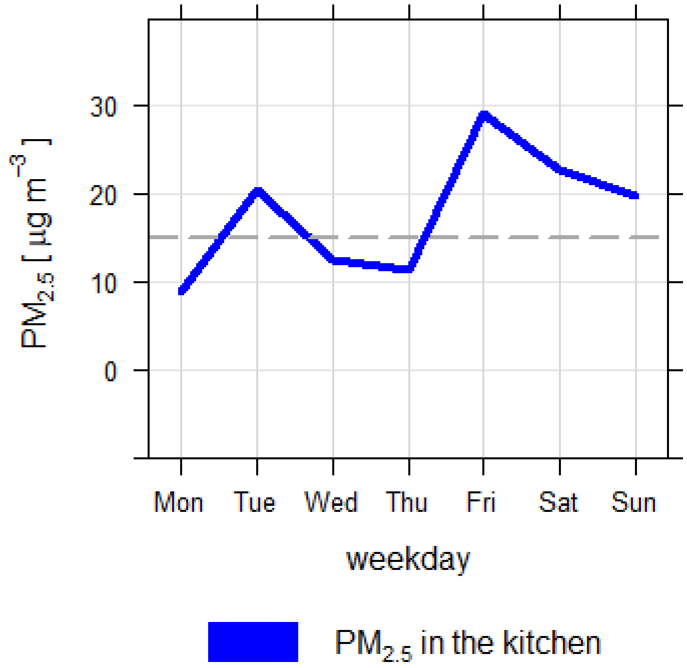

For peer review only - http://bmjopen.bmj.com/site/about/guidelines.xhtml

```
Hourly average levels of particulate matter in the kitchen
during the 2 weeks. Time is shown using the 24 hour clock.
```

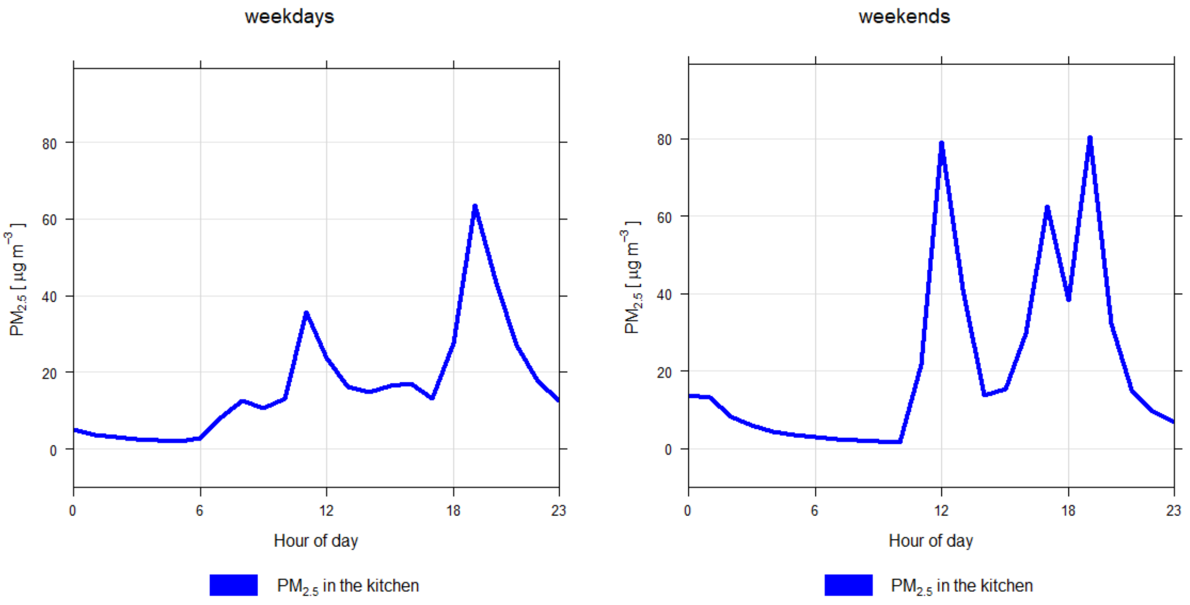

What activities do you think contributed to the peaks seen in these graphs? Think about if there's anything you do differently on certain days, or at different times of day. Activities that could contribute to higher PM levels include cooking, vacuuming or dusting.

If your results are consistently high, you may want to take action to address this, and some suggestions are at the bottom of the document.

Total volatile organic compounds in your living room

Total Volatile Organic Compounds (TVOCs) are a large group of many different chemicals in gas form. They are found in many products we use in our homes. Common indoor sources include personal care products (e.g. deodorants and hair products), paints and cleaning products, smoking and burning wood.

Each chemical has its own toxicity and potential for causing different health effects. Unlike particulate matter, there is no maximum guideline value for TVOCs, and how strong they smell is not a good indicator of

For peer review only - http://bmjopen.bmj.com/site/about/guidelines.xhtml

health risk. High levels of VOCs can cause eye, nose and, throat irritations, nausea, headaches, dizziness and worsening of asthma symptoms. To protect your health, it is best to limit your exposure to products and materials that contain VOCs.

We measured TVOCs using a low cost sensor, which means that the actual measurements may not be accurate. However, the trends in the measurements are accurate, so the graph below shows you when you have peaks in TVOCs.

```
Daily and hourly average levels of total volatile organic compounds
in the living room during the 2 weeks.The time for each day runs fro
shown by the thicker vertical line.
```

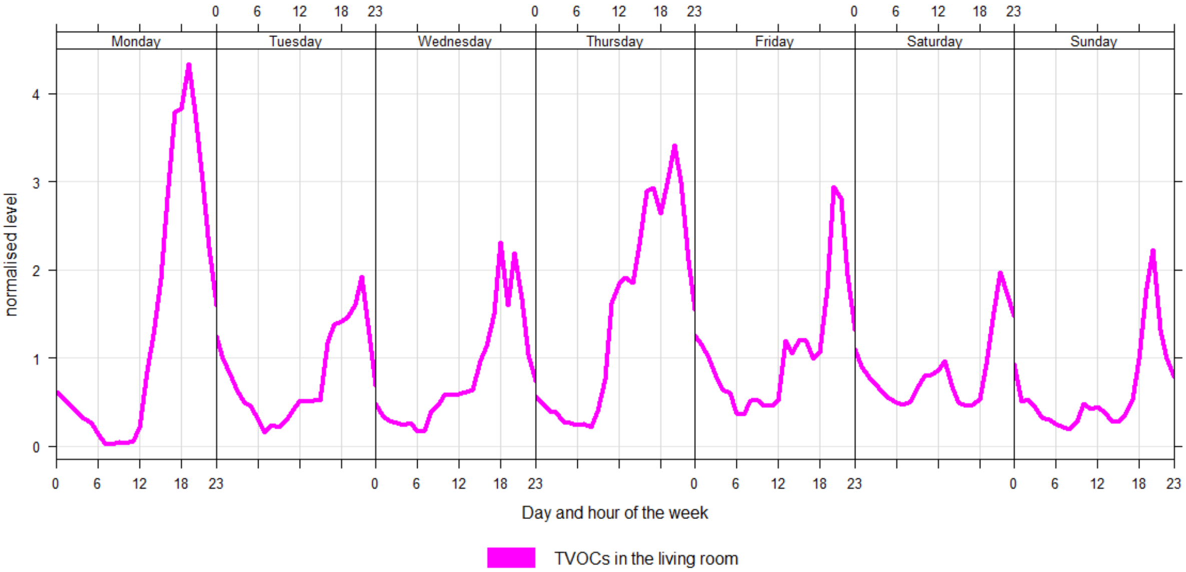

You can see spikes in the TVOCs in the evenings, and sometimes in the morning. What are you doing at that time of day? Is there a link to cooking or cleaning?

Carbon dioxide in your child's bedroom

Carbon dioxide (CO_2) is exhaled in our breath and it is often used as a way to understand how well-ventilated a space is. Studies have shown that high levels may increase the chance of catching virus such as colds, flu, and COVID-19 and also decrease your ability to concentrate.

The graph below shows the hourly average levels of CO_2 in your child's bedroom. It is very likely that levels during the night are higher due to the CO_2 breathed out and lower ventilation rates. These levels are not a cause of serious health concerns. Outdoor CO_2 concentrations are shown in the black dashed line and are usually around 400 ppm (might be a bit higher in urban areas). The unit *parts per million* (ppm) refers to molecules. For example, 400 ppm of CO_2 means that for every million molecules of air, there are 400 molecules of CO_2.

```
Hourly average levels of carbon dioxide levels in the
bedroom during the 2 weeks. Time is shown using the 24 hour clock.
```

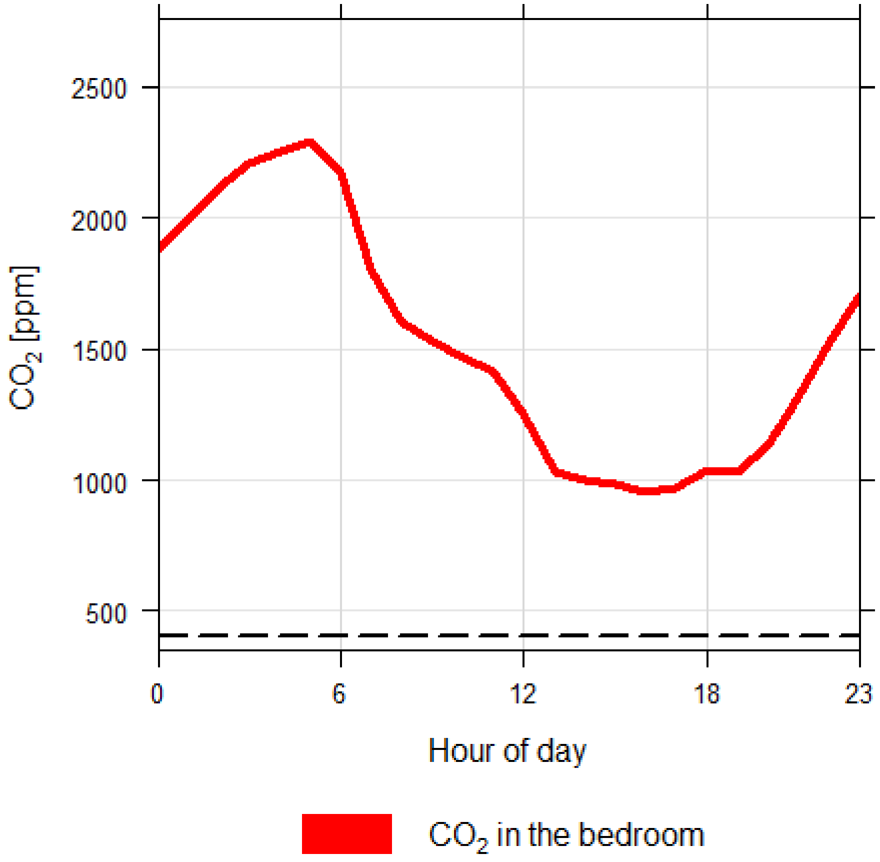

Summary of your indoor air quality

Below we show average levels of particulate matter (in µg/m^3) and CO_2 (in ppm) measured in different rooms over the two week period. The colour

codes give an indication that you may want to consider some actions to improve the indoor air quality in your home.

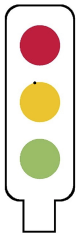

action recommended

could be improved

good

Pollutants	Kitchen	Living_room	Bedroom
$PM_{2.5}$	17	8	18
CO_2	738	749	1527

We have noticed that your maximum PM levels occur at 19 o' clock, most likely during cooking activities. We have also noticed that carbon dioxide concentrations in the bedroom are often quite high exceeding the recommended 1,000 ppm. You may consider ventilating this room more.

Protect yourself and your family

- Even in the cold months, open windows from time to time to allow fresh air to move into the house. This will reduce the levels of many harmful pollutants including PM, TVOCs and CO_2. Aim to open the windows for at least 5 minutes every day, especially if we have found relatively high carbon dioxide levels.
- Use fans/extractors/natural ventilation in the kitchen during cooking to remove fumes, especially if PM levels were above recommended levels.
  Vacuum the carpets and rugs at least once or twice a week to remove
- dust and allergens, especially in rooms with high PM levels. Consider purchasing low-VOC options for paints and furniture. These information
- are displayed on the label of these products.
  Where possible, store unused paints, cleaning products or other
- chemicals in a garage, shed or other place where people do not spend much time. Alternatively, store them in a sealed container.

For peer review only - http://bmjopen.bmj.com/site/about/guidelines.xhtml

We would like to thank you again for your participation. If you have any concerns about these findings, please do not hesitate to contact the BiB Community office at xxxxx xx xxxx

INGENIOUS
Understanding Air Pollution in Homes

[study ID]

For peer review only - http://bmjopen.bmj.com/site/about/guidelines.xhtml
