# Supplementary material for: Understanding the patterns and health impact of indoor air pollutant exposures in Bradford, UK: a study protocol
Source: BMJ Open. 2023 Nov 30;13(12):e081099. doi: 10.1136/bmjopen-2023-081099 (PMC10711829; doi:10.1136/bmjopen-2023-081099)
Supplement: Reviewer comments [file bmjopen-2023-081099.reviewer_comments.pdf]

PEER REVIEW HISTORY

BMJ Open publishes all reviews undertaken for accepted manuscripts. Reviewers are asked to complete a checklist review form (**http://bmjopen.bmj.com/site/about/resources/checklist.pdf**) and are provided with free text boxes to elaborate on their assessment. These free text comments are reproduced below.

ARTICLE DETAILS

**TITLE (PROVISIONAL)**	Understanding the patterns and health impact of indoor air pollutant exposures in Bradford, UK: A study protocol
**AUTHORS**	Ikeda, Erika; Hamilton, Jacqueline; Wood, Chantelle; Chatzidiakou, Lia; Warburton, Thomas; Ruangkanit, Athina; Shao, Yunqi; Genes, Denisa; Waiblinger, Dagmar; Yang, Tiffany; Giorio, Chiara; McFiggans, Gordon; O'Meara, Simon; Edwards, Pete; Bates, Elizabeth; Shaw, David; Jones, Roderic; Carslaw, Nicola; McEachan, Rosemary

This article was not externally reviewed at BMJ Open. Protocols that have been independently assessed prior to submission to BMJ Open are usually fast-tracked to publication on the grounds that further substantial changes will not be possible. This independent assessment will usually be external, independent review for both a substantial grant award from a non-commercial or government funder and ethics approval.
